# A method for detecting outliers in linear-circular non-parametric regression

**DOI:** 10.1371/journal.pone.0286448

**Published:** 2023-06-12

**Authors:** Sümeyra Sert, Filiz Kardiyen

**Affiliations:** 1 Department of Statistics, Selcuk University, Selcuklu, Konya, Turkey; 2 Department of Statistics, Gazi University, Ankara, Turkey; Cairo University, EGYPT

## Abstract

This study proposes a robust outlier detection method based on the circular median for non-parametric linear-circular regression in case the response variable includes outlier(s) and the residuals are Wrapped-Cauchy distributed. Nadaraya-Watson and local linear regression methods were employed to obtain non-parametric regression fits. The proposed method’s performance was investigated by using a real dataset and a comprehensive simulation study with different sample sizes, contamination, and heterogeneity degrees. The method performs quite well in medium and higher contamination degrees, and its performance increases as the sample size and the homogeneity of data increase. In addition, when the response variable of linear-circular regression contains outliers, the Local Linear Estimation method fits the data set better than the Nadaraya Watson method.

## 1. Introduction

Circular (directional) data are measured on directions, angles, and rotations; and are primarily used in engineering, meteorology, ocean science, geography, geology, medicine, and neuroscience ([1–3]). Such data consist of angles measured based on a specific reference point and assumed to be on the unit circle. Due to their geometric structure, conventional statistical methods cannot be applied to circular data. Thus, the need to analyze this type of data has arisen ([1]).

Non-parametric circular regression is a popular research topic. The first implementation for non-parametric circular regression was made by Di Marzio et al. [4]. They defined circular kernel functions and extended the least squares method for the local polynomial regression model. Nadaraya-Watson (NW) and local linear (LL) non-parametric regression models and kernel weight functions were defined by Di Marzio et al. [5] when the response variable was circular. In their study, these methods were examined separately for the models, including linear and circular explanatory variables, respectively. The local trigonometric and Nadaraya-Watson estimators were compared by Oliveira et al. [6] when the explanatory and response variables were circular and linear, respectively. They also obtained the optimal bandwidth value through leave-one-out cross-validation (CV). Besides, Oliveira et al. [7] developed an R package named *NPCirc* for non-parametric density estimation and regression analysis and extended it in [8]. Xu [9] developed a non-parametric smoothing method to estimate the periodic functions of both circular density estimation and linear-circular non-parametric regression. Sikaroudi and Park [10] presented a mixture of linear-linear regression models as an alternative for parametric and non-parametric linear-circular regression. Alonso-Pena et al. [11] developed different non-parametric tests to examine the equality and parallelism of the non-parametric regression curves across various groups, including linear-circular non-parametric regression cases. Meilán-Vila et al. [12] introduced local linear estimators for non-parametric multiple regression when the response variable was circular. Meilán-Vila et al. [13] proposed circular trend surface estimators considering a spatial linear-circular non-parametric regression model. Recently, Di Marzio et al. [14] has addressed the problem of estimating the Kernel regression function in the presence of measurement errors when the predictor and/or response variable is circular.

The concept of outliers in statistics is used for observations with significant distances from other observations. Outliers are among the most important problems encountered in modelling and forecasting because of undesirable effects on estimation. Since circular data differs from linear data in its geometric structure, detecting outliers requires special investigation.

The studies on detecting outliers for circular regression are generally based on simple circular regression. Abuzaid and Hussin [15] developed numerical and graphical methods using circular residuals for detecting a single outlier in circular regression. Abuzaid et al. [16] proposed a Mean Circular Error (MCE) statistic based on row deletion from the data to detect outliers. Rana et al. [17] developed an outlier detection method for the case in which both explanatory and response variables contain outliers. Mahmood et al. [18] suggested a robust approach (*RCD*_*U*_) based on circular median and generated cut-off points for different parameters of Von Mises (VM) distribution for univariate circular data. In another study, Mahmood et al. [19] proposed a robust method for detecting outliers in the simple circular regression model (*RCD*_*y*_) when both explanatory and response variables were circular. Alkasadi et al. [20] developed an outlier detection procedure for multiple circular regression models. They showed that the proposed statistic uses the DFFITc statistic and performs well in detecting outliers in multiple circular regression models.

All the abovementioned methods have been proposed for the cases when the residuals come from a well-defined VM distribution. In addition, the Wrapped-Cauchy (WC) error was assumed by Kato et al. [21], and its impact on the performance of simple circular regression was discussed by Abuzaid and Allahham [22].

The current study proposes a new method to detect outliers with circular residuals coming from the WC distribution in linear-circular non-parametric regression. Accordingly, this paper is organized as follows. Section 2 introduces the concept of a circular outlier, circular distance, and the proposed method. A comprehensive simulation study in Section 3 investigates the performance of the proposed method. In Section 4, a real data example is presented, and the implementation of NW and LL estimators are compared in the presence of outlier(s). Finally, the results are interpreted in Section 5.

## 2. Materials and method

### 2.1 Circular outlier and circular distance

The distance and outlier concepts for circular data differ from linear data due to their geometric structure. In circular (angular) data, a circular observation that is far from the main mass of the data (e.g., mean direction) can be referred to as an outlier ([23]).

Let *θ*_*i*_ and *θ*_*j*_, *i*, *j* = 1, 2, …, *n* be random circular observations taken over from the n-dimensional unit circle. Then the circular distance between *θ*_*i*_ and *θ*_*j*_ angular observations is described in Eq (1), which demonstrates the maximum distance between two circular (angular) observations. Note that the distance cannot be greater than *π* ([2]).


$$cd=\pi -\left|\pi -\left|\theta_{i}-\theta_{j}\right|\right|$$ (1)

The mean direction is used as a measure of location for circular data and is estimated using Eq (2)

$$\bar{\theta }=\left\{\begin{array}{ccc} \arctan\left(\frac{S}{C}\right) & , & C>0,S>0\\ \arctan\left(\frac{S}{C}\right)+\pi & , & C<0\\ \arctan\left(\frac{S}{C}\right)+2\pi & , & C>0,S<0 \end{array}\right.$$ (2)

where $S=\sum_{i=1}^{n}\sin\left(\theta_{i}\right)$ and $C=\sum_{i=1}^{n}\cos\left(\theta_{i}\right)$ ([1]).

The mean direction does not exhibit robust behaviour if the arithmetic mean is used to calculate the mean direction. Otenio and Anderson-Cook [24] stated that the circular median displayed more robust behaviour than the mean direction. He and Simpson [25] suggested using the circular median instead of the circular mean, especially when the dataset does not follow VM distribution. The circular median is the angle *θ* that minimizes Eq (3) ([1]).


$$d\left(\theta \right)=\pi -\frac{1}{n}\sum_{i=1}^{n}\left|\pi -\left|\theta_{i}-\theta \;\right|\right|$$ (3)

### 2.2 Method

This study proposes an outlier detection method based on the distances of linear circular regression residuals from the median value for the WC distributed data. Circular distributions such as VM or WC are symmetric; therefore, circular mean accurately represents the centre of the data. However, as He and Simpson [25] stated, the circular median is more robust than the circular mean when the data distribution is not symmetric. In the case of outliers included in a WC distributed data set, the distances from the circular mean may not work well for outlier detection since the distribution will deviate from symmetry to a certain degree due to outliers. Therefore, we have based our method on median distances.

Our method follows a two-step procedure. In the first stage, the cut-off points are calculated for the combinations of sample size and concentration parameters to determine whether the data is an outlier or not; in the second stage, the observations exceeding the corresponding cut-off value are defined as outliers. The procedure can be summarised in steps as follows.

Step 1. Calculate the absolute value of circular residuals from the fitted regression model.

$$e_{i}=\pi -\left|\pi -\left|y_{i}-{\hat{y}}_{i}\right|\right|$$

Step 2. Compute the absolute value of the circular residuals’ (*e*_*i*_) distances from their circular median.

$$dist_{i}=\pi -\left|\pi -\left|e_{i}-cmed\right|\right|$$

Step 3. Calculate the 90%, 95%, and 99% quantiles for the distances *dist*_*i*_.
Step 4. Repeat Step 3 2000 times and set the mean quantiles as cut-off values.
Step 5. Attribute the observations *dist*_*i*_ > *cut*—*off* as the outlier.

Cut-off points were produced for the NW and LL methods and are given in Tables 1–6.

**Table 1:** Cut-off points for NW under different concentration parameters and sample sizes, *q* = 0.90.

*ρ* n	0.10	0.20	0.30	0.40	0.50	0.60	0.70	0.80	0.85	0.90	0.95	0.99
**20**	1.4027	1.4011	1.3848	1.3572	1.2752	1.1792	1.0088	0.7610	0.6020	0.4125	0.2119	0.0573
**30**	1.4362	1.4463	1.4488	1.4209	1.3619	1.2644	1.0955	0.8053	0.6310	0.4385	0.2255	0.0549
**40**	1.4525	1.4501	1.4678	1.4573	1.4170	1.3148	1.1303	0.8412	0.6674	0.4558	0.2302	0.0552
**50**	1.4478	1.4764	1.4863	1.4932	1.4525	1.3413	1.1549	0.8756	0.6785	0.4645	0.2297	0.0553
**100**	1.4396	1.4771	1.5358	1.5526	1.5193	1.4170	1.2245	0.9227	0.7164	0.4858	0.2420	0.0542
**200**	1.4276	1.4843	1.5566	1.5848	1.5564	1.4688	1.2757	0.9574	0.7455	0.5059	0.2512	0.0531

**Table 2:** Cut-off points for LL under different concentration parameters and sample sizes, *q* = 0.90.

*ρ* n	0.10	0.20	0.30	0.40	0.50	0.60	0.70	0.80	0.85	0.90	0.95	0.99
**20**	1.3933	1.3889	1.3625	1.3305	1.2479	1.1531	0.9948	0.7659	0.6155	0.4297	0.2233	0.0535
**30**	1.4465	1.4589	1.4410	1.4087	1.3509	1.2654	1.1079	0.8256	0.6551	0.4588	0.2374	0.0523
**40**	1.4605	1.4592	1.4644	1.4497	1.4191	1.3212	1.1458	0.8665	0.6930	0.4768	0.2413	0.0509
**50**	1.4553	1.4793	1.4926	1.4980	1.4552	1.3560	1.1732	0.9013	0.7014	0.4808	0.2393	0.0511
**100**	1.4527	1.4818	1.5321	1.5556	1.5277	1.4285	1.2450	0.9413	0.7362	0.5014	0.2503	0.0510
**200**	1.4333	1.4874	1.5614	1.5892	1.5621	1.4787	1.2884	0.9714	0.7560	0.5151	0.2569	0.0511

**Table 3:** Cut-off points for NW under different concentration parameters and sample sizes, *q* = 0.95.

*ρ* n	0.10	0.20	0.30	0.40	0.50	0.60	0.70	0.80	0.85	0.90	0.95	0.99
**20**	1.5971	1.6262	1.6319	1.6474	1.6068	1.5499	1.3897	1.1419	0.9628	0.7016	0.3897	0.1035
**30**	1.6309	1.6716	1.7149	1.7349	1.7273	1.6775	1.5578	1.2695	1.0778	0.8078	0.4613	0.1041
**40**	1.6462	1.6851	1.7437	1.7882	1.7958	1.7613	1.6078	1.3273	1.1198	0.8435	0.4638	0.0983
**50**	1.6385	1.7040	1.7661	1.8227	1.8498	1.8032	1.6682	1.4163	1.1781	0.8808	0.4836	0.1045
**100**	1.6218	1.7114	1.8227	1.8922	1.9311	1.9100	1.7888	1.5259	1.2777	0.9530	0.5125	0.1036
**200**	1.6046	1.7199	1.8425	1.9328	1.9769	1.9750	1.8670	1.5932	1.3501	1.0067	0.5384	0.1085

**Table 4:** Cut-off points for LL under different concentration parameters and sample sizes, *q* = 0.95.

*ρ* n	0.10	0.20	0.30	0.40	0.50	0.60	0.70	0.80	0.85	0.90	0.95	0.99
**20**	1.6169	1.6416	1.6340	1.6307	1.5942	1.5291	1.3835	1.1514	0.9841	0.7317	0.4148	0.1060
**30**	1.6616	1.7071	1.7246	1.7339	1.7309	1.6842	1.5694	1.2986	1.1134	0.8430	0.4873	0.1076
**40**	1.6785	1.7052	1.7582	1.7881	1.8017	1.7712	1.6331	1.3576	1.1590	0.8823	0.4893	0.1007
**50**	1.6679	1.7170	1.7794	1.8312	1.8515	1.8124	1.6887	1.4499	1.2127	0.9108	0.5075	0.1069
**100**	1.6475	1.7208	1.8270	1.8948	1.9345	1.9194	1.8071	1.5482	1.3014	0.9765	0.5299	0.1062
**200**	1.6189	1.7234	1.8459	1.9345	1.9826	1.9817	1.8785	1.6080	1.3633	1.0211	0.5502	0.1101

**Table 5:** Cut-off points for NW under different concentration parameters and sample sizes, *q* = 0.99.

*ρ* n	0.10	0.20	0.30	0.40	0.50	0.60	0.70	0.80	0.85	0.90	0.95	0.99
**20**	1.7756	1.8186	1.8577	1.9203	1.9328	1.9239	1.8369	1.6388	1.4623	1.2014	0.8096	0.2533
**30**	1.8061	1.8668	1.9486	2.0223	2.0707	2.0823	2.0639	1.8753	1.7255	1.4555	1.0318	0.3481
**40**	1.8152	1.8867	1.9828	2.0845	2.1584	2.2017	2.1783	2.0115	1.8824	1.6028	1.1729	0.3951
**50**	1.8038	1.8974	2.0056	2.1144	2.2043	2.2529	2.2348	2.1483	1.9970	1.7126	1.2413	0.4515
**100**	1.7805	1.9084	2.0623	2.1858	2.2988	2.3863	2.4169	2.3542	2.2367	1.9853	1.4694	0.4588
**200**	1.7605	1.9128	2.0812	2.2258	2.3532	2.4600	2.5160	2.4882	2.3962	2.2077	1.6672	0.5408

**Table 6:** Cut-off points for LL under different concentration parameters and sample sizes, *q* = 0.99.

*ρ* n	0.10	0.20	0.30	0.40	0.50	0.60	0.70	0.80	0.85	0.90	0.95	0.99
**20**	1.8224	1.8624	1.8801	1.9184	1.9309	1.9141	1.8415	1.6598	1.4912	1.2423	0.8533	0.2762
**30**	1.8551	1.9205	1.9734	2.0314	2.0838	2.0942	2.0729	1.8999	1.7649	1.4988	1.0765	0.3723
**40**	1.8668	1.9155	2.0024	2.0932	2.1702	2.2118	2.1921	2.0384	1.9183	1.6433	1.2132	0.4258
**50**	1.8478	1.9253	2.0246	2.1303	2.2091	2.2653	2.2470	2.1725	2.0264	1.7452	1.2799	0.4772
**100**	1.8168	1.9229	2.0666	2.1898	2.3022	2.3925	2.4238	2.3680	2.2555	2.0116	1.4975	0.4796
**200**	1.7807	1.9194	2.0827	2.2274	2.3548	2.4596	2.5203	2.4951	2.4046	2.2201	1.6882	0.5556

The performance of the proposed method is determined by three different measures as given in below ([18]).

- Masking (M): Rate of detected outliers as inliers.
$$M=\frac{\text{Number}\;\text{of}\;\text{observations}\;\text{that}\;\text{cannot}\;\text{be}\;\text{detected}\;\text{even}\;\text{if}\;\text{they}\;\text{are}\;\text{outliers}}{\text{Number}\;\text{of}\;\text{outliers}}$$ (4)
- Swamping (S): Rate of inlier observations detected as outliers.
$$S=\frac{\text{Number}\;\text{of}\;\text{observations}\;\text{detected}\;\text{as}\;\text{outliers}\;\text{in}\;\text{the}\;\text{absence}\;\text{of}\;\text{outliers}}{n-\text{Number}\;\text{of}\;\text{outliers}}$$ (5)
- True Detection Rate (TDR): Rate of true detected outliers,
  where *n* denotes the sample size.

## 3. Simulation study

A simulation study was performed to evaluate the outlier detection performance of the proposed method, with five factors: Sample size, contamination degree, concentration values, regression estimation procedure, and the number of outliers. Simulations were conducted via a crossover design with the factor levels:

**Table:** 

Factors	Levels
Sample size	20, 40, 50, 100 and 200
Contamination degree	0.10, 0.20, 0.30, 0.40,0.45, 0.50, 0.55, 0.60, 0.65, 0.70, 0.75, 0.80, 0.85, 0.90
Concentration parameter	0.10, 0.20, 0.30, 0.40,0.45, 0.50, 0.55, 0.60, 0.65, 0.70, 0.75, 0.80, 0.85, 0.90, 0.95, 0.99
Regression estimation method	NW and LL
Percentage of Contamination	0.01, 0.05, 0.10

Note that since some percentage values of *n* = 20, 40 and 50 are less than 1, the outlier number was rounded to 1 in these cases.

The data for the explanatory variable *X* of the linear-circular regression model come from *X* ~ *N* (3, 0.25). Then the circular response *y*_*i*_ is generated through Eq (6).


$${\text{y}}_{i}\text{=sin}\left(1.5\times \left(x_{i}-\frac{\pi }{2}\right)\right)+\left(2\times \sqrt{\frac{2}{3}}\right)\times \cos\left(\frac{x_{i}}{3}\right)+\varepsilon_{i}\;\left(\mathrm{mod}\;2\pi \right),\;\varepsilon_{i}∼WC\left(0,\rho \right).$$ (6)

NW and LL methods with Gaussian kernel are used to obtain non-parametric regression fits as defined in Di Marzio et al. [5]. In addition, the leave-one-out Cross Validation (CV) is used to obtain bandwidths to estimate regressions and is given in Eq (7).


$$cv=\left(\sum_{i=1}^{n}\left(-\cos\left(\Theta_{i}-{\hat{f}}^{-1}\left(x_{i}\right)\right)\right)\right),\;i=1,2,\ldots ,n$$ (7)

The optimal bandwidth is the value minimizing Eq (7). Here, ${\hat{f}}^{-1}$ denotes the estimated value with the exclusion of the pair of observations (*X*_*i*_, Θ_i_) from the whole data set. The procedure is iterated by excluding only one pair of observations from the data set. The contaminated *y*_*k*_ is generated as suggested by Abuzaid et al. [16] and Mahmood et al. [19]:

$$y_{k}=y+\gamma \pi (\text{mod}\;2\pi \text{)}$$ (8)

where *γ* refers to the contamination degree.

All the outputs of the performed simulation through the designed experiment were obtained, yet only some were included within the text for space and simplicity. Since the results of performance indicators for all sample sizes are consistent, only the results for the concentration parameters *ρ* = 0.70, *ρ* = 0.90 and *q* = 0.95 when *n* = 40,100 and 200 are included inside the paper and given in Figs 1–14.

**Fig 1:**
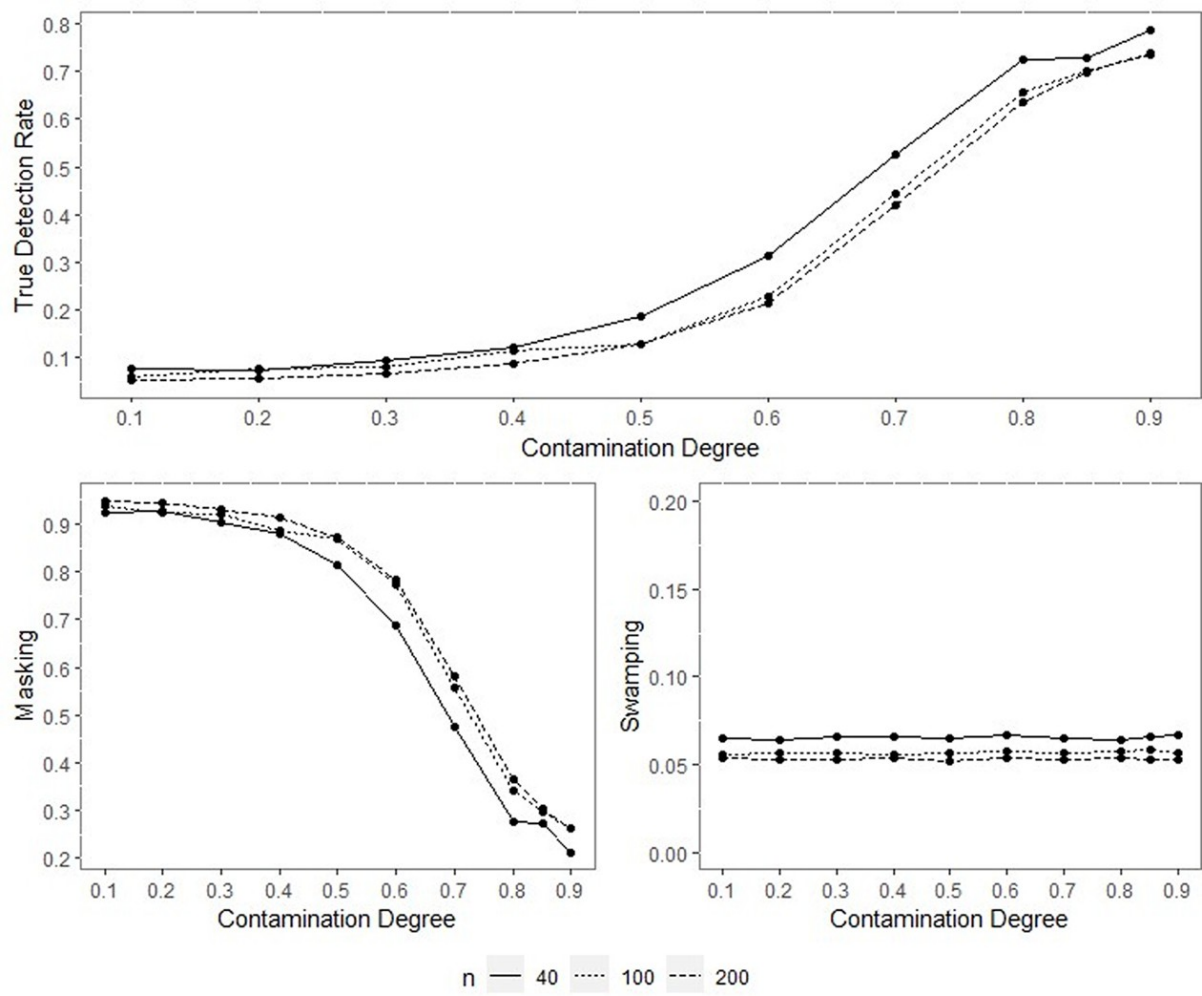
TDR, M, and S of NW for different contamination degrees with percentage of contamination 1%, *ρ* = 0.70, *q* = 0.95.

**Fig 2:**
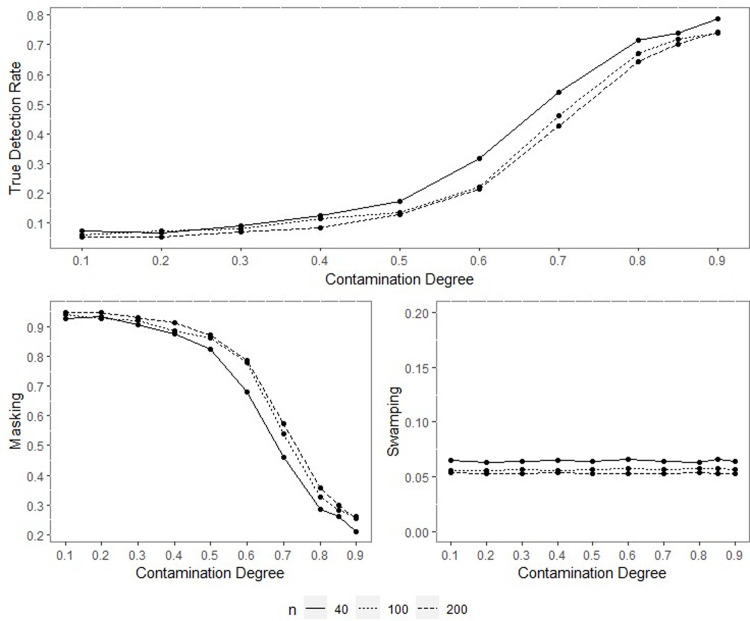
TDR, M, and S of LL for different contamination degrees with percentage of contamination 1%, *ρ* = 0.70, *q* = 0.95.

**Fig 3:**
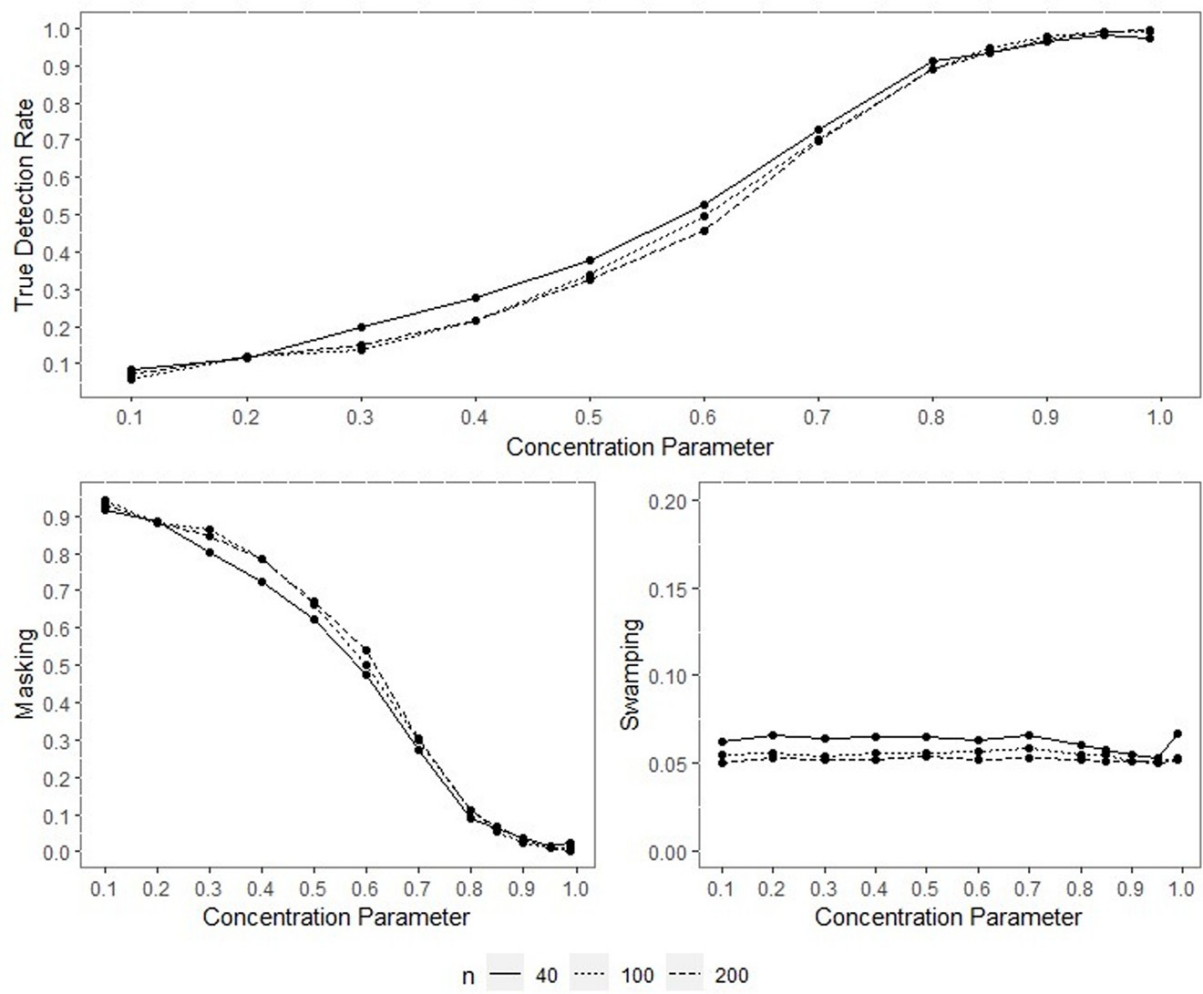
TDR, M, and S of NW for different concentration parameters with percentage of contamination 1%, *γ* = 0.85, *q* = 0.95.

**Fig 4:**
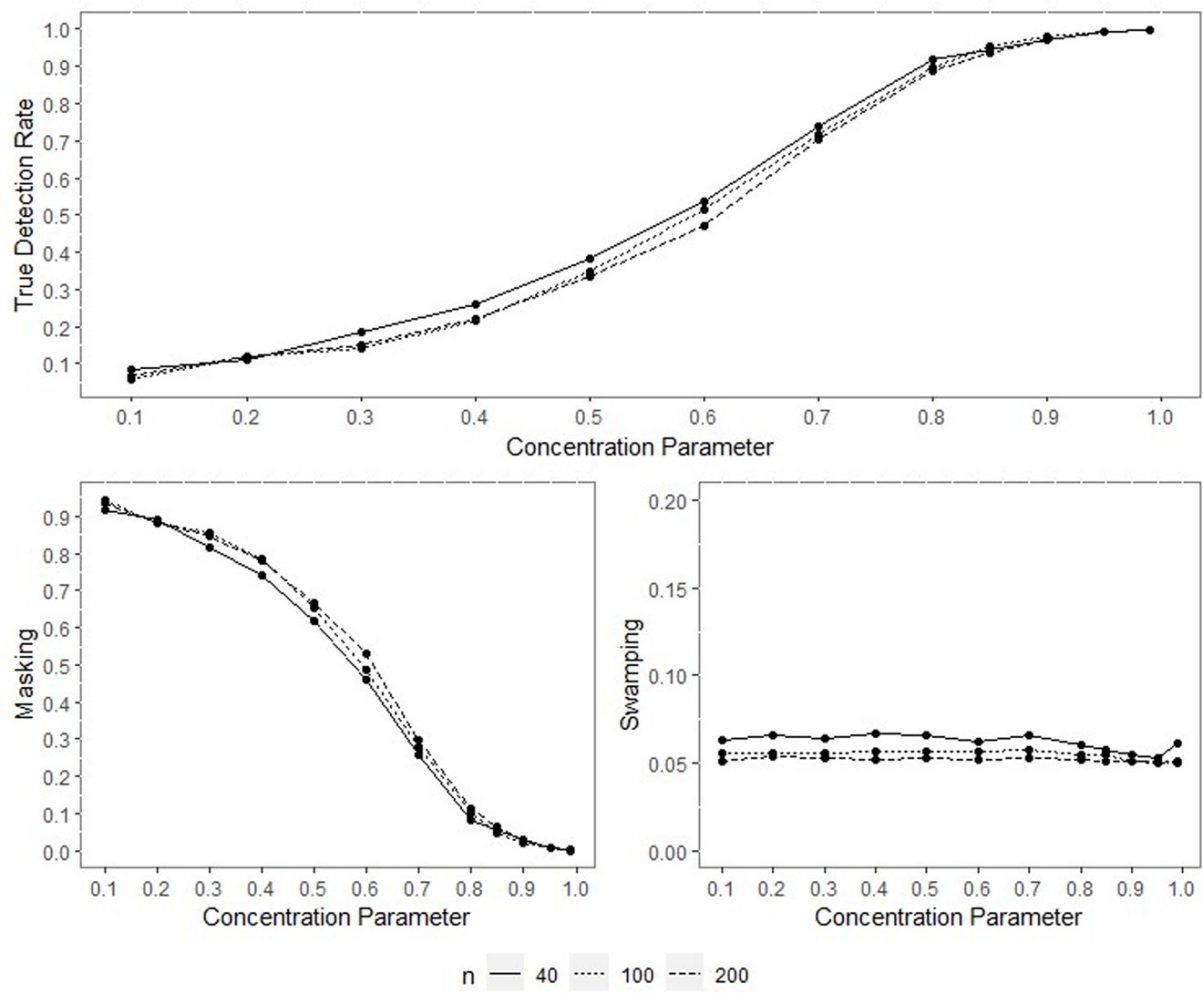
TDR, M, and S of LL for different concentration parameters with percentage of contamination 1%, *γ* = 0.85, *q* = 0.95.

**Fig 5:**
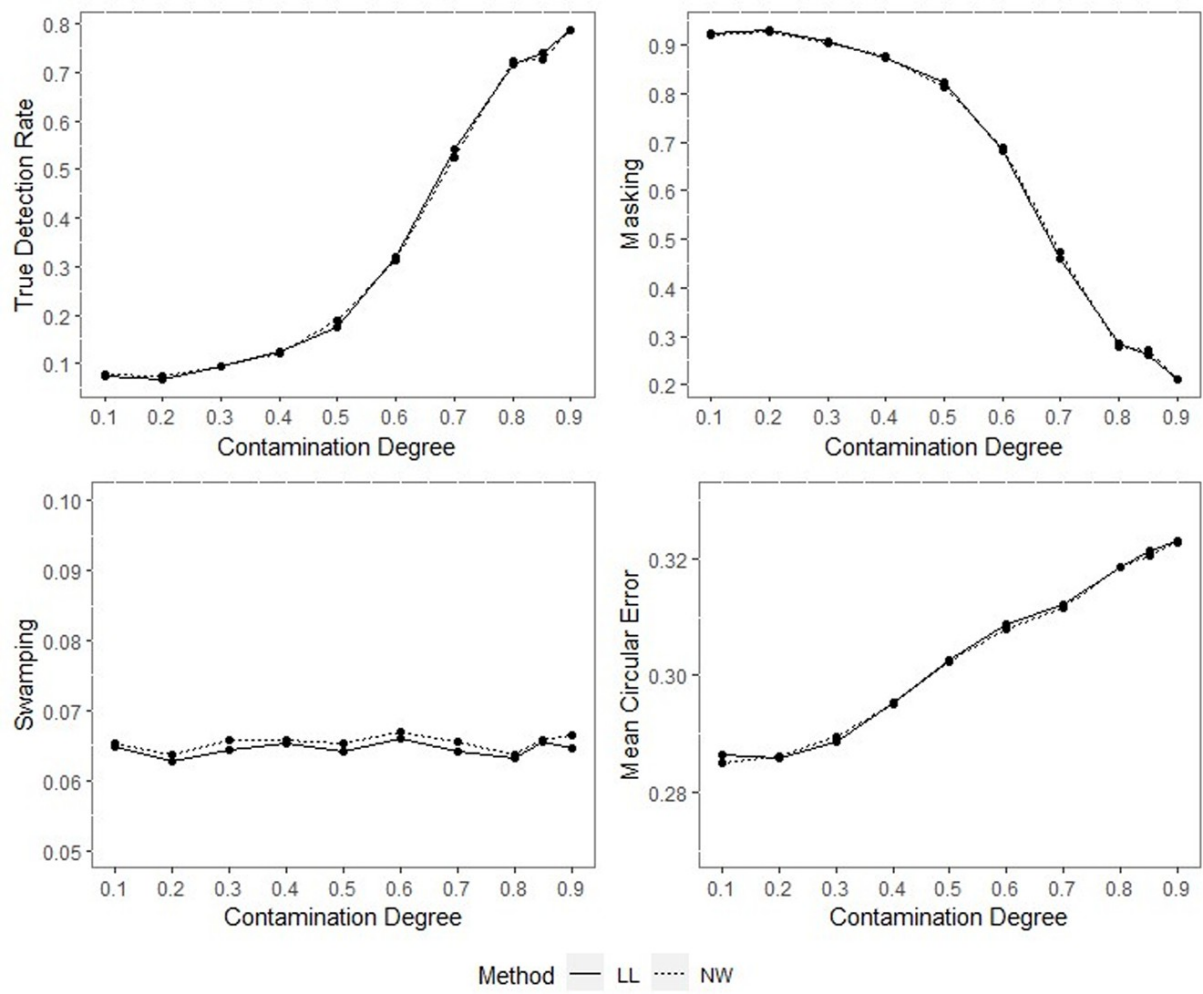
TDR, M, S and MCE values of NW and LL for different contamination degrees with percentage of contamination 1%, *ρ* = 0.70, *q* = 0.95, *n* = 40.

**Fig 6:**
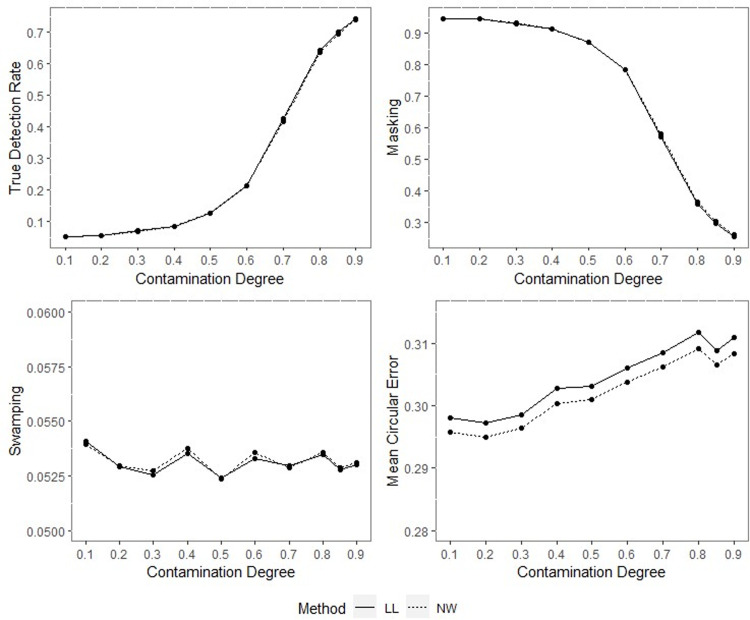
TDR, M, S and MCE values of NW and LL for different contamination degrees with percentage of contamination 1%, *ρ* = 0.70, *q* = 0.95, *n* = 200.

**Fig 7:**
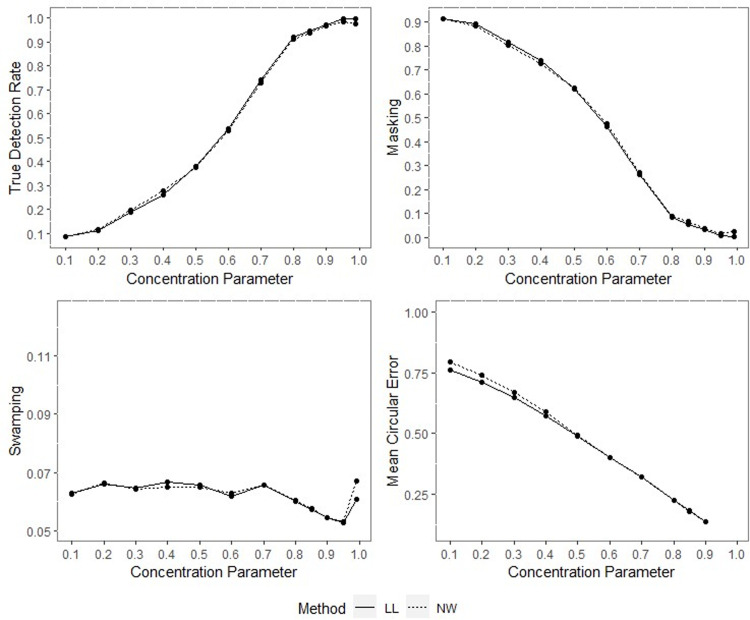
TDR, M, S and MCE values of NW and LL, for different concentration parameters with percentage of contamination 1%, *γ* = 0.85, *q* = 0.95, *n* = 40.

**Fig 8:**
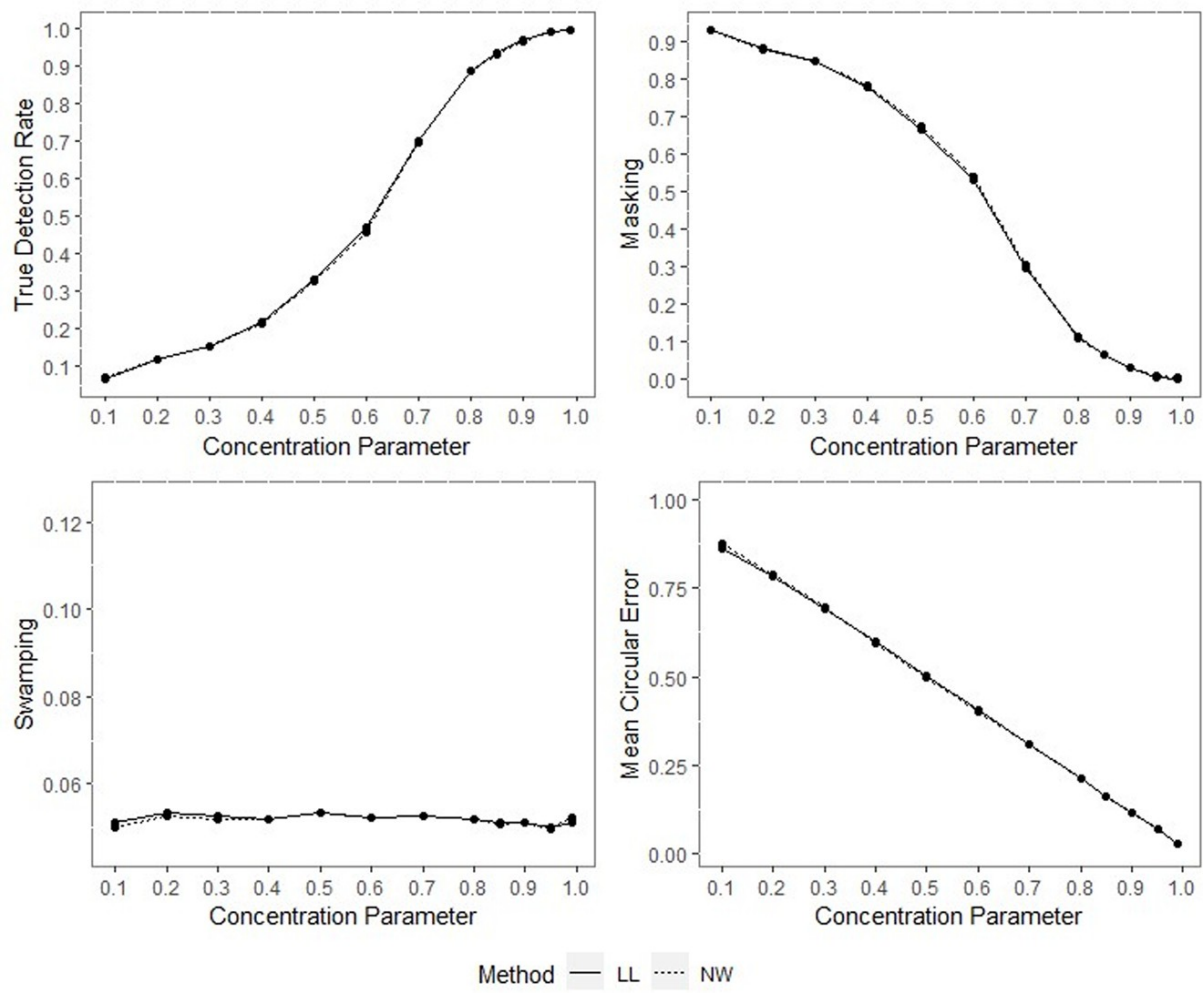
TDR, M, S and MCE values of NW and LL for different concentration parameters with percentage of contamination 1%, *γ* = 0.85, *q* = 0.95, *n* = 200.

**Fig 9:**
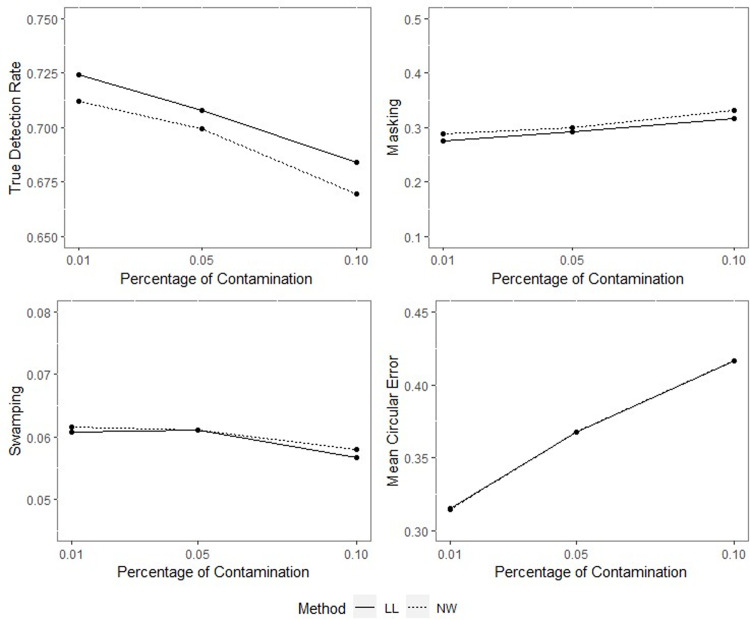
TDR, M, S and MCE values of NW and LL for different percentages of contamination with *ρ* = 0.70, *γ* = 0.85, *q* = 0.95, *n* = 50.

**Fig 10:**
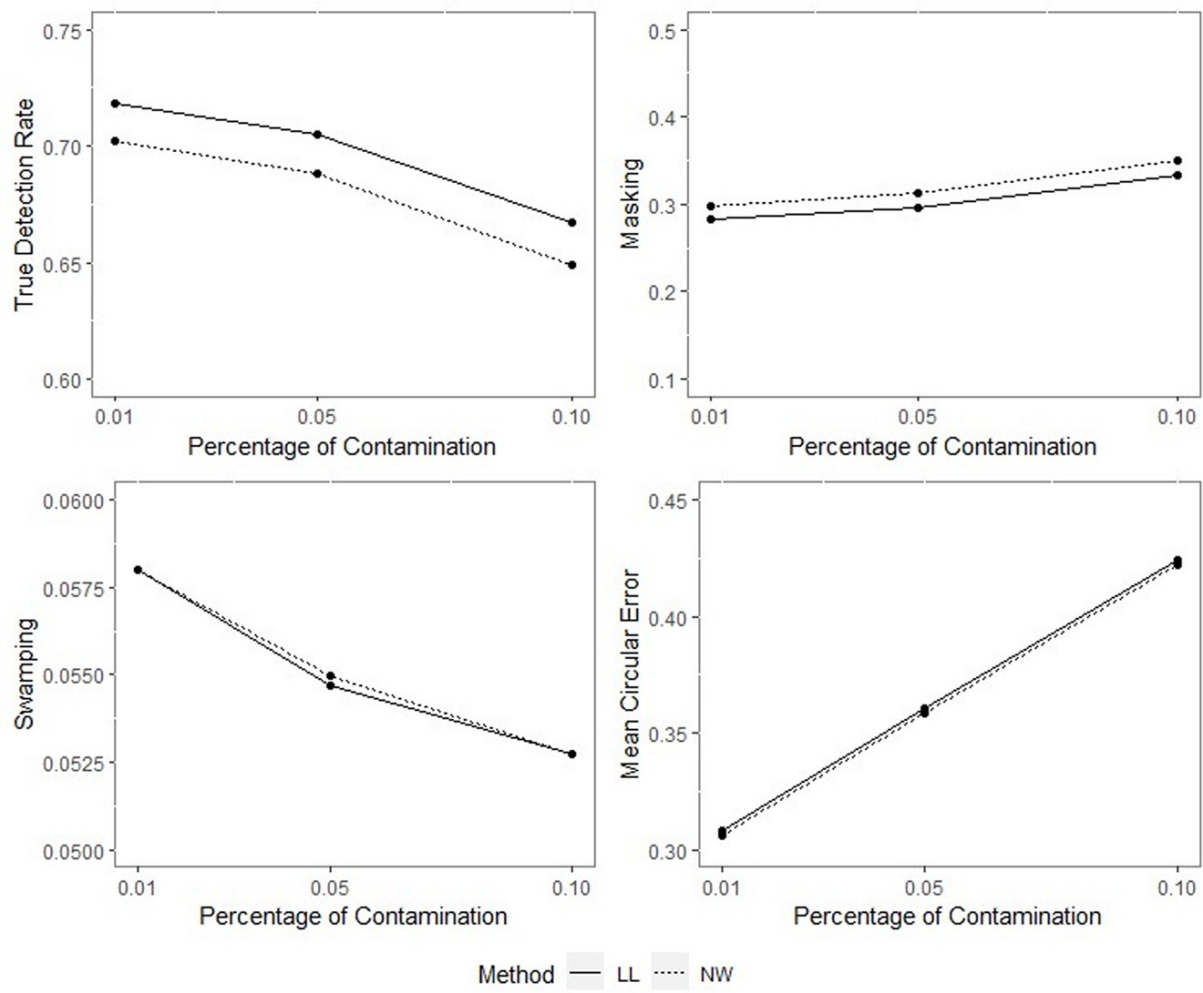
TDR, M, S and MCE values of NW and LL for different percentages of contamination with *ρ* = 0.70, *γ* = 0.85, *q* = 0.95, *n* = 100.

**Fig 11:**
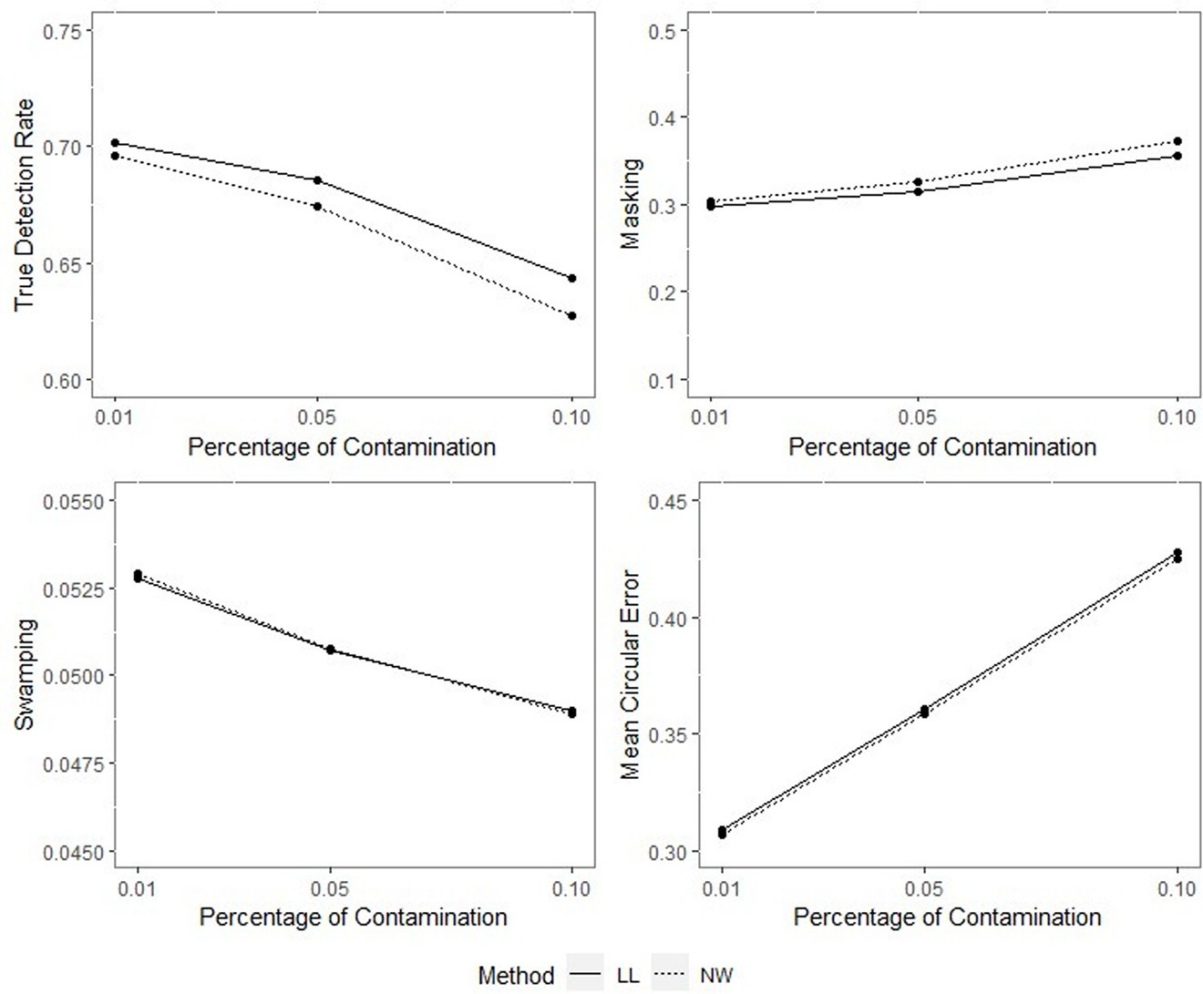
TDR, M, S and MCE values of NW and LL for different percentages of contamination with *ρ* = 0.70, *γ* = 0.85, *q* = 0.95, *n* = 200.

**Fig 12:**
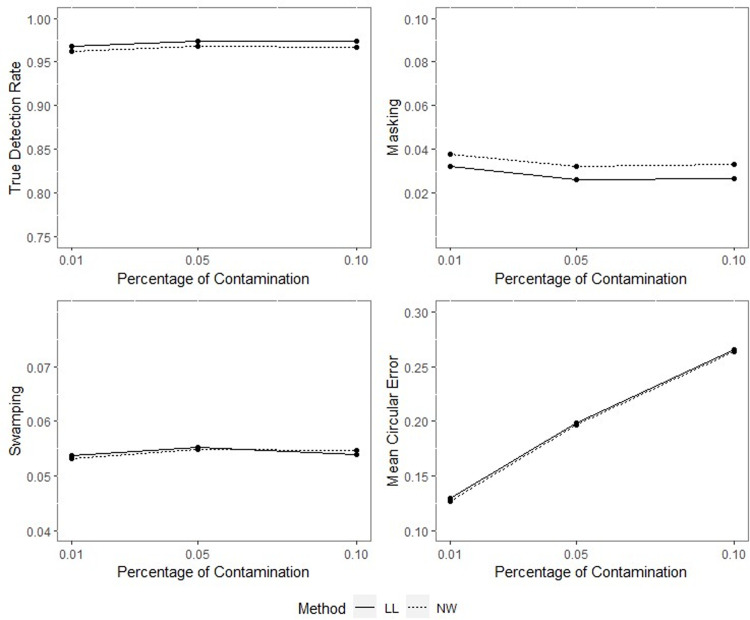
TDR, M, S and MCE values of NW and LL for different percentages of contamination with *ρ* = 0.90, *γ* = 0.85, *q* = 0.95, *n* = 50.

**Fig 13:**
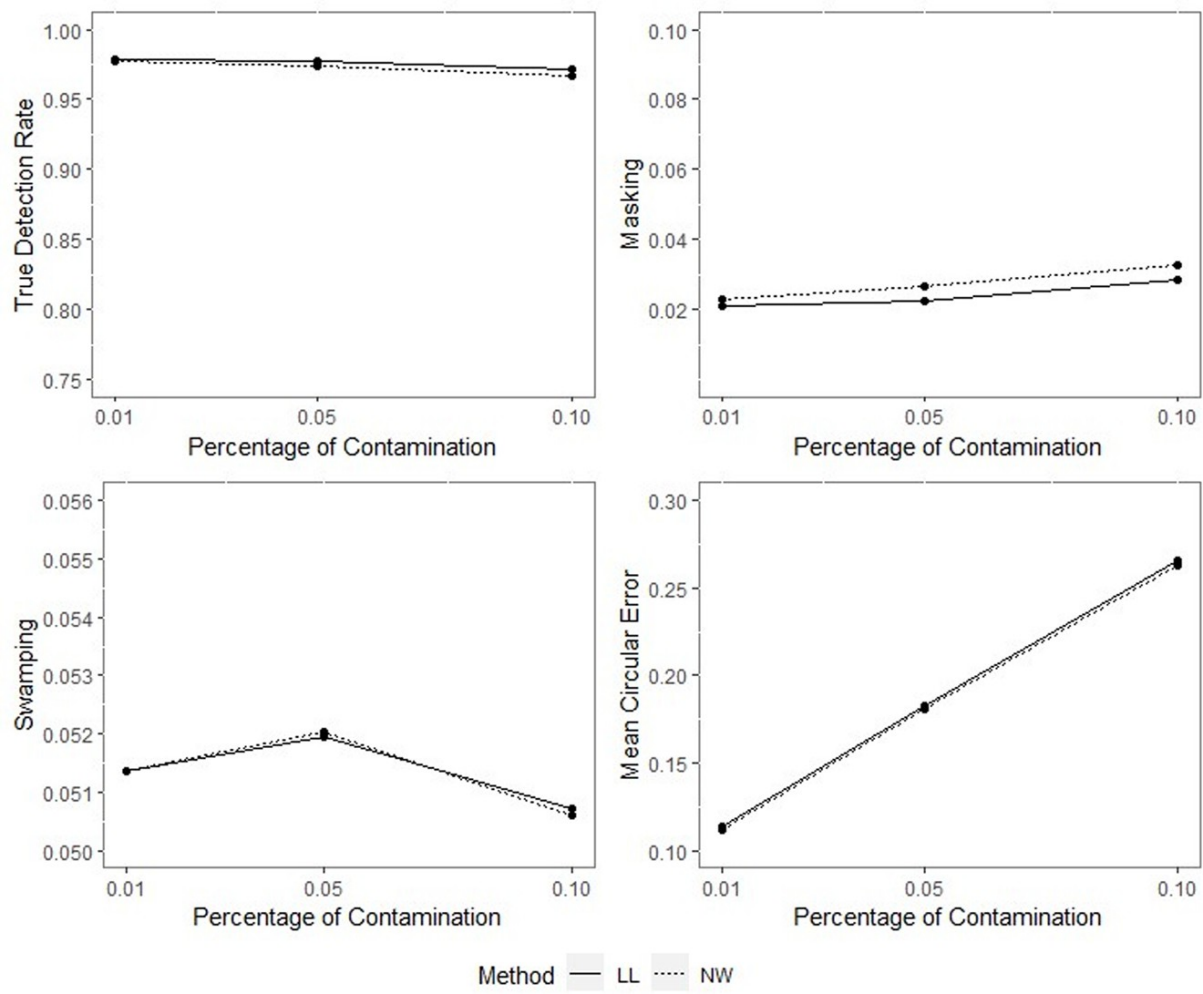
TDR, M, S and MCE values of NW and LL for different percentages of contamination with *ρ* = 0.90, *γ* = 0.85, *q* = 0.95, *n* = 100.

**Fig 14:**
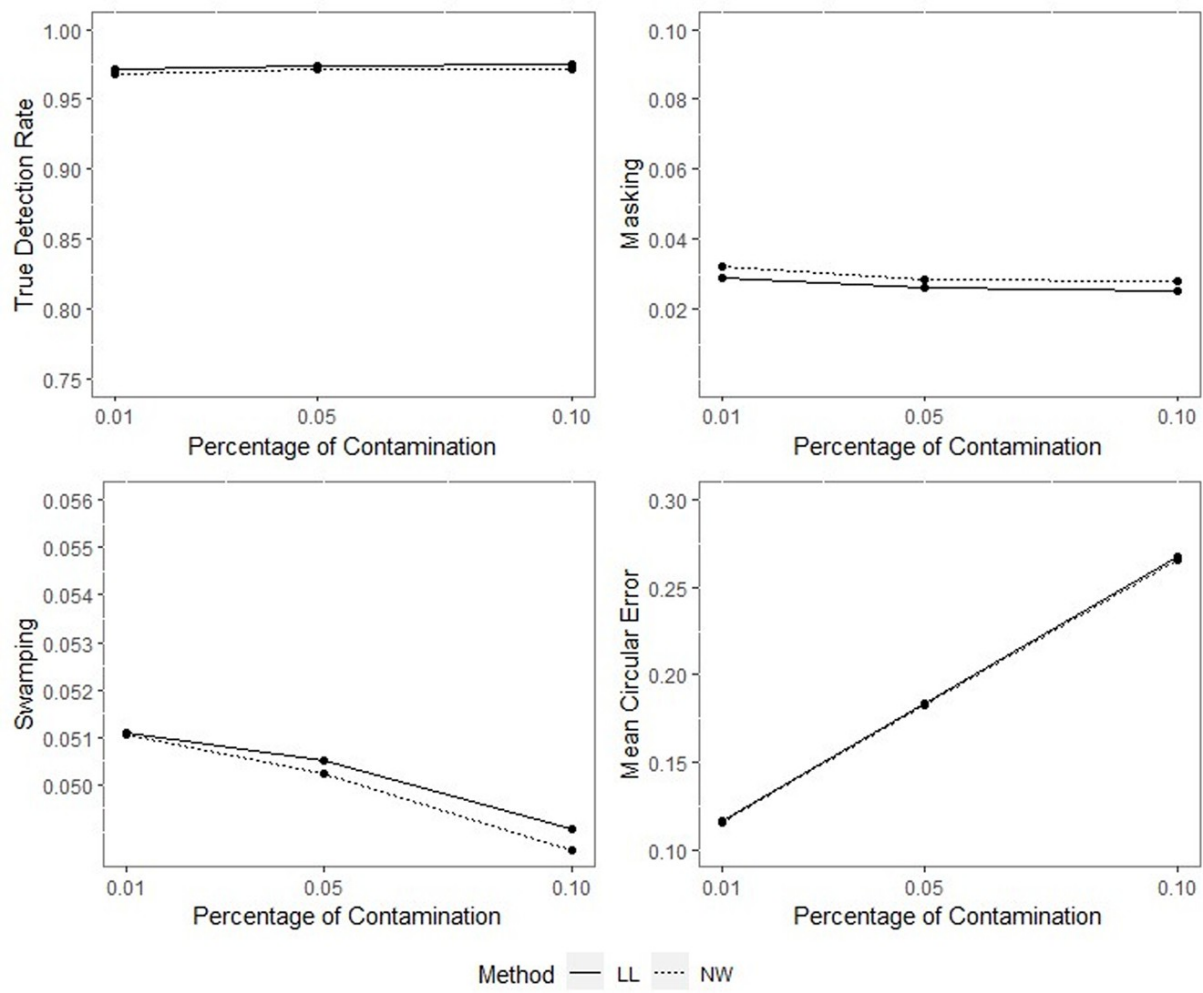
TDR, M, S and MCE values of NW and LL for different percentages of contamination with *ρ* = 0.90, *γ* = 0.85, *q* = 0.95, *n* = 200.

The outputs of the simulation showed that all the performance criteria, TDR, masking, and swamping rates, are sensitive for specific ranges at all levels of simulation design factors. On the other hand, the increasing value of the concentration parameter has a positive effect on TDR. In most cases, just as the value of the concentration parameter increases, so does TDR, and this trend can be observed more regularly at greater contamination degrees. Furthermore, the TDR improves much faster when the concentration parameter is greater than 0.70. It would be wrong to assume a significant increase occurs in TDR as the contamination degree becomes larger; however, it could be stated that the TDR is higher at large contamination degrees than at small contamination degrees. The opposite interpretation can be made for masking.

TDR performance varies depending on the concentration parameter at different sample sizes. For the 0.20–0.80 range of the concentration parameter, the TDR performs better as the sample size gets smaller, while outside this range, it is not affected by the sample size. At 0.85 and higher values of the concentration parameter, the TDR approaches 1 in all sample sizes.

The swamping rate is affected mainly by sample size since the swamping rate decreases as the sample size increases. While the percentage of contamination is effective at the smaller values of the concentration parameter on performance criteria, both percentage of contamination and sample size lose their effect as the concentration parameter becomes larger. NW and LL show performances close to one another for almost all performance evaluation criteria. However, the increase in percentage of contamination causes the LL method to yield slightly better performance values than NW.

Some of the simulation outputs are presented here as the rest has similar characteristics and are given in the S1–S5 Files.

## 4. Real data example

**Fig 15:**
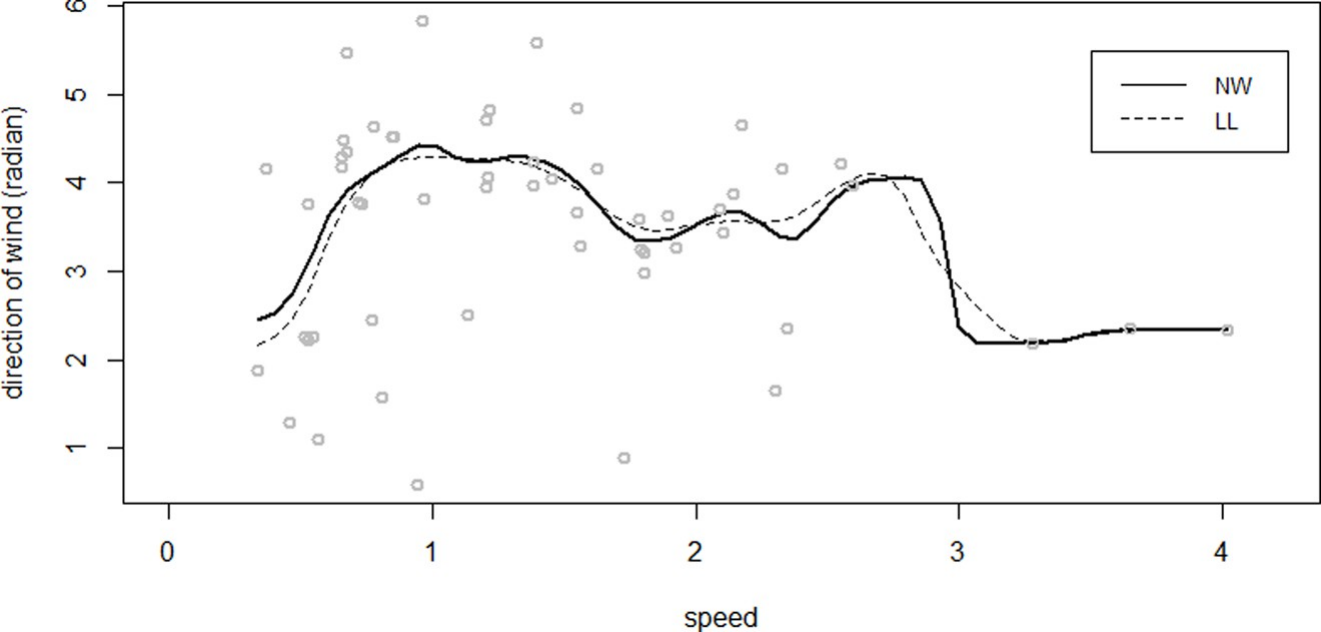
NW and LL fits for the Wind Turbine Scada dataset.

**Fig 16:**
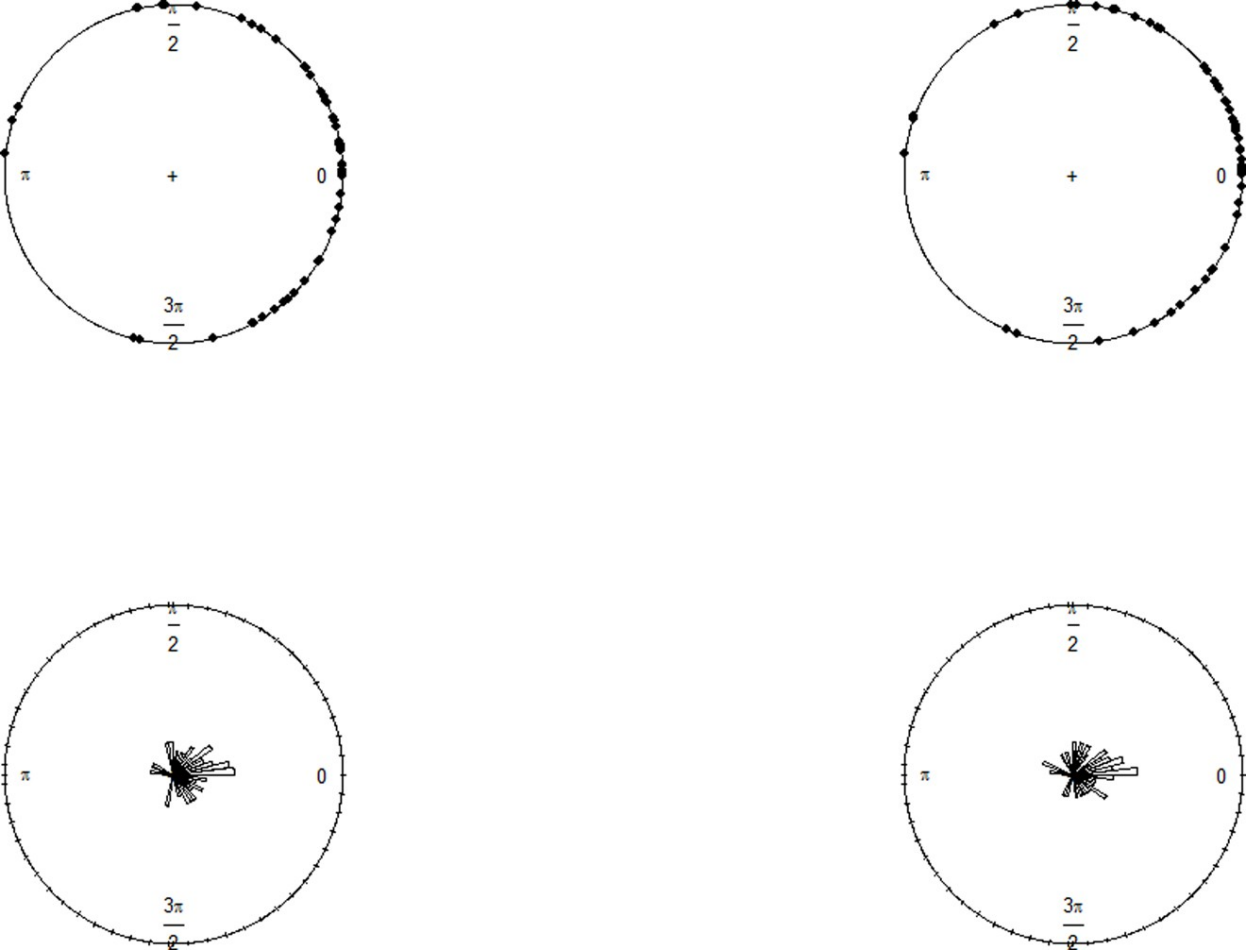
The circular plot and the rose diagram of the estimated circular residuals when NW is applied to the Wind Turbine Scada dataset (left), the circular plot and the rose diagram of the estimated circular residuals when NW is applied to the Wind Turbine Scada dataset (right).

The proposed method was implemented in the 2018 GEFC Wind Turbine Scada Dataset, which includes wind speed (m/s) and wind direction (°) measurements taken from the Scada system of a wind turbine operating and generating electricity in Turkey ([26]). To model the relationship between wind direction and wind speed, the 10-minute measurements of the related data set between 05.01.2018, 20:50–06.01.2018, 05:50 was considered, and since “wind speed” (explanatory variable) is linear, and the “wind direction” (response variable) is circular, a linear-circular kernel regression is fitted with both NW and LL methods (Fig 15). The circular plots and rose diagrams of the estimated circular residuals for both methods are given in Fig 16.

Before fitting regression models, the distribution of the data was investigated. The Watson *U*^2^ test was employed to test the null hypothesis that the distribution is WC. Because the *U*^2^ test does not exist in any software for WC distribution, the authors produced asymptotic critical value with the bootstrap method following Sun [27]. The results confirmed that the WC distribution appeared to be a good fit for the Wind Turbine Scada dataset with the mean direction 1.074 and concentration parameter 0.1831.

**Fig 17:**
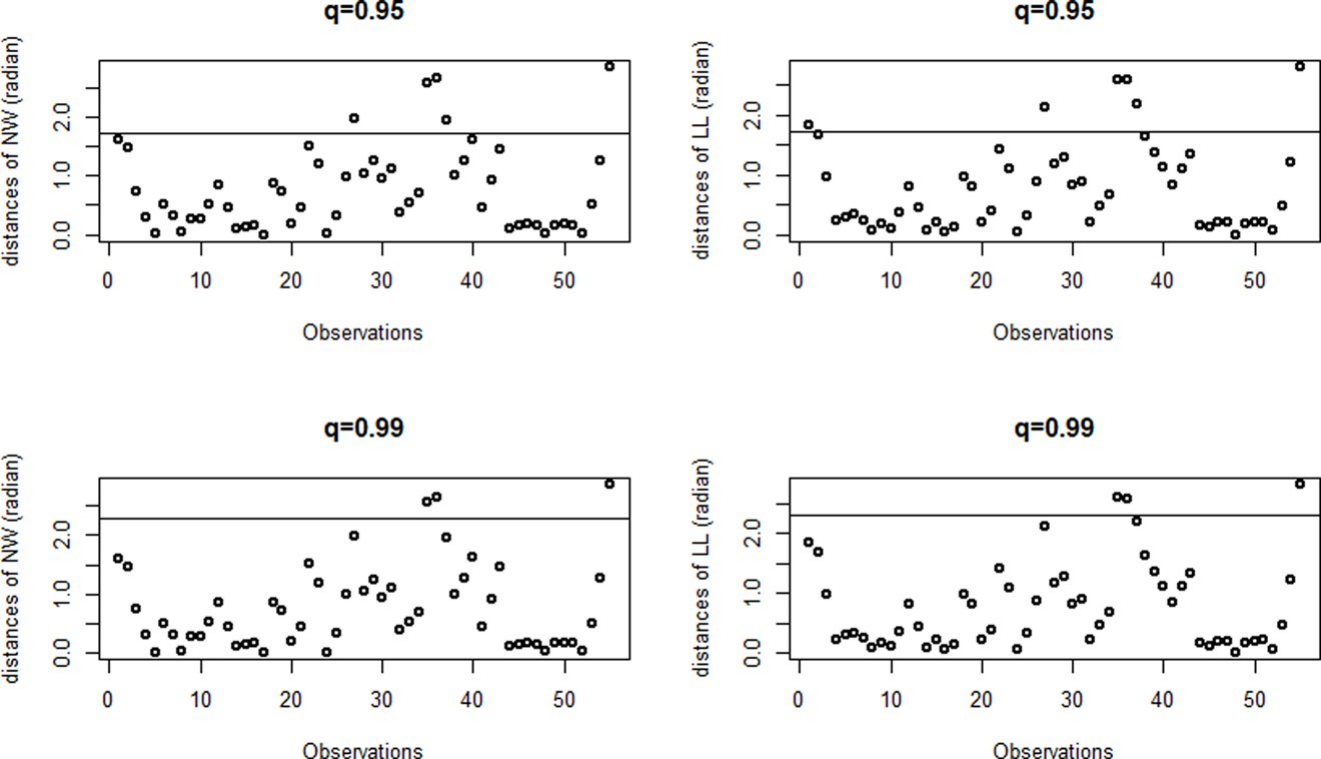
The distances and the cut-off points for the Wind Turbine Scada dataset, using NW fits (left) and using LL fits (right).

The circular distances between the absolute residuals of NW and LL and their circular medians were calculated and compared against the cut-off values 1.1989, 1.7149, and 2.2787 of NW and 1.1914, 1.7161, and 2.2930 of LL for the quantiles 0.90, 0.95 and 0.99, respectively. The investigation of outliers resulted in the 27^th^, 35^th^, 36^th^, 37^th^ and 55^th^ observations at *q* = 0.95, and 35^th^, 36^th,^ and 55^th^ observations at *q* = 0.99. The cut-off points and the distances are illustrated in Fig 17, where the straight indicate the cut-off points.

## 5. Conclusion

The present study deals with the problem of detecting outliers in non-parametric linear-circular regression. An outlier detection method based on linear-circular regression residuals distances from the circular median value has been proposed for the WC distributed errors. The corresponding cut-off points were identified via simulations. In addition, a comprehensive simulation study was carried out to evaluate the performance of the proposed procedure in terms of true detection, masking, and swamping rates. The results showed that the proposed method performs well for medium and higher contamination degrees. It was also observed that the method’s performance increases as the sample size and homogeneity of data increase. The findings were illustrated and supported through a real data set example. NW and LL methods with Gaussian kernel were used to obtain non-parametric regression fits. The results indicate that when the response variable of linear-circular regression contains outliers, the Local Linear Estimation method is preferable to the Nadaraya-Watson method.

It should be noted that although the proposed method is quite satisfactory for outlier detection in a linear-circular non-parametric regression model, the method and, therefore, the generated cut-off values are model specific. Thus, the use of calculated cut-off values is limited only to the linear-circular non-parametric regression and estimation methods used in this study. Further studies are planned to address these issues and develop outlier detection methods for circular-linear and circular-circular non-parametric regression models.

## Supporting information

S1 File Simulation results for n = 20. (PDF)

S2 File Simulation results for n = 40. (PDF)

S3 File Simulation results for n = 50. (PDF)

S4 File Simulation results for n = 100. (PDF)

S5 File Simulation results for n = 200. (PDF)
