# Supplementary material for: A method for detecting outliers in linear-circular non-parametric regression
Source: PLoS One. 2023 Jun 12;18(6):e0286448. doi: 10.1371/journal.pone.0286448 (PMC10259788; doi:10.1371/journal.pone.0286448)
Supplement: S1 File — (PDF) [file pone.0286448.s001.pdf]

A Method for Detecting Outliers in Linear-Circular Non-Parametric Regression

Sümeyra Sert[1]* *and* Filiz Kardiyen[2]

[1] *Selcuk University, Department of Statistics, 42250, Selcuklu, Konya, Turkey;*

[1]ORCID id: https://orcid.org/0000-0002-4647-1583

sumeyra.sert@selcuk.edu.tr

[2]*Gazi University, Department of Statistics, Teknikokullar, 06500, Ankara, Turkey.*

[2]ORCID id: https://orcid.org/0000-0002-8730-2751

fyuva@gazi.edu.tr

Supplementary File (n=20)

Table 1. Simulation results for n=20, %1 percentage of contamination

		**q=0.95**								**q=0.99**							
		NW				LL				NW				LL			
γ	ρ	TDR	M	S	MCE	TDR	M	S	MCE	TDR	M	S	MCE	TDR	M	S	MCE
**0.10**	0.1	0.0770	0.9230	0.0831	0.7183	0.0750	0.9250	0.0820	0.6638	0.0490	0.9510	0.0515	0.7183	0.0500	0.9500	0.0511	0.6638
	0.2	0.0770	0.9230	0.0772	0.6828	0.0710	0.9290	0.0794	0.6323	0.0430	0.9570	0.0464	0.6828	0.0390	0.9610	0.0462	0.6323
	0.3	0.0710	0.9290	0.0813	0.6151	0.0780	0.9220	0.0829	0.5747	0.0430	0.9570	0.0511	0.6151	0.0440	0.9560	0.0516	0.5747
	0.4	0.0680	0.9320	0.0775	0.5353	0.0760	0.9240	0.0777	0.5057	0.0390	0.9610	0.0454	0.5353	0.0400	0.9600	0.0451	0.5057
	0.5	0.0940	0.9060	0.0831	0.4671	0.0840	0.9160	0.0815	0.4479	0.0560	0.9440	0.0497	0.4671	0.0490	0.9510	0.0492	0.4479
	0.6	0.0860	0.9140	0.0786	0.3676	0.0810	0.9190	0.0781	0.3540	0.0500	0.9500	0.0471	0.3676	0.0480	0.9520	0.0458	0.3540
	0.7	0.0760	0.9240	0.0792	0.2783	0.0730	0.9270	0.0794	0.2719	0.0480	0.9520	0.0471	0.2783	0.0460	0.9540	0.0470	0.2719
	0.8	0.0770	0.9230	0.0695	0.1852	0.0750	0.9250	0.0695	0.1820	0.0410	0.9590	0.0409	0.1852	0.0390	0.9610	0.0416	0.1820
	0.85	0.0620	0.9380	0.0628	0.1375	0.0630	0.9370	0.0616	0.1371	0.0330	0.9670	0.0378	0.1375	0.0360	0.9640	0.0375	0.1371
	0.9	0.0630	0.9370	0.0617	0.0938	0.0630	0.9370	0.0618	0.0947	0.0320	0.9680	0.0345	0.0938	0.0310	0.9690	0.0345	0.0947
	0.95	0.0830	0.9170	0.0547	0.0468	0.0900	0.9100	0.0550	0.0482	0.0150	0.9850	0.0272	0.0468	0.0190	0.9810	0.0278	0.0482
	0.99	0.8220	0.1780	0.0542	0.0108	0.9030	0.0970	0.0527	0.0115	0.1810	0.8190	0.0169	0.0108	0.2890	0.7110	0.0171	0.0115
**0.20**	0.1	0.0750	0.9250	0.0834	0.7171	0.0870	0.9130	0.0864	0.6648	0.0550	0.9450	0.0517	0.7171	0.0630	0.9370	0.0539	0.6648
	0.2	0.0670	0.9330	0.0756	0.6927	0.0730	0.9270	0.0775	0.6448	0.0450	0.9550	0.0459	0.6927	0.0450	0.9550	0.0475	0.6448
	0.3	0.0790	0.9210	0.0824	0.6235	0.0790	0.9210	0.0815	0.5733	0.0470	0.9530	0.0511	0.6235	0.0450	0.9550	0.0502	0.5733
	0.4	0.0840	0.9160	0.0806	0.5442	0.0780	0.9220	0.0802	0.5146	0.0550	0.9450	0.0468	0.5442	0.0510	0.9490	0.0490	0.5146
	0.5	0.0950	0.9050	0.0805	0.4563	0.1020	0.8980	0.0769	0.4338	0.0660	0.9340	0.0478	0.4563	0.0670	0.9330	0.0454	0.4338
	0.6	0.0740	0.9260	0.0769	0.3767	0.0820	0.9180	0.0764	0.3616	0.0400	0.9600	0.0447	0.3767	0.0410	0.9590	0.0463	0.3616
	0.7	0.0870	0.9130	0.0735	0.2781	0.0890	0.9110	0.0736	0.2714	0.0470	0.9530	0.0450	0.2781	0.0460	0.9540	0.0436	0.2714
	0.8	0.0880	0.9120	0.0721	0.1955	0.0970	0.9030	0.0708	0.1930	0.0500	0.9500	0.0433	0.1955	0.0520	0.9480	0.0429	0.1930
	0.85	0.0920	0.9080	0.0652	0.1436	0.0960	0.9040	0.0651	0.1431	0.0440	0.9560	0.0385	0.1436	0.0420	0.9580	0.0384	0.1431
	0.9	0.1270	0.8730	0.0606	0.0987	0.1230	0.8770	0.0589	0.0990	0.0460	0.9540	0.0343	0.0987	0.0460	0.9540	0.0342	0.0990
	0.95	0.5760	0.4240	0.0577	0.0554	0.6630	0.3370	0.0577	0.0572	0.0460	0.9540	0.0291	0.0554	0.0520	0.9480	0.0294	0.0572
	0.99	0.9180	0.0820	0.0739	0.0165	0.9780	0.0220	0.0671	0.0180	0.8680	0.1320	0.0198	0.0165	0.9410	0.0590	0.0197	0.0180

Table 1. (continued)

		q=0.95								q=0.99							
		NW				LL				NW				LL			
γ	ρ	TDR	M	S	MCE	TDR	M	S	MCE	TDR	M	S	MCE	TDR	M	S	MCE
**0.30**	0.1	0.08	0.9200	0.0832	0.7224	0.084	0.9160	0.0857	0.6674	0.0510	0.9490	0.0517	0.7224	0.0470	0.9530	0.0544	0.6674
	0.2	0.088	0.9120	0.0799	0.6902	0.085	0.9150	0.0817	0.6386	0.0580	0.9420	0.0494	0.6902	0.0580	0.9420	0.0490	0.6386
	0.3	0.11	0.8900	0.0779	0.6175	0.097	0.9030	0.0766	0.5789	0.0680	0.9320	0.0470	0.6175	0.0510	0.9490	0.0464	0.5789
	0.4	0.123	0.8770	0.0789	0.5534	0.113	0.8870	0.0805	0.5252	0.0690	0.9310	0.0451	0.5534	0.0690	0.9310	0.0459	0.5252
	0.5	0.12	0.8800	0.0795	0.4646	0.116	0.8840	0.0776	0.4462	0.0680	0.9320	0.0478	0.4646	0.0690	0.9310	0.0464	0.4462
	0.6	0.103	0.8970	0.0766	0.3812	0.105	0.8950	0.0742	0.3657	0.0550	0.9450	0.0454	0.3812	0.0540	0.9460	0.0435	0.3657
	0.7	0.106	0.8940	0.0725	0.2881	0.105	0.8950	0.0726	0.2826	0.0630	0.9370	0.0437	0.2881	0.0600	0.9400	0.0435	0.2826
	0.8	0.119	0.8810	0.0682	0.1986	0.121	0.8790	0.0669	0.1967	0.0450	0.9550	0.0405	0.1986	0.0420	0.9580	0.0401	0.1967
	0.85	0.156	0.8440	0.0652	0.1550	0.179	0.8210	0.0643	0.1533	0.0660	0.9340	0.0393	0.1550	0.0660	0.9340	0.0386	0.1533
	0.9	0.391	0.6090	0.0607	0.1063	0.474	0.5260	0.0603	0.1076	0.0590	0.9410	0.0332	0.1063	0.0570	0.9430	0.0334	0.1076
	0.95	0.881	0.1190	0.0563	0.0625	0.93	0.0700	0.0553	0.0637	0.2390	0.7610	0.0283	0.0625	0.2630	0.7370	0.0279	0.0637
	0.99	0.941	0.0590	0.0971	0.0256	0.994	0.0060	0.0817	0.0273	0.9210	0.0790	0.0252	0.0256	0.9830	0.0170	0.0215	0.0273
**0.40**	0.1	0.087	0.9130	0.0805	0.7335	0.082	0.9180	0.0817	0.6659	0.0500	0.9500	0.0492	0.7335	0.0530	0.9470	0.0497	0.6659
	0.2	0.088	0.9120	0.0750	0.6784	0.09	0.9100	0.0783	0.6376	0.0580	0.9420	0.0467	0.6784	0.0530	0.9470	0.0483	0.6376
	0.3	0.088	0.9120	0.0804	0.6321	0.098	0.9020	0.0805	0.5901	0.0480	0.9520	0.0476	0.6321	0.0580	0.9420	0.0486	0.5901
	0.4	0.127	0.8730	0.0773	0.5569	0.114	0.8860	0.0768	0.5279	0.0780	0.9220	0.0456	0.5569	0.0730	0.9270	0.0458	0.5279
	0.5	0.148	0.8520	0.0762	0.4673	0.151	0.8490	0.0744	0.4451	0.0930	0.9070	0.0442	0.4673	0.0950	0.9050	0.0441	0.4451
	0.6	0.127	0.8730	0.0745	0.3835	0.12	0.8800	0.0751	0.3704	0.0610	0.9390	0.0425	0.3835	0.0640	0.9360	0.0435	0.3704
	0.7	0.159	0.8410	0.0741	0.3001	0.145	0.8550	0.0723	0.2915	0.0660	0.9340	0.0437	0.3001	0.0680	0.9320	0.0419	0.2915
	0.8	0.229	0.7710	0.0675	0.2098	0.238	0.7620	0.0676	0.2072	0.0930	0.9070	0.0421	0.2098	0.0940	0.9060	0.0401	0.2072
	0.85	0.382	0.6180	0.0651	0.1661	0.415	0.5850	0.0652	0.1644	0.0800	0.9200	0.0383	0.1661	0.0870	0.9130	0.0378	0.1644
	0.9	0.773	0.2270	0.0598	0.1181	0.801	0.1990	0.0586	0.1184	0.1890	0.8110	0.0327	0.1181	0.1880	0.8120	0.0319	0.1184
	0.95	0.934	0.0660	0.0546	0.0697	0.963	0.0370	0.0530	0.0712	0.7440	0.2560	0.0245	0.0697	0.7990	0.2010	0.0236	0.0712
	0.99	0.948	0.0520	0.1075	0.0366	0.997	0.0030	0.0893	0.0388	0.9380	0.0620	0.0281	0.0366	0.9930	0.0070	0.0236	0.0388

Table 1. (continued)

		**q=0.95**								**q=0.99**							
		NW				LL				NW				LL			
γ	ρ	TDR	M	S	MCE	TDR	M	S	MCE	TDR	M	S	MCE	TDR	M	S	MCE
	0.1	0.073	0.9270	0.0790	0.7288	0.095	0.9050	0.0834	0.6694	0.0530	0.9470	0.0482	0.7288	0.0600	0.9400	0.0507	0.6694
	0.2	0.101	0.8990	0.0757	0.6873	0.108	0.8920	0.0782	0.6362	0.0630	0.9370	0.0449	0.6873	0.0740	0.9260	0.0474	0.6362
	0.3	0.113	0.8870	0.0766	0.6315	0.105	0.8950	0.0771	0.5873	0.0550	0.9450	0.0462	0.6315	0.0660	0.9340	0.0472	0.5873
	0.4	0.14	0.8600	0.0787	0.5592	0.133	0.8670	0.0806	0.5278	0.0860	0.9140	0.0451	0.5592	0.0750	0.9250	0.0460	0.5278
**0.50**	0.5	0.175	0.8250	0.0802	0.4823	0.17	0.8300	0.0796	0.4624	0.1020	0.8980	0.0479	0.4823	0.0950	0.9050	0.0467	0.4624
	0.6	0.193	0.8070	0.0766	0.3988	0.198	0.8020	0.0747	0.3824	0.1110	0.8890	0.0458	0.3988	0.1100	0.8900	0.0448	0.3824
	0.7	0.272	0.7280	0.0736	0.3107	0.268	0.7320	0.0711	0.2996	0.1180	0.8820	0.0434	0.3107	0.1110	0.8890	0.0409	0.2996
	0.8	0.459	0.5410	0.0704	0.2221	0.484	0.5160	0.0694	0.2186	0.1580	0.8420	0.0431	0.2221	0.1560	0.8440	0.0413	0.2186
	0.85	0.678	0.3220	0.0633	0.1728	0.711	0.2890	0.0614	0.1719	0.2080	0.7920	0.0379	0.1728	0.2050	0.7950	0.0371	0.1719
	0.9	0.892	0.1080	0.0633	0.1349	0.916	0.0840	0.0604	0.1348	0.5780	0.4220	0.0352	0.1349	0.5950	0.4050	0.0354	0.1348
	0.95	0.956	0.0440	0.0578	0.0861	0.981	0.0190	0.0544	0.0880	0.9030	0.0970	0.0268	0.0861	0.9340	0.0660	0.0264	0.0880
	0.99	0.962	0.0380	0.1293	0.0522	0.994	0.0060	0.0996	0.0546	0.9500	0.0500	0.0314	0.0522	0.9920	0.0080	0.0263	0.0546
	0.1	0.0900	0.9100	0.0831	0.7222	0.107	0.8930	0.0817	0.6690	0.0580	0.9420	0.0510	0.7222	0.0540	0.9460	0.0515	0.6690
	0.2	0.1010	0.8990	0.0792	0.6899	0.101	0.8990	0.0793	0.6429	0.0630	0.9370	0.0490	0.6899	0.0560	0.9440	0.0484	0.6429
	0.3	0.1430	0.8570	0.0815	0.6315	0.134	0.8660	0.0801	0.5928	0.0890	0.9110	0.0495	0.6315	0.0880	0.9120	0.0490	0.5928
	0.4	0.1840	0.8160	0.0757	0.5603	0.171	0.8290	0.0741	0.5285	0.1100	0.8900	0.0435	0.5603	0.1140	0.8860	0.0430	0.5285
**0.60**	0.5	0.2350	0.7650	0.0765	0.4881	0.226	0.7740	0.0761	0.4638	0.1200	0.8800	0.0442	0.4881	0.1300	0.8700	0.0445	0.4638
	0.6	0.2840	0.7160	0.0748	0.4010	0.276	0.7240	0.0739	0.3895	0.1440	0.8560	0.0443	0.4010	0.1360	0.8640	0.0445	0.3895
	0.7	0.4100	0.5900	0.0751	0.3234	0.407	0.5930	0.0726	0.3140	0.1750	0.8250	0.0451	0.3234	0.1740	0.8260	0.0427	0.3140
	0.8	0.6950	0.3050	0.0685	0.2330	0.725	0.2750	0.0667	0.2291	0.2880	0.7120	0.0404	0.2330	0.2890	0.7110	0.0394	0.2291
	0.85	0.8400	0.1600	0.0609	0.1883	0.84	0.1600	0.0595	0.1858	0.5260	0.4740	0.0364	0.1883	0.5260	0.4740	0.0358	0.1858
	0.9	0.9300	0.0700	0.0605	0.1457	0.942	0.0580	0.0595	0.1448	0.7900	0.2100	0.0322	0.1457	0.8190	0.1810	0.0312	0.1448
	0.95	0.9590	0.0410	0.0598	0.1017	0.981	0.0190	0.0557	0.1039	0.9340	0.0660	0.0273	0.1017	0.9670	0.0330	0.0268	0.1039
	0.99	0.9470	0.0530	0.1254	0.0662	0.994	0.0060	0.1016	0.0691	0.9420	0.0580	0.0306	0.0662	0.9920	0.0080	0.0254	0.0691

Table 1. (continued)

		q=0.95								q=0.99							
		NW				LL				NW				LL			
γ	ρ	TDR	M	S	MCE	TDR	M	S	MCE	TDR	M	S	MCE	TDR	M	S	MCE
**0.70**	0.1	0.0960	0.9040	0.0793	0.7146	0.078	0.9220	0.0812	0.6633	0.054	0.9460	0.0497	0.7146	0.0490	0.9510	0.0482	0.6633
	0.2	0.1100	0.8900	0.0745	0.6911	0.113	0.8870	0.0772	0.6441	0.065	0.9350	0.0474	0.6911	0.0670	0.9330	0.0462	0.6441
	0.3	0.1560	0.8440	0.0738	0.6370	0.158	0.8420	0.0741	0.5868	0.094	0.9060	0.0435	0.6370	0.0990	0.9010	0.0445	0.5868
	0.4	0.2350	0.7650	0.0758	0.5642	0.22	0.7800	0.0757	0.5301	0.14	0.8600	0.0426	0.5642	0.1290	0.8710	0.0448	0.5301
	0.5	0.2750	0.7250	0.0765	0.4888	0.267	0.7330	0.0738	0.4650	0.156	0.8440	0.0459	0.4888	0.1550	0.8450	0.0437	0.4650
	0.6	0.4300	0.5700	0.0742	0.4151	0.437	0.5630	0.0735	0.3993	0.249	0.7510	0.0428	0.4151	0.2420	0.7580	0.0412	0.3993
	0.7	0.5740	0.4260	0.0730	0.3243	0.579	0.4210	0.0705	0.3161	0.316	0.6840	0.0418	0.3243	0.3020	0.6980	0.0411	0.3161
	0.8	0.8100	0.1900	0.0689	0.2468	0.815	0.1850	0.0674	0.2428	0.589	0.4110	0.0419	0.2468	0.6010	0.3990	0.0404	0.2428
	0.85	0.8970	0.1030	0.0617	0.1989	0.911	0.0890	0.0622	0.1972	0.74	0.2600	0.0360	0.1989	0.7590	0.2410	0.0347	0.1972
	0.9	0.9480	0.0520	0.0598	0.1592	0.957	0.0430	0.0586	0.1597	0.897	0.1030	0.0342	0.1592	0.9070	0.0930	0.0333	0.1597
	0.95	0.9620	0.0380	0.0571	0.1121	0.984	0.0160	0.0552	0.1141	0.95	0.0500	0.0255	0.1121	0.9750	0.0250	0.0245	0.1141
	0.99	0.9720	0.0280	0.1081	0.0804	0.993	0.0070	0.0887	0.0831	0.961	0.0390	0.0278	0.0804	0.9910	0.0090	0.0247	0.0831
**0.80**	0.1	0.0820	0.9180	0.0806	0.7177	0.0860	0.9140	0.0811	0.6712	0.0630	0.9370	0.0490	0.7177	0.0580	0.9420	0.0505	0.6712
	0.2	0.1340	0.8660	0.0755	0.6897	0.1230	0.8770	0.0756	0.6386	0.0910	0.9090	0.0474	0.6897	0.0790	0.9210	0.0438	0.6386
	0.3	0.1650	0.8350	0.0791	0.6408	0.1710	0.8290	0.0787	0.5968	0.1010	0.8990	0.0465	0.6408	0.1110	0.8890	0.0486	0.5968
	0.4	0.2750	0.7250	0.0761	0.5689	0.2690	0.7310	0.0748	0.5355	0.1740	0.8260	0.0436	0.5689	0.1830	0.8170	0.0437	0.5355
	0.5	0.4190	0.5810	0.0782	0.5013	0.4060	0.5940	0.0759	0.4755	0.2600	0.7400	0.0456	0.5013	0.2640	0.7360	0.0453	0.4755
	0.6	0.5330	0.4670	0.0724	0.4105	0.5320	0.4680	0.0696	0.3920	0.3380	0.6620	0.0423	0.4105	0.3570	0.6430	0.0426	0.3920
	0.7	0.6940	0.3060	0.0742	0.3416	0.6930	0.3070	0.0723	0.3306	0.4920	0.5080	0.0444	0.3416	0.4920	0.5080	0.0420	0.3306
	0.8	0.8910	0.1090	0.0688	0.2572	0.8850	0.1150	0.0673	0.2533	0.7620	0.2380	0.0408	0.2572	0.7600	0.2400	0.0398	0.2533
	0.85	0.9240	0.0760	0.0628	0.2079	0.9280	0.0720	0.0611	0.2064	0.8450	0.1550	0.0371	0.2079	0.8550	0.1450	0.0362	0.2064
	0.9	0.9630	0.0370	0.0605	0.1670	0.9690	0.0310	0.0583	0.1666	0.9290	0.0710	0.0332	0.1670	0.9280	0.0720	0.0316	0.1666
	0.95	0.9750	0.0250	0.0584	0.1279	0.9860	0.0140	0.0557	0.1288	0.9670	0.0330	0.0275	0.1279	0.9810	0.0190	0.0267	0.1288
	0.99	0.9690	0.0310	0.1050	0.0921	0.9870	0.0130	0.0837	0.0946	0.9660	0.0340	0.0270	0.0921	0.9870	0.0130	0.0223	0.0946

Table 1. (continued)

		q=0.95								q=0.99							
		NW				LL				NW				LL			
γ	ρ	TDR	M	S	MCE	TDR	M	S	MCE	TDR	M	S	MCE	TDR	M	S	MCE
**0.85**	0.1	0.1110	0.8890	0.0837	0.7258	0.099	0.901	0.0820	0.6709	0.073	0.927	0.0507	0.7258	0.062	0.938	0.0507	0.6709
	0.2	0.1280	0.8720	0.0742	0.6944	0.132	0.868	0.0732	0.6402	0.091	0.909	0.0447	0.6944	0.083	0.917	0.0460	0.6402
	0.3	0.2150	0.7850	0.0785	0.6414	0.205	0.795	0.0807	0.5975	0.121	0.879	0.0472	0.6414	0.131	0.869	0.0493	0.5975
	0.4	0.3110	0.6890	0.0759	0.5717	0.294	0.706	0.0783	0.5363	0.192	0.808	0.0441	0.5717	0.172	0.828	0.0468	0.5363
	0.5	0.4200	0.5800	0.0766	0.5005	0.427	0.573	0.0754	0.4758	0.281	0.719	0.0450	0.5005	0.281	0.719	0.0443	0.4758
	0.6	0.5840	0.4160	0.0744	0.4221	0.575	0.425	0.0727	0.4072	0.413	0.587	0.0436	0.4221	0.407	0.593	0.0429	0.4072
	0.7	0.7380	0.2620	0.0734	0.3440	0.735	0.265	0.0719	0.3326	0.572	0.428	0.0448	0.3440	0.568	0.432	0.0424	0.3326
	0.8	0.8890	0.1110	0.0694	0.2590	0.878	0.122	0.0680	0.2533	0.799	0.201	0.0398	0.2590	0.79	0.21	0.0391	0.2533
	0.85	0.9310	0.0690	0.0649	0.2205	0.935	0.065	0.0637	0.2186	0.89	0.11	0.0402	0.2205	0.884	0.116	0.0395	0.2186
	0.9	0.9500	0.0500	0.0614	0.1725	0.964	0.036	0.0592	0.1733	0.926	0.074	0.0338	0.1725	0.939	0.061	0.0331	0.1733
	0.95	0.9760	0.0240	0.0596	0.1309	0.985	0.015	0.0564	0.1322	0.958	0.042	0.0277	0.1309	0.969	0.031	0.0268	0.1322
	0.99	0.9550	0.0450	0.0924	0.0966	0.99	0.01	0.0801	0.1007	0.953	0.047	0.0255	0.0966	0.99	0.01	0.0237	0.1007
**0.90**	0.1	0.1010	0.8990	0.0772	0.7333	0.0970	0.9030	0.0816	0.6785	0.0620	0.9380	0.0484	0.7333	0.0600	0.9400	0.0467	0.6785
	0.2	0.1430	0.8570	0.0739	0.6874	0.1340	0.8660	0.0748	0.6406	0.0880	0.9120	0.0450	0.6874	0.0790	0.9210	0.0455	0.6406
	0.3	0.2030	0.7970	0.0745	0.6328	0.2070	0.7930	0.0750	0.5887	0.1380	0.8620	0.0426	0.6328	0.1370	0.8630	0.0453	0.5887
	0.4	0.3000	0.7000	0.0718	0.5631	0.3000	0.7000	0.0742	0.5295	0.1980	0.8020	0.0423	0.5631	0.1980	0.8020	0.0446	0.5295
	0.5	0.4500	0.5500	0.0738	0.5000	0.4260	0.5740	0.0746	0.4767	0.2860	0.7140	0.0442	0.5000	0.2820	0.7180	0.0438	0.4767
	0.6	0.6020	0.3980	0.0748	0.4293	0.6060	0.3940	0.0739	0.4106	0.4420	0.5580	0.0438	0.4293	0.4370	0.5630	0.0435	0.4106
	0.7	0.7900	0.2100	0.0741	0.3427	0.7800	0.2200	0.0722	0.3330	0.6430	0.3570	0.0448	0.3427	0.6330	0.3670	0.0432	0.3330
	0.8	0.9140	0.0860	0.0666	0.2601	0.9090	0.0910	0.0652	0.2557	0.8440	0.1560	0.0398	0.2601	0.8380	0.1620	0.0382	0.2557
	0.85	0.9270	0.0730	0.0619	0.2143	0.9250	0.0750	0.0623	0.2139	0.8910	0.1090	0.0367	0.2143	0.8900	0.1100	0.0360	0.2139
	0.9	0.9630	0.0370	0.0606	0.1772	0.9720	0.0280	0.0591	0.1776	0.9410	0.0590	0.0325	0.1772	0.9480	0.0520	0.0318	0.1776
	0.95	0.9770	0.0230	0.0582	0.1350	0.9810	0.0190	0.0564	0.1362	0.9630	0.0370	0.0284	0.1350	0.9710	0.0290	0.0278	0.1362
	0.99	0.9440	0.0560	0.0774	0.0982	0.9910	0.0090	0.0682	0.1030	0.9380	0.0620	0.0225	0.0982	0.9900	0.0100	0.0198	0.1030

Table 2. Simulation results for n=20, %10 percentage of contamination

		**q=0.95**								**q=0.99**							
		NW				LL				NW				LL			
γ	ρ	TDR	M	S	MCE	TDR	M	S	MCE	TDR	M	S	MCE	TDR	M	S	MCE
**0.10**	0.1	0.0905	0.9095	0.0837	0.7232	0.0855	0.9145	0.0829	0.6766	0.0555	0.9445	0.0519	0.7232	0.0455	0.9545	0.0513	0.6766
	0.2	0.0655	0.9345	0.0786	0.6809	0.0690	0.9310	0.0779	0.6317	0.0425	0.9575	0.0476	0.6809	0.0405	0.9595	0.0474	0.6317
	0.3	0.0705	0.9295	0.0808	0.6237	0.0760	0.9240	0.0793	0.5843	0.0430	0.9570	0.0486	0.6237	0.0450	0.9550	0.0479	0.5843
	0.4	0.0915	0.9085	0.0793	0.5429	0.0860	0.9140	0.0811	0.5164	0.0550	0.9450	0.0467	0.5429	0.0465	0.9535	0.0474	0.5164
	0.5	0.0970	0.9030	0.0827	0.4674	0.0955	0.9045	0.0789	0.4471	0.0590	0.9410	0.0492	0.4674	0.0520	0.9480	0.0488	0.4471
	0.6	0.0805	0.9195	0.0803	0.3746	0.0770	0.9230	0.0797	0.3619	0.0465	0.9535	0.0496	0.3746	0.0455	0.9545	0.0499	0.3619
	0.7	0.0865	0.9135	0.0734	0.2805	0.0835	0.9165	0.0734	0.2731	0.0505	0.9495	0.0461	0.2805	0.0460	0.9540	0.0445	0.2731
	0.8	0.0765	0.9235	0.0689	0.1849	0.0705	0.9295	0.0694	0.1825	0.0395	0.9605	0.0396	0.1849	0.0375	0.9625	0.0389	0.1825
	0.85	0.0760	0.9240	0.0621	0.1365	0.0745	0.9255	0.0620	0.1370	0.0455	0.9545	0.0363	0.1365	0.0430	0.9570	0.0358	0.1370
	0.9	0.0620	0.9380	0.0603	0.0927	0.0615	0.9385	0.0601	0.0939	0.0325	0.9675	0.0333	0.0927	0.0345	0.9655	0.0329	0.0939
	0.95	0.0790	0.9210	0.0558	0.0477	0.0775	0.9225	0.0568	0.0494	0.0260	0.9740	0.0273	0.0477	0.0250	0.9750	0.0276	0.0494
	0.99	0.7820	0.2180	0.0625	0.0115	0.8765	0.1235	0.0591	0.0121	0.1505	0.8495	0.0179	0.0115	0.2230	0.7770	0.0172	0.0121
**0.20**	0.1	0.0850	0.9150	0.0821	0.7286	0.0850	0.9150	0.0820	0.6638	0.0515	0.9485	0.0503	0.7286	0.0535	0.9465	0.0509	0.6638
	0.2	0.0925	0.9075	0.0771	0.6790	0.0890	0.9110	0.0783	0.6396	0.0560	0.9440	0.0483	0.6790	0.0505	0.9495	0.0452	0.6396
	0.3	0.0835	0.9165	0.0814	0.6238	0.0805	0.9195	0.0798	0.5844	0.0535	0.9465	0.0509	0.6238	0.0535	0.9465	0.0489	0.5844
	0.4	0.0840	0.9160	0.0772	0.5437	0.0815	0.9185	0.0757	0.5158	0.0470	0.9530	0.0464	0.5437	0.0465	0.9535	0.0470	0.5158
	0.5	0.0905	0.9095	0.0776	0.4684	0.0855	0.9145	0.0771	0.4453	0.0535	0.9465	0.0454	0.4684	0.0530	0.9470	0.0446	0.4453
	0.6	0.0845	0.9155	0.0771	0.3771	0.0875	0.9125	0.0762	0.3625	0.0505	0.9495	0.0485	0.3771	0.0495	0.9505	0.0458	0.3625
	0.7	0.0870	0.9130	0.0711	0.2848	0.0900	0.9100	0.0703	0.2774	0.0535	0.9465	0.0409	0.2848	0.0570	0.9430	0.0406	0.2774
	0.8	0.0875	0.9125	0.0652	0.1924	0.0895	0.9105	0.0646	0.1900	0.0505	0.9495	0.0368	0.1924	0.0500	0.9500	0.0366	0.1900
	0.85	0.0910	0.9090	0.0597	0.1459	0.0920	0.9080	0.0604	0.1463	0.0480	0.9520	0.0359	0.1459	0.0460	0.9540	0.0357	0.1463
	0.9	0.1180	0.8820	0.0599	0.1068	0.1175	0.8825	0.0581	0.1068	0.0500	0.9500	0.0335	0.1068	0.0540	0.9460	0.0319	0.1068
	0.95	0.4840	0.5160	0.0554	0.0569	0.5475	0.4525	0.0537	0.0580	0.0320	0.9680	0.0253	0.0569	0.0295	0.9705	0.0256	0.0580
	0.99	0.9110	0.0890	0.1037	0.0220	0.9705	0.0295	0.0859	0.0235	0.8335	0.1665	0.0237	0.0220	0.9070	0.0930	0.0197	0.0235

Table 2. (continued)

		**q=0.95**								**q=0.99**							
		NW				LL				NW				LL			
γ	ρ	TDR	M	S	MCE	TDR	M	S	MCE	TDR	M	S	MCE	TDR	M	S	MCE
**0.30**	0.1	0.0925	0.9075	0.0816	0.7270	0.0870	0.9130	0.0825	0.6762	0.0600	0.9400	0.0515	0.7270	0.0515	0.9485	0.0497	0.6762
	0.2	0.0825	0.9175	0.0778	0.6810	0.0885	0.9115	0.0793	0.6284	0.0525	0.9475	0.0486	0.6810	0.0565	0.9435	0.0481	0.6284
	0.3	0.0860	0.9140	0.0758	0.6283	0.0845	0.9155	0.0792	0.5888	0.0515	0.9485	0.0454	0.6283	0.0455	0.9545	0.0466	0.5888
	0.4	0.0970	0.9030	0.0749	0.5566	0.0940	0.9060	0.0759	0.5270	0.0530	0.9470	0.0441	0.5566	0.0575	0.9425	0.0457	0.5270
	0.5	0.1050	0.8950	0.0754	0.4721	0.1065	0.8935	0.0731	0.4479	0.0650	0.9350	0.0434	0.4721	0.0670	0.9330	0.0438	0.4479
	0.6	0.1120	0.8880	0.0761	0.3938	0.1090	0.8910	0.0753	0.3742	0.0660	0.9340	0.0451	0.3938	0.0665	0.9335	0.0463	0.3742
	0.7	0.0990	0.9010	0.0735	0.3082	0.1000	0.9000	0.0715	0.2966	0.0555	0.9445	0.0424	0.3082	0.0555	0.9445	0.0412	0.2966
	0.8	0.1160	0.8840	0.0682	0.2141	0.1175	0.8825	0.0656	0.2090	0.0570	0.9430	0.0416	0.2141	0.0555	0.9445	0.0394	0.2090
	0.85	0.1325	0.8675	0.0615	0.1669	0.1285	0.8715	0.0616	0.1660	0.0480	0.9520	0.0358	0.1669	0.0475	0.9525	0.0354	0.1660
	0.9	0.3180	0.6820	0.0587	0.1201	0.3295	0.6705	0.0573	0.1204	0.0590	0.9410	0.0322	0.1201	0.0575	0.9425	0.0327	0.1204
	0.95	0.8305	0.1695	0.0554	0.0755	0.8795	0.1205	0.0511	0.0767	0.1685	0.8315	0.0269	0.0755	0.1545	0.8455	0.0262	0.0767
	0.99	0.9470	0.0530	0.1564	0.0412	0.9755	0.0245	0.1179	0.0424	0.9205	0.0795	0.0382	0.0412	0.9610	0.0390	0.0288	0.0424
**0.40**	0.1	0.0970	0.9030	0.0777	0.7236	0.1045	0.8955	0.0831	0.6714	0.0620	0.9380	0.0486	0.7236	0.0615	0.9385	0.0516	0.6714
	0.2	0.0945	0.9055	0.0757	0.6955	0.1030	0.8970	0.0747	0.6495	0.0600	0.9400	0.0454	0.6955	0.0635	0.9365	0.0442	0.6495
	0.3	0.0860	0.9140	0.0803	0.6337	0.0860	0.9140	0.0757	0.5867	0.0540	0.9460	0.0487	0.6337	0.0565	0.9435	0.0457	0.5867
	0.4	0.0950	0.9050	0.0793	0.5660	0.0990	0.9010	0.0794	0.5335	0.0510	0.9490	0.0460	0.5660	0.0525	0.9475	0.0483	0.5335
	0.5	0.1255	0.8745	0.0746	0.4858	0.1395	0.8605	0.0752	0.4651	0.0690	0.9310	0.0411	0.4858	0.0730	0.9270	0.0426	0.4651
	0.6	0.1290	0.8710	0.0704	0.4001	0.1270	0.8730	0.0701	0.3862	0.0785	0.9215	0.0405	0.4001	0.0790	0.9210	0.0401	0.3862
	0.7	0.1290	0.8710	0.0714	0.3180	0.1370	0.8630	0.0697	0.3105	0.0630	0.9370	0.0408	0.3180	0.0605	0.9395	0.0392	0.3105
	0.8	0.1970	0.8030	0.0654	0.2323	0.2050	0.7950	0.0653	0.2274	0.0765	0.9235	0.0393	0.2323	0.0740	0.9260	0.0384	0.2274
	0.85	0.3090	0.6910	0.0599	0.1825	0.3225	0.6775	0.0599	0.1813	0.0750	0.9250	0.0357	0.1825	0.0745	0.9255	0.0352	0.1813
	0.9	0.6590	0.3410	0.0624	0.1456	0.7070	0.2930	0.0598	0.1449	0.1190	0.8810	0.0345	0.1456	0.1125	0.8875	0.0337	0.1449
	0.95	0.9080	0.0920	0.0610	0.0994	0.9400	0.0600	0.0544	0.1007	0.6235	0.3765	0.0264	0.0994	0.6905	0.3095	0.0253	0.1007
	0.99	0.9750	0.0250	0.2140	0.0648	0.9795	0.0205	0.1482	0.0655	0.9630	0.0370	0.0486	0.0648	0.9730	0.0270	0.0332	0.0655

Table 2. (continued)

		q=0.95								q=0.99							
		NW				LL				NW				LL			
γ	ρ	TDR	M	S	MCE	TDR	M	S	MCE	TDR	M	S	MCE	TDR	M	S	MCE
**0.50**	0.1	0.0845	0.9155	0.0754	0.7272	0.0885	0.9115	0.0844	0.6690	0.0465	0.9535	0.0469	0.7272	0.0570	0.9430	0.0492	0.6690
	0.2	0.0960	0.9040	0.0769	0.6928	0.0935	0.9065	0.0744	0.6402	0.0540	0.9460	0.0468	0.6928	0.0525	0.9475	0.0446	0.6402
	0.3	0.1085	0.8915	0.0777	0.6379	0.1085	0.8915	0.0757	0.5976	0.0695	0.9305	0.0464	0.6379	0.0710	0.9290	0.0453	0.5976
	0.4	0.1400	0.8600	0.0727	0.5806	0.1385	0.8615	0.0764	0.5476	0.0760	0.9240	0.0413	0.5806	0.0765	0.9235	0.0434	0.5476
	0.5	0.1705	0.8295	0.0738	0.5007	0.1615	0.8385	0.0728	0.4749	0.1010	0.8990	0.0417	0.5007	0.1025	0.8975	0.0411	0.4749
	0.6	0.1800	0.8200	0.0750	0.4218	0.1860	0.8140	0.0741	0.4064	0.0955	0.9045	0.0424	0.4218	0.0940	0.9060	0.0420	0.4064
	0.7	0.2260	0.7740	0.0716	0.3391	0.2200	0.7800	0.0687	0.3272	0.1025	0.8975	0.0414	0.3391	0.1055	0.8945	0.0393	0.3272
	0.8	0.3750	0.6250	0.0643	0.2539	0.4020	0.5980	0.0640	0.2487	0.1245	0.8755	0.0377	0.2539	0.1215	0.8785	0.0357	0.2487
	0.85	0.6165	0.3835	0.0612	0.2116	0.6380	0.3620	0.0592	0.2061	0.1760	0.8240	0.0361	0.2116	0.1775	0.8225	0.0354	0.2061
	0.9	0.8535	0.1465	0.0606	0.1717	0.8690	0.1310	0.0573	0.1700	0.4145	0.5855	0.0337	0.1717	0.4185	0.5815	0.0321	0.1700
	0.95	0.9545	0.0455	0.0639	0.1277	0.9660	0.0340	0.0578	0.1282	0.8735	0.1265	0.0258	0.1277	0.8915	0.1085	0.0249	0.1282
	0.99	0.9755	0.0245	0.2517	0.0941	0.9850	0.0150	0.1732	0.0948	0.9695	0.0305	0.0535	0.0941	0.9790	0.0210	0.0387	0.0948
**0.60**	0.1	0.0775	0.9225	0.0791	0.7246	0.0880	0.9120	0.0833	0.6709	0.0500	0.9500	0.0501	0.7246	0.0545	0.9455	0.0504	0.6709
	0.2	0.0985	0.9015	0.0744	0.6925	0.1010	0.8990	0.0759	0.6351	0.0570	0.9430	0.0453	0.6925	0.0600	0.9400	0.0460	0.6351
	0.3	0.1315	0.8685	0.0765	0.6450	0.1335	0.8665	0.0776	0.6055	0.0770	0.9230	0.0453	0.6450	0.0805	0.9195	0.0473	0.6055
	0.4	0.1655	0.8345	0.0728	0.5818	0.1640	0.8360	0.0732	0.5438	0.1005	0.8995	0.0401	0.5818	0.1000	0.9000	0.0421	0.5438
	0.5	0.2055	0.7945	0.0714	0.5123	0.2010	0.7990	0.0723	0.4864	0.1080	0.8920	0.0402	0.5123	0.1145	0.8855	0.0418	0.4864
	0.6	0.2635	0.7365	0.0726	0.4377	0.2630	0.7370	0.0719	0.4196	0.1370	0.8630	0.0419	0.4377	0.1265	0.8735	0.0434	0.4196
	0.7	0.3715	0.6285	0.0692	0.3624	0.3755	0.6245	0.0666	0.3498	0.1755	0.8245	0.0396	0.3624	0.1790	0.8210	0.0386	0.3498
	0.8	0.6270	0.3730	0.0647	0.2808	0.6430	0.3570	0.0633	0.2747	0.2590	0.7410	0.0389	0.2808	0.2460	0.7540	0.0373	0.2747
	0.85	0.7840	0.2160	0.0627	0.2379	0.7895	0.2105	0.0599	0.2330	0.4015	0.5985	0.0365	0.2379	0.4110	0.5890	0.0348	0.2330
	0.9	0.9070	0.0930	0.0612	0.1983	0.9155	0.0845	0.0596	0.1977	0.7110	0.2890	0.0332	0.1983	0.7415	0.2585	0.0319	0.1977
	0.95	0.9735	0.0265	0.0661	0.1603	0.9790	0.0210	0.0581	0.1599	0.9420	0.0580	0.0289	0.1603	0.9540	0.0460	0.0272	0.1599
	0.99	0.9770	0.0230	0.2516	0.1261	0.9865	0.0135	0.1729	0.1265	0.9745	0.0255	0.0533	0.1261	0.9815	0.0185	0.0409	0.1265

Table 2. (continued)

		q=0.95								q=0.99							
		NW				LL				NW				LL			
γ	ρ	TDR	M	S	MCE	TDR	M	S	MCE	TDR	M	S	MCE	TDR	M	S	MCE
**0.70**	0.1	0.0925	0.9075	0.0822	0.7306	0.1000	0.9000	0.0804	0.6727	0.0545	0.9455	0.0515	0.7306	0.0570	0.9430	0.0493	0.6727
	0.2	0.1145	0.8855	0.0698	0.6940	0.1050	0.8950	0.0737	0.6458	0.0680	0.9320	0.0414	0.6940	0.0615	0.9385	0.0424	0.6458
	0.3	0.1385	0.8615	0.0741	0.6564	0.1405	0.8595	0.0758	0.6141	0.0730	0.9270	0.0425	0.6564	0.0815	0.9185	0.0434	0.6141
	0.4	0.2105	0.7895	0.0682	0.5880	0.1960	0.8040	0.0697	0.5525	0.1230	0.8770	0.0373	0.5880	0.1160	0.8840	0.0396	0.5525
	0.5	0.2730	0.7270	0.0732	0.5300	0.2680	0.7320	0.0707	0.5036	0.1615	0.8385	0.0400	0.5300	0.1495	0.8505	0.0399	0.5036
	0.6	0.3545	0.6455	0.0707	0.4509	0.3550	0.6450	0.0703	0.4323	0.1880	0.8120	0.0408	0.4509	0.2020	0.7980	0.0411	0.4323
	0.7	0.5270	0.4730	0.0682	0.3799	0.5275	0.4725	0.0662	0.3674	0.2970	0.7030	0.0401	0.3799	0.2815	0.7185	0.0382	0.3674
	0.8	0.7950	0.2050	0.0639	0.3025	0.7860	0.2140	0.0620	0.2940	0.5025	0.4975	0.0377	0.3025	0.5035	0.4965	0.0363	0.2940
	0.85	0.8670	0.1330	0.0614	0.2614	0.8625	0.1375	0.0601	0.2574	0.6795	0.3205	0.0368	0.2614	0.6905	0.3095	0.0352	0.2574
	0.9	0.9345	0.0655	0.0608	0.2225	0.9385	0.0615	0.0572	0.2203	0.8590	0.1410	0.0331	0.2225	0.8650	0.1350	0.0309	0.2203
	0.95	0.9735	0.0265	0.0664	0.1873	0.9720	0.0280	0.0603	0.1849	0.9595	0.0405	0.0266	0.1873	0.9545	0.0455	0.0250	0.1849
	0.99	0.9815	0.0185	0.2298	0.1542	0.9895	0.0105	0.1516	0.1554	0.9740	0.0260	0.0466	0.1542	0.9845	0.0155	0.0398	0.1554
**0.80**	0.1	0.0925	0.9075	0.0763	0.7252	0.0920	0.9080	0.0798	0.6661	0.0925	0.9075	0.0763	0.7252	0.0920	0.9080	0.0798	0.6661
	0.2	0.1315	0.8685	0.0678	0.6961	0.1275	0.8725	0.0702	0.6479	0.1315	0.8685	0.0678	0.6961	0.1275	0.8725	0.0702	0.6479
	0.3	0.1810	0.8190	0.0714	0.6553	0.1755	0.8245	0.0734	0.6135	0.1810	0.8190	0.0714	0.6553	0.1755	0.8245	0.0734	0.6135
	0.4	0.2395	0.7605	0.0691	0.6010	0.2305	0.7695	0.0716	0.5620	0.2395	0.7605	0.0691	0.6010	0.2305	0.7695	0.0716	0.5620
	0.5	0.3390	0.6610	0.0685	0.5347	0.3265	0.6735	0.0679	0.5046	0.3390	0.6610	0.0685	0.5347	0.3265	0.6735	0.0679	0.5046
	0.6	0.4775	0.5225	0.0696	0.4657	0.4675	0.5325	0.0689	0.4448	0.4775	0.5225	0.0696	0.4657	0.4675	0.5325	0.0689	0.4448
	0.7	0.6740	0.3260	0.0674	0.3927	0.6560	0.3440	0.0642	0.3777	0.6740	0.3260	0.0674	0.3927	0.6560	0.3440	0.0642	0.3777
	0.8	0.8710	0.1290	0.0612	0.3215	0.8620	0.1380	0.0596	0.3139	0.8710	0.1290	0.0612	0.3215	0.8620	0.1380	0.0596	0.3139
	0.85	0.9285	0.0715	0.0663	0.2920	0.9205	0.0795	0.0627	0.2869	0.9285	0.0715	0.0663	0.2920	0.9205	0.0795	0.0627	0.2869
	0.9	0.9495	0.0505	0.0580	0.2440	0.9565	0.0435	0.0556	0.2428	0.9495	0.0505	0.0580	0.2440	0.9565	0.0435	0.0556	0.2428
	0.95	0.9780	0.0220	0.0648	0.2112	0.9745	0.0255	0.0582	0.2092	0.9780	0.0220	0.0648	0.2112	0.9745	0.0255	0.0582	0.2092
	0.99	0.9860	0.0140	0.1821	0.1783	0.9895	0.0105	0.1340	0.1802	0.9860	0.0140	0.1821	0.1783	0.9895	0.0105	0.1340	0.1802

Table 2. (continued)

		**q=0.95**								**q=0.99**							
		NW				LL				NW				LL			
γ	ρ	TDR	M	S	MCE	TDR	M	S	MCE	TDR	M	S	MCE	TDR	M	S	MCE
**0.85**	0.1	0.0895	0.9105	0.0749	0.7161	0.0880	0.9120	0.0773	0.6680	0.0515	0.9485	0.0457	0.7161	0.0570	0.9430	0.0477	0.6680
	0.2	0.1195	0.8805	0.0763	0.7049	0.1210	0.8790	0.0744	0.6548	0.0755	0.9245	0.0458	0.7049	0.0750	0.9250	0.0439	0.6548
	0.3	0.1955	0.8045	0.0711	0.6637	0.1845	0.8155	0.0693	0.6150	0.1145	0.8855	0.0408	0.6637	0.1210	0.8790	0.0417	0.6150
	0.4	0.2585	0.7415	0.0650	0.5981	0.2490	0.7510	0.0712	0.5633	0.1550	0.8450	0.0377	0.5981	0.1505	0.8495	0.0387	0.5633
	0.5	0.3785	0.6215	0.0697	0.5430	0.3545	0.6455	0.0688	0.5154	0.2395	0.7605	0.0385	0.5430	0.2265	0.7735	0.0392	0.5154
	0.6	0.5345	0.4655	0.0716	0.4767	0.5100	0.4900	0.0712	0.4525	0.3560	0.6440	0.0418	0.4767	0.3475	0.6525	0.0412	0.4525
	0.7	0.7325	0.2675	0.0690	0.4010	0.7195	0.2805	0.0673	0.3861	0.5450	0.4550	0.0396	0.4010	0.5320	0.4680	0.0387	0.3861
	0.8	0.8755	0.1245	0.0664	0.3346	0.8660	0.1340	0.0655	0.3285	0.7640	0.2360	0.0401	0.3346	0.7535	0.2465	0.0393	0.3285
	0.85	0.9325	0.0675	0.0608	0.2940	0.9225	0.0775	0.0595	0.2883	0.8685	0.1315	0.0376	0.2940	0.8615	0.1385	0.0343	0.2883
	0.9	0.9560	0.0440	0.0608	0.2547	0.9430	0.0570	0.0582	0.2519	0.9245	0.0755	0.0344	0.2547	0.9145	0.0855	0.0317	0.2519
	0.95	0.9730	0.0270	0.0603	0.2192	0.9735	0.0265	0.0556	0.2190	0.9645	0.0355	0.0282	0.2192	0.9660	0.0340	0.0276	0.2190
	0.99	0.9785	0.0215	0.1474	0.1876	0.9810	0.0190	0.1111	0.1885	0.9760	0.0240	0.0385	0.1876	0.9800	0.0200	0.0312	0.1885
**0.90**	0.1	0.0865	0.9135	0.0781	0.7327	0.1035	0.8965	0.0774	0.6727	0.0535	0.9465	0.0469	0.7327	0.0570	0.9430	0.0463	0.6727
	0.2	0.1160	0.8840	0.0787	0.7059	0.1225	0.8775	0.0755	0.6571	0.0745	0.9255	0.0479	0.7059	0.0695	0.9305	0.0439	0.6571
	0.3	0.1765	0.8235	0.0667	0.6635	0.1750	0.8250	0.0707	0.6217	0.1120	0.8880	0.0392	0.6635	0.1110	0.8890	0.0404	0.6217
	0.4	0.2860	0.7140	0.0678	0.6069	0.2690	0.7310	0.0688	0.5652	0.1700	0.8300	0.0377	0.6069	0.1650	0.8350	0.0390	0.5652
	0.5	0.4175	0.5825	0.0723	0.5416	0.3825	0.6175	0.0708	0.5162	0.2615	0.7385	0.0389	0.5416	0.2595	0.7405	0.0381	0.5162
	0.6	0.5740	0.4260	0.0697	0.4784	0.5530	0.4470	0.0678	0.4582	0.4090	0.5910	0.0396	0.4784	0.3850	0.6150	0.0394	0.4582
	0.7	0.7680	0.2320	0.0699	0.4086	0.7570	0.2430	0.0678	0.3951	0.6085	0.3915	0.0421	0.4086	0.5910	0.4090	0.0401	0.3951
	0.8	0.8905	0.1095	0.0616	0.3320	0.8775	0.1225	0.0596	0.3242	0.7990	0.2010	0.0372	0.3320	0.7945	0.2055	0.0356	0.3242
	0.85	0.9400	0.0600	0.0627	0.3018	0.9365	0.0635	0.0611	0.2980	0.8950	0.1050	0.0361	0.3018	0.8945	0.1055	0.0341	0.2980
	0.9	0.9605	0.0395	0.0589	0.2625	0.9560	0.0440	0.0567	0.2614	0.9425	0.0575	0.0332	0.2625	0.9395	0.0605	0.0326	0.2614
	0.95	0.9705	0.0295	0.0590	0.2222	0.9715	0.0285	0.0571	0.2234	0.9575	0.0425	0.0264	0.2222	0.9635	0.0365	0.0258	0.2234
	0.99	0.9780	0.0220	0.1176	0.1942	0.9855	0.0145	0.0959	0.1971	0.9725	0.0275	0.0308	0.1942	0.9845	0.0155	0.0258	0.1971
