# Supplementary material for: A method for detecting outliers in linear-circular non-parametric regression
Source: PLoS One. 2023 Jun 12;18(6):e0286448. doi: 10.1371/journal.pone.0286448 (PMC10259788; doi:10.1371/journal.pone.0286448)
Supplement: S2 File — (PDF) [file pone.0286448.s002.pdf]

A Method for Detecting Outliers in Linear-Circular Non-Parametric Regression

Sümeyra Sert[1]* *and* Filiz Kardiyen[2]

[1] *Selcuk University, Department of Statistics, 42250, Selcuklu, Konya, Turkey;*

[1]ORCID id: https://orcid.org/0000-0002-4647-1583

sumeyra.sert@selcuk.edu.tr

[2]*Gazi University, Department of Statistics, Teknikokullar, 06500, Ankara, Turkey.*

[2]ORCID id: https://orcid.org/0000-0002-8730-2751

fyuva@gazi.edu.tr

Supplementary File (n=40)

Table 1. Simulation results for n=40, %1 percentage of contamination

		**q=0.95**								**q=0.99**							
		NW				LL				NW				LL			
γ	ρ	TDR	M	S	MCE	TDR	M	S	MCE	TDR	M	S	MCE	TDR	M	S	MCE
**0.10**	0.1	0.0650	0.9350	0.0625	0.7903	0.0660	0.9340	0.0659	0.7535	0.0320	0.9680	0.0322	0.7903	0.0360	0.9640	0.0319	0.7535
	0.2	0.0550	0.9450	0.0646	0.7366	0.0590	0.9410	0.0675	0.7102	0.0220	0.9780	0.0320	0.7366	0.0240	0.9760	0.0331	0.7102
	0.3	0.0650	0.9350	0.0682	0.6651	0.0670	0.9330	0.0686	0.6459	0.0410	0.9590	0.0328	0.6651	0.0320	0.9680	0.0336	0.6459
	0.4	0.0650	0.9350	0.0689	0.5733	0.0730	0.9270	0.0690	0.5598	0.0260	0.9740	0.0308	0.5733	0.0290	0.9710	0.0312	0.5598
	0.5	0.0730	0.9270	0.0714	0.4790	0.0690	0.9310	0.0698	0.4717	0.0360	0.9640	0.0338	0.4790	0.0310	0.9690	0.0319	0.4717
	0.6	0.0660	0.9340	0.0666	0.3859	0.0700	0.9300	0.0665	0.3843	0.0370	0.9630	0.0311	0.3859	0.0350	0.9650	0.0317	0.3843
	0.7	0.0770	0.9230	0.0652	0.2849	0.0740	0.9260	0.0648	0.2864	0.0380	0.9620	0.0292	0.2849	0.0370	0.9630	0.0292	0.2864
	0.8	0.0560	0.9440	0.0649	0.1930	0.0550	0.9450	0.0652	0.1953	0.0250	0.9750	0.0304	0.1930	0.0230	0.9770	0.0305	0.1953
	0.85	0.0580	0.9420	0.0587	0.1420	0.0590	0.9410	0.0586	0.1443	0.0270	0.9730	0.0261	0.1420	0.0240	0.9760	0.0259	0.1443
	0.9	0.0630	0.9370	0.0551	0.0946	0.0640	0.9360	0.0548	0.0970	0.0320	0.9680	0.0247	0.0946	0.0290	0.9710	0.0248	0.0970
	0.95	0.0830	0.9170	0.0509	0.0474	0.0780	0.9220	0.0515	0.0489	0.0260	0.9740	0.0198	0.0474	0.0260	0.9740	0.0203	0.0489
	0.99	0.9090	0.0910	0.0544	0.0105	0.9540	0.0460	0.0521	0.0110	0.0210	0.9790	0.0128	0.0105	0.0220	0.9780	0.0131	0.0110
**0.20**	0.1	0.0590	0.9410	0.0599	0.7986	0.0550	0.9450	0.0614	0.7595	0.0300	0.9700	0.0316	0.7986	0.0260	0.9740	0.0311	0.7595
	0.2	0.0660	0.9340	0.0643	0.7367	0.0760	0.9240	0.0649	0.7143	0.0300	0.9700	0.0316	0.7367	0.0330	0.9670	0.0330	0.7143
	0.3	0.0930	0.9070	0.0657	0.6626	0.0930	0.9070	0.0657	0.6416	0.0420	0.9580	0.0315	0.6626	0.0540	0.9460	0.0314	0.6416
	0.4	0.0680	0.9320	0.0672	0.5740	0.0640	0.9360	0.0674	0.5630	0.0270	0.9730	0.0303	0.5740	0.0270	0.9730	0.0299	0.5630
	0.5	0.0800	0.9200	0.0671	0.4790	0.0730	0.9270	0.0679	0.4725	0.0490	0.9510	0.0299	0.4790	0.0400	0.9600	0.0303	0.4725
	0.6	0.0850	0.9150	0.0657	0.3796	0.0830	0.9170	0.0647	0.3779	0.0470	0.9530	0.0308	0.3796	0.0470	0.9530	0.0305	0.3779
	0.7	0.0730	0.9270	0.0636	0.2860	0.0670	0.9330	0.0627	0.2860	0.0340	0.9660	0.0285	0.2860	0.0370	0.9630	0.0288	0.2860
	0.8	0.0840	0.9160	0.0607	0.1921	0.0830	0.9170	0.0604	0.1945	0.0410	0.9590	0.0293	0.1921	0.0390	0.9610	0.0299	0.1945
	0.85	0.0820	0.9180	0.0581	0.1420	0.0840	0.9160	0.0582	0.1442	0.0240	0.9760	0.0256	0.1420	0.0260	0.9740	0.0258	0.1442
	0.9	0.0920	0.9080	0.0553	0.0980	0.0960	0.9040	0.0555	0.1007	0.0400	0.9600	0.0247	0.0980	0.0390	0.9610	0.0249	0.1007
	0.95	0.5690	0.4310	0.0490	0.0477	0.6220	0.3780	0.0496	0.0495	0.0360	0.9640	0.0178	0.0477	0.0360	0.9640	0.0178	0.0495
	0.99	0.9570	0.0430	0.0629	0.0128	0.9910	0.0090	0.0584	0.0137	0.8510	0.1490	0.0117	0.0128	0.9100	0.0900	0.0119	0.0137

Table 1. (continued)

		**q=0.95**								**q=0.99**							
		NW				LL				NW				LL			
γ	ρ	TDR	M	S	MCE	TDR	M	S	MCE	TDR	M	S	MCE	TDR	M	S	MCE
**0.30**	0.1	0.0540	0.9460	0.0617	0.7964	0.057	0.9430	0.0643	0.7624	0.0240	0.9760	0.0319	0.7964	0.0260	0.9740	0.0322	0.7624
	0.2	0.0590	0.9410	0.0639	0.7362	0.066	0.9340	0.0651	0.7023	0.0330	0.9670	0.0314	0.7362	0.0280	0.9720	0.0330	0.7023
	0.3	0.0800	0.9200	0.0665	0.6643	0.085	0.9150	0.0643	0.6448	0.0410	0.9590	0.0314	0.6643	0.0390	0.9610	0.0310	0.6448
	0.4	0.0750	0.9250	0.0669	0.5764	0.069	0.9310	0.0674	0.5643	0.0320	0.9680	0.0292	0.5764	0.0280	0.9720	0.0292	0.5643
	0.5	0.0860	0.9140	0.0666	0.4823	0.095	0.9050	0.0654	0.4756	0.0460	0.9540	0.0302	0.4823	0.0500	0.9500	0.0292	0.4756
	0.6	0.0800	0.9200	0.0657	0.3878	0.081	0.9190	0.0648	0.3853	0.0360	0.9640	0.0297	0.3878	0.0350	0.9650	0.0283	0.3853
	0.7	0.0950	0.9050	0.0657	0.2894	0.093	0.9070	0.0644	0.2887	0.0310	0.9690	0.0284	0.2894	0.0280	0.9720	0.0289	0.2887
	0.8	0.0890	0.9110	0.0603	0.1940	0.084	0.9160	0.0602	0.1957	0.0310	0.9690	0.0285	0.1940	0.0350	0.9650	0.0282	0.1957
	0.85	0.1100	0.8900	0.0592	0.1497	0.11	0.8900	0.0589	0.1520	0.0280	0.9720	0.0262	0.1497	0.0270	0.9730	0.0264	0.1520
	0.9	0.2620	0.7380	0.0543	0.1008	0.275	0.7250	0.0543	0.1034	0.0410	0.9590	0.0249	0.1008	0.0420	0.9580	0.0250	0.1034
	0.95	0.9000	0.1000	0.0510	0.0545	0.942	0.0580	0.0512	0.0566	0.0480	0.9520	0.0192	0.0545	0.0520	0.9480	0.0195	0.0566
	0.99	0.9730	0.0270	0.0696	0.0178	0.998	0.0020	0.0622	0.0187	0.9460	0.0540	0.0122	0.0178	0.9840	0.0160	0.0120	0.0187
**0.40**	0.1	0.0870	0.9130	0.0624	0.7910	0.076	0.9240	0.0621	0.7561	0.0500	0.9500	0.0333	0.7910	0.0440	0.9560	0.0311	0.7561
	0.2	0.0850	0.9150	0.0652	0.7400	0.074	0.9260	0.0658	0.7079	0.0390	0.9610	0.0328	0.7400	0.0420	0.9580	0.0327	0.7079
	0.3	0.0930	0.9070	0.0648	0.6626	0.086	0.9140	0.0649	0.6404	0.0420	0.9580	0.0310	0.6626	0.0380	0.9620	0.0316	0.6404
	0.4	0.0890	0.9110	0.0646	0.5758	0.092	0.9080	0.0642	0.5637	0.0420	0.9580	0.0273	0.5758	0.0390	0.9610	0.0282	0.5637
	0.5	0.1150	0.8850	0.0689	0.4897	0.114	0.8860	0.0671	0.4829	0.0610	0.9390	0.0302	0.4897	0.0610	0.9390	0.0298	0.4829
	0.6	0.1000	0.9000	0.0647	0.3869	0.100	0.9000	0.0645	0.3844	0.0560	0.9440	0.0300	0.3869	0.0480	0.9520	0.0291	0.3844
	0.7	0.1210	0.8790	0.0657	0.2950	0.126	0.8740	0.0653	0.2953	0.0440	0.9560	0.0302	0.2950	0.0540	0.9460	0.0296	0.2953
	0.8	0.1610	0.8390	0.0608	0.2010	0.165	0.8350	0.0606	0.2038	0.0410	0.9590	0.0289	0.2010	0.0430	0.9570	0.0287	0.2038
	0.85	0.2820	0.7180	0.0597	0.1563	0.292	0.7080	0.0593	0.1583	0.0460	0.9540	0.0263	0.1563	0.0460	0.9540	0.0265	0.1583
	0.9	0.7570	0.2430	0.0577	0.1101	0.772	0.2280	0.0576	0.1125	0.0560	0.9440	0.0256	0.1101	0.0560	0.9440	0.0256	0.1125
	0.95	0.9580	0.0420	0.0505	0.0605	0.974	0.0260	0.0509	0.0627	0.2300	0.7700	0.0189	0.0605	0.2420	0.7580	0.0192	0.0627
	0.99	0.9750	0.0250	0.0796	0.0236	0.998	0.0020	0.0685	0.0248	0.9580	0.0420	0.0124	0.0236	0.9920	0.0080	0.0120	0.0248

Table 1. (continued)

		**q=0.95**								**q=0.99**							
		NW				LL				NW				LL			
γ	ρ	TDR	M	S	MCE	TDR	M	S	MCE	TDR	M	S	MCE	TDR	M	S	MCE
**0.50**	0.1	0.057	0.9430	0.0614	0.7880	0.072	0.9280	0.0636	0.7537	0.0250	0.9750	0.0332	0.7880	0.0380	0.9620	0.0329	0.7537
	0.2	0.081	0.9190	0.0665	0.7324	0.077	0.9230	0.0654	0.7056	0.0420	0.9580	0.0342	0.7324	0.0410	0.9590	0.0333	0.7056
	0.3	0.103	0.8970	0.0666	0.6628	0.103	0.8970	0.0654	0.6450	0.0470	0.9530	0.0315	0.6628	0.0560	0.9440	0.0311	0.6450
	0.4	0.127	0.8730	0.0659	0.5792	0.124	0.8760	0.0675	0.5682	0.0610	0.9390	0.0302	0.5792	0.0640	0.9360	0.0300	0.5682
	0.5	0.147	0.8530	0.0655	0.4855	0.157	0.8430	0.0667	0.4787	0.0650	0.9350	0.0297	0.4855	0.0630	0.9370	0.0297	0.4787
	0.6	0.163	0.8370	0.0655	0.3982	0.161	0.8390	0.0651	0.3955	0.0690	0.9310	0.0303	0.3982	0.0700	0.9300	0.0307	0.3955
	0.7	0.187	0.8130	0.0654	0.3023	0.175	0.8250	0.0642	0.3025	0.0520	0.9480	0.0294	0.3023	0.0520	0.9480	0.0293	0.3025
	0.8	0.374	0.6260	0.0626	0.2083	0.38	0.6200	0.0632	0.2100	0.0770	0.9230	0.0294	0.2083	0.0760	0.9240	0.0293	0.2100
	0.85	0.657	0.3430	0.0597	0.1623	0.694	0.3060	0.0596	0.1652	0.0780	0.9220	0.0267	0.1623	0.0770	0.9230	0.0269	0.1652
	0.9	0.899	0.1010	0.0557	0.1164	0.929	0.0710	0.0556	0.1190	0.1110	0.8890	0.0258	0.1164	0.1180	0.8820	0.0259	0.1190
	0.95	0.978	0.0220	0.0519	0.0673	0.99	0.0100	0.0517	0.0694	0.8100	0.1900	0.0188	0.0673	0.8590	0.1410	0.0192	0.0694
	0.99	0.982	0.0180	0.0836	0.0314	0.999	0.0010	0.0723	0.0327	0.9720	0.0280	0.0136	0.0314	0.9930	0.0070	0.0129	0.0327
**0.60**	0.1	0.0800	0.9200	0.0598	0.7950	0.088	0.9120	0.0638	0.7538	0.0800	0.9200	0.0598	0.7950	0.088	0.9120	0.0638	0.7538
	0.2	0.0990	0.9010	0.0658	0.7395	0.089	0.9110	0.0668	0.7114	0.0990	0.9010	0.0658	0.7395	0.089	0.9110	0.0668	0.7114
	0.3	0.1140	0.8860	0.0648	0.6687	0.122	0.8780	0.0664	0.6483	0.1140	0.8860	0.0648	0.6687	0.122	0.8780	0.0664	0.6483
	0.4	0.1520	0.8480	0.0678	0.5832	0.157	0.8430	0.0666	0.5697	0.1520	0.8480	0.0678	0.5832	0.157	0.8430	0.0666	0.5697
	0.5	0.1840	0.8160	0.0655	0.4873	0.178	0.8220	0.0665	0.4794	0.1840	0.8160	0.0655	0.4873	0.178	0.8220	0.0665	0.4794
	0.6	0.2270	0.7730	0.0648	0.3968	0.217	0.7830	0.0637	0.3951	0.2270	0.7730	0.0648	0.3968	0.217	0.7830	0.0637	0.3951
	0.7	0.3120	0.6880	0.0669	0.3078	0.319	0.6810	0.0661	0.3087	0.3120	0.6880	0.0669	0.3078	0.319	0.6810	0.0661	0.3087
	0.8	0.6350	0.3650	0.0599	0.2111	0.675	0.3250	0.0594	0.2129	0.6350	0.3650	0.0599	0.2111	0.675	0.3250	0.0594	0.2129
	0.85	0.8160	0.1840	0.0579	0.1666	0.842	0.1580	0.0575	0.1691	0.8160	0.1840	0.0579	0.1666	0.842	0.1580	0.0575	0.1691
	0.9	0.9460	0.0540	0.0547	0.1219	0.955	0.0450	0.0543	0.1241	0.9460	0.0540	0.0547	0.1219	0.955	0.0450	0.0543	0.1241
	0.95	0.9800	0.0200	0.0539	0.0767	0.989	0.0110	0.0529	0.0784	0.9800	0.0200	0.0539	0.0767	0.989	0.0110	0.0529	0.0784
	0.99	0.9820	0.0180	0.0827	0.0381	0.998	0.0020	0.0709	0.0397	0.9820	0.0180	0.0827	0.0381	0.998	0.0020	0.0709	0.0397

Table 1. (continued)

		q=0.95								q=0.99							
		NW				LL				NW				LL			
γ	ρ	TDR	M	S	MCE	TDR	M	S	MCE	TDR	M	S	MCE	TDR	M	S	MCE
**0.70**	0.1	0.0830	0.9170	0.0595	0.7953	0.088	0.9120	0.0612	0.7595	0.041	0.9590	0.0300	0.7953	0.0480	0.9520	0.0304	0.7595
	0.2	0.0980	0.9020	0.0615	0.7416	0.099	0.9010	0.0635	0.7113	0.045	0.9550	0.0305	0.7416	0.0550	0.9450	0.0321	0.7113
	0.3	0.1380	0.8620	0.0652	0.6758	0.146	0.8540	0.0637	0.6556	0.061	0.9390	0.0297	0.6758	0.0580	0.9420	0.0290	0.6556
	0.4	0.1820	0.8180	0.0638	0.5845	0.198	0.8020	0.0644	0.5690	0.079	0.9210	0.0292	0.5845	0.0860	0.9140	0.0287	0.5690
	0.5	0.2640	0.7360	0.0654	0.4958	0.274	0.7260	0.0663	0.4868	0.125	0.8750	0.0287	0.4958	0.1280	0.8720	0.0301	0.4868
	0.6	0.3430	0.6570	0.0644	0.4006	0.356	0.6440	0.0629	0.3981	0.156	0.8440	0.0289	0.4006	0.1460	0.8540	0.0289	0.3981
	0.7	0.5260	0.4740	0.0655	0.3116	0.541	0.4590	0.0643	0.3123	0.165	0.8350	0.0293	0.3116	0.1660	0.8340	0.0298	0.3123
	0.8	0.8360	0.1640	0.0630	0.2233	0.858	0.1420	0.0626	0.2249	0.316	0.6840	0.0290	0.2233	0.3200	0.6800	0.0292	0.2249
	0.85	0.9090	0.0910	0.0592	0.1749	0.926	0.0740	0.0596	0.1774	0.521	0.4790	0.0276	0.1749	0.5500	0.4500	0.0273	0.1774
	0.9	0.9550	0.0450	0.0537	0.1275	0.969	0.0310	0.0535	0.1302	0.839	0.1610	0.0251	0.1275	0.8720	0.1280	0.0254	0.1302
	0.95	0.9720	0.0280	0.0521	0.0835	0.982	0.0180	0.0517	0.0851	0.952	0.0480	0.0200	0.0835	0.9680	0.0320	0.0201	0.0851
	0.99	0.9850	0.0150	0.0789	0.0468	1	0.0000	0.0680	0.0482	0.983	0.0170	0.0138	0.0468	0.9990	0.0010	0.0135	0.0482
**0.80**	0.1	0.0740	0.9260	0.0624	0.7948	0.0780	0.9220	0.0629	0.7600	0.0450	0.9550	0.0332	0.7948	0.0380	0.9620	0.0303	0.7600
	0.2	0.1340	0.8660	0.0646	0.7436	0.1210	0.8790	0.0678	0.7108	0.0620	0.9380	0.0331	0.7436	0.0640	0.9360	0.0337	0.7108
	0.3	0.1710	0.8290	0.0628	0.6749	0.1720	0.8280	0.0614	0.6547	0.0810	0.9190	0.0290	0.6749	0.0880	0.9120	0.0290	0.6547
	0.4	0.2330	0.7670	0.0644	0.5904	0.2330	0.7670	0.0657	0.5778	0.1100	0.8900	0.0287	0.5904	0.1070	0.8930	0.0287	0.5778
	0.5	0.3420	0.6580	0.0646	0.4935	0.3380	0.6620	0.0637	0.4870	0.1630	0.8370	0.0285	0.4935	0.1470	0.8530	0.0282	0.4870
	0.6	0.4480	0.5520	0.0636	0.4086	0.4580	0.5420	0.0639	0.4055	0.2080	0.7920	0.0285	0.4086	0.2030	0.7970	0.0287	0.4055
	0.7	0.7230	0.2770	0.0638	0.3187	0.7160	0.2840	0.0632	0.3185	0.3560	0.6440	0.0275	0.3187	0.3480	0.6520	0.0277	0.3185
	0.8	0.8720	0.1280	0.0624	0.2253	0.8790	0.1210	0.0623	0.2268	0.6180	0.3820	0.0286	0.2253	0.6380	0.3620	0.0282	0.2268
	0.85	0.9190	0.0810	0.0606	0.1795	0.9280	0.0720	0.0604	0.1819	0.7710	0.2290	0.0259	0.1795	0.7860	0.2140	0.0261	0.1819
	0.9	0.9730	0.0270	0.0545	0.1321	0.9770	0.0230	0.0537	0.1343	0.9130	0.0870	0.0239	0.1321	0.9280	0.0720	0.0238	0.1343
	0.95	0.9810	0.0190	0.0514	0.0872	0.9910	0.0090	0.0514	0.0894	0.9650	0.0350	0.0192	0.0872	0.9770	0.0230	0.0192	0.0894
	0.99	0.9800	0.0200	0.0684	0.0515	0.9970	0.0030	0.0616	0.0530	0.9760	0.0240	0.0124	0.0515	0.9960	0.0040	0.0119	0.0530

Table 1. (continued)

		**q=0.95**								**q=0.99**							
		NW				LL				NW				LL			
γ	ρ	TDR	M	S	MCE	TDR	M	S	MCE	TDR	M	S	MCE	TDR	M	S	MCE
**0.85**	0.1	0.0850	0.9150	0.0625	0.7961	0.085	0.915	0.0627	0.7601	0.043	0.957	0.0315	0.7961	0.046	0.954	0.0310	0.7601
	0.2	0.1150	0.8850	0.0663	0.7387	0.109	0.891	0.0658	0.7127	0.059	0.941	0.0342	0.7387	0.055	0.945	0.0333	0.7127
	0.3	0.1970	0.8030	0.0641	0.6674	0.186	0.814	0.0644	0.6482	0.096	0.904	0.0309	0.6674	0.095	0.905	0.0306	0.6482
	0.4	0.2760	0.7240	0.0649	0.5890	0.26	0.74	0.0667	0.5747	0.139	0.861	0.0292	0.5890	0.133	0.867	0.0302	0.5747
	0.5	0.3760	0.6240	0.0651	0.4943	0.381	0.619	0.0656	0.4877	0.176	0.824	0.0284	0.4943	0.178	0.822	0.0280	0.4877
	0.6	0.5270	0.4730	0.0627	0.4025	0.538	0.462	0.0618	0.3997	0.275	0.725	0.0289	0.4025	0.28	0.72	0.0281	0.3997
	0.7	0.7280	0.2720	0.0657	0.3205	0.74	0.26	0.0656	0.3215	0.382	0.618	0.0294	0.3205	0.414	0.586	0.0290	0.3215
	0.8	0.9120	0.0880	0.0605	0.2247	0.919	0.081	0.0599	0.2263	0.72	0.28	0.0287	0.2247	0.736	0.264	0.0280	0.2263
	0.85	0.9350	0.0650	0.0576	0.1790	0.945	0.055	0.0572	0.1813	0.837	0.163	0.0257	0.1790	0.868	0.132	0.0253	0.1813
	0.9	0.9650	0.0350	0.0545	0.1353	0.97	0.03	0.0543	0.1377	0.927	0.073	0.0251	0.1353	0.939	0.061	0.0253	0.1377
	0.95	0.9830	0.0170	0.0529	0.0915	0.994	0.006	0.0528	0.0936	0.967	0.033	0.0201	0.0915	0.978	0.022	0.0202	0.0936
	0.99	0.9750	0.0250	0.0671	0.0540	0.998	0.002	0.0608	0.0557	0.97	0.03	0.0132	0.0540	0.997	0.003	0.0128	0.0557
**0.90**	0.1	0.0790	0.9210	0.0590	0.7965	0.0900	0.9100	0.0624	0.7631	0.0430	0.9570	0.0301	0.7965	0.0500	0.9500	0.0307	0.7631
	0.2	0.1130	0.8870	0.0641	0.7428	0.1130	0.8870	0.0625	0.7190	0.0630	0.9370	0.0313	0.7428	0.0550	0.9450	0.0306	0.7190
	0.3	0.1900	0.8100	0.0626	0.6707	0.1900	0.8100	0.0658	0.6516	0.0960	0.9040	0.0302	0.6707	0.0990	0.9010	0.0310	0.6516
	0.4	0.2930	0.7070	0.0650	0.5909	0.2930	0.7070	0.0636	0.5777	0.1520	0.8480	0.0277	0.5909	0.1470	0.8530	0.0288	0.5777
	0.5	0.4250	0.5750	0.0653	0.5093	0.4240	0.5760	0.0649	0.4999	0.2200	0.7800	0.0284	0.5093	0.2080	0.7920	0.0278	0.4999
	0.6	0.5760	0.4240	0.0659	0.4164	0.5840	0.4160	0.0651	0.4138	0.3180	0.6820	0.0299	0.4164	0.3210	0.6790	0.0291	0.4138
	0.7	0.7880	0.2120	0.0666	0.3228	0.7890	0.2110	0.0645	0.3230	0.5190	0.4810	0.0291	0.3228	0.5260	0.4740	0.0295	0.3230
	0.8	0.9010	0.0990	0.0609	0.2265	0.9050	0.0950	0.0605	0.2276	0.7730	0.2270	0.0290	0.2265	0.7750	0.2250	0.0290	0.2276
	0.85	0.9440	0.0560	0.0575	0.1798	0.9430	0.0570	0.0573	0.1821	0.8650	0.1350	0.0256	0.1798	0.8780	0.1220	0.0259	0.1821
	0.9	0.9590	0.0410	0.0562	0.1370	0.9680	0.0320	0.0562	0.1393	0.9270	0.0730	0.0244	0.1370	0.9420	0.0580	0.0246	0.1393
	0.95	0.9770	0.0230	0.0495	0.0909	0.9870	0.0130	0.0499	0.0931	0.9690	0.0310	0.0195	0.0909	0.9800	0.0200	0.0196	0.0931
	0.99	0.9790	0.0210	0.0588	0.0555	0.9960	0.0040	0.0559	0.0572	0.9750	0.0250	0.0121	0.0555	0.9950	0.0050	0.0118	0.0572

Table 2. Simulation results for n=40, %5 percentage of contamination

		**q=0.95**								**q=0.99**							
		NW				LL				NW				LL			
γ	ρ	TDR	M	S	MCE	TDR	M	S	MCE	TDR	M	S	MCE	TDR	M	S	MCE
	0.1	0.0650	0.9350	0.0655	0.7916	0.0740	0.9260	0.0662	0.7537	0.0375	0.9625	0.0339	0.7916	0.0345	0.9655	0.0333	0.7537
	0.2	0.0660	0.9340	0.0652	0.7407	0.0660	0.9340	0.0655	0.7129	0.0380	0.9620	0.0328	0.7407	0.0350	0.9650	0.0326	0.7129
	0.3	0.0650	0.9350	0.0667	0.6679	0.0635	0.9365	0.0667	0.6476	0.0275	0.9725	0.0320	0.6679	0.0275	0.9725	0.0313	0.6476
	0.4	0.0685	0.9315	0.0674	0.5683	0.0670	0.9330	0.0678	0.5560	0.0290	0.9710	0.0302	0.5683	0.0270	0.9730	0.0300	0.5560
**0.10**	0.5	0.0675	0.9325	0.0681	0.4808	0.0670	0.9330	0.0679	0.4736	0.0345	0.9655	0.0316	0.4808	0.0350	0.9650	0.0311	0.4736
	0.6	0.0660	0.9340	0.0665	0.3879	0.0630	0.9370	0.0657	0.3857	0.0315	0.9685	0.0310	0.3879	0.0355	0.9645	0.0302	0.3857
	0.7	0.0655	0.9345	0.0660	0.2873	0.0675	0.9325	0.0649	0.2885	0.0305	0.9695	0.0298	0.2873	0.0330	0.9670	0.0296	0.2885
	0.8	0.0665	0.9335	0.0632	0.1928	0.0680	0.9320	0.0638	0.1953	0.0350	0.9650	0.0292	0.1928	0.0330	0.9670	0.0290	0.1953
	0.85	0.0575	0.9425	0.0602	0.1413	0.0575	0.9425	0.0603	0.1447	0.0230	0.9770	0.0262	0.1413	0.0210	0.9790	0.0267	0.1447
	0.9	0.0605	0.9395	0.0528	0.0926	0.0600	0.9400	0.0526	0.0951	0.0250	0.9750	0.0241	0.0926	0.0255	0.9745	0.0242	0.0951
	0.95	0.0710	0.9290	0.0478	0.0452	0.0710	0.9290	0.0484	0.0469	0.0250	0.9750	0.0172	0.0452	0.0250	0.9750	0.0176	0.0469
	0.99	0.9155	0.0845	0.0593	0.0120	0.9495	0.0505	0.0567	0.0126	0.0205	0.9795	0.0133	0.0120	0.0205	0.9795	0.0135	0.0126
	0.1	0.0715	0.9285	0.0598	0.7939	0.0705	0.9295	0.0627	0.7588	0.0345	0.9655	0.0310	0.7939	0.0350	0.9650	0.0321	0.7588
	0.2	0.0660	0.9340	0.0658	0.7383	0.0650	0.9350	0.0656	0.7114	0.0360	0.9640	0.0342	0.7383	0.0325	0.9675	0.0335	0.7114
	0.3	0.0710	0.9290	0.0659	0.6601	0.0680	0.9320	0.0646	0.6399	0.0310	0.9690	0.0316	0.6601	0.0370	0.9630	0.0306	0.6399
	0.4	0.0685	0.9315	0.0671	0.5758	0.0700	0.9300	0.0671	0.5647	0.0295	0.9705	0.0309	0.5758	0.0300	0.9700	0.0309	0.5647
**0.20**	0.5	0.0720	0.9280	0.0662	0.4756	0.0740	0.9260	0.0651	0.4692	0.0350	0.9650	0.0290	0.4756	0.0305	0.9695	0.0294	0.4692
	0.6	0.0775	0.9225	0.0653	0.3873	0.0765	0.9235	0.0650	0.3847	0.0335	0.9665	0.0296	0.3873	0.0350	0.9650	0.0300	0.3847
	0.7	0.0630	0.9370	0.0647	0.2919	0.0625	0.9375	0.0623	0.2916	0.0275	0.9725	0.0293	0.2919	0.0285	0.9715	0.0291	0.2916
	0.8	0.0725	0.9275	0.0608	0.1941	0.0715	0.9285	0.0604	0.1961	0.0320	0.9680	0.0273	0.1941	0.0315	0.9685	0.0271	0.1961
	0.85	0.0735	0.9265	0.0582	0.1459	0.0725	0.9275	0.0580	0.1487	0.0275	0.9725	0.0258	0.1459	0.0260	0.9740	0.0262	0.1487
	0.9	0.0865	0.9135	0.0549	0.1015	0.0910	0.9090	0.0543	0.1039	0.0225	0.9775	0.0254	0.1015	0.0235	0.9765	0.0252	0.1039
	0.95	0.4850	0.5150	0.0494	0.0536	0.5565	0.4435	0.0504	0.0554	0.0250	0.9750	0.0191	0.0536	0.0255	0.9745	0.0196	0.0554
	0.99	0.9660	0.0340	0.0727	0.0159	0.9870	0.0130	0.0669	0.0168	0.8330	0.1670	0.0117	0.0159	0.8775	0.1225	0.0121	0.0168

Table 2. (continued)

		**q=0.95**								**q=0.99**							
		NW				LL				NW				LL			
γ	ρ	TDR	M	S	MCE	TDR	M	S	MCE	TDR	M	S	MCE	TDR	M	S	MCE
**0.30**	0.1	0.0645	0.9355	0.0666	0.7926	0.0695	0.9305	0.0652	0.7589	0.0350	0.9650	0.0359	0.7926	0.0370	0.9630	0.0335	0.7589
	0.2	0.0745	0.9255	0.0653	0.7376	0.0730	0.9270	0.0656	0.7127	0.0325	0.9675	0.0321	0.7376	0.0325	0.9675	0.0322	0.7127
	0.3	0.0710	0.9290	0.0662	0.6612	0.0630	0.9370	0.0658	0.6417	0.0290	0.9710	0.0322	0.6612	0.0340	0.9660	0.0321	0.6417
	0.4	0.0750	0.9250	0.0648	0.5807	0.0780	0.9220	0.0643	0.5656	0.0330	0.9670	0.0271	0.5807	0.0310	0.9690	0.0282	0.5656
	0.5	0.0840	0.9160	0.0649	0.4865	0.0860	0.9140	0.0636	0.4803	0.0425	0.9575	0.0276	0.4865	0.0385	0.9615	0.0267	0.4803
	0.6	0.0875	0.9125	0.0640	0.3907	0.0895	0.9105	0.0637	0.3880	0.0390	0.9610	0.0283	0.3907	0.0375	0.9625	0.0281	0.3880
	0.7	0.1000	0.9000	0.0637	0.2988	0.0970	0.9030	0.0641	0.2993	0.0460	0.9540	0.0286	0.2988	0.0455	0.9545	0.0282	0.2993
	0.8	0.1000	0.9000	0.0603	0.2037	0.1040	0.8960	0.0605	0.2056	0.0440	0.9560	0.0275	0.2037	0.0445	0.9555	0.0273	0.2056
	0.85	0.1070	0.8930	0.0589	0.1578	0.1070	0.8930	0.0590	0.1604	0.0285	0.9715	0.0259	0.1578	0.0290	0.9710	0.0265	0.1604
	0.9	0.2530	0.7470	0.0569	0.1127	0.2485	0.7515	0.0563	0.1153	0.0410	0.9590	0.0257	0.1127	0.0420	0.9580	0.0260	0.1153
	0.95	0.8770	0.1230	0.0500	0.0624	0.9180	0.0820	0.0509	0.0648	0.0370	0.9630	0.0192	0.0624	0.0380	0.9620	0.0199	0.0648
	0.99	0.9695	0.0305	0.0934	0.0260	0.9935	0.0065	0.0819	0.0273	0.9390	0.0610	0.0131	0.0260	0.9725	0.0275	0.0125	0.0273
**0.40**	0.1	0.0600	0.9400	0.0594	0.7898	0.0570	0.9430	0.0623	0.7569	0.0285	0.9715	0.0307	0.7898	0.0280	0.9720	0.0312	0.7569
	0.2	0.0800	0.9200	0.0638	0.7363	0.0770	0.9230	0.0657	0.7077	0.0455	0.9545	0.0316	0.7363	0.0425	0.9575	0.0324	0.7077
	0.3	0.1010	0.8990	0.0626	0.6699	0.1015	0.8985	0.0624	0.6526	0.0460	0.9540	0.0295	0.6699	0.0445	0.9555	0.0289	0.6526
	0.4	0.0885	0.9115	0.0647	0.5826	0.0930	0.9070	0.0651	0.5736	0.0400	0.9600	0.0286	0.5826	0.0435	0.9565	0.0290	0.5736
	0.5	0.0955	0.9045	0.0656	0.4953	0.0990	0.9010	0.0657	0.4882	0.0425	0.9575	0.0290	0.4953	0.0410	0.9590	0.0297	0.4882
	0.6	0.1035	0.8965	0.0646	0.4034	0.1000	0.9000	0.0633	0.4005	0.0355	0.9645	0.0289	0.4034	0.0410	0.9590	0.0282	0.4005
	0.7	0.1120	0.8880	0.0631	0.3108	0.1090	0.8910	0.0627	0.3097	0.0405	0.9595	0.0277	0.3108	0.0425	0.9575	0.0278	0.3097
	0.8	0.1465	0.8535	0.0611	0.2127	0.1485	0.8515	0.0604	0.2151	0.0475	0.9525	0.0288	0.2127	0.0490	0.9510	0.0285	0.2151
	0.85	0.2745	0.7255	0.0607	0.1701	0.2725	0.7275	0.0605	0.1732	0.0505	0.9495	0.0276	0.1701	0.0510	0.9490	0.0271	0.1732
	0.9	0.6865	0.3135	0.0538	0.1207	0.7350	0.2650	0.0537	0.1232	0.0525	0.9475	0.0234	0.1207	0.0560	0.9440	0.0234	0.1232
	0.95	0.9515	0.0485	0.0520	0.0745	0.9750	0.0250	0.0516	0.0770	0.1845	0.8155	0.0187	0.0745	0.1975	0.8025	0.0190	0.0770
	0.99	0.9845	0.0155	0.1103	0.0394	0.9970	0.0030	0.0947	0.0412	0.9640	0.0360	0.0146	0.0394	0.9910	0.0090	0.0137	0.0412

Table 2. (continued)

		**q=0.95**								**q=0.99**							
		NW				LL				NW				LL			
γ	ρ	TDR	M	S	MCE	TDR	M	S	MCE	TDR	M	S	MCE	TDR	M	S	MCE
	0.1	0.0755	0.9245	0.0628	0.7939	0.0785	0.9215	0.0647	0.7532	0.0385	0.9615	0.0320	0.7939	0.0415	0.9585	0.0325	0.7532
	0.2	0.0850	0.9150	0.0638	0.7375	0.0880	0.9120	0.0646	0.7133	0.0395	0.9605	0.0308	0.7375	0.0450	0.9550	0.0322	0.7133
	0.3	0.1000	0.9000	0.0632	0.6734	0.1095	0.8905	0.0632	0.6542	0.0525	0.9475	0.0288	0.6734	0.0510	0.9490	0.0306	0.6542
	0.4	0.1235	0.8765	0.0647	0.5807	0.1245	0.8755	0.0652	0.5709	0.0515	0.9485	0.0284	0.5807	0.0475	0.9525	0.0287	0.5709
**0.50**	0.5	0.1345	0.8655	0.0618	0.5001	0.1400	0.8600	0.0625	0.4938	0.0600	0.9400	0.0264	0.5001	0.0640	0.9360	0.0258	0.4938
	0.6	0.1370	0.8630	0.0610	0.4069	0.1375	0.8625	0.0611	0.4043	0.0540	0.9460	0.0268	0.4069	0.0530	0.9470	0.0270	0.4043
	0.7	0.1665	0.8335	0.0626	0.3148	0.1680	0.8320	0.0617	0.3143	0.0550	0.9450	0.0278	0.3148	0.0540	0.9460	0.0277	0.3143
	0.8	0.3235	0.6765	0.0600	0.2241	0.3340	0.6660	0.0602	0.2266	0.0610	0.9390	0.0277	0.2241	0.0605	0.9395	0.0282	0.2266
	0.85	0.5965	0.4035	0.0573	0.1783	0.6325	0.3675	0.0568	0.1808	0.0700	0.9300	0.0260	0.1783	0.0690	0.9310	0.0259	0.1808
	0.9	0.8855	0.1145	0.0536	0.1343	0.9125	0.0875	0.0534	0.1368	0.1270	0.8730	0.0243	0.1343	0.1180	0.8820	0.0244	0.1368
	0.95	0.9710	0.0290	0.0524	0.0895	0.9835	0.0165	0.0512	0.0916	0.7630	0.2370	0.0189	0.0895	0.8180	0.1820	0.0188	0.0916
	0.99	0.9845	0.0155	0.1129	0.0536	0.9965	0.0035	0.0953	0.0552	0.9775	0.0225	0.0149	0.0536	0.9925	0.0075	0.0133	0.0552
	0.1	0.0610	0.9390	0.0606	0.7960	0.0640	0.9360	0.0607	0.7552	0.0350	0.9650	0.0310	0.7960	0.0330	0.9670	0.0293	0.7552
	0.2	0.0870	0.9130	0.0622	0.7475	0.0865	0.9135	0.0637	0.7202	0.0410	0.9590	0.0309	0.7475	0.0450	0.9550	0.0298	0.7202
	0.3	0.1255	0.8745	0.0632	0.6737	0.1265	0.8735	0.0654	0.6565	0.0555	0.9445	0.0303	0.6737	0.0600	0.9400	0.0316	0.6565
	0.4	0.1525	0.8475	0.0617	0.5935	0.1485	0.8515	0.0620	0.5832	0.0690	0.9310	0.0258	0.5935	0.0710	0.9290	0.0262	0.5832
**0.60**	0.5	0.1600	0.8400	0.0629	0.5110	0.1640	0.8360	0.0625	0.5049	0.0670	0.9330	0.0260	0.5110	0.0635	0.9365	0.0257	0.5049
	0.6	0.2155	0.7845	0.0619	0.4178	0.2140	0.7860	0.0608	0.4145	0.0800	0.9200	0.0270	0.4178	0.0815	0.9185	0.0271	0.4145
	0.7	0.2910	0.7090	0.0655	0.3310	0.3020	0.6980	0.0651	0.3297	0.0750	0.9250	0.0294	0.3310	0.0755	0.9245	0.0294	0.3297
	0.8	0.6105	0.3895	0.0615	0.2386	0.6345	0.3655	0.0618	0.2400	0.1065	0.8935	0.0283	0.2386	0.1125	0.8875	0.0281	0.2400
	0.85	0.8090	0.1910	0.0587	0.1935	0.8410	0.1590	0.0586	0.1962	0.1475	0.8525	0.0265	0.1935	0.1395	0.8605	0.0266	0.1962
	0.9	0.9255	0.0745	0.0533	0.1471	0.9390	0.0610	0.0522	0.1488	0.4810	0.5190	0.0234	0.1471	0.5160	0.4840	0.0232	0.1488
	0.95	0.9775	0.0225	0.0539	0.1057	0.9880	0.0120	0.0517	0.1075	0.9165	0.0835	0.0194	0.1057	0.9540	0.0460	0.0194	0.1075
	0.99	0.9865	0.0135	0.1139	0.0690	0.9975	0.0025	0.0978	0.0708	0.9815	0.0185	0.0150	0.0690	0.9965	0.0035	0.0131	0.0708

Table 2. (continued)

		q=0.95								q=0.99							
		NW				LL				NW				LL			
γ	ρ	TDR	M	S	MCE	TDR	M	S	MCE	TDR	M	S	MCE	TDR	M	S	MCE
**0.70**	0.1	0.0685	0.9315	0.0614	0.7907	0.0715	0.9285	0.0614	0.7584	0.0355	0.9645	0.0314	0.7907	0.0350	0.9650	0.0296	0.7584
	0.2	0.0980	0.9020	0.0627	0.7512	0.1045	0.8955	0.0661	0.7188	0.0525	0.9475	0.0294	0.7512	0.0575	0.9425	0.0326	0.7188
	0.3	0.1375	0.8625	0.0638	0.6770	0.1335	0.8665	0.0633	0.6612	0.0680	0.9320	0.0298	0.6770	0.0605	0.9395	0.0289	0.6612
	0.4	0.1880	0.8120	0.0614	0.5985	0.1795	0.8205	0.0613	0.5869	0.0870	0.9130	0.0263	0.5985	0.0750	0.9250	0.0252	0.5869
	0.5	0.2535	0.7465	0.0633	0.5136	0.2455	0.7545	0.0626	0.5048	0.1035	0.8965	0.0253	0.5136	0.1050	0.8950	0.0251	0.5048
	0.6	0.3320	0.6680	0.0645	0.4337	0.3265	0.6735	0.0637	0.4301	0.1320	0.8680	0.0281	0.4337	0.1320	0.8680	0.0279	0.4301
	0.7	0.4900	0.5100	0.0639	0.3398	0.4990	0.5010	0.0637	0.3397	0.1625	0.8375	0.0286	0.3398	0.1640	0.8360	0.0289	0.3397
	0.8	0.7980	0.2020	0.0596	0.2488	0.8120	0.1880	0.0597	0.2492	0.2825	0.7175	0.0266	0.2488	0.2785	0.7215	0.0269	0.2492
	0.85	0.8930	0.1070	0.0582	0.2073	0.9085	0.0915	0.0576	0.2095	0.4650	0.5350	0.0258	0.2073	0.4960	0.5040	0.0261	0.2095
	0.9	0.9540	0.0460	0.0548	0.1620	0.9660	0.0340	0.0548	0.1651	0.8045	0.1955	0.0245	0.1620	0.8385	0.1615	0.0249	0.1651
	0.95	0.9795	0.0205	0.0517	0.1173	0.9890	0.0110	0.0513	0.1195	0.9520	0.0480	0.0192	0.1173	0.9660	0.0340	0.0192	0.1195
	0.99	0.9890	0.0110	0.1104	0.0828	0.9955	0.0045	0.0932	0.0846	0.9815	0.0185	0.0142	0.0828	0.9915	0.0085	0.0123	0.0846
**0.80**	0.1	0.0665	0.9335	0.0597	0.8025	0.0655	0.9345	0.0627	0.7640	0.0330	0.9670	0.0310	0.8025	0.0275	0.9725	0.0312	0.7640
	0.2	0.1170	0.8830	0.0619	0.7497	0.1055	0.8945	0.0617	0.7230	0.0635	0.9365	0.0290	0.7497	0.0530	0.9470	0.0299	0.7230
	0.3	0.1610	0.8390	0.0628	0.6833	0.1540	0.8460	0.0637	0.6625	0.0685	0.9315	0.0282	0.6833	0.0735	0.9265	0.0292	0.6625
	0.4	0.2300	0.7700	0.0607	0.6031	0.2235	0.7765	0.0624	0.5865	0.1075	0.8925	0.0257	0.6031	0.1070	0.8930	0.0260	0.5865
	0.5	0.3165	0.6835	0.0642	0.5235	0.3160	0.6840	0.0636	0.5118	0.1395	0.8605	0.0267	0.5235	0.1480	0.8520	0.0267	0.5118
	0.6	0.4385	0.5615	0.0619	0.4370	0.4460	0.5540	0.0611	0.4323	0.1990	0.8010	0.0268	0.4370	0.1950	0.8050	0.0266	0.4323
	0.7	0.6780	0.3220	0.0631	0.3454	0.6770	0.3230	0.0623	0.3446	0.3195	0.6805	0.0289	0.3454	0.3185	0.6815	0.0276	0.3446
	0.8	0.8610	0.1390	0.0613	0.2596	0.8720	0.1280	0.0610	0.2612	0.5780	0.4220	0.0284	0.2596	0.5985	0.4015	0.0286	0.2612
	0.85	0.9305	0.0695	0.0595	0.2177	0.9370	0.0630	0.0584	0.2202	0.7500	0.2500	0.0269	0.2177	0.7770	0.2230	0.0270	0.2202
	0.9	0.9635	0.0365	0.0539	0.1714	0.9720	0.0280	0.0532	0.1739	0.9005	0.0995	0.0238	0.1714	0.9190	0.0810	0.0238	0.1739
	0.95	0.9775	0.0225	0.0523	0.1297	0.9900	0.0100	0.0518	0.1325	0.9635	0.0365	0.0193	0.1297	0.9790	0.0210	0.0196	0.1325
	0.99	0.9860	0.0140	0.0897	0.0949	0.9985	0.0015	0.0787	0.0971	0.9830	0.0170	0.0133	0.0949	0.9970	0.0030	0.0121	0.0971

Table 2. (continued)

		q=0.95								q=0.99							
		NW				LL				NW				LL			
γ	ρ	TDR	M	S	MCE	TDR	M	S	MCE	TDR	M	S	MCE	TDR	M	S	MCE
**0.85**	0.1	0.0710	0.9290	0.0617	0.7992	0.0660	0.9340	0.0604	0.7635	0.0390	0.9610	0.0319	0.7992	0.0345	0.9655	0.0314	0.7635
	0.2	0.1205	0.8795	0.0618	0.7562	0.1130	0.8870	0.0614	0.7247	0.0550	0.9450	0.0293	0.7562	0.0540	0.9460	0.0295	0.7247
	0.3	0.1765	0.8235	0.0604	0.6816	0.1645	0.8355	0.0612	0.6599	0.0815	0.9185	0.0287	0.6816	0.0860	0.9140	0.0282	0.6599
	0.4	0.2675	0.7325	0.0638	0.6069	0.2735	0.7265	0.0635	0.5952	0.1245	0.8755	0.0262	0.6069	0.1190	0.8810	0.0267	0.5952
	0.5	0.3720	0.6280	0.0645	0.5212	0.3600	0.6400	0.0642	0.5136	0.1635	0.8365	0.0282	0.5212	0.1715	0.8285	0.0270	0.5136
	0.6	0.5005	0.4995	0.0619	0.4381	0.5015	0.4985	0.0615	0.4344	0.2420	0.7580	0.0276	0.4381	0.2475	0.7525	0.0288	0.4344
	0.7	0.7225	0.2775	0.0616	0.3464	0.7290	0.2710	0.0604	0.3454	0.3835	0.6165	0.0284	0.3464	0.3975	0.6025	0.0282	0.3454
	0.8	0.8970	0.1030	0.0593	0.2615	0.9060	0.0940	0.0590	0.2630	0.6915	0.3085	0.0271	0.2615	0.7005	0.2995	0.0271	0.2630
	0.85	0.9400	0.0600	0.0555	0.2180	0.9425	0.0575	0.0554	0.2197	0.8180	0.1820	0.0255	0.2180	0.8455	0.1545	0.0257	0.2197
	0.9	0.9760	0.0240	0.0554	0.1800	0.9800	0.0200	0.0550	0.1821	0.9360	0.0640	0.0248	0.1800	0.9430	0.0570	0.0250	0.1821
	0.95	0.9770	0.0230	0.0524	0.1338	0.9870	0.0130	0.0516	0.1362	0.9625	0.0375	0.0197	0.1338	0.9750	0.0250	0.0199	0.1362
	0.99	0.9845	0.0155	0.0832	0.0995	0.9965	0.0035	0.0718	0.1017	0.9785	0.0215	0.0145	0.0995	0.9950	0.0050	0.0137	0.1017
**0.90**	0.1	0.0710	0.9290	0.0604	0.7968	0.0690	0.9310	0.0630	0.7629	0.0375	0.9625	0.0299	0.7968	0.0370	0.9630	0.0306	0.7629
	0.2	0.1295	0.8705	0.0639	0.7414	0.1250	0.8750	0.0654	0.7135	0.0680	0.9320	0.0307	0.7414	0.0680	0.9320	0.0323	0.7135
	0.3	0.1895	0.8105	0.0601	0.6901	0.1810	0.8190	0.0604	0.6673	0.0875	0.9125	0.0274	0.6901	0.0870	0.9130	0.0278	0.6673
	0.4	0.2680	0.7320	0.0633	0.6061	0.2640	0.7360	0.0636	0.5920	0.1175	0.8825	0.0273	0.6061	0.1305	0.8695	0.0264	0.5920
	0.5	0.4140	0.5860	0.0616	0.5200	0.4220	0.5780	0.0627	0.5135	0.1995	0.8005	0.0252	0.5200	0.2050	0.7950	0.0256	0.5135
	0.6	0.5795	0.4205	0.0625	0.4378	0.5780	0.4220	0.0622	0.4334	0.3125	0.6875	0.0270	0.4378	0.3135	0.6865	0.0274	0.4334
	0.7	0.7690	0.2310	0.0660	0.3578	0.7680	0.2320	0.0650	0.3557	0.4970	0.5030	0.0313	0.3578	0.5175	0.4825	0.0306	0.3557
	0.8	0.8945	0.1055	0.0623	0.2678	0.8995	0.1005	0.0626	0.2700	0.7375	0.2625	0.0291	0.2678	0.7570	0.2430	0.0294	0.2700
	0.85	0.9420	0.0580	0.0576	0.2230	0.9485	0.0515	0.0576	0.2255	0.8515	0.1485	0.0260	0.2230	0.8645	0.1355	0.0264	0.2255
	0.9	0.9630	0.0370	0.0551	0.1801	0.9725	0.0275	0.0551	0.1830	0.9225	0.0775	0.0249	0.1801	0.9350	0.0650	0.0246	0.1830
	0.95	0.9795	0.0205	0.0494	0.1359	0.9915	0.0085	0.0491	0.1385	0.9675	0.0325	0.0184	0.1359	0.9785	0.0215	0.0189	0.1385
	0.99	0.9805	0.0195	0.0702	0.1024	0.9930	0.0070	0.0623	0.1045	0.9760	0.0240	0.0125	0.1024	0.9915	0.0085	0.0121	0.1045

Table 3. Simulation results for n=40, %10 percentage of contamination

		q=0.95								q=0.99							
		NW				LL				NW				LL			
γ	ρ	TDR	M	S	MCE	TDR	M	S	MCE	TDR	M	S	MCE	TDR	M	S	MCE
**0.10**	0.1	0.0628	0.9373	0.0638	0.7893	0.0648	0.9353	0.0637	0.7611	0.0323	0.9678	0.0333	0.7893	0.0323	0.9678	0.0312	0.7611
	0.2	0.0608	0.9393	0.0637	0.7400	0.0610	0.9390	0.0659	0.7132	0.0258	0.9743	0.0311	0.7400	0.0280	0.9720	0.0322	0.7132
	0.3	0.0710	0.9290	0.0674	0.6600	0.0690	0.9310	0.0663	0.6426	0.0345	0.9655	0.0322	0.6600	0.0343	0.9658	0.0309	0.6426
	0.4	0.0703	0.9298	0.0690	0.5699	0.0670	0.9330	0.0680	0.5611	0.0295	0.9705	0.0314	0.5699	0.0313	0.9688	0.0312	0.5611
	0.5	0.0680	0.9320	0.0696	0.4786	0.0703	0.9298	0.0698	0.4722	0.0330	0.9670	0.0329	0.4786	0.0295	0.9705	0.0330	0.4722
	0.6	0.0618	0.9383	0.0657	0.3831	0.0605	0.9395	0.0648	0.3800	0.0285	0.9715	0.0294	0.3831	0.0288	0.9713	0.0305	0.3800
	0.7	0.0678	0.9323	0.0658	0.2898	0.0650	0.9350	0.0654	0.2894	0.0288	0.9713	0.0286	0.2898	0.0300	0.9700	0.0288	0.2894
	0.8	0.0613	0.9388	0.0600	0.1905	0.0635	0.9365	0.0607	0.1926	0.0280	0.9720	0.0289	0.1905	0.0285	0.9715	0.0287	0.1926
	0.85	0.0658	0.9343	0.0581	0.1418	0.0633	0.9368	0.0579	0.1451	0.0275	0.9725	0.0252	0.1418	0.0275	0.9725	0.0256	0.1451
	0.9	0.0590	0.9410	0.0539	0.0955	0.0598	0.9403	0.0532	0.0974	0.0235	0.9765	0.0238	0.0955	0.0243	0.9758	0.0236	0.0974
	0.95	0.0695	0.9305	0.0499	0.0499	0.0725	0.9275	0.0509	0.0519	0.0230	0.9770	0.0192	0.0499	0.0240	0.9760	0.0199	0.0519
	0.99	0.8838	0.1163	0.0643	0.0136	0.9378	0.0623	0.0591	0.0143	0.0235	0.9765	0.0135	0.0136	0.0233	0.9768	0.0134	0.0143
**0.20**	0.1	0.0598	0.9403	0.0586	0.7939	0.0595	0.9405	0.0619	0.7603	0.0308	0.9693	0.0297	0.7939	0.0273	0.9728	0.0312	0.7603
	0.2	0.0683	0.9318	0.0652	0.7344	0.0653	0.9348	0.0661	0.7085	0.0340	0.9660	0.0334	0.7344	0.0330	0.9670	0.0334	0.7085
	0.3	0.0733	0.9268	0.0650	0.6650	0.0710	0.9290	0.0651	0.6476	0.0345	0.9655	0.0304	0.6650	0.0368	0.9633	0.0311	0.6476
	0.4	0.0735	0.9265	0.0666	0.5713	0.0713	0.9288	0.0674	0.5593	0.0308	0.9693	0.0302	0.5713	0.0320	0.9680	0.0289	0.5593
	0.5	0.0778	0.9223	0.0670	0.4837	0.0788	0.9213	0.0656	0.4752	0.0325	0.9675	0.0294	0.4837	0.0368	0.9633	0.0299	0.4752
	0.6	0.0713	0.9288	0.0630	0.3939	0.0753	0.9248	0.0635	0.3923	0.0325	0.9675	0.0283	0.3939	0.0363	0.9638	0.0281	0.3923
	0.7	0.0738	0.9263	0.0625	0.2968	0.0738	0.9263	0.0618	0.2970	0.0325	0.9675	0.0288	0.2968	0.0313	0.9688	0.0281	0.2970
	0.8	0.0715	0.9285	0.0610	0.2014	0.0720	0.9280	0.0608	0.2033	0.0343	0.9658	0.0284	0.2014	0.0340	0.9660	0.0287	0.2033
	0.85	0.0708	0.9293	0.0578	0.1540	0.0710	0.9290	0.0568	0.1565	0.0295	0.9705	0.0258	0.1540	0.0320	0.9680	0.0257	0.1565
	0.9	0.0728	0.9273	0.0519	0.1057	0.0755	0.9245	0.0523	0.1085	0.0285	0.9715	0.0246	0.1057	0.0283	0.9718	0.0248	0.1085
	0.95	0.3610	0.6390	0.0513	0.0628	0.4118	0.5883	0.0512	0.0645	0.0200	0.9800	0.0203	0.0628	0.0200	0.9800	0.0204	0.0645
	0.99	0.9653	0.0348	0.0986	0.0245	0.9863	0.0138	0.0859	0.0257	0.7108	0.2893	0.0128	0.0245	0.7883	0.2118	0.0123	0.0257

Table 3. (continued)

		q=0.95								q=0.99							
		NW				LL				NW				LL			
γ	ρ	TDR	M	S	MCE	TDR	M	S	MCE	TDR	M	S	MCE	TDR	M	S	MCE
**0.30**	0.1	0.0645	0.9355	0.0631	0.7935	0.0668	0.9333	0.0624	0.7633	0.0335	0.9665	0.0331	0.7935	0.0340	0.9660	0.0311	0.7633
	0.2	0.0743	0.9258	0.0656	0.7379	0.0743	0.9258	0.0653	0.7126	0.0363	0.9638	0.0343	0.7379	0.0353	0.9648	0.0337	0.7126
	0.3	0.0705	0.9295	0.0632	0.6700	0.0680	0.9320	0.0629	0.6498	0.0280	0.9720	0.0283	0.6700	0.0298	0.9703	0.0284	0.6498
	0.4	0.0880	0.9120	0.0652	0.5820	0.0865	0.9135	0.0649	0.5695	0.0420	0.9580	0.0284	0.5820	0.0415	0.9585	0.0284	0.5695
	0.5	0.0830	0.9170	0.0643	0.4960	0.0818	0.9183	0.0637	0.4903	0.0343	0.9658	0.0268	0.4960	0.0350	0.9650	0.0269	0.4903
	0.6	0.0755	0.9245	0.0619	0.4026	0.0760	0.9240	0.0599	0.3995	0.0355	0.9645	0.0279	0.4026	0.0348	0.9653	0.0266	0.3995
	0.7	0.0833	0.9168	0.0616	0.3102	0.0828	0.9173	0.0605	0.3103	0.0333	0.9668	0.0277	0.3102	0.0330	0.9670	0.0277	0.3103
	0.8	0.0865	0.9135	0.0613	0.2207	0.0885	0.9115	0.0608	0.2226	0.0338	0.9663	0.0276	0.2207	0.0335	0.9665	0.0268	0.2226
	0.85	0.0948	0.9053	0.0556	0.1679	0.0975	0.9025	0.0560	0.1700	0.0310	0.9690	0.0246	0.1679	0.0305	0.9695	0.0244	0.1700
	0.9	0.1765	0.8235	0.0548	0.1266	0.1808	0.8193	0.0544	0.1296	0.0333	0.9668	0.0245	0.1266	0.0350	0.9650	0.0246	0.1296
	0.95	0.8228	0.1773	0.0515	0.0786	0.8693	0.1308	0.0499	0.0806	0.0345	0.9655	0.0188	0.0786	0.0350	0.9650	0.0190	0.0806
	0.99	0.9773	0.0228	0.1470	0.0433	0.9915	0.0085	0.1092	0.0451	0.9388	0.0613	0.0143	0.0433	0.9665	0.0335	0.0124	0.0451
**0.40**	0.1	0.0660	0.9340	0.0627	0.7950	0.0658	0.9343	0.0624	0.7608	0.0373	0.9628	0.0326	0.7950	0.0343	0.9658	0.0310	0.7608
	0.2	0.0733	0.9268	0.0628	0.7470	0.0738	0.9263	0.0650	0.7248	0.0360	0.9640	0.0303	0.7470	0.0378	0.9623	0.0314	0.7248
	0.3	0.0723	0.9278	0.0616	0.6817	0.0790	0.9210	0.0630	0.6604	0.0340	0.9660	0.0282	0.6817	0.0350	0.9650	0.0290	0.6604
	0.4	0.0923	0.9078	0.0627	0.5970	0.0948	0.9053	0.0627	0.5849	0.0400	0.9600	0.0267	0.5970	0.0380	0.9620	0.0276	0.5849
	0.5	0.0923	0.9078	0.0634	0.5067	0.0958	0.9043	0.0643	0.4983	0.0373	0.9628	0.0267	0.5067	0.0393	0.9608	0.0265	0.4983
	0.6	0.0935	0.9065	0.0601	0.4200	0.0993	0.9008	0.0604	0.4156	0.0433	0.9568	0.0254	0.4200	0.0415	0.9585	0.0254	0.4156
	0.7	0.1025	0.8975	0.0616	0.3278	0.1045	0.8955	0.0607	0.3278	0.0398	0.9603	0.0272	0.3278	0.0393	0.9608	0.0268	0.3278
	0.8	0.1273	0.8728	0.0579	0.2356	0.1270	0.8730	0.0578	0.2379	0.0408	0.9593	0.0268	0.2356	0.0423	0.9578	0.0267	0.2379
	0.85	0.2075	0.7925	0.0557	0.1943	0.2033	0.7968	0.0554	0.1962	0.0463	0.9538	0.0254	0.1943	0.0488	0.9513	0.0248	0.1962
	0.9	0.5765	0.4235	0.0531	0.1482	0.6098	0.3903	0.0523	0.1506	0.0490	0.9510	0.0235	0.1482	0.0508	0.9493	0.0232	0.1506
	0.95	0.9438	0.0563	0.0543	0.1062	0.9645	0.0355	0.0520	0.1081	0.0988	0.9013	0.0205	0.1062	0.0960	0.9040	0.0205	0.1081
	0.99	0.9858	0.0143	0.1987	0.0666	0.9953	0.0048	0.1275	0.0687	0.9710	0.0290	0.0160	0.0666	0.9888	0.0113	0.0123	0.0687

Table 3. (continued)

		q=0.95								q=0.99							
		NW				LL				NW				LL			
γ	ρ	TDR	M	S	MCE	TDR	M	S	MCE	TDR	M	S	MCE	TDR	M	S	MCE
**0.50**	0.1	0.0650	0.9350	0.0597	0.8028	0.0693	0.9308	0.0593	0.7666	0.0288	0.9713	0.0308	0.8028	0.0310	0.9690	0.0280	0.7666
	0.2	0.0768	0.9233	0.0609	0.7582	0.0815	0.9185	0.0614	0.7280	0.0388	0.9613	0.0283	0.7582	0.0425	0.9575	0.0307	0.7280
	0.3	0.0860	0.9140	0.0567	0.6824	0.0880	0.9120	0.0573	0.6590	0.0383	0.9618	0.0269	0.6824	0.0393	0.9608	0.0265	0.6590
	0.4	0.1043	0.8958	0.0615	0.6011	0.1025	0.8975	0.0631	0.5882	0.0450	0.9550	0.0251	0.6011	0.0438	0.9563	0.0266	0.5882
	0.5	0.1165	0.8835	0.0619	0.5193	0.1185	0.8815	0.0611	0.5120	0.0480	0.9520	0.0253	0.5193	0.0473	0.9528	0.0247	0.5120
	0.6	0.1223	0.8778	0.0592	0.4337	0.1240	0.8760	0.0595	0.4316	0.0490	0.9510	0.0239	0.4337	0.0530	0.9470	0.0241	0.4316
	0.7	0.1495	0.8505	0.0595	0.3475	0.1463	0.8538	0.0581	0.3456	0.0550	0.9450	0.0248	0.3475	0.0510	0.9490	0.0250	0.3456
	0.8	0.2705	0.7295	0.0584	0.2615	0.2778	0.7223	0.0579	0.2631	0.0620	0.9380	0.0266	0.2615	0.0633	0.9368	0.0266	0.2631
	0.85	0.4943	0.5058	0.0548	0.2164	0.5215	0.4785	0.0543	0.2177	0.0595	0.9405	0.0239	0.2164	0.0598	0.9403	0.0241	0.2177
	0.9	0.8463	0.1538	0.0520	0.1743	0.8755	0.1245	0.0509	0.1767	0.0905	0.9095	0.0231	0.1743	0.0893	0.9108	0.0231	0.1767
	0.95	0.9663	0.0338	0.0520	0.1302	0.9770	0.0230	0.0495	0.1329	0.6153	0.3848	0.0190	0.1302	0.6770	0.3230	0.0189	0.1329
	0.99	0.9908	0.0093	0.2355	0.0962	0.9963	0.0038	0.1553	0.0986	0.9785	0.0215	0.0199	0.0962	0.9898	0.0103	0.0140	0.0986
**0.60**	0.1	0.0710	0.9290	0.0630	0.7964	0.0678	0.9323	0.0627	0.7628	0.0380	0.9620	0.0328	0.7964	0.0360	0.9640	0.0317	0.7628
	0.2	0.0815	0.9185	0.0608	0.7545	0.0860	0.9140	0.0625	0.7208	0.0373	0.9628	0.0288	0.7545	0.0400	0.9600	0.0323	0.7208
	0.3	0.1050	0.8950	0.0600	0.6884	0.1040	0.8960	0.0601	0.6646	0.0443	0.9558	0.0260	0.6884	0.0480	0.9520	0.0273	0.6646
	0.4	0.1205	0.8795	0.0575	0.6159	0.1265	0.8735	0.0573	0.6003	0.0538	0.9463	0.0231	0.6159	0.0555	0.9445	0.0224	0.6003
	0.5	0.1615	0.8385	0.0580	0.5372	0.1600	0.8400	0.0583	0.5268	0.0618	0.9383	0.0227	0.5372	0.0593	0.9408	0.0214	0.5268
	0.6	0.1805	0.8195	0.0575	0.4514	0.1805	0.8195	0.0571	0.4464	0.0723	0.9278	0.0239	0.4514	0.0753	0.9248	0.0234	0.4464
	0.7	0.2605	0.7395	0.0609	0.3704	0.2530	0.7470	0.0597	0.3682	0.0785	0.9215	0.0272	0.3704	0.0743	0.9258	0.0269	0.3682
	0.8	0.5303	0.4698	0.0587	0.2842	0.5588	0.4413	0.0586	0.2846	0.0923	0.9078	0.0266	0.2842	0.0933	0.9068	0.0260	0.2846
	0.85	0.7543	0.2458	0.0567	0.2454	0.7848	0.2153	0.0560	0.2465	0.1148	0.8853	0.0239	0.2454	0.1118	0.8883	0.0238	0.2465
	0.9	0.9278	0.0723	0.0538	0.2049	0.9400	0.0600	0.0533	0.2069	0.3340	0.6660	0.0236	0.2049	0.3433	0.6568	0.0241	0.2069
	0.95	0.9763	0.0238	0.0525	0.1595	0.9790	0.0210	0.0487	0.1612	0.8785	0.1215	0.0186	0.1595	0.9085	0.0915	0.0181	0.1612
	0.99	0.9930	0.0070	0.2383	0.1282	0.9963	0.0038	0.1497	0.1298	0.9865	0.0135	0.0210	0.1282	0.9938	0.0063	0.0148	0.1298

Table 3. (continued)

		q=0.95								q=0.99							
		NW				LL				NW				LL			
γ	ρ	TDR	M	S	MCE	TDR	M	S	MCE	TDR	M	S	MCE	TDR	M	S	MCE
	0.1	0.0695	0.9305	0.0588	0.8045	0.0705	0.9295	0.0621	0.7662	0.0340	0.9660	0.0296	0.8045	0.0358	0.9643	0.0302	0.7662
	0.2	0.0890	0.9110	0.0584	0.7588	0.0898	0.9103	0.0585	0.7296	0.0435	0.9565	0.0266	0.7588	0.0460	0.9540	0.0273	0.7296
	0.3	0.1265	0.8735	0.0535	0.6973	0.1240	0.8760	0.0552	0.6748	0.0603	0.9398	0.0231	0.6973	0.0598	0.9403	0.0236	0.6748
	0.4	0.1575	0.8425	0.0554	0.6229	0.1600	0.8400	0.0570	0.6074	0.0718	0.9283	0.0226	0.6229	0.0688	0.9313	0.0227	0.6074
**0.70**	0.5	0.2070	0.7930	0.0573	0.5504	0.2065	0.7935	0.0567	0.5413	0.0850	0.9150	0.0221	0.5504	0.0808	0.9193	0.0219	0.5413
	0.6	0.2718	0.7283	0.0595	0.4726	0.2755	0.7245	0.0578	0.4671	0.1108	0.8893	0.0236	0.4726	0.1080	0.8920	0.0237	0.4671
	0.7	0.4433	0.5568	0.0586	0.3857	0.4515	0.5485	0.0576	0.3839	0.1308	0.8693	0.0252	0.3857	0.1258	0.8743	0.0249	0.3839
	0.8	0.7418	0.2583	0.0589	0.3068	0.7608	0.2393	0.0583	0.3074	0.2220	0.7780	0.0271	0.3068	0.2213	0.7788	0.0272	0.3074
	0.85	0.8763	0.1238	0.0561	0.2677	0.8868	0.1133	0.0556	0.2692	0.3433	0.6568	0.0252	0.2677	0.3478	0.6523	0.0244	0.2692
	0.9	0.9393	0.0608	0.0528	0.2272	0.9535	0.0465	0.0521	0.2303	0.7458	0.2543	0.0238	0.2272	0.7780	0.2220	0.0235	0.2303
	0.95	0.9778	0.0223	0.0542	0.1889	0.9863	0.0138	0.0510	0.1915	0.9440	0.0560	0.0189	0.1889	0.9570	0.0430	0.0191	0.1915
	0.99	0.9915	0.0085	0.2103	0.1560	0.9955	0.0045	0.1414	0.1581	0.9855	0.0145	0.0201	0.1560	0.9933	0.0068	0.0144	0.1581
	0.1	0.0710	0.9290	0.0589	0.8028	0.0780	0.9220	0.0589	0.7679	0.0313	0.9688	0.0289	0.8028	0.0355	0.9645	0.0293	0.7679
	0.2	0.1040	0.8960	0.0546	0.7627	0.1073	0.8928	0.0596	0.7284	0.0513	0.9488	0.0256	0.7627	0.0475	0.9525	0.0276	0.7284
	0.3	0.1448	0.8553	0.0577	0.7013	0.1443	0.8558	0.0576	0.6804	0.0723	0.9278	0.0263	0.7013	0.0700	0.9300	0.0256	0.6804
	0.4	0.2058	0.7943	0.0567	0.6357	0.2010	0.7990	0.0570	0.6197	0.0933	0.9068	0.0218	0.6357	0.0833	0.9168	0.0212	0.6197
**0.80**	0.5	0.2800	0.7200	0.0591	0.5622	0.2730	0.7270	0.0574	0.5512	0.1098	0.8903	0.0216	0.5622	0.1075	0.8925	0.0222	0.5512
	0.6	0.4190	0.5810	0.0589	0.4838	0.4258	0.5743	0.0585	0.4794	0.1730	0.8270	0.0241	0.4838	0.1795	0.8205	0.0238	0.4794
	0.7	0.6100	0.3900	0.0608	0.4124	0.6338	0.3663	0.0603	0.4105	0.2570	0.7430	0.0255	0.4124	0.2640	0.7360	0.0260	0.4105
	0.8	0.8443	0.1558	0.0586	0.3297	0.8543	0.1458	0.0582	0.3302	0.4995	0.5005	0.0285	0.3297	0.5150	0.4850	0.0275	0.3302
	0.85	0.9245	0.0755	0.0590	0.2946	0.9308	0.0693	0.0573	0.2955	0.6965	0.3035	0.0263	0.2946	0.7190	0.2810	0.0260	0.2955
	0.9	0.9590	0.0410	0.0539	0.2506	0.9653	0.0348	0.0524	0.2531	0.8848	0.1153	0.0237	0.2506	0.9045	0.0955	0.0235	0.2531
	0.95	0.9788	0.0213	0.0524	0.2119	0.9843	0.0158	0.0513	0.2147	0.9620	0.0380	0.0191	0.2119	0.9728	0.0273	0.0195	0.2147
	0.99	0.9885	0.0115	0.1622	0.1800	0.9960	0.0040	0.1203	0.1827	0.9823	0.0178	0.0168	0.1800	0.9925	0.0075	0.0144	0.1827

Table 3. (continued)

		q=0.95								q=0.99							
		NW				LL				NW				LL			
γ	ρ	TDR	M	S	MCE	TDR	M	S	MCE	TDR	M	S	MCE	TDR	M	S	MCE
**0.85**	0.1	0.0718	0.9283	0.0584	0.8016	0.0735	0.9265	0.0629	0.7653	0.0355	0.9645	0.0298	0.8016	0.0413	0.9588	0.0322	0.7653
	0.2	0.1033	0.8968	0.0561	0.7560	0.1043	0.8958	0.0581	0.7305	0.0505	0.9495	0.0256	0.7560	0.0475	0.9525	0.0265	0.7305
	0.3	0.1513	0.8488	0.0549	0.7041	0.1468	0.8533	0.0559	0.6788	0.0698	0.9303	0.0240	0.7041	0.0680	0.9320	0.0249	0.6788
	0.4	0.2153	0.7848	0.0565	0.6396	0.2098	0.7903	0.0581	0.6221	0.0868	0.9133	0.0227	0.6396	0.0925	0.9075	0.0230	0.6221
	0.5	0.3215	0.6785	0.0567	0.5676	0.3238	0.6763	0.0567	0.5561	0.1378	0.8623	0.0224	0.5676	0.1435	0.8565	0.0218	0.5561
	0.6	0.4773	0.5228	0.0584	0.4957	0.4745	0.5255	0.0568	0.4897	0.2028	0.7973	0.0243	0.4957	0.2020	0.7980	0.0239	0.4897
	0.7	0.6850	0.3150	0.0596	0.4125	0.6905	0.3095	0.0578	0.4098	0.3465	0.6535	0.0262	0.4125	0.3585	0.6415	0.0259	0.4098
	0.8	0.8745	0.1255	0.0579	0.3352	0.8813	0.1188	0.0566	0.3361	0.6428	0.3573	0.0260	0.3352	0.6580	0.3420	0.0263	0.3361
	0.85	0.9328	0.0673	0.0555	0.2976	0.9405	0.0595	0.0548	0.2997	0.7953	0.2048	0.0238	0.2976	0.8143	0.1858	0.0242	0.2997
	0.9	0.9658	0.0343	0.0533	0.2602	0.9695	0.0305	0.0530	0.2624	0.9195	0.0805	0.0236	0.2602	0.9255	0.0745	0.0233	0.2624
	0.95	0.9813	0.0188	0.0548	0.2230	0.9873	0.0128	0.0529	0.2255	0.9665	0.0335	0.0203	0.2230	0.9758	0.0243	0.0202	0.2255
	0.99	0.9913	0.0088	0.1360	0.1909	0.9948	0.0053	0.1058	0.1932	0.9863	0.0138	0.0175	0.1909	0.9918	0.0083	0.0146	0.1932
**0.90**	0.1	0.0698	0.9303	0.0608	0.8027	0.0715	0.9285	0.0596	0.7614	0.0320	0.9680	0.0299	0.8027	0.0348	0.9653	0.0298	0.7614
	0.2	0.1075	0.8925	0.0558	0.7726	0.1068	0.8933	0.0599	0.7325	0.0558	0.9443	0.0256	0.7726	0.0528	0.9473	0.0290	0.7325
	0.3	0.1480	0.8520	0.0525	0.7124	0.1485	0.8515	0.0550	0.6833	0.0653	0.9348	0.0213	0.7124	0.0673	0.9328	0.0233	0.6833
	0.4	0.2305	0.7695	0.0551	0.6459	0.2293	0.7708	0.0556	0.6275	0.0998	0.9003	0.0214	0.6459	0.0933	0.9068	0.0218	0.6275
	0.5	0.3473	0.6528	0.0580	0.5780	0.3435	0.6565	0.0565	0.5631	0.1483	0.8518	0.0225	0.5780	0.1513	0.8488	0.0214	0.5631
	0.6	0.5360	0.4640	0.0606	0.4944	0.5295	0.4705	0.0592	0.4889	0.2630	0.7370	0.0252	0.4944	0.2613	0.7388	0.0247	0.4889
	0.7	0.7358	0.2643	0.0599	0.4185	0.7323	0.2678	0.0585	0.4155	0.4488	0.5513	0.0253	0.4185	0.4560	0.5440	0.0246	0.4155
	0.8	0.8958	0.1043	0.0583	0.3434	0.8955	0.1045	0.0574	0.3425	0.7273	0.2728	0.0274	0.3434	0.7345	0.2655	0.0269	0.3425
	0.85	0.9375	0.0625	0.0567	0.3044	0.9398	0.0603	0.0560	0.3055	0.8425	0.1575	0.0254	0.3044	0.8498	0.1503	0.0253	0.3055
	0.9	0.9643	0.0358	0.0545	0.2672	0.9700	0.0300	0.0538	0.2698	0.9270	0.0730	0.0250	0.2672	0.9353	0.0648	0.0250	0.2698
	0.95	0.9828	0.0173	0.0502	0.2271	0.9873	0.0128	0.0499	0.2292	0.9698	0.0303	0.0188	0.2271	0.9773	0.0228	0.0184	0.2292
	0.99	0.9833	0.0168	0.1032	0.1964	0.9943	0.0058	0.0860	0.2000	0.9773	0.0228	0.0158	0.1964	0.9920	0.0080	0.0143	0.2000
