# Supplementary material for: A method for detecting outliers in linear-circular non-parametric regression
Source: PLoS One. 2023 Jun 12;18(6):e0286448. doi: 10.1371/journal.pone.0286448 (PMC10259788; doi:10.1371/journal.pone.0286448)
Supplement: S3 File — (PDF) [file pone.0286448.s003.pdf]

A Method for Detecting Outliers in Linear-Circular Non-Parametric Regression

Sümeyra Sert[1]* *and* Filiz Kardiyen[2]

[1] *Selcuk University, Department of Statistics, 42250, Selcuklu, Konya, Turkey;*

[1]ORCID id: https://orcid.org/0000-0002-4647-1583

sumeyra.sert@selcuk.edu.tr

[2]*Gazi University, Department of Statistics, Teknikokullar, 06500, Ankara, Turkey.*

[2]ORCID id: https://orcid.org/0000-0002-8730-2751

fyuva@gazi.edu.tr

Supplementary File (n=50)

Table 1. Simulation results for n=50 , %1 percentage of contamination

		q=0.95								q=0.99							
		NW				LL				NW				LL			
γ	ρ	TDR	M	S	MCE	TDR	M	S	MCE	TDR	M	S	MCE	TDR	M	S	MCE
	0.1	0.0570	0.9430	0.0614	0.8093	0.0590	0.9410	0.0616	0.7823	0.0250	0.9750	0.0310	0.8093	0.0280	0.9720	0.0292	0.7823
	0.2	0.0610	0.9390	0.0609	0.7471	0.0540	0.9460	0.0634	0.7253	0.0270	0.9730	0.0288	0.7471	0.0230	0.9770	0.0299	0.7253
	0.3	0.0560	0.9440	0.0618	0.6703	0.0550	0.9450	0.0614	0.6550	0.0250	0.9750	0.0273	0.6703	0.0270	0.9730	0.0266	0.6550
	0.4	0.0670	0.9330	0.0637	0.5786	0.0660	0.9340	0.0638	0.5717	0.0290	0.9710	0.0273	0.5786	0.0240	0.9760	0.0268	0.5717
**0.10**	0.5	0.0630	0.9370	0.0644	0.4807	0.0620	0.9380	0.0644	0.4780	0.0240	0.9760	0.0280	0.4807	0.0300	0.9700	0.0276	0.4780
	0.6	0.0730	0.9270	0.0628	0.3777	0.0730	0.9270	0.0625	0.3778	0.0350	0.9650	0.0280	0.3777	0.0300	0.9700	0.0279	0.3778
	0.7	0.0630	0.9370	0.0619	0.2846	0.0670	0.9330	0.0613	0.2859	0.0390	0.9610	0.0269	0.2846	0.0370	0.9630	0.0266	0.2859
	0.8	0.0570	0.9430	0.0576	0.1905	0.0560	0.9440	0.0581	0.1932	0.0290	0.9710	0.0246	0.1905	0.0290	0.9710	0.0243	0.1932
	0.85	0.0600	0.9400	0.0564	0.1415	0.0590	0.9410	0.0569	0.1445	0.0260	0.9740	0.0240	0.1415	0.0260	0.9740	0.0239	0.1445
	0.9	0.0600	0.9400	0.0539	0.0944	0.0590	0.9410	0.0546	0.0970	0.0200	0.9800	0.0229	0.0944	0.0220	0.9780	0.0230	0.0970
	0.95	0.0710	0.9290	0.0511	0.0484	0.0820	0.9180	0.0514	0.0500	0.0180	0.9820	0.0193	0.0484	0.0200	0.9800	0.0196	0.0500
	0.99	0.9320	0.0680	0.0498	0.0094	0.9620	0.0380	0.0494	0.0099	0.0130	0.9870	0.0104	0.0094	0.0160	0.9840	0.0106	0.0099
	0.1	0.0550	0.9450	0.0616	0.8049	0.0600	0.9400	0.0633	0.7805	0.0280	0.9720	0.0315	0.8049	0.0320	0.9680	0.0313	0.7805
	0.2	0.0550	0.9450	0.0595	0.7499	0.0560	0.9440	0.0635	0.7241	0.0270	0.9730	0.0288	0.7499	0.0270	0.9730	0.0300	0.7241
	0.3	0.0630	0.9370	0.0634	0.6713	0.0640	0.9360	0.0636	0.6587	0.0200	0.9800	0.0276	0.6713	0.0200	0.9800	0.0266	0.6587
	0.4	0.0740	0.9260	0.0645	0.5770	0.0820	0.9180	0.0652	0.5685	0.0350	0.9650	0.0283	0.5770	0.0360	0.9640	0.0272	0.5685
**0.20**	0.5	0.0550	0.9450	0.0638	0.4802	0.0550	0.9450	0.0639	0.4779	0.0140	0.9860	0.0269	0.4802	0.0170	0.9830	0.0281	0.4779
	0.6	0.0640	0.9360	0.0628	0.3834	0.0650	0.9350	0.0628	0.3833	0.0220	0.9780	0.0268	0.3834	0.0230	0.9770	0.0265	0.3833
	0.7	0.0790	0.9210	0.0623	0.2896	0.0810	0.9190	0.0623	0.2909	0.0390	0.9610	0.0279	0.2896	0.0400	0.9600	0.0279	0.2909
	0.8	0.0640	0.9360	0.0580	0.1928	0.0660	0.9340	0.0578	0.1954	0.0260	0.9740	0.0249	0.1928	0.0260	0.9740	0.0247	0.1954
	0.85	0.0750	0.9250	0.0570	0.1438	0.0770	0.9230	0.0569	0.1462	0.0350	0.9650	0.0232	0.1438	0.0340	0.9660	0.0232	0.1462
	0.9	0.0840	0.9160	0.0544	0.0981	0.0810	0.9190	0.0552	0.1006	0.0240	0.9760	0.0230	0.0981	0.0250	0.9750	0.0235	0.1006
	0.95	0.5060	0.4940	0.0488	0.0480	0.5970	0.4030	0.0488	0.0495	0.0220	0.9780	0.0176	0.0480	0.0240	0.9760	0.0177	0.0495
	0.99	0.9710	0.0290	0.0540	0.0119	0.9930	0.0070	0.0523	0.0124	0.8260	0.1740	0.0106	0.0119	0.8830	0.1170	0.0108	0.0124

Table 1. (continued)

		**q=0.95**								**q=0.99**							
		NW				LL				NW				LL			
γ	ρ	TDR	M	S	MCE	TDR	M	S	MCE	TDR	M	S	MCE	TDR	M	S	MCE
**0.30**	0.1	0.053	0.9470	0.0576	0.8080	0.059	0.9410	0.0609	0.7798	0.0320	0.9680	0.0297	0.8080	0.0260	0.9740	0.0290	0.7798
	0.2	0.063	0.9370	0.0601	0.7471	0.064	0.9360	0.0626	0.7278	0.0310	0.9690	0.0293	0.7471	0.0280	0.9720	0.0290	0.7278
	0.3	0.083	0.9170	0.0623	0.6660	0.086	0.9140	0.0608	0.6514	0.0450	0.9550	0.0272	0.6660	0.0490	0.9510	0.0273	0.6514
	0.4	0.078	0.9220	0.0619	0.5746	0.085	0.9150	0.0613	0.5659	0.0370	0.9630	0.0260	0.5746	0.0370	0.9630	0.0274	0.5659
	0.5	0.075	0.9250	0.0652	0.4877	0.072	0.9280	0.0652	0.4822	0.0320	0.9680	0.0272	0.4877	0.0360	0.9640	0.0273	0.4822
	0.6	0.068	0.9320	0.0645	0.3850	0.076	0.9240	0.0654	0.3854	0.0250	0.9750	0.0274	0.3850	0.0270	0.9730	0.0271	0.3854
	0.7	0.094	0.9060	0.0629	0.2940	0.095	0.9050	0.0627	0.2956	0.0470	0.9530	0.0275	0.2940	0.0460	0.9540	0.0274	0.2956
	0.8	0.104	0.8960	0.0583	0.1964	0.106	0.8940	0.0581	0.1994	0.0390	0.9610	0.0247	0.1964	0.0370	0.9630	0.0246	0.1994
	0.85	0.109	0.8910	0.0581	0.1493	0.116	0.8840	0.0576	0.1517	0.0350	0.9650	0.0232	0.1493	0.0380	0.9620	0.0235	0.1517
	0.9	0.245	0.7550	0.0525	0.0995	0.265	0.7350	0.0525	0.1019	0.0260	0.9740	0.0224	0.0995	0.0310	0.9690	0.0228	0.1019
	0.95	0.904	0.0960	0.0503	0.0538	0.936	0.0640	0.0509	0.0556	0.0380	0.9620	0.0185	0.0538	0.0360	0.9640	0.0188	0.0556
	0.99	0.97	0.0300	0.0650	0.0177	0.993	0.0070	0.0588	0.0185	0.9490	0.0510	0.0124	0.0177	0.9780	0.0220	0.0123	0.0185
**0.40**	0.1	0.059	0.9410	0.0609	0.8135	0.065	0.9350	0.0604	0.7830	0.0280	0.9720	0.0298	0.8135	0.0340	0.9660	0.0287	0.7830
	0.2	0.062	0.9380	0.0604	0.7512	0.075	0.9250	0.0626	0.7273	0.0320	0.9680	0.0286	0.7512	0.0330	0.9670	0.0283	0.7273
	0.3	0.077	0.9230	0.0635	0.6749	0.071	0.9290	0.0629	0.6617	0.0330	0.9670	0.0280	0.6749	0.0310	0.9690	0.0272	0.6617
	0.4	0.087	0.9130	0.0633	0.5758	0.088	0.9120	0.0643	0.5695	0.0340	0.9660	0.0278	0.5758	0.0380	0.9620	0.0262	0.5695
	0.5	0.095	0.9050	0.0625	0.4851	0.096	0.9040	0.0613	0.4816	0.0430	0.9570	0.0253	0.4851	0.0410	0.9590	0.0255	0.4816
	0.6	0.114	0.8860	0.0613	0.3916	0.114	0.8860	0.0615	0.3906	0.0430	0.9570	0.0258	0.3916	0.0510	0.9490	0.0255	0.3906
	0.7	0.094	0.9060	0.0619	0.2977	0.099	0.9010	0.0619	0.2996	0.0340	0.9660	0.0266	0.2977	0.0390	0.9610	0.0269	0.2996
	0.8	0.129	0.8710	0.0568	0.1987	0.134	0.8660	0.0568	0.2012	0.0370	0.9630	0.0227	0.1987	0.0400	0.9600	0.0230	0.2012
	0.85	0.254	0.7460	0.0557	0.1501	0.257	0.7430	0.0558	0.1527	0.0330	0.9670	0.0233	0.1501	0.0330	0.9670	0.0233	0.1527
	0.9	0.71	0.2900	0.0547	0.1069	0.753	0.2470	0.0553	0.1096	0.0460	0.9540	0.0225	0.1069	0.0500	0.9500	0.0227	0.1096
	0.95	0.964	0.0360	0.0503	0.0578	0.974	0.0260	0.0503	0.0595	0.1270	0.8730	0.0183	0.0578	0.1440	0.8560	0.0184	0.0595
	0.99	0.981	0.0190	0.0682	0.0210	0.996	0.0040	0.0589	0.0218	0.9750	0.0250	0.0113	0.0210	0.9930	0.0070	0.0111	0.0218

Table 1. (continued)

		**q=0.95**								**q=0.99**							
		NW				LL				NW				LL			
γ	ρ	TDR	M	S	MCE	TDR	M	S	MCE	TDR	M	S	MCE	TDR	M	S	MCE
**0.50**	0.1	0.078	0.9220	0.0592	0.8112	0.081	0.9190	0.0618	0.7775	0.0370	0.9630	0.0288	0.8112	0.0410	0.9590	0.0294	0.7775
	0.2	0.076	0.9240	0.0620	0.7477	0.084	0.9160	0.0624	0.7237	0.0370	0.9630	0.0303	0.7477	0.0340	0.9660	0.0293	0.7237
	0.3	0.097	0.9030	0.0622	0.6743	0.091	0.9090	0.0627	0.6597	0.0410	0.9590	0.0272	0.6743	0.0420	0.9580	0.0268	0.6597
	0.4	0.112	0.8880	0.0639	0.5764	0.113	0.8870	0.0629	0.5674	0.0430	0.9570	0.0277	0.5764	0.0480	0.9520	0.0267	0.5674
	0.5	0.13	0.8700	0.0641	0.4968	0.128	0.8720	0.0643	0.4921	0.0490	0.9510	0.0255	0.4968	0.0490	0.9510	0.0265	0.4921
	0.6	0.164	0.8360	0.0643	0.3948	0.151	0.8490	0.0640	0.3935	0.0630	0.9370	0.0265	0.3948	0.0700	0.9300	0.0263	0.3935
	0.7	0.207	0.7930	0.0620	0.3016	0.208	0.7920	0.0618	0.3038	0.0590	0.9410	0.0255	0.3016	0.0610	0.9390	0.0261	0.3038
	0.8	0.306	0.6940	0.0558	0.2014	0.314	0.6860	0.0555	0.2038	0.0540	0.9460	0.0239	0.2014	0.0540	0.9460	0.0238	0.2038
	0.85	0.641	0.3590	0.0575	0.1596	0.651	0.3490	0.0571	0.1618	0.0620	0.9380	0.0237	0.1596	0.0690	0.9310	0.0236	0.1618
	0.9	0.899	0.1010	0.0532	0.1094	0.915	0.0850	0.0528	0.1115	0.1060	0.8940	0.0216	0.1094	0.1050	0.8950	0.0221	0.1115
	0.95	0.964	0.0360	0.0502	0.0634	0.987	0.0130	0.0501	0.0653	0.7760	0.2240	0.0179	0.0634	0.8110	0.1890	0.0180	0.0653
	0.99	0.985	0.0150	0.0693	0.0276	1	0.0000	0.0612	0.0287	0.9770	0.0230	0.0118	0.0276	0.9980	0.0020	0.0116	0.0287
**0.60**	0.1	0.0730	0.9270	0.0603	0.8087	0.079	0.9210	0.0637	0.7799	0.0460	0.9540	0.0307	0.8087	0.0420	0.9580	0.0313	0.7799
	0.2	0.1080	0.8920	0.0576	0.7556	0.097	0.9030	0.0583	0.7314	0.0460	0.9540	0.0264	0.7556	0.0430	0.9570	0.0259	0.7314
	0.3	0.1110	0.8890	0.0637	0.6702	0.11	0.8900	0.0638	0.6557	0.0520	0.9480	0.0282	0.6702	0.0490	0.9510	0.0280	0.6557
	0.4	0.1530	0.8470	0.0622	0.5855	0.157	0.8430	0.0616	0.5761	0.0730	0.9270	0.0253	0.5855	0.0570	0.9430	0.0252	0.5761
	0.5	0.1570	0.8430	0.0611	0.4892	0.16	0.8400	0.0623	0.4868	0.0690	0.9310	0.0259	0.4892	0.0650	0.9350	0.0258	0.4868
	0.6	0.1960	0.8040	0.0612	0.4011	0.198	0.8020	0.0606	0.3990	0.0680	0.9320	0.0256	0.4011	0.0650	0.9350	0.0251	0.3990
	0.7	0.3160	0.6840	0.0626	0.3062	0.313	0.6870	0.0625	0.3073	0.0960	0.9040	0.0267	0.3062	0.1020	0.8980	0.0273	0.3073
	0.8	0.6130	0.3870	0.0581	0.2105	0.645	0.3550	0.0579	0.2130	0.1060	0.8940	0.0244	0.2105	0.1100	0.8900	0.0245	0.2130
	0.85	0.8480	0.1520	0.0567	0.1632	0.864	0.1360	0.0570	0.1658	0.1230	0.8770	0.0243	0.1632	0.1210	0.8790	0.0243	0.1658
	0.9	0.9450	0.0550	0.0543	0.1163	0.958	0.0420	0.0539	0.1189	0.4050	0.5950	0.0228	0.1163	0.4310	0.5690	0.0229	0.1189
	0.95	0.9770	0.0230	0.0508	0.0710	0.99	0.0100	0.0512	0.0729	0.9270	0.0730	0.0190	0.0710	0.9460	0.0540	0.0192	0.0729
	0.99	0.9870	0.0130	0.0709	0.0346	1	0.0000	0.0634	0.0357	0.9740	0.0260	0.0128	0.0346	0.9970	0.0030	0.0124	0.0357

Table 1. (continued)

		q=0.95								q=0.99							
		NW				LL				NW				LL			
γ	ρ	TDR	M	S	MCE	TDR	M	S	MCE	TDR	M	S	MCE	TDR	M	S	MCE
**0.70**	0.1	0.0740	0.9260	0.0603	0.8157	0.064	0.9360	0.0614	0.7811	0.036	0.9640	0.0287	0.8157	0.0310	0.9690	0.0294	0.7811
	0.2	0.0870	0.9130	0.0588	0.7542	0.101	0.8990	0.0603	0.7350	0.045	0.9550	0.0279	0.7542	0.0400	0.9600	0.0266	0.7350
	0.3	0.1430	0.8570	0.0606	0.6760	0.153	0.8470	0.0599	0.6576	0.069	0.9310	0.0276	0.6760	0.0750	0.9250	0.0262	0.6576
	0.4	0.1970	0.8030	0.0608	0.5887	0.187	0.8130	0.0593	0.5796	0.074	0.9260	0.0252	0.5887	0.0880	0.9120	0.0241	0.5796
	0.5	0.2490	0.7510	0.0625	0.4961	0.253	0.7470	0.0642	0.4910	0.102	0.8980	0.0269	0.4961	0.1030	0.8970	0.0268	0.4910
	0.6	0.3230	0.6770	0.0619	0.4034	0.334	0.6660	0.0615	0.4017	0.129	0.8710	0.0254	0.4034	0.1200	0.8800	0.0259	0.4017
	0.7	0.5070	0.4930	0.0613	0.3078	0.531	0.4690	0.0618	0.3099	0.155	0.8450	0.0263	0.3078	0.1650	0.8350	0.0268	0.3099
	0.8	0.7940	0.2060	0.0559	0.2128	0.805	0.1950	0.0560	0.2156	0.228	0.7720	0.0251	0.2128	0.2240	0.7760	0.0250	0.2156
	0.85	0.9070	0.0930	0.0569	0.1691	0.923	0.0770	0.0569	0.1718	0.406	0.5940	0.0235	0.1691	0.4180	0.5820	0.0234	0.1718
	0.9	0.9630	0.0370	0.0531	0.1201	0.971	0.0290	0.0534	0.1228	0.785	0.2150	0.0216	0.1201	0.8300	0.1700	0.0218	0.1228
	0.95	0.9800	0.0200	0.0496	0.0754	0.99	0.0100	0.0492	0.0774	0.962	0.0380	0.0187	0.0754	0.9800	0.0200	0.0189	0.0774
	0.99	0.9850	0.0150	0.0660	0.0393	0.999	0.0010	0.0590	0.0404	0.98	0.0200	0.0119	0.0393	0.9970	0.0030	0.0117	0.0404
**0.80**	0.1	0.0700	0.9300	0.0597	0.8106	0.0680	0.9320	0.0622	0.7772	0.0390	0.9610	0.0302	0.8106	0.0410	0.9590	0.0296	0.7772
	0.2	0.1030	0.8970	0.0599	0.7550	0.1080	0.8920	0.0635	0.7275	0.0480	0.9520	0.0287	0.7550	0.0430	0.9570	0.0299	0.7275
	0.3	0.1590	0.8410	0.0631	0.6796	0.1530	0.8470	0.0610	0.6634	0.0670	0.9330	0.0276	0.6796	0.0770	0.9230	0.0260	0.6634
	0.4	0.2380	0.7620	0.0624	0.5887	0.2290	0.7710	0.0624	0.5816	0.0960	0.9040	0.0265	0.5887	0.0870	0.9130	0.0260	0.5816
	0.5	0.3420	0.6580	0.0628	0.4983	0.3570	0.6430	0.0626	0.4937	0.1540	0.8460	0.0268	0.4983	0.1660	0.8340	0.0270	0.4937
	0.6	0.4260	0.5740	0.0612	0.4057	0.4330	0.5670	0.0611	0.4036	0.1810	0.8190	0.0252	0.4057	0.1770	0.8230	0.0252	0.4036
	0.7	0.6850	0.3150	0.0620	0.3121	0.7010	0.2990	0.0620	0.3133	0.3070	0.6930	0.0259	0.3121	0.3170	0.6830	0.0262	0.3133
	0.8	0.8830	0.1170	0.0564	0.2160	0.8940	0.1060	0.0568	0.2192	0.5170	0.4830	0.0251	0.2160	0.5430	0.4570	0.0249	0.2192
	0.85	0.9370	0.0630	0.0577	0.1742	0.9440	0.0560	0.0574	0.1766	0.7440	0.2560	0.0245	0.1742	0.7670	0.2330	0.0248	0.1766
	0.9	0.9610	0.0390	0.0529	0.1267	0.9600	0.0400	0.0539	0.1289	0.8980	0.1020	0.0236	0.1267	0.9130	0.0870	0.0237	0.1289
	0.95	0.9830	0.0170	0.0504	0.0795	0.9930	0.0070	0.0505	0.0816	0.9640	0.0360	0.0181	0.0795	0.9800	0.0200	0.0183	0.0816
	0.99	0.9850	0.0150	0.0626	0.0441	0.9990	0.0010	0.0580	0.0453	0.9780	0.0220	0.0119	0.0441	0.9970	0.0030	0.0118	0.0453

Table 1. (continued)

		**q=0.95**								**q=0.99**							
		NW				LL				NW				LL			
γ	ρ	TDR	M	S	MCE	TDR	M	S	MCE	TDR	M	S	MCE	TDR	M	S	MCE
**0.85**	0.1	0.0680	0.9320	0.0593	0.8152	0.071	0.929	0.0633	0.7826	0.031	0.969	0.0283	0.8152	0.033	0.967	0.0304	0.7826
	0.2	0.1020	0.8980	0.0579	0.7520	0.104	0.896	0.0622	0.7265	0.054	0.946	0.0263	0.7520	0.041	0.959	0.0283	0.7265
	0.3	0.1520	0.8480	0.0621	0.6736	0.149	0.851	0.0620	0.6589	0.074	0.926	0.0271	0.6736	0.07	0.93	0.0263	0.6589
	0.4	0.2550	0.7450	0.0594	0.5976	0.249	0.751	0.0602	0.5874	0.112	0.888	0.0247	0.5976	0.095	0.905	0.0238	0.5874
	0.5	0.3650	0.6350	0.0615	0.4989	0.384	0.616	0.0607	0.4955	0.174	0.826	0.0253	0.4989	0.18	0.82	0.0255	0.4955
	0.6	0.5550	0.4450	0.0625	0.4023	0.561	0.439	0.0628	0.4017	0.262	0.738	0.0267	0.4023	0.247	0.753	0.0254	0.4017
	0.7	0.7120	0.2880	0.0617	0.3142	0.724	0.276	0.0608	0.3153	0.384	0.616	0.0275	0.3142	0.397	0.603	0.0270	0.3153
	0.8	0.8760	0.1240	0.0583	0.2191	0.886	0.114	0.0577	0.2222	0.602	0.398	0.0235	0.2191	0.622	0.378	0.0234	0.2222
	0.85	0.9380	0.0620	0.0578	0.1744	0.946	0.054	0.0571	0.1769	0.803	0.197	0.0242	0.1744	0.819	0.181	0.0241	0.1769
	0.9	0.9620	0.0380	0.0532	0.1268	0.968	0.032	0.0538	0.1294	0.921	0.079	0.0228	0.1268	0.934	0.066	0.0228	0.1294
	0.95	0.9830	0.0170	0.0478	0.0802	0.991	0.009	0.0482	0.0818	0.972	0.028	0.0178	0.0802	0.982	0.018	0.0176	0.0818
	0.99	0.9850	0.0150	0.0577	0.0455	0.998	0.002	0.0531	0.0467	0.98	0.02	0.0115	0.0455	0.998	0.002	0.0113	0.0467
**0.90**	0.1	0.1010	0.8990	0.0581	0.8132	0.0890	0.9110	0.0618	0.7811	0.0480	0.9520	0.0297	0.8132	0.0430	0.9570	0.0303	0.7811
	0.2	0.1210	0.8790	0.0608	0.7504	0.1290	0.8710	0.0620	0.7318	0.0610	0.9390	0.0289	0.7504	0.0660	0.9340	0.0287	0.7318
	0.3	0.1570	0.8430	0.0598	0.6819	0.1700	0.8300	0.0589	0.6648	0.0720	0.9280	0.0253	0.6819	0.0780	0.9220	0.0252	0.6648
	0.4	0.2520	0.7480	0.0599	0.5918	0.2550	0.7450	0.0591	0.5826	0.1140	0.8860	0.0252	0.5918	0.1100	0.8900	0.0240	0.5826
	0.5	0.3940	0.6060	0.0621	0.5004	0.3960	0.6040	0.0622	0.4953	0.1840	0.8160	0.0253	0.5004	0.1830	0.8170	0.0252	0.4953
	0.6	0.5380	0.4620	0.0597	0.4021	0.5510	0.4490	0.0599	0.4009	0.2750	0.7250	0.0258	0.4021	0.2610	0.7390	0.0254	0.4009
	0.7	0.7970	0.2030	0.0621	0.3120	0.8090	0.1910	0.0621	0.3137	0.5080	0.4920	0.0273	0.3120	0.5230	0.4770	0.0271	0.3137
	0.8	0.8840	0.1160	0.0580	0.2231	0.8870	0.1130	0.0573	0.2254	0.7090	0.2910	0.0248	0.2231	0.7150	0.2850	0.0247	0.2254
	0.85	0.9310	0.0690	0.0561	0.1739	0.9380	0.0620	0.0559	0.1769	0.8440	0.1560	0.0232	0.1739	0.8580	0.1420	0.0234	0.1769
	0.9	0.9590	0.0410	0.0537	0.1273	0.9680	0.0320	0.0538	0.1297	0.9100	0.0900	0.0215	0.1273	0.9280	0.0720	0.0215	0.1297
	0.95	0.9870	0.0130	0.0508	0.0838	0.9870	0.0130	0.0512	0.0855	0.9720	0.0280	0.0190	0.0838	0.9760	0.0240	0.0190	0.0855
	0.99	0.9830	0.0170	0.0559	0.0465	0.9970	0.0030	0.0522	0.0476	0.9760	0.0240	0.0104	0.0465	0.9960	0.0040	0.0106	0.0476

Table 2. Simulation results for n=50, %5 percentage of contamination

		**q=0.95**								**q=0.99**							
		NW				LL				NW				LL			
γ	ρ	TDR	M	S	MCE	TDR	M	S	MCE	TDR	M	S	MCE	TDR	M	S	MCE
**0.10**	0.1	0.0590	0.9410	0.0589	0.8146	0.0650	0.9350	0.0613	0.7856	0.0293	0.9707	0.0283	0.8146	0.0333	0.9667	0.0299	0.7856
	0.2	0.0553	0.9447	0.0598	0.7546	0.0607	0.9393	0.0613	0.7312	0.0283	0.9717	0.0280	0.7546	0.0303	0.9697	0.0279	0.7312
	0.3	0.0607	0.9393	0.0626	0.6662	0.0597	0.9403	0.0632	0.6492	0.0280	0.9720	0.0277	0.6662	0.0270	0.9730	0.0286	0.6492
	0.4	0.0600	0.9400	0.0628	0.5802	0.0600	0.9400	0.0614	0.5723	0.0277	0.9723	0.0261	0.5802	0.0247	0.9753	0.0258	0.5723
	0.5	0.0647	0.9353	0.0632	0.4839	0.0680	0.9320	0.0640	0.4813	0.0273	0.9727	0.0266	0.4839	0.0270	0.9730	0.0265	0.4813
	0.6	0.0633	0.9367	0.0638	0.3844	0.0607	0.9393	0.0644	0.3839	0.0277	0.9723	0.0274	0.3844	0.0270	0.9730	0.0273	0.3839
	0.7	0.0647	0.9353	0.0620	0.2893	0.0617	0.9383	0.0619	0.2906	0.0290	0.9710	0.0267	0.2893	0.0297	0.9703	0.0274	0.2906
	0.8	0.0567	0.9433	0.0581	0.1930	0.0560	0.9440	0.0581	0.1951	0.0257	0.9743	0.0250	0.1930	0.0247	0.9753	0.0248	0.1951
	0.85	0.0587	0.9413	0.0568	0.1440	0.0597	0.9403	0.0568	0.1467	0.0240	0.9760	0.0234	0.1440	0.0240	0.9760	0.0235	0.1467
	0.9	0.0573	0.9427	0.0530	0.0959	0.0590	0.9410	0.0534	0.0983	0.0227	0.9773	0.0217	0.0959	0.0243	0.9757	0.0219	0.0983
	0.95	0.0687	0.9313	0.0496	0.0501	0.0720	0.9280	0.0502	0.0515	0.0180	0.9820	0.0189	0.0501	0.0190	0.9810	0.0192	0.0515
	0.99	0.9213	0.0787	0.0516	0.0111	0.9560	0.0440	0.0497	0.0116	0.0157	0.9843	0.0114	0.0111	0.0170	0.9830	0.0114	0.0116
**0.20**	0.1	0.0590	0.9410	0.0616	0.8099	0.0670	0.9330	0.0622	0.7802	0.0253	0.9747	0.0312	0.8099	0.0310	0.9690	0.0301	0.7802
	0.2	0.0720	0.9280	0.0590	0.7539	0.0687	0.9313	0.0616	0.7315	0.0340	0.9660	0.0279	0.7539	0.0303	0.9697	0.0277	0.7315
	0.3	0.0713	0.9287	0.0635	0.6646	0.0743	0.9257	0.0624	0.6488	0.0340	0.9660	0.0286	0.6646	0.0353	0.9647	0.0281	0.6488
	0.4	0.0700	0.9300	0.0640	0.5766	0.0673	0.9327	0.0636	0.5698	0.0273	0.9727	0.0283	0.5766	0.0290	0.9710	0.0279	0.5698
	0.5	0.0690	0.9310	0.0618	0.4873	0.0700	0.9300	0.0626	0.4828	0.0283	0.9717	0.0264	0.4873	0.0303	0.9697	0.0260	0.4828
	0.6	0.0613	0.9387	0.0595	0.3863	0.0610	0.9390	0.0609	0.3849	0.0240	0.9760	0.0264	0.3863	0.0250	0.9750	0.0265	0.3849
	0.7	0.0683	0.9317	0.0616	0.2909	0.0690	0.9310	0.0609	0.2923	0.0300	0.9700	0.0272	0.2909	0.0287	0.9713	0.0267	0.2923
	0.8	0.0617	0.9383	0.0565	0.1976	0.0607	0.9393	0.0562	0.1995	0.0270	0.9730	0.0234	0.1976	0.0280	0.9720	0.0233	0.1995
	0.85	0.0740	0.9260	0.0577	0.1539	0.0730	0.9270	0.0580	0.1566	0.0317	0.9683	0.0247	0.1539	0.0310	0.9690	0.0247	0.1566
	0.9	0.0777	0.9223	0.0524	0.1014	0.0790	0.9210	0.0529	0.1040	0.0247	0.9753	0.0217	0.1014	0.0243	0.9757	0.0221	0.1040
	0.95	0.4263	0.5737	0.0494	0.0563	0.5020	0.4980	0.0499	0.0580	0.0200	0.9800	0.0191	0.0563	0.0200	0.9800	0.0191	0.0580
	0.99	0.9733	0.0267	0.0718	0.0186	0.9920	0.0080	0.0649	0.0196	0.7153	0.2847	0.0112	0.0186	0.7963	0.2037	0.0111	0.0196

Table 2. (continued)

		q=0.95								q=0.99							
		NW				LL				NW				LL			
γ	ρ	TDR	M	S	MCE	TDR	M	S	MCE	TDR	M	S	MCE	TDR	M	S	MCE
**0.30**	0.1	0.0577	0.9423	0.0596	0.8112	0.0657	0.9343	0.0627	0.7748	0.0283	0.9717	0.0294	0.8112	0.0310	0.9690	0.0315	0.7748
	0.2	0.0723	0.9277	0.0594	0.7521	0.0723	0.9277	0.0624	0.7311	0.0347	0.9653	0.0282	0.7521	0.0323	0.9677	0.0288	0.7311
	0.3	0.0727	0.9273	0.0594	0.6741	0.0707	0.9293	0.0611	0.6566	0.0303	0.9697	0.0263	0.6741	0.0307	0.9693	0.0269	0.6566
	0.4	0.0700	0.9300	0.0606	0.5829	0.0753	0.9247	0.0596	0.5730	0.0283	0.9717	0.0246	0.5829	0.0313	0.9687	0.0246	0.5730
	0.5	0.0817	0.9183	0.0623	0.4933	0.0813	0.9187	0.0627	0.4875	0.0330	0.9670	0.0245	0.4933	0.0343	0.9657	0.0251	0.4875
	0.6	0.0793	0.9207	0.0607	0.3993	0.0790	0.9210	0.0615	0.3986	0.0287	0.9713	0.0259	0.3993	0.0267	0.9733	0.0251	0.3986
	0.7	0.0860	0.9140	0.0607	0.3024	0.0860	0.9140	0.0606	0.3040	0.0327	0.9673	0.0259	0.3024	0.0317	0.9683	0.0254	0.3040
	0.8	0.0847	0.9153	0.0554	0.2058	0.0837	0.9163	0.0553	0.2085	0.0270	0.9730	0.0238	0.2058	0.0277	0.9723	0.0239	0.2085
	0.85	0.0963	0.9037	0.0565	0.1623	0.0983	0.9017	0.0563	0.1655	0.0313	0.9687	0.0232	0.1623	0.0330	0.9670	0.0235	0.1655
	0.9	0.2020	0.7980	0.0499	0.1108	0.2100	0.7900	0.0510	0.1138	0.0293	0.9707	0.0206	0.1108	0.0313	0.9687	0.0211	0.1138
	0.95	0.8777	0.1223	0.0498	0.0666	0.9143	0.0857	0.0500	0.0688	0.0370	0.9630	0.0180	0.0666	0.0413	0.9587	0.0185	0.0688
	0.99	0.9797	0.0203	0.0922	0.0302	0.9940	0.0060	0.0799	0.0317	0.9470	0.0530	0.0114	0.0302	0.9707	0.0293	0.0113	0.0317
**0.40**	0.1	0.0597	0.9403	0.0594	0.8102	0.0633	0.9367	0.0633	0.7810	0.0310	0.9690	0.0292	0.8102	0.0317	0.9683	0.0307	0.7810
	0.2	0.0617	0.9383	0.0594	0.7586	0.0647	0.9353	0.0598	0.7362	0.0327	0.9673	0.0269	0.7586	0.0297	0.9703	0.0268	0.7362
	0.3	0.0787	0.9213	0.0611	0.6704	0.0810	0.9190	0.0608	0.6569	0.0347	0.9653	0.0262	0.6704	0.0323	0.9677	0.0264	0.6569
	0.4	0.0937	0.9063	0.0626	0.5924	0.0947	0.9053	0.0607	0.5835	0.0413	0.9587	0.0258	0.5924	0.0413	0.9587	0.0240	0.5835
	0.5	0.0853	0.9147	0.0613	0.4982	0.0880	0.9120	0.0604	0.4949	0.0400	0.9600	0.0252	0.4982	0.0437	0.9563	0.0252	0.4949
	0.6	0.0933	0.9067	0.0617	0.4069	0.0947	0.9053	0.0611	0.4058	0.0387	0.9613	0.0264	0.4069	0.0387	0.9613	0.0261	0.4058
	0.7	0.1157	0.8843	0.0599	0.3132	0.1167	0.8833	0.0592	0.3136	0.0477	0.9523	0.0261	0.3132	0.0473	0.9527	0.0257	0.3136
	0.8	0.1263	0.8737	0.0569	0.2219	0.1253	0.8747	0.0570	0.2246	0.0443	0.9557	0.0232	0.2219	0.0433	0.9567	0.0233	0.2246
	0.85	0.2047	0.7953	0.0549	0.1721	0.2050	0.7950	0.0550	0.1752	0.0393	0.9607	0.0223	0.1721	0.0417	0.9583	0.0225	0.1752
	0.9	0.6220	0.3780	0.0535	0.1283	0.6763	0.3237	0.0542	0.1309	0.0423	0.9577	0.0215	0.1283	0.0450	0.9550	0.0218	0.1309
	0.95	0.9517	0.0483	0.0490	0.0817	0.9713	0.0287	0.0489	0.0839	0.0973	0.9027	0.0186	0.0817	0.0977	0.9023	0.0187	0.0839
	0.99	0.9887	0.0113	0.1094	0.0461	0.9963	0.0037	0.0905	0.0476	0.9727	0.0273	0.0125	0.0461	0.9880	0.0120	0.0123	0.0476

Table 2. (continued)

		**q=0.95**								**q=0.99**							
		NW				LL				NW				LL			
γ	ρ	TDR	M	S	MCE	TDR	M	S	MCE	TDR	M	S	MCE	TDR	M	S	MCE
**0.50**	0.1	0.0690	0.9310	0.0582	0.8130	0.0643	0.9357	0.0597	0.7782	0.0327	0.9673	0.0284	0.8130	0.0300	0.9700	0.0289	0.7782
	0.2	0.0820	0.9180	0.0570	0.7593	0.0800	0.9200	0.0588	0.7399	0.0353	0.9647	0.0260	0.7593	0.0387	0.9613	0.0259	0.7399
	0.3	0.0940	0.9060	0.0597	0.6774	0.0940	0.9060	0.0592	0.6642	0.0410	0.9590	0.0259	0.6774	0.0440	0.9560	0.0256	0.6642
	0.4	0.1010	0.8990	0.0581	0.5932	0.0977	0.9023	0.0573	0.5820	0.0387	0.9613	0.0234	0.5932	0.0397	0.9603	0.0223	0.5820
	0.5	0.1123	0.8877	0.0591	0.5090	0.1160	0.8840	0.0587	0.5039	0.0407	0.9593	0.0245	0.5090	0.0447	0.9553	0.0245	0.5039
	0.6	0.1320	0.8680	0.0587	0.4122	0.1380	0.8620	0.0589	0.4106	0.0490	0.9510	0.0241	0.4122	0.0523	0.9477	0.0239	0.4106
	0.7	0.1520	0.8480	0.0590	0.3254	0.1550	0.8450	0.0599	0.3271	0.0513	0.9487	0.0251	0.3254	0.0547	0.9453	0.0248	0.3271
	0.8	0.2517	0.7483	0.0566	0.2376	0.2520	0.7480	0.0566	0.2397	0.0513	0.9487	0.0244	0.2376	0.0483	0.9517	0.0246	0.2397
	0.85	0.5803	0.4197	0.0530	0.1876	0.6020	0.3980	0.0533	0.1901	0.0640	0.9360	0.0221	0.1876	0.0650	0.9350	0.0219	0.1901
	0.9	0.8690	0.1310	0.0524	0.1437	0.8977	0.1023	0.0527	0.1467	0.0780	0.9220	0.0224	0.1437	0.0840	0.9160	0.0224	0.1467
	0.95	0.9730	0.0270	0.0494	0.0981	0.9857	0.0143	0.0487	0.1003	0.6713	0.3287	0.0178	0.0981	0.7230	0.2770	0.0181	0.1003
	0.99	0.9877	0.0123	0.1161	0.0637	0.9967	0.0033	0.0987	0.0656	0.9787	0.0213	0.0134	0.0637	0.9943	0.0057	0.0124	0.0656
**0.60**	0.1	0.0687	0.9313	0.0563	0.8123	0.0710	0.9290	0.0601	0.7812	0.0337	0.9663	0.0276	0.8123	0.0327	0.9673	0.0294	0.7812
	0.2	0.0823	0.9177	0.0574	0.7555	0.0853	0.9147	0.0595	0.7347	0.0360	0.9640	0.0267	0.7555	0.0393	0.9607	0.0267	0.7347
	0.3	0.1063	0.8937	0.0594	0.6866	0.1107	0.8893	0.0586	0.6717	0.0510	0.9490	0.0239	0.6866	0.0473	0.9527	0.0246	0.6717
	0.4	0.1243	0.8757	0.0583	0.6078	0.1257	0.8743	0.0583	0.5988	0.0557	0.9443	0.0224	0.6078	0.0520	0.9480	0.0215	0.5988
	0.5	0.1520	0.8480	0.0584	0.5184	0.1493	0.8507	0.0584	0.5143	0.0620	0.9380	0.0234	0.5184	0.0600	0.9400	0.0228	0.5143
	0.6	0.1803	0.8197	0.0606	0.4283	0.1787	0.8213	0.0601	0.4262	0.0650	0.9350	0.0241	0.4283	0.0650	0.9350	0.0244	0.4262
	0.7	0.2487	0.7513	0.0600	0.3386	0.2530	0.7470	0.0595	0.3387	0.0710	0.9290	0.0259	0.3386	0.0753	0.9247	0.0260	0.3387
	0.8	0.5480	0.4520	0.0566	0.2492	0.5780	0.4220	0.0552	0.2512	0.0890	0.9110	0.0229	0.2492	0.0900	0.9100	0.0230	0.2512
	0.85	0.7987	0.2013	0.0550	0.2037	0.8220	0.1780	0.0549	0.2063	0.1020	0.8980	0.0233	0.2037	0.1040	0.8960	0.0236	0.2063
	0.9	0.9403	0.0597	0.0536	0.1618	0.9517	0.0483	0.0535	0.1636	0.2850	0.7150	0.0219	0.1618	0.3040	0.6960	0.0221	0.1636
	0.95	0.9810	0.0190	0.0517	0.1168	0.9887	0.0113	0.0506	0.1187	0.9130	0.0870	0.0177	0.1168	0.9317	0.0683	0.0180	0.1187
	0.99	0.9930	0.0070	0.1213	0.0828	0.9993	0.0007	0.0967	0.0848	0.9863	0.0137	0.0140	0.0828	0.9963	0.0037	0.0131	0.0848

Table 2. (continued)

		q=0.95								q=0.99							
		NW				LL				NW				LL			
γ	ρ	TDR	M	S	MCE	TDR	M	S	MCE	TDR	M	S	MCE	TDR	M	S	MCE
**0.70**	0.1	0.0750	0.9250	0.0571	0.8131	0.0823	0.9177	0.0596	0.7848	0.0403	0.9597	0.0282	0.8131	0.0393	0.9607	0.0281	0.7848
	0.2	0.0910	0.9090	0.0562	0.7607	0.0993	0.9007	0.0580	0.7380	0.0413	0.9587	0.0257	0.7607	0.0427	0.9573	0.0251	0.7380
	0.3	0.1220	0.8780	0.0591	0.6889	0.1277	0.8723	0.0595	0.6757	0.0550	0.9450	0.0239	0.6889	0.0543	0.9457	0.0249	0.6757
	0.4	0.1580	0.8420	0.0585	0.6045	0.1597	0.8403	0.0587	0.5942	0.0677	0.9323	0.0231	0.6045	0.0683	0.9317	0.0230	0.5942
	0.5	0.2233	0.7767	0.0576	0.5295	0.2280	0.7720	0.0572	0.5268	0.0887	0.9113	0.0226	0.5295	0.0930	0.9070	0.0229	0.5268
	0.6	0.3100	0.6900	0.0587	0.4383	0.3097	0.6903	0.0587	0.4358	0.1090	0.8910	0.0240	0.4383	0.1090	0.8910	0.0235	0.4358
	0.7	0.4717	0.5283	0.0591	0.3500	0.4817	0.5183	0.0584	0.3506	0.1460	0.8540	0.0256	0.3500	0.1397	0.8603	0.0250	0.3506
	0.8	0.7610	0.2390	0.0555	0.2627	0.7743	0.2257	0.0549	0.2647	0.1817	0.8183	0.0230	0.2627	0.1850	0.8150	0.0228	0.2647
	0.85	0.8950	0.1050	0.0544	0.2197	0.9077	0.0923	0.0542	0.2217	0.3227	0.6773	0.0224	0.2197	0.3297	0.6703	0.0222	0.2217
	0.9	0.9567	0.0433	0.0526	0.1764	0.9653	0.0347	0.0532	0.1791	0.7430	0.2570	0.0219	0.1764	0.7810	0.2190	0.0220	0.1791
	0.95	0.9863	0.0137	0.0492	0.1330	0.9893	0.0107	0.0485	0.1350	0.9603	0.0397	0.0182	0.1330	0.9697	0.0303	0.0181	0.1350
	0.99	0.9893	0.0107	0.1066	0.0986	0.9940	0.0060	0.0899	0.1004	0.9820	0.0180	0.0130	0.0986	0.9890	0.0110	0.0121	0.1004
**0.80**	0.1	0.0673	0.9327	0.0604	0.8119	0.0670	0.9330	0.0619	0.7834	0.0290	0.9710	0.0297	0.8119	0.0270	0.9730	0.0300	0.7834
	0.2	0.1007	0.8993	0.0538	0.7671	0.0997	0.9003	0.0578	0.7441	0.0413	0.9587	0.0242	0.7671	0.0477	0.9523	0.0249	0.7441
	0.3	0.1513	0.8487	0.0578	0.6955	0.1457	0.8543	0.0585	0.6795	0.0607	0.9393	0.0236	0.6955	0.0587	0.9413	0.0231	0.6795
	0.4	0.2097	0.7903	0.0593	0.6165	0.2060	0.7940	0.0589	0.6073	0.0783	0.9217	0.0227	0.6165	0.0733	0.9267	0.0225	0.6073
	0.5	0.2900	0.7100	0.0574	0.5345	0.3010	0.6990	0.0574	0.5297	0.1140	0.8860	0.0221	0.5345	0.1207	0.8793	0.0228	0.5297
	0.6	0.4247	0.5753	0.0588	0.4433	0.4390	0.5610	0.0584	0.4420	0.1887	0.8113	0.0227	0.4433	0.1923	0.8077	0.0229	0.4420
	0.7	0.6537	0.3463	0.0602	0.3620	0.6690	0.3310	0.0590	0.3629	0.2643	0.7357	0.0248	0.3620	0.2773	0.7227	0.0247	0.3629
	0.8	0.8373	0.1627	0.0569	0.2740	0.8507	0.1493	0.0563	0.2761	0.4383	0.5617	0.0238	0.2740	0.4530	0.5470	0.0239	0.2761
	0.85	0.9207	0.0793	0.0566	0.2316	0.9297	0.0703	0.0561	0.2349	0.6750	0.3250	0.0230	0.2316	0.7060	0.2940	0.0231	0.2349
	0.9	0.9660	0.0340	0.0531	0.1899	0.9713	0.0287	0.0534	0.1923	0.8927	0.1073	0.0220	0.1899	0.9080	0.0920	0.0223	0.1923
	0.95	0.9800	0.0200	0.0504	0.1463	0.9893	0.0107	0.0502	0.1490	0.9647	0.0353	0.0180	0.1463	0.9777	0.0223	0.0182	0.1490
	0.99	0.9893	0.0107	0.0921	0.1127	0.9973	0.0027	0.0775	0.1145	0.9860	0.0140	0.0121	0.1127	0.9950	0.0050	0.0119	0.1145

Table 2. (continued)

		**q=0.95**								**q=0.99**							
		NW				LL				NW				LL			
γ	ρ	TDR	M	S	MCE	TDR	M	S	MCE	TDR	M	S	MCE	TDR	M	S	MCE
**0.85**	0.1	0.0723	0.9277	0.0589	0.8180	0.0717	0.9283	0.0602	0.7883	0.0347	0.9653	0.0285	0.8180	0.0380	0.9620	0.0283	0.7883
	0.2	0.0993	0.9007	0.0529	0.7635	0.1030	0.8970	0.0540	0.7408	0.0443	0.9557	0.0237	0.7635	0.0483	0.9517	0.0241	0.7408
	0.3	0.1567	0.8433	0.0555	0.6916	0.1530	0.8470	0.0557	0.6777	0.0710	0.9290	0.0233	0.6916	0.0673	0.9327	0.0233	0.6777
	0.4	0.2393	0.7607	0.0581	0.6222	0.2367	0.7633	0.0584	0.6111	0.0947	0.9053	0.0220	0.6222	0.0930	0.9070	0.0221	0.6111
	0.5	0.3377	0.6623	0.0583	0.5368	0.3357	0.6643	0.0585	0.5310	0.1403	0.8597	0.0228	0.5368	0.1403	0.8597	0.0225	0.5310
	0.6	0.4920	0.5080	0.0591	0.4514	0.4987	0.5013	0.0583	0.4491	0.2057	0.7943	0.0238	0.4514	0.2137	0.7863	0.0227	0.4491
	0.7	0.6997	0.3003	0.0611	0.3676	0.7077	0.2923	0.0611	0.3675	0.3550	0.6450	0.0266	0.3676	0.3700	0.6300	0.0267	0.3675
	0.8	0.8643	0.1357	0.0563	0.2795	0.8733	0.1267	0.0561	0.2822	0.5747	0.4253	0.0238	0.2795	0.5893	0.4107	0.0234	0.2822
	0.85	0.9300	0.0700	0.0548	0.2357	0.9343	0.0657	0.0542	0.2378	0.7900	0.2100	0.0225	0.2357	0.8080	0.1920	0.0226	0.2378
	0.9	0.9680	0.0320	0.0549	0.1967	0.9740	0.0260	0.0552	0.1989	0.9167	0.0833	0.0229	0.1967	0.9253	0.0747	0.0233	0.1989
	0.95	0.9823	0.0177	0.0517	0.1541	0.9880	0.0120	0.0519	0.1563	0.9660	0.0340	0.0201	0.1541	0.9763	0.0237	0.0202	0.1563
	0.99	0.9887	0.0113	0.0789	0.1180	0.9977	0.0023	0.0690	0.1200	0.9840	0.0160	0.0114	0.1180	0.9960	0.0040	0.0109	0.1200
**0.90**	0.1	0.0763	0.9237	0.0580	0.8141	0.0783	0.9217	0.0601	0.7831	0.0400	0.9600	0.0290	0.8141	0.0330	0.9670	0.0291	0.7831
	0.2	0.1007	0.8993	0.0570	0.7691	0.1047	0.8953	0.0592	0.7416	0.0443	0.9557	0.0260	0.7691	0.0450	0.9550	0.0262	0.7416
	0.3	0.1637	0.8363	0.0565	0.7012	0.1567	0.8433	0.0566	0.6826	0.0737	0.9263	0.0229	0.7012	0.0670	0.9330	0.0224	0.6826
	0.4	0.2737	0.7263	0.0564	0.6165	0.2523	0.7477	0.0564	0.6080	0.1223	0.8777	0.0210	0.6165	0.1120	0.8880	0.0211	0.6080
	0.5	0.3670	0.6330	0.0574	0.5364	0.3663	0.6337	0.0574	0.5307	0.1650	0.8350	0.0210	0.5364	0.1677	0.8323	0.0220	0.5307
	0.6	0.5430	0.4570	0.0582	0.4511	0.5570	0.4430	0.0581	0.4499	0.2630	0.7370	0.0228	0.4511	0.2743	0.7257	0.0220	0.4499
	0.7	0.7520	0.2480	0.0600	0.3728	0.7607	0.2393	0.0593	0.3726	0.4363	0.5637	0.0249	0.3728	0.4553	0.5447	0.0252	0.3726
	0.8	0.8923	0.1077	0.0546	0.2797	0.8977	0.1023	0.0538	0.2815	0.6890	0.3110	0.0220	0.2797	0.7090	0.2910	0.0221	0.2815
	0.85	0.9340	0.0660	0.0561	0.2417	0.9410	0.0590	0.0561	0.2441	0.8347	0.1653	0.0233	0.2417	0.8460	0.1540	0.0237	0.2441
	0.9	0.9623	0.0377	0.0527	0.1959	0.9707	0.0293	0.0530	0.1985	0.9217	0.0783	0.0217	0.1959	0.9323	0.0677	0.0217	0.1985
	0.95	0.9877	0.0123	0.0497	0.1555	0.9920	0.0080	0.0487	0.1574	0.9780	0.0220	0.0180	0.1555	0.9847	0.0153	0.0179	0.1574
	0.99	0.9843	0.0157	0.0703	0.1214	0.9963	0.0037	0.0642	0.1238	0.9800	0.0200	0.0116	0.1214	0.9950	0.0050	0.0110	0.1238

Table 3. Simulation results for n=50 , %10 percentage of contamination

		**q=0.95**								**q=0.99**							
		NW				LL				NW				LL			
γ	ρ	TDR	M	S	MCE	TDR	M	S	MCE	TDR	M	S	MCE	TDR	M	S	MCE
**0.10**	0.1	0.0570	0.9430	0.0578	0.8127	0.0656	0.9344	0.0634	0.7794	0.0274	0.9726	0.0287	0.8127	0.0290	0.9710	0.0306	0.7794
	0.2	0.0620	0.9380	0.0618	0.7415	0.0616	0.9384	0.0632	0.7256	0.0266	0.9734	0.0298	0.7415	0.0262	0.9738	0.0308	0.7256
	0.3	0.0660	0.9340	0.0610	0.6669	0.0652	0.9348	0.0620	0.6540	0.0308	0.9692	0.0266	0.6669	0.0280	0.9720	0.0261	0.6540
	0.4	0.0642	0.9358	0.0631	0.5730	0.0646	0.9354	0.0615	0.5642	0.0280	0.9720	0.0268	0.5730	0.0270	0.9730	0.0253	0.5642
	0.5	0.0680	0.9320	0.0630	0.4814	0.0672	0.9328	0.0631	0.4782	0.0272	0.9728	0.0276	0.4814	0.0302	0.9698	0.0286	0.4782
	0.6	0.0616	0.9384	0.0640	0.3828	0.0610	0.9390	0.0638	0.3828	0.0258	0.9742	0.0285	0.3828	0.0278	0.9722	0.0278	0.3828
	0.7	0.0634	0.9366	0.0611	0.2872	0.0638	0.9362	0.0606	0.2891	0.0270	0.9730	0.0271	0.2872	0.0260	0.9740	0.0267	0.2891
	0.8	0.0638	0.9362	0.0580	0.1951	0.0642	0.9358	0.0579	0.1981	0.0260	0.9740	0.0245	0.1951	0.0264	0.9736	0.0247	0.1981
	0.85	0.0634	0.9366	0.0565	0.1477	0.0636	0.9364	0.0567	0.1509	0.0250	0.9750	0.0235	0.1477	0.0260	0.9740	0.0239	0.1509
	0.9	0.0534	0.9466	0.0538	0.0976	0.0546	0.9454	0.0548	0.0998	0.0208	0.9792	0.0221	0.0976	0.0206	0.9794	0.0225	0.0998
	0.95	0.0628	0.9372	0.0499	0.0499	0.0646	0.9354	0.0507	0.0514	0.0144	0.9856	0.0179	0.0499	0.0146	0.9854	0.0179	0.0514
	0.99	0.9018	0.0982	0.0550	0.0136	0.9482	0.0518	0.0547	0.0143	0.0142	0.9858	0.0113	0.0136	0.0150	0.9850	0.0116	0.0143
**0.20**	0.1	0.0592	0.9408	0.0594	0.8110	0.0634	0.9366	0.0613	0.7794	0.0326	0.9674	0.0302	0.8110	0.0292	0.9708	0.0286	0.7794
	0.2	0.0668	0.9332	0.0623	0.7493	0.0668	0.9332	0.0631	0.7282	0.0322	0.9678	0.0300	0.7493	0.0330	0.9670	0.0308	0.7282
	0.3	0.0664	0.9336	0.0632	0.6661	0.0670	0.9330	0.0625	0.6510	0.0308	0.9692	0.0300	0.6661	0.0296	0.9704	0.0275	0.6510
	0.4	0.0766	0.9234	0.0625	0.5825	0.0716	0.9284	0.0619	0.5726	0.0292	0.9708	0.0261	0.5825	0.0280	0.9720	0.0251	0.5726
	0.5	0.0640	0.9360	0.0608	0.4930	0.0666	0.9334	0.0623	0.4875	0.0240	0.9760	0.0245	0.4930	0.0254	0.9746	0.0253	0.4875
	0.6	0.0726	0.9274	0.0628	0.3924	0.0704	0.9296	0.0623	0.3924	0.0338	0.9662	0.0268	0.3924	0.0310	0.9690	0.0266	0.3924
	0.7	0.0800	0.9200	0.0591	0.2966	0.0780	0.9220	0.0588	0.2987	0.0332	0.9668	0.0255	0.2966	0.0340	0.9660	0.0261	0.2987
	0.8	0.0702	0.9298	0.0556	0.2008	0.0702	0.9298	0.0558	0.2042	0.0262	0.9738	0.0221	0.2008	0.0252	0.9748	0.0226	0.2042
	0.85	0.0698	0.9302	0.0548	0.1561	0.0708	0.9292	0.0549	0.1589	0.0268	0.9732	0.0232	0.1561	0.0270	0.9730	0.0233	0.1589
	0.9	0.0644	0.9356	0.0518	0.1073	0.0670	0.9330	0.0524	0.1100	0.0206	0.9794	0.0221	0.1073	0.0206	0.9794	0.0225	0.1100
	0.95	0.3192	0.6808	0.0483	0.0610	0.3746	0.6254	0.0483	0.0627	0.0204	0.9796	0.0178	0.0610	0.0210	0.9790	0.0181	0.0627
	0.99	0.9750	0.0250	0.0833	0.0240	0.9876	0.0124	0.0726	0.0252	0.5266	0.4734	0.0105	0.0240	0.6556	0.3444	0.0108	0.0252

Table 3. (continued)

		q=0.95								q=0.99							
		NW				LL				NW				LL			
γ	ρ	TDR	M	S	MCE	TDR	M	S	MCE	TDR	M	S	MCE	TDR	M	S	MCE
**0.30**	0.1	0.0632	0.9368	0.0602	0.8061	0.0614	0.9386	0.0596	0.7769	0.0324	0.9676	0.0288	0.8061	0.0316	0.9684	0.0292	0.7769
	0.2	0.0608	0.9392	0.0582	0.7527	0.0628	0.9372	0.0606	0.7332	0.0278	0.9722	0.0270	0.7527	0.0296	0.9704	0.0270	0.7332
	0.3	0.0714	0.9286	0.0598	0.6742	0.0764	0.9236	0.0604	0.6589	0.0306	0.9694	0.0259	0.6742	0.0310	0.9690	0.0259	0.6589
	0.4	0.0798	0.9202	0.0605	0.5904	0.0776	0.9224	0.0591	0.5830	0.0344	0.9656	0.0241	0.5904	0.0342	0.9658	0.0237	0.5830
	0.5	0.0684	0.9316	0.0602	0.5006	0.0698	0.9302	0.0609	0.4951	0.0240	0.9760	0.0241	0.5006	0.0266	0.9734	0.0242	0.4951
	0.6	0.0720	0.9280	0.0584	0.4038	0.0738	0.9262	0.0589	0.4022	0.0292	0.9708	0.0233	0.4038	0.0308	0.9692	0.0235	0.4022
	0.7	0.0826	0.9174	0.0594	0.3139	0.0834	0.9166	0.0587	0.3143	0.0348	0.9652	0.0237	0.3139	0.0362	0.9638	0.0240	0.3143
	0.8	0.0804	0.9196	0.0539	0.2176	0.0792	0.9208	0.0536	0.2200	0.0294	0.9706	0.0228	0.2176	0.0298	0.9702	0.0226	0.2200
	0.85	0.0968	0.9032	0.0545	0.1724	0.0942	0.9058	0.0546	0.1762	0.0326	0.9674	0.0228	0.1724	0.0338	0.9662	0.0234	0.1762
	0.9	0.1610	0.8390	0.0505	0.1251	0.1622	0.8378	0.0502	0.1277	0.0302	0.9698	0.0217	0.1251	0.0310	0.9690	0.0220	0.1277
	0.95	0.8312	0.1688	0.0490	0.0792	0.8746	0.1254	0.0493	0.0818	0.0270	0.9730	0.0179	0.0792	0.0274	0.9726	0.0180	0.0818
	0.99	0.9780	0.0220	0.1214	0.0436	0.9934	0.0066	0.0895	0.0455	0.9348	0.0652	0.0119	0.0436	0.9596	0.0404	0.0115	0.0455
**0.40**	0.1	0.0690	0.9310	0.0598	0.8131	0.0658	0.9342	0.0608	0.7857	0.0368	0.9632	0.0309	0.8131	0.0304	0.9696	0.0285	0.7857
	0.2	0.0702	0.9298	0.0572	0.7580	0.0708	0.9292	0.0613	0.7376	0.0346	0.9654	0.0276	0.7580	0.0318	0.9682	0.0282	0.7376
	0.3	0.0766	0.9234	0.0579	0.6826	0.0776	0.9224	0.0572	0.6680	0.0340	0.9660	0.0245	0.6826	0.0318	0.9682	0.0230	0.6680
	0.4	0.0766	0.9234	0.0599	0.6004	0.0778	0.9222	0.0591	0.5893	0.0300	0.9700	0.0237	0.6004	0.0276	0.9724	0.0232	0.5893
	0.5	0.0936	0.9064	0.0574	0.5085	0.0964	0.9036	0.0572	0.5033	0.0354	0.9646	0.0213	0.5085	0.0370	0.9630	0.0224	0.5033
	0.6	0.0786	0.9214	0.0586	0.4215	0.0786	0.9214	0.0594	0.4199	0.0316	0.9684	0.0226	0.4215	0.0302	0.9698	0.0224	0.4199
	0.7	0.1032	0.8968	0.0594	0.3341	0.1016	0.8984	0.0584	0.3348	0.0414	0.9586	0.0244	0.3341	0.0388	0.9612	0.0252	0.3348
	0.8	0.1164	0.8836	0.0556	0.2413	0.1132	0.8868	0.0552	0.2434	0.0368	0.9632	0.0229	0.2413	0.0372	0.9628	0.0226	0.2434
	0.85	0.1788	0.8212	0.0546	0.1961	0.1814	0.8186	0.0548	0.1989	0.0438	0.9562	0.0227	0.1961	0.0442	0.9558	0.0223	0.1989
	0.9	0.5374	0.4626	0.0518	0.1496	0.5818	0.4182	0.0526	0.1525	0.0410	0.9590	0.0217	0.1496	0.0436	0.9564	0.0219	0.1525
	0.95	0.9384	0.0616	0.0502	0.1031	0.9634	0.0366	0.0491	0.1061	0.0714	0.9286	0.0167	0.1031	0.0684	0.9316	0.0173	0.1061
	0.99	0.9850	0.0150	0.1656	0.0675	0.9964	0.0036	0.1139	0.0703	0.9632	0.0368	0.0134	0.0675	0.9866	0.0134	0.0116	0.0703

Table 3. (continued)

		q=0.95								q=0.99							
		NW				LL				NW				LL			
γ	ρ	TDR	M	S	MCE	TDR	M	S	MCE	TDR	M	S	MCE	TDR	M	S	MCE
**0.50**	0.1	0.0666	0.9334	0.0591	0.8123	0.0632	0.9368	0.0603	0.7806	0.0352	0.9648	0.0287	0.8123	0.0288	0.9712	0.0289	0.7806
	0.2	0.0758	0.9242	0.0574	0.7602	0.0810	0.9190	0.0599	0.7366	0.0336	0.9664	0.0260	0.7602	0.0354	0.9646	0.0269	0.7366
	0.3	0.0898	0.9102	0.0566	0.6904	0.0856	0.9144	0.0579	0.6754	0.0368	0.9632	0.0238	0.6904	0.0368	0.9632	0.0222	0.6754
	0.4	0.0922	0.9078	0.0570	0.6077	0.0916	0.9084	0.0559	0.6007	0.0326	0.9674	0.0215	0.6077	0.0334	0.9666	0.0215	0.6007
	0.5	0.0982	0.9018	0.0569	0.5255	0.0972	0.9028	0.0568	0.5199	0.0382	0.9618	0.0213	0.5255	0.0398	0.9602	0.0217	0.5199
	0.6	0.1176	0.8824	0.0572	0.4395	0.1212	0.8788	0.0574	0.4371	0.0390	0.9610	0.0217	0.4395	0.0382	0.9618	0.0215	0.4371
	0.7	0.1282	0.8718	0.0572	0.3487	0.1250	0.8750	0.0566	0.3487	0.0420	0.9580	0.0232	0.3487	0.0406	0.9594	0.0233	0.3487
	0.8	0.2052	0.7948	0.0524	0.2598	0.2008	0.7992	0.0520	0.2614	0.0428	0.9572	0.0210	0.2598	0.0452	0.9548	0.0212	0.2614
	0.85	0.4450	0.5550	0.0541	0.2209	0.4698	0.5302	0.0541	0.2239	0.0514	0.9486	0.0228	0.2209	0.0520	0.9480	0.0227	0.2239
	0.9	0.8272	0.1728	0.0527	0.1776	0.8624	0.1376	0.0522	0.1803	0.0584	0.9416	0.0227	0.1776	0.0586	0.9414	0.0230	0.1803
	0.95	0.9662	0.0338	0.0494	0.1315	0.9754	0.0246	0.0482	0.1339	0.5166	0.4834	0.0172	0.1315	0.5758	0.4242	0.0176	0.1339
	0.99	0.9902	0.0098	0.1968	0.0971	0.9968	0.0032	0.1250	0.0995	0.9770	0.0230	0.0145	0.0971	0.9910	0.0090	0.0119	0.0995
**0.60**	0.1	0.0710	0.9290	0.0593	0.8139	0.0726	0.9274	0.0602	0.7811	0.0360	0.9640	0.0301	0.8139	0.0362	0.9638	0.0296	0.7811
	0.2	0.0778	0.9222	0.0530	0.7676	0.0834	0.9166	0.0572	0.7438	0.0380	0.9620	0.0244	0.7676	0.0352	0.9648	0.0255	0.7438
	0.3	0.0996	0.9004	0.0551	0.7038	0.0956	0.9044	0.0545	0.6841	0.0376	0.9624	0.0221	0.7038	0.0406	0.9594	0.0218	0.6841
	0.4	0.1282	0.8718	0.0527	0.6240	0.1268	0.8732	0.0530	0.6103	0.0464	0.9536	0.0196	0.6240	0.0500	0.9500	0.0196	0.6103
	0.5	0.1384	0.8616	0.0548	0.5374	0.1412	0.8588	0.0551	0.5316	0.0528	0.9472	0.0202	0.5374	0.0494	0.9506	0.0198	0.5316
	0.6	0.1682	0.8318	0.0558	0.4582	0.1698	0.8302	0.0570	0.4561	0.0606	0.9394	0.0216	0.4582	0.0576	0.9424	0.0210	0.4561
	0.7	0.2254	0.7746	0.0584	0.3743	0.2280	0.7720	0.0578	0.3749	0.0714	0.9286	0.0224	0.3743	0.0692	0.9308	0.0225	0.3749
	0.8	0.4594	0.5406	0.0545	0.2878	0.4740	0.5260	0.0547	0.2897	0.0744	0.9256	0.0229	0.2878	0.0730	0.9270	0.0231	0.2897
	0.85	0.7392	0.2608	0.0540	0.2481	0.7716	0.2284	0.0543	0.2499	0.0860	0.9140	0.0226	0.2481	0.0896	0.9104	0.0224	0.2499
	0.9	0.9256	0.0744	0.0507	0.2032	0.9408	0.0592	0.0510	0.2058	0.2206	0.7794	0.0206	0.2032	0.2212	0.7788	0.0208	0.2058
	0.95	0.9796	0.0204	0.0521	0.1639	0.9844	0.0156	0.0505	0.1659	0.8828	0.1172	0.0198	0.1639	0.9062	0.0938	0.0193	0.1659
	0.99	0.9922	0.0078	0.2046	0.1282	0.9980	0.0020	0.1312	0.1307	0.9838	0.0162	0.0148	0.1282	0.9946	0.0054	0.0118	0.1307

Table 3. (continued)

		q=0.95								q=0.99							
		NW				LL				NW				LL			
γ	ρ	TDR	M	S	MCE	TDR	M	S	MCE	TDR	M	S	MCE	TDR	M	S	MCE
**0.70**	0.1	0.0678	0.9322	0.0555	0.8118	0.0678	0.9322	0.0585	0.7824	0.0316	0.9684	0.0269	0.8118	0.0304	0.9696	0.0288	0.7824
	0.2	0.0854	0.9146	0.0524	0.7723	0.0842	0.9158	0.0581	0.7440	0.0390	0.9610	0.0228	0.7723	0.0400	0.9600	0.0258	0.7440
	0.3	0.1224	0.8776	0.0542	0.7071	0.1222	0.8778	0.0532	0.6901	0.0504	0.9496	0.0219	0.7071	0.0484	0.9516	0.0208	0.6901
	0.4	0.1442	0.8558	0.0522	0.6369	0.1488	0.8512	0.0522	0.6258	0.0520	0.9480	0.0179	0.6369	0.0536	0.9464	0.0178	0.6258
	0.5	0.1930	0.8070	0.0536	0.5506	0.1926	0.8074	0.0529	0.5440	0.0648	0.9352	0.0193	0.5506	0.0662	0.9338	0.0193	0.5440
	0.6	0.2598	0.7402	0.0557	0.4711	0.2634	0.7366	0.0558	0.4676	0.0866	0.9134	0.0198	0.4711	0.0888	0.9112	0.0195	0.4676
	0.7	0.4082	0.5918	0.0569	0.3942	0.4124	0.5876	0.0570	0.3934	0.1160	0.8840	0.0238	0.3942	0.1156	0.8844	0.0235	0.3934
	0.8	0.7188	0.2812	0.0526	0.3079	0.7354	0.2646	0.0522	0.3088	0.1498	0.8502	0.0226	0.3079	0.1490	0.8510	0.0224	0.3088
	0.85	0.8810	0.1190	0.0541	0.2716	0.8930	0.1070	0.0539	0.2729	0.2546	0.7454	0.0213	0.2716	0.2628	0.7372	0.0211	0.2729
	0.9	0.9492	0.0508	0.0506	0.2289	0.9596	0.0404	0.0510	0.2313	0.6672	0.3328	0.0211	0.2289	0.7196	0.2804	0.0208	0.2313
	0.95	0.9840	0.0160	0.0500	0.1894	0.9900	0.0100	0.0480	0.1919	0.9454	0.0546	0.0170	0.1894	0.9630	0.0370	0.0173	0.1919
	0.99	0.9902	0.0098	0.1783	0.1568	0.9952	0.0048	0.1210	0.1589	0.9830	0.0170	0.0153	0.1568	0.9908	0.0092	0.0130	0.1589
**0.80**	0.1	0.0636	0.9364	0.0559	0.8257	0.0652	0.9348	0.0593	0.7924	0.0278	0.9722	0.0263	0.8257	0.0300	0.9700	0.0277	0.7924
	0.2	0.0940	0.9060	0.0551	0.7726	0.0910	0.9090	0.0579	0.7490	0.0428	0.9572	0.0246	0.7726	0.0412	0.9588	0.0246	0.7490
	0.3	0.1336	0.8664	0.0534	0.7142	0.1332	0.8668	0.0551	0.6931	0.0578	0.9422	0.0205	0.7142	0.0554	0.9446	0.0212	0.6931
	0.4	0.1978	0.8022	0.0537	0.6412	0.1868	0.8132	0.0510	0.6284	0.0760	0.9240	0.0176	0.6412	0.0662	0.9338	0.0173	0.6284
	0.5	0.2548	0.7452	0.0533	0.5648	0.2610	0.7390	0.0534	0.5575	0.0954	0.9046	0.0181	0.5648	0.0964	0.9036	0.0188	0.5575
	0.6	0.4004	0.5996	0.0542	0.4865	0.3992	0.6008	0.0542	0.4836	0.1528	0.8472	0.0196	0.4865	0.1528	0.8472	0.0194	0.4836
	0.7	0.6088	0.3912	0.0588	0.4114	0.6202	0.3798	0.0581	0.4112	0.2302	0.7698	0.0242	0.4114	0.2378	0.7622	0.0239	0.4112
	0.8	0.8436	0.1564	0.0538	0.3280	0.8546	0.1454	0.0532	0.3291	0.3892	0.6108	0.0216	0.3280	0.4036	0.5964	0.0214	0.3291
	0.85	0.9220	0.0780	0.0538	0.2921	0.9296	0.0704	0.0538	0.2943	0.6454	0.3546	0.0230	0.2921	0.6714	0.3286	0.0232	0.2943
	0.9	0.9650	0.0350	0.0514	0.2530	0.9710	0.0290	0.0519	0.2550	0.8794	0.1206	0.0218	0.2530	0.8966	0.1034	0.0218	0.2550
	0.95	0.9868	0.0132	0.0498	0.2134	0.9894	0.0106	0.0489	0.2153	0.9668	0.0332	0.0180	0.2134	0.9738	0.0262	0.0180	0.2153
	0.99	0.9946	0.0054	0.1310	0.1826	0.9980	0.0020	0.0979	0.1850	0.9896	0.0104	0.0138	0.1826	0.9972	0.0028	0.0114	0.1850

Table 3. (continued)

		**q=0.95**								**q=0.99**							
		NW				LL				NW				LL			
γ	ρ	TDR	M	S	MCE	TDR	M	S	MCE	TDR	M	S	MCE	TDR	M	S	MCE
**0.85**	0.1	0.0718	0.9282	0.0587	0.8136	0.0678	0.9322	0.0612	0.7831	0.0328	0.9672	0.0284	0.8136	0.0310	0.9690	0.0283	0.7831
	0.2	0.0984	0.9016	0.0502	0.7815	0.1016	0.8984	0.0559	0.7512	0.0448	0.9552	0.0209	0.7815	0.0460	0.9540	0.0229	0.7512
	0.3	0.1436	0.8564	0.0514	0.7111	0.1496	0.8504	0.0523	0.6948	0.0554	0.9446	0.0194	0.7111	0.0614	0.9386	0.0204	0.6948
	0.4	0.2090	0.7910	0.0509	0.6467	0.2074	0.7926	0.0505	0.6334	0.0800	0.9200	0.0177	0.6467	0.0778	0.9222	0.0181	0.6334
	0.5	0.2990	0.7010	0.0516	0.5705	0.3032	0.6968	0.0521	0.5639	0.1178	0.8822	0.0183	0.5705	0.1234	0.8766	0.0187	0.5639
	0.6	0.4626	0.5374	0.0554	0.4956	0.4682	0.5318	0.0540	0.4912	0.1906	0.8094	0.0207	0.4956	0.1940	0.8060	0.0193	0.4912
	0.7	0.6694	0.3306	0.0580	0.4167	0.6840	0.3160	0.0567	0.4166	0.3216	0.6784	0.0239	0.4167	0.3270	0.6730	0.0242	0.4166
	0.8	0.8718	0.1282	0.0554	0.3397	0.8798	0.1202	0.0544	0.3414	0.5482	0.4518	0.0224	0.3397	0.5664	0.4336	0.0224	0.3414
	0.85	0.9290	0.0710	0.0557	0.3014	0.9362	0.0638	0.0549	0.3032	0.7456	0.2544	0.0221	0.3014	0.7674	0.2326	0.0226	0.3032
	0.9	0.9668	0.0332	0.0547	0.2634	0.9734	0.0266	0.0539	0.2659	0.9088	0.0912	0.0222	0.2634	0.9226	0.0774	0.0224	0.2659
	0.95	0.9830	0.0170	0.0527	0.2231	0.9904	0.0096	0.0519	0.2258	0.9690	0.0310	0.0185	0.2231	0.9802	0.0198	0.0186	0.2258
	0.99	0.9894	0.0106	0.1068	0.1904	0.9946	0.0054	0.0887	0.1933	0.9836	0.0164	0.0138	0.1904	0.9926	0.0074	0.0125	0.1933
**0.90**	0.1	0.0628	0.9372	0.0589	0.8192	0.0658	0.9342	0.0599	0.7886	0.0302	0.9698	0.0283	0.8192	0.0300	0.9700	0.0278	0.7886
	0.2	0.0982	0.9018	0.0512	0.7784	0.1016	0.8984	0.0540	0.7539	0.0394	0.9606	0.0210	0.7784	0.0440	0.9560	0.0213	0.7539
	0.3	0.1528	0.8472	0.0509	0.7145	0.1436	0.8564	0.0513	0.6961	0.0644	0.9356	0.0196	0.7145	0.0604	0.9396	0.0197	0.6961
	0.4	0.2222	0.7778	0.0508	0.6499	0.2238	0.7762	0.0524	0.6396	0.0818	0.9182	0.0172	0.6499	0.0856	0.9144	0.0182	0.6396
	0.5	0.3502	0.6498	0.0538	0.5770	0.3482	0.6518	0.0537	0.5696	0.1388	0.8612	0.0174	0.5770	0.1406	0.8594	0.0188	0.5696
	0.6	0.5198	0.4802	0.0560	0.5036	0.5196	0.4804	0.0558	0.5007	0.2286	0.7714	0.0202	0.5036	0.2356	0.7644	0.0207	0.5007
	0.7	0.7088	0.2912	0.0571	0.4211	0.7114	0.2886	0.0560	0.4202	0.4012	0.5988	0.0228	0.4211	0.4116	0.5884	0.0226	0.4202
	0.8	0.8866	0.1134	0.0555	0.3450	0.8846	0.1154	0.0552	0.3454	0.6676	0.3324	0.0218	0.3450	0.6812	0.3188	0.0219	0.3454
	0.85	0.9388	0.0612	0.0564	0.3100	0.9432	0.0568	0.0563	0.3120	0.8218	0.1782	0.0241	0.3100	0.8426	0.1574	0.0238	0.3120
	0.9	0.9682	0.0318	0.0506	0.2653	0.9728	0.0272	0.0513	0.2679	0.9220	0.0780	0.0210	0.2653	0.9312	0.0688	0.0215	0.2679
	0.95	0.9834	0.0166	0.0494	0.2281	0.9886	0.0114	0.0491	0.2308	0.9718	0.0282	0.0173	0.2281	0.9796	0.0204	0.0175	0.2308
	0.99	0.9884	0.0116	0.0922	0.1979	0.9950	0.0050	0.0785	0.2005	0.9842	0.0158	0.0121	0.1979	0.9938	0.0062	0.0112	0.2005
