# Supplementary material for: A method for detecting outliers in linear-circular non-parametric regression
Source: PLoS One. 2023 Jun 12;18(6):e0286448. doi: 10.1371/journal.pone.0286448 (PMC10259788; doi:10.1371/journal.pone.0286448)
Supplement: S4 File — (PDF) [file pone.0286448.s004.pdf]

A Method for Detecting Outliers in Linear-Circular Non-Parametric Regression

Sümeyra Sert[1]* *and* Filiz Kardiyen[2]

[1] *Selcuk University, Department of Statistics, 42250, Selcuklu, Konya, Turkey;*

[1]ORCID id: https://orcid.org/0000-0002-4647-1583

sumeyra.sert@selcuk.edu.tr

[2]*Gazi University, Department of Statistics, Teknikokullar, 06500, Ankara, Turkey.*

[2]ORCID id: https://orcid.org/0000-0002-8730-2751

fyuva@gazi.edu.tr

Supplementary File (n=100)

Table 1. Simulation results for n=100, %1 percentage of contamination

		q=0.95								q=0.99							
		NW				LL				NW				LL			
γ	ρ	TDR	M	S	MCE	TDR	M	S	MCE	TDR	M	S	MCE	TDR	M	S	MCE
**0.10**	0.1	0.0600	0.9400	0.0564	0.8481	0.0730	0.9270	0.0557	0.8283	0.0320	0.9680	0.0235	0.8481	0.0270	0.9730	0.0225	0.8283
	0.2	0.0570	0.9430	0.0559	0.7778	0.0640	0.9360	0.0572	0.7649	0.0220	0.9780	0.0211	0.7778	0.0210	0.9790	0.0224	0.7649
	0.3	0.0690	0.9310	0.0571	0.6839	0.0740	0.9260	0.0568	0.6782	0.0240	0.9760	0.0200	0.6839	0.0230	0.9770	0.0208	0.6782
	0.4	0.0590	0.9410	0.0577	0.5838	0.0610	0.9390	0.0578	0.5827	0.0200	0.9800	0.0201	0.5838	0.0170	0.9830	0.0202	0.5827
	0.5	0.0530	0.9470	0.0579	0.4870	0.0470	0.9530	0.0575	0.4882	0.0120	0.9880	0.0190	0.4870	0.0150	0.9850	0.0191	0.4882
	0.6	0.0480	0.9520	0.0578	0.3875	0.0530	0.9470	0.0577	0.3896	0.0110	0.9890	0.0197	0.3875	0.0100	0.9900	0.0196	0.3896
	0.7	0.0610	0.9390	0.0560	0.2918	0.0590	0.9410	0.0560	0.2945	0.0240	0.9760	0.0183	0.2918	0.0220	0.9780	0.0186	0.2945
	0.8	0.0580	0.9420	0.0556	0.1968	0.0590	0.9410	0.0557	0.1995	0.0180	0.9820	0.0188	0.1968	0.0160	0.9840	0.0188	0.1995
	0.85	0.0570	0.9430	0.0544	0.1448	0.0580	0.9420	0.0548	0.1467	0.0200	0.9800	0.0180	0.1448	0.0190	0.9810	0.0178	0.1467
	0.9	0.0620	0.9380	0.0519	0.0963	0.0610	0.9390	0.0517	0.0978	0.0200	0.9800	0.0183	0.0963	0.0200	0.9800	0.0182	0.0978
	0.95	0.0560	0.9440	0.0505	0.0475	0.0610	0.9390	0.0505	0.0483	0.0130	0.9870	0.0152	0.0475	0.0130	0.9870	0.0152	0.0483
	0.99	0.9690	0.0310	0.0527	0.0100	0.9820	0.0180	0.0514	0.0102	0.0140	0.9860	0.0116	0.0100	0.0150	0.9850	0.0117	0.0102
**0.20**	0.1	0.0670	0.9330	0.0538	0.8499	0.0570	0.9430	0.0544	0.8311	0.0310	0.9690	0.0223	0.8499	0.0210	0.9790	0.0219	0.8311
	0.2	0.0740	0.9260	0.0580	0.7726	0.0700	0.9300	0.0574	0.7613	0.0310	0.9690	0.0228	0.7726	0.0190	0.9810	0.0213	0.7613
	0.3	0.0630	0.9370	0.0547	0.6877	0.0610	0.9390	0.0553	0.6821	0.0190	0.9810	0.0184	0.6877	0.0210	0.9790	0.0190	0.6821
	0.4	0.0550	0.9450	0.0568	0.5858	0.0610	0.9390	0.0565	0.5831	0.0180	0.9820	0.0196	0.5858	0.0210	0.9790	0.0190	0.5831
	0.5	0.0710	0.9290	0.0575	0.4910	0.0700	0.9300	0.0580	0.4924	0.0220	0.9780	0.0192	0.4910	0.0230	0.9770	0.0191	0.4924
	0.6	0.0560	0.9440	0.0565	0.3931	0.0580	0.9420	0.0567	0.3951	0.0190	0.9810	0.0188	0.3931	0.0190	0.9810	0.0185	0.3951
	0.7	0.0760	0.9240	0.0562	0.2919	0.0750	0.9250	0.0557	0.2950	0.0230	0.9770	0.0188	0.2919	0.0220	0.9780	0.0187	0.2950
	0.8	0.0550	0.9450	0.0544	0.1949	0.0530	0.9470	0.0541	0.1974	0.0190	0.9810	0.0177	0.1949	0.0200	0.9800	0.0177	0.1974
	0.85	0.0790	0.9210	0.0539	0.1464	0.0790	0.9210	0.0541	0.1486	0.0270	0.9730	0.0181	0.1464	0.0270	0.9730	0.0183	0.1486
	0.9	0.0700	0.9300	0.0509	0.0963	0.0710	0.9290	0.0511	0.0979	0.0160	0.9840	0.0172	0.0963	0.0180	0.9820	0.0172	0.0979
	0.95	0.5610	0.4390	0.0514	0.0497	0.6330	0.3670	0.0517	0.0508	0.0160	0.9840	0.0156	0.0497	0.0170	0.9830	0.0157	0.0508
	0.99	0.9840	0.0160	0.0515	0.0108	0.9940	0.0060	0.0507	0.0111	0.9040	0.0960	0.0115	0.0108	0.9280	0.0720	0.0114	0.0111

Table 1. (continued)

		q=0.95								q=0.99							
		NW				LL				NW				LL			
γ	ρ	TDR	M	S	MCE	TDR	M	S	MCE	TDR	M	S	MCE	TDR	M	S	MCE
**0.30**	0.1	0.062	0.9380	0.0532	0.8504	0.05	0.9500	0.0560	0.8280	0.0240	0.9760	0.0223	0.8504	0.0210	0.9790	0.0239	0.8280
	0.2	0.067	0.9330	0.0548	0.7786	0.06	0.9400	0.0561	0.7656	0.0310	0.9690	0.0205	0.7786	0.0300	0.9700	0.0206	0.7656
	0.3	0.07	0.9300	0.0566	0.6785	0.066	0.9340	0.0560	0.6736	0.0250	0.9750	0.0201	0.6785	0.0270	0.9730	0.0199	0.6736
	0.4	0.075	0.9250	0.0576	0.5826	0.067	0.9330	0.0575	0.5820	0.0330	0.9670	0.0192	0.5826	0.0320	0.9680	0.0196	0.5820
	0.5	0.064	0.9360	0.0571	0.4890	0.067	0.9330	0.0576	0.4905	0.0230	0.9770	0.0199	0.4890	0.0230	0.9770	0.0194	0.4905
	0.6	0.076	0.9240	0.0572	0.3928	0.083	0.9170	0.0571	0.3951	0.0290	0.9710	0.0184	0.3928	0.0260	0.9740	0.0185	0.3951
	0.7	0.079	0.9210	0.0568	0.2972	0.081	0.9190	0.0567	0.2994	0.0330	0.9670	0.0195	0.2972	0.0370	0.9630	0.0195	0.2994
	0.8	0.08	0.9200	0.0531	0.1943	0.083	0.9170	0.0533	0.1968	0.0220	0.9780	0.0182	0.1943	0.0200	0.9800	0.0185	0.1968
	0.85	0.09	0.9100	0.0545	0.1486	0.095	0.9050	0.0544	0.1509	0.0270	0.9730	0.0187	0.1486	0.0270	0.9730	0.0187	0.1509
	0.9	0.197	0.8030	0.0516	0.0992	0.201	0.7990	0.0515	0.1008	0.0180	0.9820	0.0177	0.0992	0.0180	0.9820	0.0176	0.1008
	0.95	0.936	0.0640	0.0513	0.0516	0.949	0.0510	0.0513	0.0527	0.0330	0.9670	0.0155	0.0516	0.0340	0.9660	0.0155	0.0527
	0.99	0.991	0.0090	0.0551	0.0128	0.998	0.0020	0.0527	0.0132	0.9820	0.0180	0.0118	0.0128	0.9910	0.0090	0.0118	0.0132
**0.40**	0.1	0.054	0.9460	0.0529	0.8474	0.06	0.9400	0.0541	0.8277	0.0250	0.9750	0.0221	0.8474	0.0260	0.9740	0.0221	0.8277
	0.2	0.075	0.9250	0.0564	0.7756	0.077	0.9230	0.0571	0.7644	0.0360	0.9640	0.0221	0.7756	0.0310	0.9690	0.0214	0.7644
	0.3	0.089	0.9110	0.0558	0.6832	0.09	0.9100	0.0563	0.6768	0.0250	0.9750	0.0193	0.6832	0.0330	0.9670	0.0204	0.6768
	0.4	0.082	0.9180	0.0573	0.5864	0.091	0.9090	0.0569	0.5848	0.0370	0.9630	0.0191	0.5864	0.0330	0.9670	0.0190	0.5848
	0.5	0.08	0.9200	0.0584	0.4933	0.083	0.9170	0.0586	0.4948	0.0280	0.9720	0.0194	0.4933	0.0260	0.9740	0.0192	0.4948
	0.6	0.088	0.9120	0.0562	0.3905	0.087	0.9130	0.0568	0.3936	0.0250	0.9750	0.0184	0.3905	0.0280	0.9720	0.0180	0.3936
	0.7	0.113	0.8870	0.0553	0.2952	0.115	0.8850	0.0554	0.2981	0.0390	0.9610	0.0184	0.2952	0.0390	0.9610	0.0185	0.2981
	0.8	0.122	0.8780	0.0548	0.1993	0.123	0.8770	0.0549	0.2019	0.0330	0.9670	0.0186	0.1993	0.0330	0.9670	0.0184	0.2019
	0.85	0.195	0.8050	0.0543	0.1517	0.199	0.8010	0.0544	0.1542	0.0240	0.9760	0.0185	0.1517	0.0260	0.9740	0.0184	0.1542
	0.9	0.723	0.2770	0.0514	0.1006	0.756	0.2440	0.0516	0.1023	0.0280	0.9720	0.0169	0.1006	0.0280	0.9720	0.0168	0.1023
	0.95	0.974	0.0260	0.0513	0.0547	0.983	0.0170	0.0515	0.0558	0.0610	0.9390	0.0155	0.0547	0.0610	0.9390	0.0157	0.0558
	0.99	0.993	0.0070	0.0563	0.0158	0.999	0.0010	0.0533	0.0162	0.9860	0.0140	0.0117	0.0158	0.9960	0.0040	0.0117	0.0162

Table 1. (continued)

		**q=0.95**								**q=0.99**							
		NW				LL				NW				LL			
γ	ρ	TDR	M	S	MCE	TDR	M	S	MCE	TDR	M	S	MCE	TDR	M	S	MCE
**0.50**	0.1	0.059	0.9410	0.0539	0.8507	0.057	0.9430	0.0549	0.8310	0.0260	0.9740	0.0223	0.8507	0.0230	0.9770	0.0226	0.8310
	0.2	0.08	0.9200	0.0553	0.7738	0.078	0.9220	0.0555	0.7630	0.0330	0.9670	0.0212	0.7738	0.0310	0.9690	0.0214	0.7630
	0.3	0.083	0.9170	0.0540	0.6841	0.078	0.9220	0.0553	0.6798	0.0230	0.9770	0.0177	0.6841	0.0260	0.9740	0.0189	0.6798
	0.4	0.108	0.8920	0.0561	0.5858	0.107	0.8930	0.0561	0.5839	0.0330	0.9670	0.0189	0.5858	0.0260	0.9740	0.0186	0.5839
	0.5	0.11	0.8900	0.0561	0.4916	0.11	0.8900	0.0568	0.4927	0.0420	0.9580	0.0182	0.4916	0.0390	0.9610	0.0187	0.4927
	0.6	0.113	0.8870	0.0570	0.3941	0.117	0.8830	0.0571	0.3962	0.0440	0.9560	0.0188	0.3941	0.0460	0.9540	0.0187	0.3962
	0.7	0.129	0.8710	0.0563	0.2960	0.137	0.8630	0.0563	0.2991	0.0380	0.9620	0.0189	0.2960	0.0410	0.9590	0.0190	0.2991
	0.8	0.228	0.7720	0.0554	0.2017	0.236	0.7640	0.0552	0.2043	0.0450	0.9550	0.0189	0.2017	0.0450	0.9550	0.0190	0.2043
	0.85	0.576	0.4240	0.0539	0.1528	0.607	0.3930	0.0539	0.1547	0.0510	0.9490	0.0181	0.1528	0.0480	0.9520	0.0183	0.1547
	0.9	0.919	0.0810	0.0532	0.1062	0.934	0.0660	0.0533	0.1077	0.0510	0.9490	0.0175	0.1062	0.0530	0.9470	0.0175	0.1077
	0.95	0.983	0.0170	0.0493	0.0562	0.986	0.0140	0.0494	0.0572	0.4160	0.5840	0.0151	0.0562	0.4620	0.5380	0.0153	0.0572
	0.99	0.992	0.0080	0.0596	0.0186	0.999	0.0010	0.0552	0.0191	0.9880	0.0120	0.0119	0.0186	0.9980	0.0020	0.0117	0.0191
**0.60**	0.1	0.0470	0.9530	0.0559	0.8504	0.064	0.9360	0.0546	0.8329	0.0190	0.9810	0.0235	0.8504	0.0220	0.9780	0.0215	0.8329
	0.2	0.0960	0.9040	0.0550	0.7773	0.086	0.9140	0.0565	0.7655	0.0510	0.9490	0.0212	0.7773	0.0380	0.9620	0.0217	0.7655
	0.3	0.1120	0.8880	0.0547	0.6869	0.105	0.8950	0.0556	0.6808	0.0330	0.9670	0.0182	0.6869	0.0370	0.9630	0.0192	0.6808
	0.4	0.1350	0.8650	0.0567	0.5897	0.137	0.8630	0.0569	0.5866	0.0410	0.9590	0.0197	0.5897	0.0430	0.9570	0.0191	0.5866
	0.5	0.1550	0.8450	0.0565	0.4956	0.163	0.8370	0.0577	0.4956	0.0540	0.9460	0.0184	0.4956	0.0570	0.9430	0.0188	0.4956
	0.6	0.1550	0.8450	0.0573	0.3996	0.164	0.8360	0.0573	0.4019	0.0430	0.9570	0.0187	0.3996	0.0430	0.9570	0.0185	0.4019
	0.7	0.2280	0.7720	0.0574	0.3032	0.222	0.7780	0.0571	0.3060	0.0510	0.9490	0.0196	0.3032	0.0550	0.9450	0.0193	0.3060
	0.8	0.5560	0.4440	0.0546	0.2054	0.582	0.4180	0.0540	0.2076	0.0680	0.9320	0.0190	0.2054	0.0720	0.9280	0.0189	0.2076
	0.85	0.8340	0.1660	0.0547	0.1579	0.844	0.1560	0.0545	0.1600	0.0810	0.9190	0.0187	0.1579	0.0840	0.9160	0.0188	0.1600
	0.9	0.9410	0.0590	0.0518	0.1081	0.952	0.0480	0.0523	0.1097	0.1100	0.8900	0.0181	0.1081	0.1160	0.8840	0.0181	0.1097
	0.95	0.9830	0.0170	0.0517	0.0606	0.989	0.0110	0.0515	0.0616	0.8930	0.1070	0.0159	0.0606	0.9070	0.0930	0.0158	0.0616
	0.99	0.9980	0.0020	0.0598	0.0222	1	0.0000	0.0549	0.0226	0.9920	0.0080	0.0119	0.0222	0.9990	0.0010	0.0117	0.0226

Table 1. (continued)

		q=0.95								q=0.99							
		NW				LL				NW				LL			
γ	ρ	TDR	M	S	MCE	TDR	M	S	MCE	TDR	M	S	MCE	TDR	M	S	MCE
**0.70**	0.1	0.0670	0.9330	0.0545	0.8534	0.078	0.9220	0.0558	0.8317	0.032	0.9680	0.0231	0.8534	0.0210	0.9790	0.0226	0.8317
	0.2	0.0940	0.9060	0.0577	0.7760	0.081	0.9190	0.0583	0.7639	0.034	0.9660	0.0225	0.7760	0.0280	0.9720	0.0224	0.7639
	0.3	0.1330	0.8670	0.0538	0.6870	0.134	0.8660	0.0546	0.6818	0.045	0.9550	0.0177	0.6870	0.0440	0.9560	0.0184	0.6818
	0.4	0.1730	0.8270	0.0562	0.5947	0.178	0.8220	0.0559	0.5924	0.057	0.9430	0.0184	0.5947	0.0600	0.9400	0.0184	0.5924
	0.5	0.2250	0.7750	0.0570	0.4941	0.224	0.7760	0.0571	0.4951	0.07	0.9300	0.0189	0.4941	0.0850	0.9150	0.0186	0.4951
	0.6	0.2950	0.7050	0.0572	0.3976	0.291	0.7090	0.0575	0.4007	0.084	0.9160	0.0185	0.3976	0.0770	0.9230	0.0190	0.4007
	0.7	0.4430	0.5570	0.0565	0.3055	0.46	0.5400	0.0566	0.3077	0.103	0.8970	0.0185	0.3055	0.0990	0.9010	0.0189	0.3077
	0.8	0.7900	0.2100	0.0550	0.2069	0.812	0.1880	0.0551	0.2095	0.115	0.8850	0.0185	0.2069	0.1240	0.8760	0.0185	0.2095
	0.85	0.9100	0.0900	0.0539	0.1584	0.913	0.0870	0.0542	0.1603	0.17	0.8300	0.0186	0.1584	0.1780	0.8220	0.0185	0.1603
	0.9	0.9700	0.0300	0.0528	0.1109	0.973	0.0270	0.0532	0.1127	0.552	0.4480	0.0178	0.1109	0.5810	0.4190	0.0177	0.1127
	0.95	0.9900	0.0100	0.0510	0.0625	0.993	0.0070	0.0506	0.0636	0.962	0.0380	0.0154	0.0625	0.9730	0.0270	0.0153	0.0636
	0.99	0.9860	0.0140	0.0571	0.0251	1	0.0000	0.0534	0.0257	0.984	0.0160	0.0125	0.0251	1.0000	0.0000	0.0125	0.0257
**0.80**	0.1	0.0760	0.9240	0.0554	0.8501	0.0840	0.9160	0.0535	0.8322	0.0310	0.9690	0.0229	0.8501	0.0450	0.9550	0.0214	0.8322
	0.2	0.0920	0.9080	0.0563	0.7792	0.1090	0.8910	0.0565	0.7674	0.0330	0.9670	0.0207	0.7792	0.0290	0.9710	0.0213	0.7674
	0.3	0.1600	0.8400	0.0534	0.6892	0.1510	0.8490	0.0536	0.6838	0.0560	0.9440	0.0179	0.6892	0.0490	0.9510	0.0177	0.6838
	0.4	0.2280	0.7720	0.0561	0.5887	0.2310	0.7690	0.0565	0.5873	0.0870	0.9130	0.0184	0.5887	0.0790	0.9210	0.0184	0.5873
	0.5	0.2980	0.7020	0.0566	0.4921	0.3100	0.6900	0.0569	0.4928	0.1000	0.9000	0.0188	0.4921	0.1080	0.8920	0.0196	0.4928
	0.6	0.4400	0.5600	0.0564	0.3981	0.4440	0.5560	0.0562	0.4007	0.1230	0.8770	0.0185	0.3981	0.1100	0.8900	0.0184	0.4007
	0.7	0.6570	0.3430	0.0577	0.3054	0.6720	0.3280	0.0574	0.3084	0.2030	0.7970	0.0195	0.3054	0.2010	0.7990	0.0194	0.3084
	0.8	0.8650	0.1350	0.0540	0.2066	0.8820	0.1180	0.0538	0.2092	0.3030	0.6970	0.0187	0.2066	0.3140	0.6860	0.0187	0.2092
	0.85	0.9250	0.0750	0.0539	0.1600	0.9340	0.0660	0.0541	0.1622	0.5340	0.4660	0.0176	0.1600	0.5600	0.4400	0.0177	0.1622
	0.9	0.9760	0.0240	0.0525	0.1131	0.9810	0.0190	0.0524	0.1147	0.8720	0.1280	0.0178	0.1131	0.8910	0.1090	0.0181	0.1147
	0.95	0.9870	0.0130	0.0515	0.0646	0.9910	0.0090	0.0513	0.0657	0.9680	0.0320	0.0154	0.0646	0.9730	0.0270	0.0155	0.0657
	0.99	0.9930	0.0070	0.0542	0.0269	0.9980	0.0020	0.0517	0.0274	0.9910	0.0090	0.0119	0.0269	0.9980	0.0020	0.0119	0.0274

Table 1. (continued)

		**q=0.95**								**q=0.99**							
		NW				LL				NW				LL			
γ	ρ	TDR	M	S	MCE	TDR	M	S	MCE	TDR	M	S	MCE	TDR	M	S	MCE
	0.1	0.0580	0.9420	0.0548	0.8483	0.058	0.942	0.0555	0.8298	0.028	0.972	0.0230	0.8483	0.025	0.975	0.0225	0.8298
	0.2	0.1190	0.8810	0.0554	0.7777	0.118	0.882	0.0558	0.7668	0.043	0.957	0.0205	0.7777	0.041	0.959	0.0202	0.7668
	0.3	0.1380	0.8620	0.0542	0.6849	0.143	0.857	0.0560	0.6790	0.051	0.949	0.0186	0.6849	0.061	0.939	0.0197	0.6790
	0.4	0.2140	0.7860	0.0560	0.5927	0.217	0.783	0.0571	0.5917	0.066	0.934	0.0191	0.5927	0.07	0.93	0.0190	0.5917
**0.85**	0.5	0.3370	0.6630	0.0558	0.4948	0.347	0.653	0.0562	0.4961	0.126	0.874	0.0184	0.4948	0.124	0.876	0.0179	0.4961
	0.6	0.4980	0.5020	0.0562	0.4029	0.513	0.487	0.0568	0.4053	0.199	0.801	0.0188	0.4029	0.195	0.805	0.0187	0.4053
	0.7	0.7020	0.2980	0.0580	0.3058	0.718	0.282	0.0580	0.3083	0.258	0.742	0.0197	0.3058	0.273	0.727	0.0195	0.3083
	0.8	0.8880	0.1120	0.0546	0.2085	0.896	0.104	0.0545	0.2108	0.469	0.531	0.0183	0.2085	0.501	0.499	0.0181	0.2108
	0.85	0.9460	0.0540	0.0547	0.1619	0.954	0.046	0.0544	0.1641	0.708	0.292	0.0187	0.1619	0.729	0.271	0.0188	0.1641
	0.9	0.9770	0.0230	0.0514	0.1122	0.979	0.021	0.0514	0.1139	0.917	0.083	0.0175	0.1122	0.92	0.08	0.0175	0.1139
	0.95	0.9910	0.0090	0.0512	0.0660	0.993	0.007	0.0513	0.0670	0.98	0.02	0.0155	0.0660	0.984	0.016	0.0155	0.0670
	0.99	0.9900	0.0100	0.0525	0.0278	0.999	0.001	0.0503	0.0283	0.986	0.014	0.0118	0.0278	0.997	0.003	0.0116	0.0283
	0.1	0.0640	0.9360	0.0542	0.8524	0.0610	0.9390	0.0549	0.8286	0.0300	0.9700	0.0222	0.8524	0.0260	0.9740	0.0216	0.8286
	0.2	0.1050	0.8950	0.0546	0.7764	0.1270	0.8730	0.0556	0.7660	0.0440	0.9560	0.0200	0.7764	0.0520	0.9480	0.0210	0.7660
	0.3	0.1690	0.8310	0.0535	0.6890	0.1740	0.8260	0.0541	0.6843	0.0520	0.9480	0.0174	0.6890	0.0540	0.9460	0.0177	0.6843
	0.4	0.2610	0.7390	0.0555	0.5934	0.2800	0.7200	0.0563	0.5914	0.0950	0.9050	0.0180	0.5934	0.0900	0.9100	0.0180	0.5914
**0.90**	0.5	0.3610	0.6390	0.0563	0.4947	0.3580	0.6420	0.0561	0.4957	0.1310	0.8690	0.0186	0.4947	0.1250	0.8750	0.0181	0.4957
	0.6	0.5560	0.4440	0.0561	0.4012	0.5620	0.4380	0.0563	0.4034	0.2080	0.7920	0.0191	0.4012	0.2130	0.7870	0.0184	0.4034
	0.7	0.7360	0.2640	0.0565	0.3050	0.7380	0.2620	0.0564	0.3075	0.3460	0.6540	0.0189	0.3050	0.3590	0.6410	0.0189	0.3075
	0.8	0.8960	0.1040	0.0540	0.2074	0.9030	0.0970	0.0537	0.2099	0.6150	0.3850	0.0183	0.2074	0.6350	0.3650	0.0185	0.2099
	0.85	0.9520	0.0480	0.0543	0.1609	0.9510	0.0490	0.0542	0.1631	0.8090	0.1910	0.0181	0.1609	0.8200	0.1800	0.0177	0.1631
	0.9	0.9660	0.0340	0.0519	0.1133	0.9770	0.0230	0.0522	0.1151	0.9030	0.0970	0.0181	0.1133	0.9110	0.0890	0.0180	0.1151
	0.95	0.9880	0.0120	0.0518	0.0673	0.9920	0.0080	0.0521	0.0682	0.9770	0.0230	0.0159	0.0673	0.9810	0.0190	0.0159	0.0682
	0.99	0.9870	0.0130	0.0526	0.0286	0.9960	0.0040	0.0509	0.0291	0.9860	0.0140	0.0119	0.0286	0.9960	0.0040	0.0119	0.0291

Table 2. Simulation results for n=100, %5 percentage of contamination

		**q=0.95**								**q=0.99**							
		NW				LL				NW				LL			
γ	ρ	TDR	M	S	MCE	TDR	M	S	MCE	TDR	M	S	MCE	TDR	M	S	MCE
**0.10**	0.1	0.0570	0.9430	0.0561	0.8474	0.0566	0.9434	0.0563	0.8304	0.0214	0.9786	0.0235	0.8474	0.0214	0.9786	0.0229	0.8304
	0.2	0.0604	0.9396	0.0576	0.7725	0.0660	0.9340	0.0591	0.7598	0.0250	0.9750	0.0227	0.7725	0.0260	0.9740	0.0232	0.7598
	0.3	0.0532	0.9468	0.0563	0.6813	0.0584	0.9416	0.0572	0.6754	0.0194	0.9806	0.0194	0.6813	0.0190	0.9810	0.0200	0.6754
	0.4	0.0574	0.9426	0.0582	0.5826	0.0570	0.9430	0.0584	0.5800	0.0182	0.9818	0.0207	0.5826	0.0218	0.9782	0.0204	0.5800
	0.5	0.0574	0.9426	0.0569	0.4890	0.0570	0.9430	0.0574	0.4897	0.0202	0.9798	0.0191	0.4890	0.0202	0.9798	0.0196	0.4897
	0.6	0.0580	0.9420	0.0569	0.3887	0.0604	0.9396	0.0567	0.3911	0.0216	0.9784	0.0183	0.3887	0.0190	0.9810	0.0183	0.3911
	0.7	0.0544	0.9456	0.0564	0.2934	0.0548	0.9452	0.0565	0.2959	0.0182	0.9818	0.0189	0.2934	0.0186	0.9814	0.0191	0.2959
	0.8	0.0600	0.9400	0.0532	0.1933	0.0612	0.9388	0.0531	0.1960	0.0222	0.9778	0.0178	0.1933	0.0224	0.9776	0.0180	0.1960
	0.85	0.0536	0.9464	0.0547	0.1472	0.0540	0.9460	0.0550	0.1493	0.0150	0.9850	0.0183	0.1472	0.0144	0.9856	0.0183	0.1493
	0.9	0.0594	0.9406	0.0510	0.0978	0.0604	0.9396	0.0514	0.0994	0.0184	0.9816	0.0177	0.0978	0.0186	0.9814	0.0177	0.0994
	0.95	0.0626	0.9374	0.0508	0.0495	0.0652	0.9348	0.0509	0.0504	0.0146	0.9854	0.0153	0.0495	0.0148	0.9852	0.0154	0.0504
	0.99	0.9568	0.0432	0.0516	0.0119	0.9752	0.0248	0.0516	0.0123	0.0156	0.9844	0.0119	0.0119	0.0164	0.9836	0.0121	0.0123
**0.20**	0.1	0.0556	0.9444	0.0543	0.8490	0.0598	0.9402	0.0551	0.8311	0.0266	0.9734	0.0225	0.8490	0.0262	0.9738	0.0226	0.8311
	0.2	0.0568	0.9432	0.0562	0.7744	0.0584	0.9416	0.0575	0.7645	0.0202	0.9798	0.0222	0.7744	0.0208	0.9792	0.0220	0.7645
	0.3	0.0640	0.9360	0.0547	0.6844	0.0640	0.9360	0.0553	0.6796	0.0234	0.9766	0.0176	0.6844	0.0222	0.9778	0.0186	0.6796
	0.4	0.0580	0.9420	0.0562	0.5912	0.0570	0.9430	0.0567	0.5888	0.0190	0.9810	0.0185	0.5912	0.0196	0.9804	0.0190	0.5888
	0.5	0.0564	0.9436	0.0570	0.4924	0.0572	0.9428	0.0570	0.4939	0.0204	0.9796	0.0190	0.4924	0.0190	0.9810	0.0191	0.4939
	0.6	0.0658	0.9342	0.0567	0.3954	0.0656	0.9344	0.0565	0.3983	0.0230	0.9770	0.0187	0.3954	0.0246	0.9754	0.0184	0.3983
	0.7	0.0630	0.9370	0.0564	0.2973	0.0644	0.9356	0.0563	0.3002	0.0244	0.9756	0.0187	0.2973	0.0262	0.9738	0.0188	0.3002
	0.8	0.0688	0.9312	0.0549	0.2041	0.0706	0.9294	0.0550	0.2066	0.0224	0.9776	0.0191	0.2041	0.0220	0.9780	0.0192	0.2066
	0.85	0.0632	0.9368	0.0530	0.1528	0.0636	0.9364	0.0531	0.1550	0.0202	0.9798	0.0180	0.1528	0.0200	0.9800	0.0179	0.1550
	0.9	0.0704	0.9296	0.0508	0.1027	0.0722	0.9278	0.0512	0.1047	0.0216	0.9784	0.0168	0.1027	0.0224	0.9776	0.0171	0.1047
	0.95	0.4152	0.5848	0.0504	0.0563	0.4824	0.5176	0.0505	0.0574	0.0180	0.9820	0.0154	0.0563	0.0186	0.9814	0.0157	0.0574
	0.99	0.9840	0.0160	0.0608	0.0175	0.9914	0.0086	0.0564	0.0179	0.8372	0.1628	0.0111	0.0175	0.8950	0.1050	0.0111	0.0179

Table 2. (continued)

		q=0.95								q=0.99							
		NW				LL				NW				LL			
γ	ρ	TDR	M	S	MCE	TDR	M	S	MCE	TDR	M	S	MCE	TDR	M	S	MCE
**0.30**	0.1	0.0564	0.9436	0.0528	0.8528	0.0576	0.9424	0.0550	0.8324	0.0208	0.9792	0.0220	0.8528	0.0220	0.9780	0.0224	0.8324
	0.2	0.0682	0.9318	0.0534	0.7787	0.0636	0.9364	0.0571	0.7652	0.0262	0.9738	0.0200	0.7787	0.0286	0.9714	0.0214	0.7652
	0.3	0.0642	0.9358	0.0542	0.6859	0.0670	0.9330	0.0551	0.6804	0.0190	0.9810	0.0182	0.6859	0.0222	0.9778	0.0180	0.6804
	0.4	0.0712	0.9288	0.0547	0.5906	0.0736	0.9264	0.0557	0.5884	0.0228	0.9772	0.0174	0.5906	0.0216	0.9784	0.0179	0.5884
	0.5	0.0720	0.9280	0.0573	0.4975	0.0722	0.9278	0.0575	0.4985	0.0248	0.9752	0.0173	0.4975	0.0242	0.9758	0.0178	0.4985
	0.6	0.0760	0.9240	0.0558	0.4025	0.0760	0.9240	0.0558	0.4040	0.0266	0.9734	0.0175	0.4025	0.0254	0.9746	0.0176	0.4040
	0.7	0.0756	0.9244	0.0561	0.3056	0.0768	0.9232	0.0561	0.3086	0.0236	0.9764	0.0175	0.3056	0.0260	0.9740	0.0178	0.3086
	0.8	0.0786	0.9214	0.0532	0.2091	0.0802	0.9198	0.0532	0.2119	0.0202	0.9798	0.0180	0.2091	0.0206	0.9794	0.0179	0.2119
	0.85	0.0902	0.9098	0.0529	0.1603	0.0924	0.9076	0.0533	0.1629	0.0214	0.9786	0.0174	0.1603	0.0222	0.9778	0.0174	0.1629
	0.9	0.1612	0.8388	0.0506	0.1128	0.1660	0.8340	0.0509	0.1145	0.0228	0.9772	0.0172	0.1128	0.0232	0.9768	0.0172	0.1145
	0.95	0.9142	0.0858	0.0494	0.0654	0.9318	0.0682	0.0501	0.0666	0.0208	0.9792	0.0156	0.0654	0.0218	0.9782	0.0157	0.0666
	0.99	0.9916	0.0084	0.0749	0.0282	0.9982	0.0018	0.0665	0.0289	0.9734	0.0266	0.0111	0.0282	0.9860	0.0140	0.0112	0.0289
**0.40**	0.1	0.0584	0.9416	0.0523	0.8551	0.0590	0.9410	0.0524	0.8344	0.0238	0.9762	0.0208	0.8551	0.0274	0.9726	0.0211	0.8344
	0.2	0.0682	0.9318	0.0541	0.7799	0.0708	0.9292	0.0568	0.7666	0.0248	0.9752	0.0198	0.7799	0.0258	0.9742	0.0220	0.7666
	0.3	0.0764	0.9236	0.0527	0.6914	0.0820	0.9180	0.0532	0.6849	0.0250	0.9750	0.0162	0.6914	0.0230	0.9770	0.0176	0.6849
	0.4	0.0758	0.9242	0.0547	0.5952	0.0782	0.9218	0.0547	0.5934	0.0254	0.9746	0.0169	0.5952	0.0250	0.9750	0.0169	0.5934
	0.5	0.0776	0.9224	0.0533	0.5021	0.0756	0.9244	0.0541	0.5024	0.0224	0.9776	0.0165	0.5021	0.0210	0.9790	0.0162	0.5024
	0.6	0.0810	0.9190	0.0552	0.4110	0.0820	0.9180	0.0554	0.4134	0.0232	0.9768	0.0166	0.4110	0.0248	0.9752	0.0169	0.4134
	0.7	0.0956	0.9044	0.0552	0.3143	0.0946	0.9054	0.0552	0.3170	0.0278	0.9722	0.0171	0.3143	0.0292	0.9708	0.0170	0.3170
	0.8	0.1128	0.8872	0.0543	0.2192	0.1160	0.8840	0.0544	0.2222	0.0286	0.9714	0.0178	0.2192	0.0298	0.9702	0.0182	0.2222
	0.85	0.1638	0.8362	0.0511	0.1693	0.1682	0.8318	0.0516	0.1716	0.0296	0.9704	0.0171	0.1693	0.0298	0.9702	0.0174	0.1716
	0.9	0.6190	0.3810	0.0517	0.1261	0.6714	0.3286	0.0520	0.1282	0.0344	0.9656	0.0178	0.1261	0.0354	0.9646	0.0177	0.1282
	0.95	0.9622	0.0378	0.0502	0.0786	0.9736	0.0264	0.0506	0.0802	0.0426	0.9574	0.0156	0.0786	0.0446	0.9554	0.0159	0.0802
	0.99	0.9922	0.0078	0.0839	0.0410	0.9980	0.0020	0.0732	0.0421	0.9834	0.0166	0.0125	0.0410	0.9946	0.0054	0.0124	0.0421

Table 2. (continued)

		q=0.95								q=0.99							
		NW				LL				NW				LL			
γ	ρ	TDR	M	S	MCE	TDR	M	S	MCE	TDR	M	S	MCE	TDR	M	S	MCE
**0.50**	0.1	0.0612	0.9388	0.0524	0.8501	0.0622	0.9378	0.0549	0.8309	0.0262	0.9738	0.0227	0.8501	0.0232	0.9768	0.0217	0.8309
	0.2	0.0694	0.9306	0.0529	0.7812	0.0738	0.9262	0.0542	0.7715	0.0250	0.9750	0.0202	0.7812	0.0274	0.9726	0.0201	0.7715
	0.3	0.0754	0.9246	0.0506	0.6949	0.0802	0.9198	0.0513	0.6896	0.0232	0.9768	0.0150	0.6949	0.0258	0.9742	0.0157	0.6896
	0.4	0.0884	0.9116	0.0541	0.6009	0.0896	0.9104	0.0536	0.5996	0.0268	0.9732	0.0168	0.6009	0.0272	0.9728	0.0164	0.5996
	0.5	0.0970	0.9030	0.0528	0.5089	0.1022	0.8978	0.0531	0.5092	0.0308	0.9692	0.0158	0.5089	0.0336	0.9664	0.0156	0.5092
	0.6	0.1102	0.8898	0.0543	0.4203	0.1114	0.8886	0.0550	0.4220	0.0268	0.9732	0.0169	0.4203	0.0300	0.9700	0.0167	0.4220
	0.7	0.1398	0.8602	0.0551	0.3268	0.1420	0.8580	0.0549	0.3294	0.0364	0.9636	0.0171	0.3268	0.0388	0.9612	0.0168	0.3294
	0.8	0.2044	0.7956	0.0534	0.2324	0.2118	0.7882	0.0535	0.2352	0.0298	0.9702	0.0177	0.2324	0.0302	0.9698	0.0181	0.2352
	0.85	0.4824	0.5176	0.0531	0.1843	0.5128	0.4872	0.0533	0.1870	0.0360	0.9640	0.0179	0.1843	0.0382	0.9618	0.0179	0.1870
	0.9	0.8986	0.1014	0.0530	0.1417	0.9116	0.0884	0.0534	0.1435	0.0432	0.9568	0.0181	0.1417	0.0442	0.9558	0.0183	0.1435
	0.95	0.9794	0.0206	0.0478	0.0905	0.9856	0.0144	0.0483	0.0922	0.2236	0.7764	0.0144	0.0905	0.2538	0.7462	0.0145	0.0922
	0.99	0.9944	0.0056	0.0908	0.0553	0.9980	0.0020	0.0770	0.0567	0.9856	0.0144	0.0120	0.0553	0.9964	0.0036	0.0117	0.0567
**0.60**	0.1	0.0650	0.9350	0.0524	0.8545	0.0648	0.9352	0.0534	0.8355	0.0282	0.9718	0.0219	0.8545	0.0266	0.9734	0.0201	0.8355
	0.2	0.0812	0.9188	0.0532	0.7792	0.0866	0.9134	0.0546	0.7693	0.0316	0.9684	0.0198	0.7792	0.0352	0.9648	0.0198	0.7693
	0.3	0.0978	0.9022	0.0504	0.6987	0.1006	0.8994	0.0513	0.6918	0.0326	0.9674	0.0156	0.6987	0.0308	0.9692	0.0163	0.6918
	0.4	0.1116	0.8884	0.0514	0.6086	0.1126	0.8874	0.0519	0.6055	0.0336	0.9664	0.0150	0.6086	0.0372	0.9628	0.0153	0.6055
	0.5	0.1288	0.8712	0.0529	0.5149	0.1314	0.8686	0.0528	0.5156	0.0368	0.9632	0.0168	0.5149	0.0396	0.9604	0.0166	0.5156
	0.6	0.1572	0.8428	0.0548	0.4272	0.1608	0.8392	0.0542	0.4286	0.0440	0.9560	0.0162	0.4272	0.0440	0.9560	0.0159	0.4286
	0.7	0.2052	0.7948	0.0537	0.3374	0.2078	0.7922	0.0538	0.3399	0.0424	0.9576	0.0170	0.3374	0.0420	0.9580	0.0173	0.3399
	0.8	0.4810	0.5190	0.0537	0.2443	0.5034	0.4966	0.0540	0.2468	0.0562	0.9438	0.0178	0.2443	0.0574	0.9426	0.0178	0.2468
	0.85	0.8060	0.1940	0.0525	0.1971	0.8294	0.1706	0.0524	0.1997	0.0628	0.9372	0.0172	0.1971	0.0638	0.9362	0.0174	0.1997
	0.9	0.9402	0.0598	0.0503	0.1510	0.9504	0.0496	0.0503	0.1531	0.0886	0.9114	0.0169	0.1510	0.0940	0.9060	0.0169	0.1531
	0.95	0.9876	0.0124	0.0504	0.1088	0.9916	0.0084	0.0506	0.1103	0.8744	0.1256	0.0160	0.1088	0.8962	0.1038	0.0161	0.1103
	0.99	0.9956	0.0044	0.0933	0.0715	0.9988	0.0012	0.0785	0.0727	0.9920	0.0080	0.0126	0.0715	0.9966	0.0034	0.0123	0.0727

Table 2. (continued)

		q=0.95								q=0.99							
		NW				LL				NW				LL			
γ	ρ	TDR	M	S	MCE	TDR	M	S	MCE	TDR	M	S	MCE	TDR	M	S	MCE
**0.70**	0.1	0.0646	0.9354	0.0534	0.8559	0.0656	0.9344	0.0514	0.8378	0.0258	0.9742	0.0222	0.8559	0.0252	0.9748	0.0203	0.8378
	0.2	0.0936	0.9064	0.0525	0.7867	0.0884	0.9116	0.0540	0.7764	0.0316	0.9684	0.0189	0.7867	0.0320	0.9680	0.0186	0.7764
	0.3	0.1108	0.8892	0.0483	0.7043	0.1168	0.8832	0.0501	0.6983	0.0326	0.9674	0.0137	0.7043	0.0336	0.9664	0.0147	0.6983
	0.4	0.1554	0.8446	0.0520	0.6185	0.1578	0.8422	0.0523	0.6158	0.0488	0.9512	0.0143	0.6185	0.0480	0.9520	0.0146	0.6158
	0.5	0.1896	0.8104	0.0532	0.5273	0.1878	0.8122	0.0529	0.5271	0.0538	0.9462	0.0152	0.5273	0.0554	0.9446	0.0155	0.5271
	0.6	0.2468	0.7532	0.0533	0.4364	0.2534	0.7466	0.0526	0.4377	0.0692	0.9308	0.0151	0.4364	0.0702	0.9298	0.0156	0.4377
	0.7	0.4096	0.5904	0.0539	0.3400	0.4190	0.5810	0.0537	0.3428	0.0794	0.9206	0.0173	0.3400	0.0838	0.9162	0.0177	0.3428
	0.8	0.7572	0.2428	0.0536	0.2568	0.7774	0.2226	0.0538	0.2598	0.0990	0.9010	0.0178	0.2568	0.0998	0.9002	0.0181	0.2598
	0.85	0.9016	0.0984	0.0523	0.2103	0.9110	0.0890	0.0523	0.2127	0.1464	0.8536	0.0172	0.2103	0.1500	0.8500	0.0171	0.2127
	0.9	0.9628	0.0372	0.0510	0.1659	0.9696	0.0304	0.0513	0.1682	0.4554	0.5446	0.0179	0.1659	0.4926	0.5074	0.0178	0.1682
	0.95	0.9860	0.0140	0.0506	0.1216	0.9916	0.0084	0.0509	0.1233	0.9508	0.0492	0.0157	0.1216	0.9616	0.0384	0.0158	0.1233
	0.99	0.9940	0.0060	0.0843	0.0857	0.9996	0.0004	0.0748	0.0869	0.9900	0.0100	0.0116	0.0857	0.9978	0.0022	0.0115	0.0869
**0.80**	0.1	0.0662	0.9338	0.0499	0.8547	0.0634	0.9366	0.0523	0.8356	0.0254	0.9746	0.0205	0.8547	0.0244	0.9756	0.0198	0.8356
	0.2	0.0968	0.9032	0.0509	0.7864	0.0998	0.9002	0.0522	0.7737	0.0344	0.9656	0.0181	0.7864	0.0342	0.9658	0.0185	0.7737
	0.3	0.1232	0.8768	0.0480	0.7083	0.1268	0.8732	0.0483	0.7018	0.0362	0.9638	0.0137	0.7083	0.0418	0.9582	0.0145	0.7018
	0.4	0.1754	0.8246	0.0506	0.6175	0.1800	0.8200	0.0508	0.6152	0.0548	0.9452	0.0141	0.6175	0.0558	0.9442	0.0144	0.6152
	0.5	0.2690	0.7310	0.0514	0.5289	0.2798	0.7202	0.0517	0.5292	0.0824	0.9176	0.0151	0.5289	0.0832	0.9168	0.0150	0.5292
	0.6	0.4076	0.5924	0.0534	0.4413	0.4136	0.5864	0.0536	0.4434	0.1116	0.8884	0.0162	0.4413	0.1178	0.8822	0.0164	0.4434
	0.7	0.6054	0.3946	0.0548	0.3538	0.6242	0.3758	0.0549	0.3564	0.1566	0.8434	0.0178	0.3538	0.1622	0.8378	0.0179	0.3564
	0.8	0.8522	0.1478	0.0525	0.2628	0.8662	0.1338	0.0529	0.2659	0.2704	0.7296	0.0175	0.2628	0.2816	0.7184	0.0179	0.2659
	0.85	0.9278	0.0722	0.0526	0.2198	0.9302	0.0698	0.0529	0.2221	0.4990	0.5010	0.0177	0.2198	0.5292	0.4708	0.0176	0.2221
	0.9	0.9652	0.0348	0.0517	0.1768	0.9704	0.0296	0.0518	0.1789	0.8552	0.1448	0.0176	0.1768	0.8706	0.1294	0.0175	0.1789
	0.95	0.9892	0.0108	0.0499	0.1321	0.9932	0.0068	0.0498	0.1335	0.9724	0.0276	0.0151	0.1321	0.9794	0.0206	0.0153	0.1335
	0.99	0.9928	0.0072	0.0754	0.0964	0.9988	0.0012	0.0662	0.0978	0.9898	0.0102	0.0116	0.0964	0.9980	0.0020	0.0112	0.0978

Table 2. (continued)

		q=0.95								q=0.99							
		NW				LL				NW				LL			
γ	ρ	TDR	M	S	MCE	TDR	M	S	MCE	TDR	M	S	MCE	TDR	M	S	MCE
**0.85**	0.1	0.0728	0.9272	0.0535	0.8571	0.0706	0.9294	0.0544	0.8366	0.0264	0.9736	0.0231	0.8571	0.0290	0.9710	0.0223	0.8366
	0.2	0.0970	0.9030	0.0510	0.7879	0.1002	0.8998	0.0525	0.7747	0.0352	0.9648	0.0182	0.7879	0.0362	0.9638	0.0185	0.7747
	0.3	0.1438	0.8562	0.0482	0.7095	0.1454	0.8546	0.0490	0.7027	0.0428	0.9572	0.0138	0.7095	0.0420	0.9580	0.0143	0.7027
	0.4	0.2198	0.7802	0.0497	0.6224	0.2234	0.7766	0.0501	0.6206	0.0664	0.9336	0.0147	0.6224	0.0660	0.9340	0.0143	0.6206
	0.5	0.3182	0.6818	0.0520	0.5349	0.3216	0.6784	0.0523	0.5354	0.0976	0.9024	0.0149	0.5349	0.0960	0.9040	0.0150	0.5354
	0.6	0.4646	0.5354	0.0540	0.4453	0.4724	0.5276	0.0541	0.4463	0.1512	0.8488	0.0166	0.4453	0.1516	0.8484	0.0160	0.4463
	0.7	0.6880	0.3120	0.0550	0.3584	0.7048	0.2952	0.0547	0.3607	0.2424	0.7576	0.0174	0.3584	0.2506	0.7494	0.0179	0.3607
	0.8	0.8748	0.1252	0.0537	0.2675	0.8798	0.1202	0.0539	0.2700	0.4296	0.5704	0.0180	0.2675	0.4518	0.5482	0.0179	0.2700
	0.85	0.9288	0.0712	0.0528	0.2231	0.9358	0.0642	0.0529	0.2257	0.6520	0.3480	0.0175	0.2231	0.6816	0.3184	0.0176	0.2257
	0.9	0.9734	0.0266	0.0520	0.1809	0.9776	0.0224	0.0520	0.1829	0.9000	0.1000	0.0176	0.1809	0.9106	0.0894	0.0175	0.1829
	0.95	0.9908	0.0092	0.0502	0.1366	0.9926	0.0074	0.0503	0.1379	0.9718	0.0282	0.0155	0.1366	0.9764	0.0236	0.0156	0.1379
	0.99	0.9940	0.0060	0.0691	0.1010	0.9984	0.0016	0.0616	0.1021	0.9916	0.0084	0.0115	0.1010	0.9976	0.0024	0.0115	0.1021
**0.90**	0.1	0.0748	0.9252	0.0524	0.8537	0.0682	0.9318	0.0539	0.8350	0.0324	0.9676	0.0220	0.8537	0.0256	0.9744	0.0213	0.8350
	0.2	0.1118	0.8882	0.0511	0.7912	0.1146	0.8854	0.0524	0.7781	0.0402	0.9598	0.0177	0.7912	0.0418	0.9582	0.0179	0.7781
	0.3	0.1496	0.8504	0.0503	0.7058	0.1528	0.8472	0.0494	0.6994	0.0512	0.9488	0.0153	0.7058	0.0548	0.9452	0.0154	0.6994
	0.4	0.2310	0.7690	0.0520	0.6249	0.2284	0.7716	0.0525	0.6221	0.0718	0.9282	0.0144	0.6249	0.0678	0.9322	0.0149	0.6221
	0.5	0.3416	0.6584	0.0528	0.5386	0.3454	0.6546	0.0536	0.5390	0.1084	0.8916	0.0147	0.5386	0.1082	0.8918	0.0148	0.5390
	0.6	0.5216	0.4784	0.0536	0.4476	0.5292	0.4708	0.0535	0.4492	0.1820	0.8180	0.0158	0.4476	0.1818	0.8182	0.0160	0.4492
	0.7	0.7322	0.2678	0.0542	0.3558	0.7396	0.2604	0.0539	0.3585	0.3220	0.6780	0.0176	0.3558	0.3296	0.6704	0.0174	0.3585
	0.8	0.8830	0.1170	0.0526	0.2683	0.8904	0.1096	0.0526	0.2710	0.5864	0.4136	0.0169	0.2683	0.6072	0.3928	0.0170	0.2710
	0.85	0.9416	0.0584	0.0524	0.2256	0.9458	0.0542	0.0522	0.2280	0.7826	0.2174	0.0177	0.2256	0.7962	0.2038	0.0177	0.2280
	0.9	0.9740	0.0260	0.0511	0.1833	0.9776	0.0224	0.0513	0.1850	0.9124	0.0876	0.0172	0.1833	0.9198	0.0802	0.0172	0.1850
	0.95	0.9872	0.0128	0.0510	0.1394	0.9910	0.0090	0.0509	0.1407	0.9744	0.0256	0.0152	0.1394	0.9784	0.0216	0.0152	0.1407
	0.99	0.9926	0.0074	0.0632	0.1047	0.9984	0.0016	0.0577	0.1059	0.9890	0.0110	0.0116	0.1047	0.9978	0.0022	0.0115	0.1059

Table 3. Simulation results for n=100, % 10 percentage of contamination

		**q=0.95**								**q=0.99**							
		NW				LL				NW				LL			
γ	ρ	TDR	M	S	MCE	TDR	M	S	MCE	TDR	M	S	MCE	TDR	M	S	MCE
**0.10**	0.1	0.0521	0.9479	0.0549	0.8500	0.0544	0.9456	0.0552	0.8327	0.0207	0.9793	0.0238	0.8500	0.0211	0.9789	0.0222	0.8327
	0.2	0.0598	0.9402	0.0566	0.7716	0.0616	0.9384	0.0578	0.7618	0.0228	0.9772	0.0211	0.7716	0.0222	0.9778	0.0220	0.7618
	0.3	0.0544	0.9456	0.0550	0.6785	0.0520	0.9480	0.0562	0.6758	0.0187	0.9813	0.0197	0.6785	0.0179	0.9821	0.0190	0.6758
	0.4	0.0602	0.9398	0.0570	0.5868	0.0599	0.9401	0.0587	0.5855	0.0214	0.9786	0.0199	0.5868	0.0216	0.9784	0.0197	0.5855
	0.5	0.0607	0.9393	0.0570	0.4908	0.0606	0.9394	0.0570	0.4919	0.0200	0.9800	0.0189	0.4908	0.0199	0.9801	0.0191	0.4919
	0.6	0.0589	0.9411	0.0562	0.3917	0.0597	0.9403	0.0569	0.3942	0.0201	0.9799	0.0188	0.3917	0.0199	0.9801	0.0185	0.3942
	0.7	0.0571	0.9429	0.0561	0.2930	0.0563	0.9437	0.0557	0.2958	0.0179	0.9821	0.0185	0.2930	0.0177	0.9823	0.0186	0.2958
	0.8	0.0543	0.9457	0.0541	0.1959	0.0551	0.9449	0.0538	0.1983	0.0179	0.9821	0.0185	0.1959	0.0185	0.9815	0.0184	0.1983
	0.85	0.0570	0.9430	0.0546	0.1506	0.0575	0.9425	0.0548	0.1527	0.0178	0.9822	0.0184	0.1506	0.0175	0.9825	0.0185	0.1527
	0.9	0.0553	0.9447	0.0514	0.1004	0.0548	0.9452	0.0513	0.1019	0.0167	0.9833	0.0176	0.1004	0.0167	0.9833	0.0174	0.1019
	0.95	0.0614	0.9386	0.0494	0.0518	0.0645	0.9355	0.0499	0.0530	0.0185	0.9815	0.0151	0.0518	0.0186	0.9814	0.0153	0.0530
	0.99	0.9487	0.0513	0.0535	0.0134	0.9709	0.0291	0.0516	0.0137	0.0154	0.9846	0.0114	0.0134	0.0159	0.9841	0.0116	0.0137
**0.20**	0.1	0.0564	0.9436	0.0542	0.8500	0.0537	0.9463	0.0554	0.8272	0.0236	0.9764	0.0226	0.8500	0.0217	0.9783	0.0229	0.8272
	0.2	0.0563	0.9437	0.0560	0.7786	0.0593	0.9407	0.0581	0.7655	0.0202	0.9798	0.0211	0.7786	0.0218	0.9782	0.0227	0.7655
	0.3	0.0567	0.9433	0.0539	0.6856	0.0562	0.9438	0.0547	0.6803	0.0194	0.9806	0.0177	0.6856	0.0177	0.9823	0.0180	0.6803
	0.4	0.0558	0.9442	0.0547	0.5883	0.0550	0.9450	0.0551	0.5875	0.0188	0.9812	0.0182	0.5883	0.0191	0.9809	0.0178	0.5875
	0.5	0.0606	0.9394	0.0554	0.4945	0.0616	0.9384	0.0560	0.4954	0.0192	0.9808	0.0181	0.4945	0.0186	0.9814	0.0172	0.4954
	0.6	0.0617	0.9383	0.0548	0.3995	0.0611	0.9389	0.0553	0.4016	0.0202	0.9798	0.0172	0.3995	0.0197	0.9803	0.0170	0.4016
	0.7	0.0612	0.9388	0.0550	0.3039	0.0596	0.9404	0.0549	0.3068	0.0185	0.9815	0.0167	0.3039	0.0200	0.9800	0.0167	0.3068
	0.8	0.0583	0.9417	0.0533	0.2073	0.0596	0.9404	0.0532	0.2099	0.0194	0.9806	0.0171	0.2073	0.0207	0.9793	0.0173	0.2099
	0.85	0.0661	0.9339	0.0524	0.1597	0.0669	0.9331	0.0522	0.1620	0.0219	0.9781	0.0171	0.1597	0.0215	0.9785	0.0170	0.1620
	0.9	0.0681	0.9319	0.0510	0.1121	0.0696	0.9304	0.0513	0.1139	0.0181	0.9819	0.0179	0.1121	0.0186	0.9814	0.0178	0.1139
	0.95	0.2711	0.7289	0.0483	0.0628	0.3290	0.6710	0.0485	0.0641	0.0166	0.9834	0.0150	0.0628	0.0175	0.9825	0.0151	0.0641
	0.99	0.9836	0.0164	0.0713	0.0258	0.9922	0.0078	0.0639	0.0266	0.5858	0.4142	0.0118	0.0258	0.7253	0.2747	0.0120	0.0266

Table 3. (continued)

		q=0.95								q=0.99							
		NW				LL				NW				LL			
γ	ρ	TDR	M	S	MCE	TDR	M	S	MCE	TDR	M	S	MCE	TDR	M	S	MCE
**0.30**	0.1	0.0562	0.9438	0.0539	0.8510	0.0615	0.9385	0.0531	0.8285	0.0260	0.9740	0.0232	0.8510	0.0249	0.9751	0.0209	0.8285
	0.2	0.0572	0.9428	0.0548	0.7833	0.0583	0.9417	0.0555	0.7705	0.0202	0.9798	0.0205	0.7833	0.0206	0.9794	0.0207	0.7705
	0.3	0.0611	0.9389	0.0539	0.6911	0.0619	0.9381	0.0547	0.6860	0.0203	0.9797	0.0180	0.6911	0.0198	0.9802	0.0184	0.6860
	0.4	0.0597	0.9403	0.0534	0.5996	0.0609	0.9391	0.0530	0.5980	0.0199	0.9801	0.0164	0.5996	0.0179	0.9821	0.0160	0.5980
	0.5	0.0629	0.9371	0.0537	0.5084	0.0638	0.9362	0.0540	0.5084	0.0188	0.9812	0.0161	0.5084	0.0188	0.9812	0.0155	0.5084
	0.6	0.0632	0.9368	0.0523	0.4116	0.0650	0.9350	0.0522	0.4134	0.0170	0.9830	0.0149	0.4116	0.0164	0.9836	0.0150	0.4134
	0.7	0.0652	0.9348	0.0528	0.3148	0.0665	0.9335	0.0526	0.3178	0.0191	0.9809	0.0155	0.3148	0.0188	0.9812	0.0158	0.3178
	0.8	0.0696	0.9304	0.0531	0.2241	0.0708	0.9292	0.0531	0.2267	0.0195	0.9805	0.0170	0.2241	0.0214	0.9786	0.0170	0.2267
	0.85	0.0774	0.9226	0.0517	0.1753	0.0804	0.9196	0.0520	0.1781	0.0200	0.9800	0.0171	0.1753	0.0196	0.9804	0.0171	0.1781
	0.9	0.1233	0.8767	0.0514	0.1309	0.1274	0.8726	0.0514	0.1328	0.0229	0.9771	0.0174	0.1309	0.0228	0.9772	0.0174	0.1328
	0.95	0.8645	0.1355	0.0476	0.0825	0.9004	0.0996	0.0482	0.0842	0.0223	0.9777	0.0152	0.0825	0.0239	0.9761	0.0152	0.0842
	0.99	0.9902	0.0098	0.0947	0.0450	0.9963	0.0037	0.0786	0.0462	0.9628	0.0372	0.0112	0.0450	0.9735	0.0265	0.0111	0.0462
**0.40**	0.1	0.0571	0.9429	0.0524	0.8575	0.0570	0.9430	0.0528	0.8368	0.0249	0.9751	0.0205	0.8575	0.0216	0.9784	0.0203	0.8368
	0.2	0.0636	0.9364	0.0533	0.7842	0.0632	0.9368	0.0543	0.7733	0.0226	0.9774	0.0194	0.7842	0.0230	0.9770	0.0196	0.7733
	0.3	0.0672	0.9328	0.0495	0.7015	0.0651	0.9349	0.0499	0.6957	0.0216	0.9784	0.0143	0.7015	0.0202	0.9798	0.0151	0.6957
	0.4	0.0718	0.9282	0.0521	0.6088	0.0712	0.9288	0.0516	0.6060	0.0198	0.9802	0.0146	0.6088	0.0207	0.9793	0.0143	0.6060
	0.5	0.0741	0.9259	0.0522	0.5214	0.0760	0.9240	0.0519	0.5224	0.0233	0.9767	0.0135	0.5214	0.0231	0.9769	0.0136	0.5224
	0.6	0.0762	0.9238	0.0508	0.4236	0.0783	0.9217	0.0510	0.4259	0.0196	0.9804	0.0147	0.4236	0.0212	0.9788	0.0142	0.4259
	0.7	0.0814	0.9186	0.0526	0.3355	0.0808	0.9192	0.0525	0.3376	0.0225	0.9775	0.0162	0.3355	0.0234	0.9766	0.0163	0.3376
	0.8	0.0952	0.9048	0.0528	0.2440	0.0980	0.9020	0.0530	0.2471	0.0240	0.9760	0.0168	0.2440	0.0254	0.9746	0.0169	0.2471
	0.85	0.1352	0.8648	0.0509	0.1972	0.1420	0.8580	0.0513	0.2001	0.0274	0.9726	0.0166	0.1972	0.0279	0.9721	0.0167	0.2001
	0.9	0.4562	0.5438	0.0500	0.1531	0.5038	0.4962	0.0507	0.1560	0.0287	0.9713	0.0171	0.1531	0.0297	0.9703	0.0172	0.1560
	0.95	0.9561	0.0439	0.0473	0.1062	0.9674	0.0326	0.0476	0.1080	0.0331	0.9669	0.0147	0.1062	0.0355	0.9645	0.0149	0.1080
	0.99	0.9946	0.0054	0.1314	0.0700	0.9984	0.0016	0.0886	0.0714	0.9850	0.0150	0.0115	0.0700	0.9915	0.0085	0.0112	0.0714

Table 3. (continued)

		q=0.95								q=0.99							
		NW				LL				NW				LL			
γ	ρ	TDR	M	S	MCE	TDR	M	S	MCE	TDR	M	S	MCE	TDR	M	S	MCE
**0.50**	0.1	0.0605	0.9395	0.0522	0.8579	0.0638	0.9362	0.0529	0.8362	0.0244	0.9756	0.0207	0.8579	0.0241	0.9759	0.0208	0.8362
	0.2	0.0655	0.9345	0.0511	0.7888	0.0673	0.9327	0.0513	0.7767	0.0239	0.9761	0.0177	0.7888	0.0235	0.9765	0.0171	0.7767
	0.3	0.0742	0.9258	0.0473	0.7061	0.0764	0.9236	0.0483	0.7018	0.0231	0.9769	0.0133	0.7061	0.0248	0.9752	0.0144	0.7018
	0.4	0.0759	0.9241	0.0492	0.6243	0.0779	0.9221	0.0497	0.6226	0.0219	0.9781	0.0127	0.6243	0.0207	0.9793	0.0131	0.6226
	0.5	0.0865	0.9135	0.0505	0.5348	0.0889	0.9111	0.0512	0.5345	0.0223	0.9777	0.0125	0.5348	0.0209	0.9791	0.0129	0.5345
	0.6	0.0941	0.9059	0.0521	0.4464	0.0958	0.9042	0.0522	0.4494	0.0231	0.9769	0.0137	0.4464	0.0239	0.9761	0.0138	0.4494
	0.7	0.1086	0.8914	0.0519	0.3543	0.1079	0.8921	0.0522	0.3566	0.0298	0.9702	0.0148	0.3543	0.0297	0.9703	0.0153	0.3566
	0.8	0.1634	0.8366	0.0514	0.2681	0.1647	0.8353	0.0516	0.2709	0.0308	0.9692	0.0163	0.2681	0.0313	0.9687	0.0164	0.2709
	0.85	0.3590	0.6410	0.0506	0.2219	0.3755	0.6245	0.0506	0.2244	0.0343	0.9657	0.0169	0.2219	0.0342	0.9658	0.0169	0.2244
	0.9	0.8326	0.1674	0.0491	0.1780	0.8610	0.1390	0.0494	0.1803	0.0394	0.9606	0.0163	0.1780	0.0394	0.9606	0.0164	0.1803
	0.95	0.9728	0.0272	0.0480	0.1337	0.9805	0.0195	0.0478	0.1358	0.0990	0.9010	0.0142	0.1337	0.1018	0.8982	0.0145	0.1358
	0.99	0.9958	0.0042	0.1596	0.0998	0.9982	0.0018	0.1022	0.1014	0.9911	0.0089	0.0122	0.0998	0.9947	0.0053	0.0113	0.1014
**0.60**	0.1	0.0595	0.9405	0.0514	0.8558	0.0623	0.9377	0.0519	0.8372	0.0221	0.9779	0.0211	0.8558	0.0229	0.9771	0.0201	0.8372
	0.2	0.0711	0.9289	0.0477	0.7946	0.0754	0.9246	0.0510	0.7804	0.0258	0.9742	0.0160	0.7946	0.0257	0.9743	0.0170	0.7804
	0.3	0.0781	0.9219	0.0449	0.7183	0.0815	0.9185	0.0454	0.7127	0.0215	0.9785	0.0117	0.7183	0.0235	0.9765	0.0124	0.7127
	0.4	0.0998	0.9002	0.0462	0.6336	0.1023	0.8977	0.0468	0.6296	0.0252	0.9748	0.0115	0.6336	0.0252	0.9748	0.0114	0.6296
	0.5	0.1204	0.8796	0.0474	0.5505	0.1229	0.8771	0.0478	0.5500	0.0261	0.9739	0.0112	0.5505	0.0281	0.9719	0.0111	0.5500
	0.6	0.1370	0.8630	0.0512	0.4641	0.1376	0.8624	0.0512	0.4653	0.0315	0.9685	0.0123	0.4641	0.0326	0.9674	0.0127	0.4653
	0.7	0.1780	0.8220	0.0517	0.3773	0.1824	0.8176	0.0515	0.3796	0.0409	0.9591	0.0144	0.3773	0.0415	0.9585	0.0145	0.3796
	0.8	0.3706	0.6294	0.0512	0.2915	0.3933	0.6067	0.0513	0.2948	0.0448	0.9552	0.0165	0.2915	0.0463	0.9537	0.0166	0.2948
	0.85	0.7306	0.2694	0.0510	0.2495	0.7691	0.2309	0.0515	0.2524	0.0487	0.9513	0.0166	0.2495	0.0498	0.9502	0.0171	0.2524
	0.9	0.9237	0.0763	0.0496	0.2070	0.9360	0.0640	0.0498	0.2094	0.0705	0.9295	0.0160	0.2070	0.0723	0.9277	0.0162	0.2094
	0.95	0.9802	0.0198	0.0485	0.1636	0.9862	0.0138	0.0478	0.1657	0.7384	0.2616	0.0145	0.1636	0.7970	0.2030	0.0149	0.1657
	0.99	0.9963	0.0037	0.1624	0.1299	0.9981	0.0019	0.1054	0.1319	0.9904	0.0096	0.0128	0.1299	0.9954	0.0046	0.0113	0.1319

Table 3. (continued)

		**q=0.95**								**q=0.99**							
		NW				LL				NW				LL			
γ	ρ	TDR	M	S	MCE	TDR	M	S	MCE	TDR	M	S	MCE	TDR	M	S	MCE
**0.70**	0.1	0.0615	0.9385	0.0498	0.8581	0.0641	0.9359	0.0516	0.8375	0.0269	0.9731	0.0203	0.8581	0.0266	0.9734	0.0205	0.8375
	0.2	0.0794	0.9206	0.0476	0.7994	0.0789	0.9211	0.0490	0.7868	0.0286	0.9714	0.0160	0.7994	0.0289	0.9711	0.0163	0.7868
	0.3	0.0946	0.9054	0.0433	0.7243	0.1017	0.8983	0.0449	0.7165	0.0270	0.9730	0.0106	0.7243	0.0269	0.9731	0.0113	0.7165
	0.4	0.1230	0.8770	0.0458	0.6407	0.1257	0.8743	0.0457	0.6379	0.0320	0.9680	0.0110	0.6407	0.0320	0.9680	0.0108	0.6379
	0.5	0.1560	0.8440	0.0473	0.5679	0.1613	0.8387	0.0476	0.5674	0.0329	0.9671	0.0102	0.5679	0.0338	0.9662	0.0103	0.5674
	0.6	0.2185	0.7815	0.0497	0.4793	0.2236	0.7764	0.0498	0.4806	0.0512	0.9488	0.0124	0.4793	0.0526	0.9474	0.0128	0.4806
	0.7	0.3312	0.6688	0.0516	0.3990	0.3358	0.6642	0.0515	0.4010	0.0642	0.9358	0.0140	0.3990	0.0660	0.9340	0.0143	0.4010
	0.8	0.6906	0.3094	0.0510	0.3159	0.7147	0.2853	0.0513	0.3186	0.0806	0.9194	0.0165	0.3159	0.0822	0.9178	0.0165	0.3186
	0.85	0.8776	0.1224	0.0514	0.2755	0.8920	0.1080	0.0515	0.2778	0.1064	0.8936	0.0170	0.2755	0.1130	0.8870	0.0170	0.2778
	0.9	0.9559	0.0441	0.0491	0.2337	0.9647	0.0353	0.0496	0.2362	0.3002	0.6998	0.0159	0.2337	0.3245	0.6755	0.0160	0.2362
	0.95	0.9842	0.0158	0.0492	0.1923	0.9898	0.0102	0.0489	0.1947	0.9344	0.0656	0.0149	0.1923	0.9508	0.0492	0.0149	0.1947
	0.99	0.9965	0.0035	0.1379	0.1595	0.9986	0.0014	0.0950	0.1612	0.9919	0.0081	0.0126	0.1595	0.9970	0.0030	0.0118	0.1612
**0.80**	0.1	0.0619	0.9381	0.0490	0.8604	0.0653	0.9347	0.0500	0.8392	0.0265	0.9735	0.0189	0.8604	0.0229	0.9771	0.0197	0.8392
	0.2	0.0883	0.9117	0.0458	0.8048	0.0853	0.9147	0.0478	0.7925	0.0289	0.9711	0.0151	0.8048	0.0300	0.9700	0.0161	0.7925
	0.3	0.1096	0.8904	0.0437	0.7333	0.1152	0.8848	0.0443	0.7253	0.0305	0.9695	0.0113	0.7333	0.0323	0.9677	0.0115	0.7253
	0.4	0.1605	0.8395	0.0452	0.6542	0.1617	0.8383	0.0454	0.6503	0.0401	0.9599	0.0107	0.6542	0.0390	0.9610	0.0107	0.6503
	0.5	0.2337	0.7663	0.0474	0.5727	0.2358	0.7642	0.0475	0.5718	0.0568	0.9432	0.0107	0.5727	0.0624	0.9376	0.0114	0.5718
	0.6	0.3479	0.6521	0.0495	0.4945	0.3579	0.6421	0.0491	0.4956	0.0862	0.9138	0.0121	0.4945	0.0859	0.9141	0.0124	0.4956
	0.7	0.5743	0.4257	0.0506	0.4132	0.5932	0.4068	0.0503	0.4152	0.1325	0.8675	0.0151	0.4132	0.1392	0.8608	0.0150	0.4152
	0.8	0.8335	0.1665	0.0507	0.3341	0.8468	0.1532	0.0510	0.3368	0.2178	0.7822	0.0164	0.3341	0.2302	0.7698	0.0163	0.3368
	0.85	0.9213	0.0787	0.0500	0.2930	0.9290	0.0710	0.0505	0.2955	0.3857	0.6143	0.0163	0.2930	0.4106	0.5894	0.0167	0.2955
	0.9	0.9685	0.0315	0.0494	0.2545	0.9729	0.0271	0.0496	0.2567	0.7923	0.2077	0.0167	0.2545	0.8223	0.1777	0.0169	0.2567
	0.95	0.9890	0.0110	0.0501	0.2166	0.9926	0.0074	0.0502	0.2187	0.9667	0.0333	0.0152	0.2166	0.9753	0.0247	0.0154	0.2187
	0.99	0.9947	0.0053	0.1064	0.1837	0.9979	0.0021	0.0852	0.1855	0.9912	0.0088	0.0133	0.1837	0.9961	0.0039	0.0124	0.1855

Table 3. (continued)

		**q=0.95**								**q=0.99**							
		NW				LL				NW				LL			
γ	ρ	TDR	M	S	MCE	TDR	M	S	MCE	TDR	M	S	MCE	TDR	M	S	MCE
**0.85**	0.1	0.0628	0.9372	0.0492	0.8600	0.0624	0.9376	0.0513	0.8390	0.0267	0.9733	0.0191	0.8600	0.0244	0.9756	0.0195	0.8390
	0.2	0.0889	0.9111	0.0460	0.8015	0.0888	0.9112	0.0480	0.7894	0.0301	0.9699	0.0157	0.8015	0.0294	0.9706	0.0154	0.7894
	0.3	0.1219	0.8781	0.0418	0.7367	0.1261	0.8739	0.0418	0.7304	0.0323	0.9677	0.0107	0.7367	0.0343	0.9657	0.0107	0.7304
	0.4	0.1744	0.8256	0.0444	0.6566	0.1825	0.8175	0.0451	0.6529	0.0397	0.9603	0.0105	0.6566	0.0430	0.9570	0.0111	0.6529
	0.5	0.2801	0.7199	0.0468	0.5821	0.2835	0.7165	0.0470	0.5810	0.0612	0.9388	0.0108	0.5821	0.0654	0.9346	0.0105	0.5810
	0.6	0.4233	0.5767	0.0495	0.5008	0.4331	0.5669	0.0496	0.5011	0.1110	0.8890	0.0125	0.5008	0.1164	0.8836	0.0126	0.5011
	0.7	0.6493	0.3507	0.0528	0.4219	0.6672	0.3328	0.0527	0.4244	0.1898	0.8102	0.0152	0.4219	0.2021	0.7979	0.0156	0.4244
	0.8	0.8711	0.1289	0.0506	0.3424	0.8819	0.1181	0.0513	0.3456	0.3610	0.6390	0.0165	0.3424	0.3815	0.6185	0.0165	0.3456
	0.85	0.9363	0.0637	0.0510	0.3027	0.9419	0.0581	0.0513	0.3053	0.6174	0.3826	0.0163	0.3027	0.6417	0.3583	0.0166	0.3053
	0.9	0.9673	0.0327	0.0506	0.2633	0.9717	0.0283	0.0507	0.2653	0.8730	0.1270	0.0164	0.2633	0.8850	0.1150	0.0163	0.2653
	0.95	0.9880	0.0120	0.0477	0.2235	0.9925	0.0075	0.0478	0.2254	0.9706	0.0294	0.0147	0.2235	0.9773	0.0227	0.0149	0.2254
	0.99	0.9958	0.0042	0.0944	0.1937	0.9985	0.0015	0.0783	0.1951	0.9934	0.0066	0.0125	0.1937	0.9971	0.0029	0.0121	0.1951
**0.90**	0.1	0.0677	0.9323	0.0494	0.8627	0.0666	0.9334	0.0512	0.8430	0.0290	0.9710	0.0194	0.8627	0.0258	0.9742	0.0194	0.8430
	0.2	0.0954	0.9046	0.0448	0.8065	0.0928	0.9072	0.0472	0.7956	0.0344	0.9656	0.0145	0.8065	0.0299	0.9701	0.0146	0.7956
	0.3	0.1241	0.8759	0.0399	0.7369	0.1285	0.8715	0.0409	0.7287	0.0333	0.9667	0.0091	0.7369	0.0343	0.9657	0.0099	0.7287
	0.4	0.1997	0.8003	0.0441	0.6608	0.2049	0.7951	0.0451	0.6570	0.0481	0.9519	0.0098	0.6608	0.0508	0.9492	0.0104	0.6570
	0.5	0.3156	0.6844	0.0465	0.5839	0.3184	0.6816	0.0473	0.5825	0.0837	0.9163	0.0099	0.5839	0.0883	0.9117	0.0106	0.5825
	0.6	0.4829	0.5171	0.0487	0.5042	0.4961	0.5039	0.0492	0.5049	0.1429	0.8571	0.0116	0.5042	0.1464	0.8536	0.0116	0.5049
	0.7	0.7083	0.2917	0.0516	0.4267	0.7157	0.2843	0.0517	0.4287	0.2745	0.7255	0.0147	0.4267	0.2856	0.7144	0.0151	0.4287
	0.8	0.8866	0.1134	0.0532	0.3519	0.8913	0.1087	0.0537	0.3544	0.5518	0.4482	0.0177	0.3519	0.5751	0.4249	0.0173	0.3544
	0.85	0.9400	0.0600	0.0514	0.3083	0.9439	0.0561	0.0515	0.3104	0.7517	0.2483	0.0170	0.3083	0.7704	0.2296	0.0171	0.3104
	0.9	0.9698	0.0302	0.0496	0.2688	0.9746	0.0254	0.0500	0.2710	0.9065	0.0935	0.0167	0.2688	0.9168	0.0832	0.0167	0.2710
	0.95	0.9873	0.0127	0.0482	0.2301	0.9926	0.0074	0.0483	0.2324	0.9720	0.0280	0.0150	0.2301	0.9787	0.0213	0.0152	0.2324
	0.99	0.9935	0.0065	0.0801	0.2003	0.9986	0.0014	0.0696	0.2020	0.9911	0.0089	0.0122	0.2003	0.9973	0.0027	0.0119	0.2020
