# Supplementary material for: A method for detecting outliers in linear-circular non-parametric regression
Source: PLoS One. 2023 Jun 12;18(6):e0286448. doi: 10.1371/journal.pone.0286448 (PMC10259788; doi:10.1371/journal.pone.0286448)
Supplement: S5 File — (PDF) [file pone.0286448.s005.pdf]

**vA Method for Detecting Outliers in Linear-Circular Non-Parametric Regression**

*Sümeyra Sert*[1]* *and* Filiz Kardiyen[2]

[1] *Selcuk University, Department of Statistics, 42250, Selcuklu, Konya, Turkey;*

[1]ORCID id: https://orcid.org/0000-0002-4647-1583

sumeyra.sert@selcuk.edu.tr

[2]*Gazi University, Department of Statistics, Teknikokullar, 06500, Ankara, Turkey.*

[2]ORCID id: https://orcid.org/0000-0002-8730-2751

fyuva@gazi.edu.tr

## Supplementary File (n=200)

Table 1. Simulation results for n=200, % 1 percentage of contamination

| | | **q=0.95** | | | | | | | | **q=0.99** | | | | | | | |
|---|---|---|---|---|---|---|---|---|---|---|---|---|---|---|---|---|---|
| | | NW | | | | LL | | | | NW | | | | LL | | | |
| $\gamma$ | $\rho$ | TDR | M | S | MCE | TDR | M | S | MCE | TDR | M | S | MCE | TDR | M | S | MCE |
| **0.10** | 0.1 | 0.0555 | 0.9445 | 0.0519 | 0.8719 | 0.0545 | 0.9455 | 0.0521 | 0.8641 | 0.0195 | 0.9805 | 0.0187 | 0.8719 | 0.0185 | 0.9815 | 0.0179 | 0.8641 |
| | 0.2 | 0.0610 | 0.9390 | 0.0539 | 0.7844 | 0.0610 | 0.9390 | 0.0544 | 0.7804 | 0.0185 | 0.9815 | 0.0175 | 0.7844 | 0.0225 | 0.9775 | 0.0176 | 0.7804 |
| | 0.3 | 0.0580 | 0.9420 | 0.0515 | 0.6904 | 0.0540 | 0.9460 | 0.0517 | 0.6904 | 0.0125 | 0.9875 | 0.0136 | 0.6904 | 0.0145 | 0.9855 | 0.0134 | 0.6904 |
| | 0.4 | 0.0625 | 0.9375 | 0.0539 | 0.5919 | 0.0605 | 0.9395 | 0.0536 | 0.5938 | 0.0155 | 0.9845 | 0.0151 | 0.5919 | 0.0160 | 0.9840 | 0.0146 | 0.5938 |
| | 0.5 | 0.0605 | 0.9395 | 0.0545 | 0.4899 | 0.0615 | 0.9385 | 0.0543 | 0.4926 | 0.0165 | 0.9835 | 0.0150 | 0.4899 | 0.0160 | 0.9840 | 0.0146 | 0.4926 |
| | 0.6 | 0.0525 | 0.9475 | 0.0529 | 0.3926 | 0.0545 | 0.9455 | 0.0532 | 0.3955 | 0.0170 | 0.9830 | 0.0141 | 0.3926 | 0.0190 | 0.9810 | 0.0143 | 0.3955 |
| | 0.7 | 0.0525 | 0.9475 | 0.0540 | 0.2957 | 0.0535 | 0.9465 | 0.0541 | 0.2981 | 0.0150 | 0.9850 | 0.0145 | 0.2957 | 0.0140 | 0.9860 | 0.0147 | 0.2981 |
| | 0.8 | 0.0510 | 0.9490 | 0.0516 | 0.1943 | 0.0525 | 0.9475 | 0.0517 | 0.1958 | 0.0160 | 0.9840 | 0.0142 | 0.1943 | 0.0135 | 0.9865 | 0.0141 | 0.1958 |
| | 0.85 | 0.0495 | 0.9505 | 0.0517 | 0.1465 | 0.0510 | 0.9490 | 0.0518 | 0.1478 | 0.0150 | 0.9850 | 0.0146 | 0.1465 | 0.0145 | 0.9855 | 0.0144 | 0.1478 |
| | 0.9 | 0.0580 | 0.9420 | 0.0511 | 0.0983 | 0.0595 | 0.9405 | 0.0512 | 0.0993 | 0.0165 | 0.9835 | 0.0139 | 0.0983 | 0.0170 | 0.9830 | 0.0141 | 0.0993 |
| | 0.95 | 0.0775 | 0.9225 | 0.0498 | 0.0487 | 0.0795 | 0.9205 | 0.0497 | 0.0492 | 0.0165 | 0.9835 | 0.0132 | 0.0487 | 0.0175 | 0.9825 | 0.0131 | 0.0492 |
| | 0.99 | 0.9825 | 0.0175 | 0.0498 | 0.0102 | 0.9880 | 0.0120 | 0.0502 | 0.0104 | 0.0160 | 0.9840 | 0.0105 | 0.0102 | 0.0150 | 0.9850 | 0.0107 | 0.0104 |
| **0.20** | 0.1 | 0.0455 | 0.9545 | 0.0506 | 0.8767 | 0.0430 | 0.9570 | 0.0505 | 0.8657 | 0.0180 | 0.9820 | 0.0170 | 0.8767 | 0.0150 | 0.9850 | 0.0161 | 0.8657 |
| | 0.2 | 0.0510 | 0.9490 | 0.0543 | 0.7846 | 0.0535 | 0.9465 | 0.0532 | 0.7828 | 0.0180 | 0.9820 | 0.0177 | 0.7846 | 0.0160 | 0.9840 | 0.0169 | 0.7828 |
| | 0.3 | 0.0625 | 0.9375 | 0.0529 | 0.6859 | 0.0660 | 0.9340 | 0.0527 | 0.6861 | 0.0175 | 0.9825 | 0.0154 | 0.6859 | 0.0225 | 0.9775 | 0.0151 | 0.6861 |
| | 0.4 | 0.0550 | 0.9450 | 0.0524 | 0.5902 | 0.0555 | 0.9445 | 0.0525 | 0.5918 | 0.0150 | 0.9850 | 0.0142 | 0.5902 | 0.0135 | 0.9865 | 0.0141 | 0.5918 |
| | 0.5 | 0.0645 | 0.9355 | 0.0546 | 0.4913 | 0.0670 | 0.9330 | 0.0546 | 0.4935 | 0.0140 | 0.9860 | 0.0145 | 0.4913 | 0.0145 | 0.9855 | 0.0146 | 0.4935 |
| | 0.6 | 0.0480 | 0.9520 | 0.0532 | 0.3929 | 0.0525 | 0.9475 | 0.0529 | 0.3953 | 0.0120 | 0.9880 | 0.0139 | 0.3929 | 0.0100 | 0.9900 | 0.0141 | 0.3953 |
| | 0.7 | 0.0555 | 0.9445 | 0.0530 | 0.2950 | 0.0545 | 0.9455 | 0.0529 | 0.2972 | 0.0175 | 0.9825 | 0.0145 | 0.2950 | 0.0150 | 0.9850 | 0.0143 | 0.2972 |
| | 0.8 | 0.0685 | 0.9315 | 0.0524 | 0.1980 | 0.0700 | 0.9300 | 0.0526 | 0.1997 | 0.0175 | 0.9825 | 0.0146 | 0.1980 | 0.0160 | 0.9840 | 0.0146 | 0.1997 |
| | 0.85 | 0.0550 | 0.9450 | 0.0519 | 0.1481 | 0.0575 | 0.9425 | 0.0521 | 0.1494 | 0.0140 | 0.9860 | 0.0148 | 0.1481 | 0.0140 | 0.9860 | 0.0148 | 0.1494 |
| | 0.9 | 0.0680 | 0.9320 | 0.0508 | 0.0993 | 0.0715 | 0.9285 | 0.0508 | 0.1003 | 0.0125 | 0.9875 | 0.0137 | 0.0993 | 0.0130 | 0.9870 | 0.0138 | 0.1003 |
| | 0.95 | 0.5140 | 0.4860 | 0.0507 | 0.0504 | 0.5890 | 0.4110 | 0.0510 | 0.0510 | 0.0170 | 0.9830 | 0.0131 | 0.0504 | 0.0170 | 0.9830 | 0.0131 | 0.0510 |
| | 0.99 | 0.9905 | 0.0095 | 0.0494 | 0.0113 | 0.9940 | 0.0060 | 0.0493 | 0.0115 | 0.8030 | 0.1970 | 0.0101 | 0.0113 | 0.8685 | 0.1315 | 0.0103 | 0.0115 |

Table 1. (continued)

| | | **q=0.95** | | | | | | | | **q=0.99** | | | | | | | |
|---|---|---|---|---|---|---|---|---|---|---|---|---|---|---|---|---|---|
| | | NW | | | | LL | | | | NW | | | | LL | | | |
| $\gamma$ | $\rho$ | TDR | M | S | MCE | TDR | M | S | MCE | TDR | M | S | MCE | TDR | M | S | MCE |
| **0.30** | 0.1 | 0.0490 | 0.9510 | 0.0507 | 0.8740 | 0.0500 | 0.9500 | 0.0518 | 0.8632 | 0.0155 | 0.9845 | 0.0175 | 0.8740 | 0.0140 | 0.9860 | 0.0169 | 0.8632 |
| | 0.2 | 0.0630 | 0.9370 | 0.0533 | 0.7857 | 0.0605 | 0.9395 | 0.0537 | 0.7821 | 0.0185 | 0.9815 | 0.0175 | 0.7857 | 0.0225 | 0.9775 | 0.0169 | 0.7821 |
| | 0.3 | 0.0660 | 0.9340 | 0.0527 | 0.6894 | 0.0690 | 0.9310 | 0.0536 | 0.6885 | 0.0195 | 0.9805 | 0.0147 | 0.6894 | 0.0165 | 0.9835 | 0.0154 | 0.6885 |
| | 0.4 | 0.0595 | 0.9405 | 0.0536 | 0.5895 | 0.0590 | 0.9410 | 0.0533 | 0.5915 | 0.0180 | 0.9820 | 0.0146 | 0.5895 | 0.0150 | 0.9850 | 0.0144 | 0.5915 |
| | 0.5 | 0.0670 | 0.9330 | 0.0539 | 0.4931 | 0.0655 | 0.9345 | 0.0540 | 0.4956 | 0.0185 | 0.9815 | 0.0146 | 0.4931 | 0.0175 | 0.9825 | 0.0143 | 0.4956 |
| | 0.6 | 0.0710 | 0.9290 | 0.0534 | 0.3964 | 0.0675 | 0.9325 | 0.0533 | 0.3991 | 0.0185 | 0.9815 | 0.0135 | 0.3964 | 0.0175 | 0.9825 | 0.0135 | 0.3991 |
| | 0.7 | 0.0680 | 0.9320 | 0.0527 | 0.2964 | 0.0705 | 0.9295 | 0.0526 | 0.2985 | 0.0135 | 0.9865 | 0.0142 | 0.2964 | 0.0140 | 0.9860 | 0.0142 | 0.2985 |
| | 0.8 | 0.0680 | 0.9320 | 0.0527 | 0.1993 | 0.0665 | 0.9335 | 0.0527 | 0.2009 | 0.0170 | 0.9830 | 0.0143 | 0.1993 | 0.0175 | 0.9825 | 0.0143 | 0.2009 |
| | 0.85 | 0.0860 | 0.9140 | 0.0526 | 0.1508 | 0.0890 | 0.9110 | 0.0528 | 0.1520 | 0.0205 | 0.9795 | 0.0149 | 0.1508 | 0.0200 | 0.9800 | 0.0150 | 0.1520 |
| | 0.9 | 0.1565 | 0.8435 | 0.0507 | 0.1013 | 0.1620 | 0.8380 | 0.0508 | 0.1023 | 0.0160 | 0.9840 | 0.0138 | 0.1013 | 0.0160 | 0.9840 | 0.0138 | 0.1023 |
| | 0.95 | 0.9500 | 0.0500 | 0.0507 | 0.0528 | 0.9560 | 0.0440 | 0.0510 | 0.0534 | 0.0200 | 0.9800 | 0.0134 | 0.0528 | 0.0210 | 0.9790 | 0.0133 | 0.0534 |
| | 0.99 | 0.9955 | 0.0045 | 0.0521 | 0.0137 | 0.9980 | 0.0020 | 0.0512 | 0.0139 | 0.9765 | 0.0235 | 0.0108 | 0.0137 | 0.9850 | 0.0150 | 0.0108 | 0.0139 |
| **0.40** | 0.1 | 0.0645 | 0.9355 | 0.0498 | 0.8753 | 0.0575 | 0.9425 | 0.0512 | 0.8647 | 0.0195 | 0.9805 | 0.0166 | 0.8753 | 0.0200 | 0.9800 | 0.0165 | 0.8647 |
| | 0.2 | 0.0640 | 0.9360 | 0.0523 | 0.7874 | 0.0660 | 0.9340 | 0.0528 | 0.7835 | 0.0225 | 0.9775 | 0.0169 | 0.7874 | 0.0200 | 0.9800 | 0.0162 | 0.7835 |
| | 0.3 | 0.0680 | 0.9320 | 0.0525 | 0.6903 | 0.0750 | 0.9250 | 0.0524 | 0.6896 | 0.0220 | 0.9780 | 0.0147 | 0.6903 | 0.0200 | 0.9800 | 0.0150 | 0.6896 |
| | 0.4 | 0.0735 | 0.9265 | 0.0513 | 0.5922 | 0.0715 | 0.9285 | 0.0521 | 0.5935 | 0.0200 | 0.9800 | 0.0142 | 0.5922 | 0.0155 | 0.9845 | 0.0143 | 0.5935 |
| | 0.5 | 0.0785 | 0.9215 | 0.0540 | 0.4940 | 0.0785 | 0.9215 | 0.0536 | 0.4965 | 0.0220 | 0.9780 | 0.0143 | 0.4940 | 0.0185 | 0.9815 | 0.0146 | 0.4965 |
| | 0.6 | 0.0760 | 0.9240 | 0.0523 | 0.3978 | 0.0760 | 0.9240 | 0.0527 | 0.4004 | 0.0250 | 0.9750 | 0.0134 | 0.3978 | 0.0250 | 0.9750 | 0.0133 | 0.4004 |
| | 0.7 | 0.0855 | 0.9145 | 0.0538 | 0.3004 | 0.0855 | 0.9145 | 0.0536 | 0.3028 | 0.0265 | 0.9735 | 0.0151 | 0.3004 | 0.0250 | 0.9750 | 0.0150 | 0.3028 |
| | 0.8 | 0.1145 | 0.8855 | 0.0520 | 0.2003 | 0.1160 | 0.8840 | 0.0520 | 0.2020 | 0.0210 | 0.9790 | 0.0144 | 0.2003 | 0.0200 | 0.9800 | 0.0144 | 0.2020 |
| | 0.85 | 0.1590 | 0.8410 | 0.0528 | 0.1533 | 0.1610 | 0.8390 | 0.0530 | 0.1547 | 0.0195 | 0.9805 | 0.0147 | 0.1533 | 0.0210 | 0.9790 | 0.0148 | 0.1547 |
| | 0.9 | 0.6930 | 0.3070 | 0.0504 | 0.1032 | 0.7295 | 0.2705 | 0.0504 | 0.1042 | 0.0230 | 0.9770 | 0.0138 | 0.1032 | 0.0230 | 0.9770 | 0.0138 | 0.1042 |
| | 0.95 | 0.9760 | 0.0240 | 0.0502 | 0.0551 | 0.9780 | 0.0220 | 0.0503 | 0.0558 | 0.0315 | 0.9685 | 0.0134 | 0.0551 | 0.0315 | 0.9685 | 0.0134 | 0.0558 |
| | 0.99 | 0.9950 | 0.0050 | 0.0521 | 0.0160 | 0.9990 | 0.0010 | 0.0511 | 0.0163 | 0.9875 | 0.0125 | 0.0106 | 0.0160 | 0.9950 | 0.0050 | 0.0107 | 0.0163 |

Table 1. (continued)

| | | q=0.95 | | | | | | | | q=0.99 | | | | | | | |
|---|---|---|---|---|---|---|---|---|---|---|---|---|---|---|---|---|---|
| | | NW | | | | LL | | | | NW | | | | LL | | | |
| $\gamma$ | $\rho$ | TDR | M | S | MCE | TDR | M | S | MCE | TDR | M | S | MCE | TDR | M | S | MCE |
| **0.50** | 0.1 | 0.0535 | 0.9465 | 0.0518 | 0.8748 | 0.0625 | 0.9375 | 0.0528 | 0.8631 | 0.0185 | 0.9815 | 0.0180 | 0.8748 | 0.0165 | 0.9835 | 0.0183 | 0.8631 |
| | 0.2 | 0.0710 | 0.9290 | 0.0526 | 0.7871 | 0.0755 | 0.9245 | 0.0541 | 0.7836 | 0.0245 | 0.9755 | 0.0173 | 0.7871 | 0.0245 | 0.9755 | 0.0168 | 0.7836 |
| | 0.3 | 0.0845 | 0.9155 | 0.0527 | 0.6904 | 0.0830 | 0.9170 | 0.0526 | 0.6896 | 0.0275 | 0.9725 | 0.0148 | 0.6904 | 0.0215 | 0.9785 | 0.0148 | 0.6896 |
| | 0.4 | 0.0950 | 0.9050 | 0.0514 | 0.5948 | 0.0975 | 0.9025 | 0.0517 | 0.5964 | 0.0235 | 0.9765 | 0.0139 | 0.5948 | 0.0230 | 0.9770 | 0.0140 | 0.5964 |
| | 0.5 | 0.0970 | 0.9030 | 0.0535 | 0.4952 | 0.1040 | 0.8960 | 0.0535 | 0.4979 | 0.0235 | 0.9765 | 0.0143 | 0.4952 | 0.0245 | 0.9755 | 0.0140 | 0.4979 |
| | 0.6 | 0.1040 | 0.8960 | 0.0523 | 0.3991 | 0.1085 | 0.8915 | 0.0523 | 0.4018 | 0.0260 | 0.9740 | 0.0138 | 0.3991 | 0.0260 | 0.9740 | 0.0139 | 0.4018 |
| | 0.7 | 0.1270 | 0.8730 | 0.0524 | 0.3009 | 0.1275 | 0.8725 | 0.0524 | 0.3032 | 0.0310 | 0.9690 | 0.0139 | 0.3009 | 0.0295 | 0.9705 | 0.0139 | 0.3032 |
| | 0.8 | 0.1920 | 0.8080 | 0.0523 | 0.2040 | 0.2030 | 0.7970 | 0.0524 | 0.2056 | 0.0305 | 0.9695 | 0.0146 | 0.2040 | 0.0315 | 0.9685 | 0.0147 | 0.2056 |
| | 0.85 | 0.5030 | 0.4970 | 0.0519 | 0.1552 | 0.5395 | 0.4605 | 0.0521 | 0.1567 | 0.0300 | 0.9700 | 0.0147 | 0.1552 | 0.0285 | 0.9715 | 0.0148 | 0.1567 |
| | 0.9 | 0.9150 | 0.0850 | 0.0508 | 0.1069 | 0.9225 | 0.0775 | 0.0509 | 0.1079 | 0.0365 | 0.9635 | 0.0138 | 0.1069 | 0.0375 | 0.9625 | 0.0138 | 0.1079 |
| | 0.95 | 0.9845 | 0.0155 | 0.0499 | 0.0575 | 0.9870 | 0.0130 | 0.0501 | 0.0582 | 0.0860 | 0.9140 | 0.0130 | 0.0575 | 0.0885 | 0.9115 | 0.0130 | 0.0582 |
| | 0.99 | 0.9945 | 0.0055 | 0.0537 | 0.0189 | 0.9985 | 0.0015 | 0.0513 | 0.0192 | 0.9905 | 0.0095 | 0.0101 | 0.0189 | 0.9965 | 0.0035 | 0.0102 | 0.0192 |
| **0.60** | 0.1 | 0.0715 | 0.9285 | 0.0508 | 0.8761 | 0.0720 | 0.9280 | 0.0517 | 0.8638 | 0.0245 | 0.9755 | 0.0176 | 0.8761 | 0.0235 | 0.9765 | 0.0178 | 0.8638 |
| | 0.2 | 0.0690 | 0.9310 | 0.0524 | 0.7911 | 0.0670 | 0.9330 | 0.0523 | 0.7875 | 0.0245 | 0.9755 | 0.0158 | 0.7911 | 0.0215 | 0.9785 | 0.0153 | 0.7875 |
| | 0.3 | 0.1065 | 0.8935 | 0.0519 | 0.6944 | 0.1030 | 0.8970 | 0.0524 | 0.6933 | 0.0330 | 0.9670 | 0.0139 | 0.6944 | 0.0285 | 0.9715 | 0.0140 | 0.6933 |
| | 0.4 | 0.1005 | 0.8995 | 0.0525 | 0.5960 | 0.1100 | 0.8900 | 0.0522 | 0.5968 | 0.0265 | 0.9735 | 0.0140 | 0.5960 | 0.0265 | 0.9735 | 0.0136 | 0.5968 |
| | 0.5 | 0.1285 | 0.8715 | 0.0531 | 0.4989 | 0.1225 | 0.8775 | 0.0530 | 0.5011 | 0.0295 | 0.9705 | 0.0141 | 0.4989 | 0.0350 | 0.9650 | 0.0142 | 0.5011 |
| | 0.6 | 0.1490 | 0.8510 | 0.0526 | 0.4010 | 0.1560 | 0.8440 | 0.0529 | 0.4033 | 0.0335 | 0.9665 | 0.0137 | 0.4010 | 0.0345 | 0.9655 | 0.0140 | 0.4033 |
| | 0.7 | 0.2150 | 0.7850 | 0.0536 | 0.3037 | 0.2140 | 0.7860 | 0.0533 | 0.3060 | 0.0420 | 0.9580 | 0.0146 | 0.3037 | 0.0435 | 0.9565 | 0.0147 | 0.3060 |
| | 0.8 | 0.4975 | 0.5025 | 0.0522 | 0.2055 | 0.5190 | 0.4810 | 0.0520 | 0.2072 | 0.0465 | 0.9535 | 0.0143 | 0.2055 | 0.0500 | 0.9500 | 0.0142 | 0.2072 |
| | 0.85 | 0.8340 | 0.1660 | 0.0524 | 0.1584 | 0.8480 | 0.1520 | 0.0525 | 0.1597 | 0.0410 | 0.9590 | 0.0146 | 0.1584 | 0.0425 | 0.9575 | 0.0147 | 0.1597 |
| | 0.9 | 0.9520 | 0.0480 | 0.0509 | 0.1090 | 0.9550 | 0.0450 | 0.0512 | 0.1101 | 0.0610 | 0.9390 | 0.0137 | 0.1090 | 0.0645 | 0.9355 | 0.0137 | 0.1101 |
| | 0.95 | 0.9900 | 0.0100 | 0.0498 | 0.0606 | 0.9925 | 0.0075 | 0.0500 | 0.0612 | 0.8035 | 0.1965 | 0.0131 | 0.0606 | 0.8305 | 0.1695 | 0.0131 | 0.0612 |
| | 0.99 | 0.9965 | 0.0035 | 0.0540 | 0.0223 | 0.9990 | 0.0010 | 0.0519 | 0.0226 | 0.9930 | 0.0070 | 0.0106 | 0.0223 | 0.9980 | 0.0020 | 0.0106 | 0.0226 |

Table 1. (continued)

| | | q=0.95 | | | | | | | | q=0.99 | | | | | | | |
|---|---|---|---|---|---|---|---|---|---|---|---|---|---|---|---|---|---|
| | | NW | | | | LL | | | | NW | | | | LL | | | |
| $\gamma$ | $\rho$ | TDR | M | S | MCE | TDR | M | S | MCE | TDR | M | S | MCE | TDR | M | S | MCE |
| **0.70** | 0.1 | 0.0660 | 0.9340 | 0.0511 | 0.8749 | 0.0565 | 0.9435 | 0.0509 | 0.8642 | 0.0210 | 0.9790 | 0.0177 | 0.8749 | 0.0200 | 0.9800 | 0.0169 | 0.8642 |
| | 0.2 | 0.1090 | 0.8910 | 0.0521 | 0.7877 | 0.1050 | 0.8950 | 0.0528 | 0.7842 | 0.0360 | 0.9640 | 0.0167 | 0.7877 | 0.0355 | 0.9645 | 0.0161 | 0.7842 |
| | 0.3 | 0.1160 | 0.8840 | 0.0511 | 0.6940 | 0.1175 | 0.8825 | 0.0502 | 0.6936 | 0.0325 | 0.9675 | 0.0133 | 0.6940 | 0.0305 | 0.9695 | 0.0132 | 0.6936 |
| | 0.4 | 0.1605 | 0.8395 | 0.0528 | 0.5979 | 0.1520 | 0.8480 | 0.0526 | 0.5992 | 0.0440 | 0.9560 | 0.0134 | 0.5979 | 0.0390 | 0.9610 | 0.0139 | 0.5992 |
| | 0.5 | 0.1855 | 0.8145 | 0.0540 | 0.5013 | 0.1930 | 0.8070 | 0.0537 | 0.5037 | 0.0410 | 0.9590 | 0.0143 | 0.5013 | 0.0515 | 0.9485 | 0.0140 | 0.5037 |
| | 0.6 | 0.2355 | 0.7645 | 0.0526 | 0.4014 | 0.2445 | 0.7555 | 0.0527 | 0.4039 | 0.0455 | 0.9545 | 0.0138 | 0.4014 | 0.0530 | 0.9470 | 0.0139 | 0.4039 |
| | 0.7 | 0.4185 | 0.5815 | 0.0529 | 0.3062 | 0.4275 | 0.5725 | 0.0530 | 0.3084 | 0.0740 | 0.9260 | 0.0143 | 0.3062 | 0.0770 | 0.9230 | 0.0143 | 0.3084 |
| | 0.8 | 0.7755 | 0.2245 | 0.0525 | 0.2093 | 0.7865 | 0.2135 | 0.0527 | 0.2109 | 0.0715 | 0.9285 | 0.0147 | 0.2093 | 0.0755 | 0.9245 | 0.0147 | 0.2109 |
| | 0.85 | 0.9065 | 0.0935 | 0.0517 | 0.1595 | 0.9140 | 0.0860 | 0.0517 | 0.1609 | 0.1060 | 0.8940 | 0.0143 | 0.1595 | 0.1080 | 0.8920 | 0.0144 | 0.1609 |
| | 0.9 | 0.9665 | 0.0335 | 0.0504 | 0.1114 | 0.9690 | 0.0310 | 0.0507 | 0.1125 | 0.1875 | 0.8125 | 0.0138 | 0.1114 | 0.1965 | 0.8035 | 0.0139 | 0.1125 |
| | 0.95 | 0.9865 | 0.0135 | 0.0501 | 0.0633 | 0.9905 | 0.0095 | 0.0503 | 0.0639 | 0.9465 | 0.0535 | 0.0131 | 0.0633 | 0.9525 | 0.0475 | 0.0131 | 0.0639 |
| | 0.99 | 0.9935 | 0.0065 | 0.0531 | 0.0252 | 0.9995 | 0.0005 | 0.0518 | 0.0256 | 0.9910 | 0.0090 | 0.0106 | 0.0252 | 0.9975 | 0.0025 | 0.0106 | 0.0256 |
| **0.80** | 0.1 | 0.0775 | 0.9225 | 0.0492 | 0.8752 | 0.0730 | 0.9270 | 0.0514 | 0.8643 | 0.0275 | 0.9725 | 0.0169 | 0.8752 | 0.0250 | 0.9750 | 0.0171 | 0.8643 |
| | 0.2 | 0.1015 | 0.8985 | 0.0525 | 0.7888 | 0.1020 | 0.8980 | 0.0525 | 0.7842 | 0.0395 | 0.9605 | 0.0165 | 0.7888 | 0.0380 | 0.9620 | 0.0160 | 0.7842 |
| | 0.3 | 0.1295 | 0.8705 | 0.0525 | 0.6950 | 0.1330 | 0.8670 | 0.0519 | 0.6943 | 0.0380 | 0.9620 | 0.0142 | 0.6950 | 0.0395 | 0.9605 | 0.0141 | 0.6943 |
| | 0.4 | 0.1875 | 0.8125 | 0.0526 | 0.5962 | 0.1885 | 0.8115 | 0.0525 | 0.5973 | 0.0565 | 0.9435 | 0.0139 | 0.5962 | 0.0565 | 0.9435 | 0.0138 | 0.5973 |
| | 0.5 | 0.2740 | 0.7260 | 0.0536 | 0.5014 | 0.2755 | 0.7245 | 0.0537 | 0.5037 | 0.0705 | 0.9295 | 0.0137 | 0.5014 | 0.0700 | 0.9300 | 0.0136 | 0.5037 |
| | 0.6 | 0.3945 | 0.6055 | 0.0532 | 0.4049 | 0.4050 | 0.5950 | 0.0532 | 0.4076 | 0.0900 | 0.9100 | 0.0138 | 0.4049 | 0.1015 | 0.8985 | 0.0140 | 0.4076 |
| | 0.7 | 0.6355 | 0.3645 | 0.0536 | 0.3092 | 0.6420 | 0.3580 | 0.0535 | 0.3117 | 0.1240 | 0.8760 | 0.0145 | 0.3092 | 0.1245 | 0.8755 | 0.0145 | 0.3117 |
| | 0.8 | 0.8675 | 0.1325 | 0.0522 | 0.2099 | 0.8750 | 0.1250 | 0.0522 | 0.2116 | 0.1970 | 0.8030 | 0.0144 | 0.2099 | 0.2075 | 0.7925 | 0.0146 | 0.2116 |
| | 0.85 | 0.9325 | 0.0675 | 0.0511 | 0.1615 | 0.9360 | 0.0640 | 0.0513 | 0.1628 | 0.3230 | 0.6770 | 0.0144 | 0.1615 | 0.3450 | 0.6550 | 0.0144 | 0.1628 |
| | 0.9 | 0.9695 | 0.0305 | 0.0503 | 0.1131 | 0.9735 | 0.0265 | 0.0505 | 0.1141 | 0.7445 | 0.2555 | 0.0138 | 0.1131 | 0.7725 | 0.2275 | 0.0139 | 0.1141 |
| | 0.95 | 0.9915 | 0.0085 | 0.0500 | 0.0651 | 0.9945 | 0.0055 | 0.0500 | 0.0657 | 0.9675 | 0.0325 | 0.0128 | 0.0651 | 0.9720 | 0.0280 | 0.0128 | 0.0657 |
| | 0.99 | 0.9970 | 0.0030 | 0.0512 | 0.0272 | 0.9985 | 0.0015 | 0.0500 | 0.0275 | 0.9950 | 0.0050 | 0.0103 | 0.0272 | 0.9980 | 0.0020 | 0.0103 | 0.0275 |

Table 1. (continued)

| | | q=0.95 | | | | | | | | q=0.99 | | | | | | | |
|---|---|---|---|---|---|---|---|---|---|---|---|---|---|---|---|---|---|
| | | NW | | | | LL | | | | NW | | | | LL | | | |
| $\gamma$ | $\rho$ | TDR | M | S | MCE | TDR | M | S | MCE | TDR | M | S | MCE | TDR | M | S | MCE |
| **0.85** | 0.1 | 0.0700 | 0.9300 | 0.0500 | 0.8772 | 0.0660 | 0.9340 | 0.0513 | 0.8646 | 0.0245 | 0.9755 | 0.0172 | 0.8772 | 0.0190 | 0.9810 | 0.0171 | 0.8646 |
| | 0.2 | 0.1195 | 0.8805 | 0.0529 | 0.7879 | 0.1175 | 0.8825 | 0.0535 | 0.7838 | 0.0390 | 0.9610 | 0.0169 | 0.7879 | 0.0445 | 0.9555 | 0.0166 | 0.7838 |
| | 0.3 | 0.1515 | 0.8485 | 0.0520 | 0.6944 | 0.1520 | 0.8480 | 0.0526 | 0.6935 | 0.0430 | 0.9570 | 0.0144 | 0.6944 | 0.0405 | 0.9595 | 0.0145 | 0.6935 |
| | 0.4 | 0.2145 | 0.7855 | 0.0519 | 0.5968 | 0.2190 | 0.7810 | 0.0518 | 0.5976 | 0.0525 | 0.9475 | 0.0136 | 0.5968 | 0.0555 | 0.9445 | 0.0134 | 0.5976 |
| | 0.5 | 0.3265 | 0.6735 | 0.0536 | 0.4984 | 0.3330 | 0.6670 | 0.0533 | 0.5009 | 0.1005 | 0.8995 | 0.0141 | 0.4984 | 0.1085 | 0.8915 | 0.0141 | 0.5009 |
| | 0.6 | 0.4585 | 0.5415 | 0.0524 | 0.4027 | 0.4700 | 0.5300 | 0.0523 | 0.4055 | 0.1160 | 0.8840 | 0.0136 | 0.4027 | 0.1245 | 0.8755 | 0.0136 | 0.4055 |
| | 0.7 | 0.6960 | 0.3040 | 0.0529 | 0.3065 | 0.7015 | 0.2985 | 0.0528 | 0.3088 | 0.1875 | 0.8125 | 0.0146 | 0.3065 | 0.1955 | 0.8045 | 0.0145 | 0.3088 |
| | 0.8 | 0.8885 | 0.1115 | 0.0518 | 0.2098 | 0.8875 | 0.1125 | 0.0519 | 0.2115 | 0.3300 | 0.6700 | 0.0144 | 0.2098 | 0.3450 | 0.6550 | 0.0145 | 0.2115 |
| | 0.85 | 0.9320 | 0.0680 | 0.0510 | 0.1611 | 0.9350 | 0.0650 | 0.0512 | 0.1627 | 0.5460 | 0.4540 | 0.0143 | 0.1611 | 0.5730 | 0.4270 | 0.0142 | 0.1627 |
| | 0.9 | 0.9680 | 0.0320 | 0.0511 | 0.1153 | 0.9710 | 0.0290 | 0.0511 | 0.1163 | 0.8690 | 0.1310 | 0.0143 | 0.1153 | 0.8750 | 0.1250 | 0.0143 | 0.1163 |
| | 0.95 | 0.9910 | 0.0090 | 0.0499 | 0.0664 | 0.9940 | 0.0060 | 0.0500 | 0.0671 | 0.9775 | 0.0225 | 0.0132 | 0.0664 | 0.9810 | 0.0190 | 0.0132 | 0.0671 |
| | 0.99 | 0.9970 | 0.0030 | 0.0522 | 0.0285 | 0.9995 | 0.0005 | 0.0513 | 0.0287 | 0.9960 | 0.0040 | 0.0108 | 0.0285 | 0.9985 | 0.0015 | 0.0108 | 0.0287 |
| **0.90** | 0.1 | 0.0780 | 0.9220 | 0.0516 | 0.8742 | 0.0775 | 0.9225 | 0.0522 | 0.8634 | 0.0310 | 0.9690 | 0.0183 | 0.8742 | 0.0275 | 0.9725 | 0.0174 | 0.8634 |
| | 0.2 | 0.1155 | 0.8845 | 0.0520 | 0.7909 | 0.1180 | 0.8820 | 0.0532 | 0.7864 | 0.0400 | 0.9600 | 0.0161 | 0.7909 | 0.0375 | 0.9625 | 0.0164 | 0.7864 |
| | 0.3 | 0.1640 | 0.8360 | 0.0518 | 0.6929 | 0.1650 | 0.8350 | 0.0516 | 0.6931 | 0.0455 | 0.9545 | 0.0142 | 0.6929 | 0.0475 | 0.9525 | 0.0146 | 0.6931 |
| | 0.4 | 0.2565 | 0.7435 | 0.0531 | 0.5978 | 0.2585 | 0.7415 | 0.0525 | 0.5993 | 0.0825 | 0.9175 | 0.0144 | 0.5978 | 0.0745 | 0.9255 | 0.0142 | 0.5993 |
| | 0.5 | 0.3550 | 0.6450 | 0.0535 | 0.5013 | 0.3655 | 0.6345 | 0.0526 | 0.5039 | 0.1055 | 0.8945 | 0.0141 | 0.5013 | 0.1095 | 0.8905 | 0.0140 | 0.5039 |
| | 0.6 | 0.5295 | 0.4705 | 0.0531 | 0.4061 | 0.5355 | 0.4645 | 0.0534 | 0.4087 | 0.1555 | 0.8445 | 0.0136 | 0.4061 | 0.1600 | 0.8400 | 0.0138 | 0.4087 |
| | 0.7 | 0.7385 | 0.2615 | 0.0531 | 0.3083 | 0.7445 | 0.2555 | 0.0530 | 0.3109 | 0.2615 | 0.7385 | 0.0144 | 0.3083 | 0.2670 | 0.7330 | 0.0143 | 0.3109 |
| | 0.8 | 0.9010 | 0.0990 | 0.0528 | 0.2121 | 0.9050 | 0.0950 | 0.0528 | 0.2136 | 0.5250 | 0.4750 | 0.0140 | 0.2121 | 0.5475 | 0.4525 | 0.0141 | 0.2136 |
| | 0.85 | 0.9440 | 0.0560 | 0.0510 | 0.1624 | 0.9440 | 0.0560 | 0.0513 | 0.1638 | 0.7470 | 0.2530 | 0.0144 | 0.1624 | 0.7605 | 0.2395 | 0.0145 | 0.1638 |
| | 0.9 | 0.9790 | 0.0210 | 0.0505 | 0.1145 | 0.9780 | 0.0220 | 0.0506 | 0.1154 | 0.9045 | 0.0955 | 0.0136 | 0.1145 | 0.9080 | 0.0920 | 0.0138 | 0.1154 |
| | 0.95 | 0.9900 | 0.0100 | 0.0503 | 0.0676 | 0.9930 | 0.0070 | 0.0505 | 0.0683 | 0.9750 | 0.0250 | 0.0135 | 0.0676 | 0.9780 | 0.0220 | 0.0135 | 0.0683 |
| | 0.99 | 0.9975 | 0.0025 | 0.0511 | 0.0289 | 0.9995 | 0.0005 | 0.0507 | 0.0291 | 0.9955 | 0.0045 | 0.0106 | 0.0289 | 0.9990 | 0.0010 | 0.0107 | 0.0291 |

Table 2. Simulation results for n=200, % 5 percentage of contamination

| | | **q=0.95** | | | | | | | | **q=0.99** | | | | | | | |
|---|---|---|---|---|---|---|---|---|---|---|---|---|---|---|---|---|---|
| | | NW | | | | LL | | | | NW | | | | LL | | | |
| $\gamma$ | $\rho$ | TDR | M | S | MCE | TDR | M | S | MCE | TDR | M | S | MCE | TDR | M | S | MCE |
| **0.10** | 0.1 | 0.0472 | 0.9528 | 0.0508 | 0.8755 | 0.0504 | 0.9496 | 0.0511 | 0.8634 | 0.0185 | 0.9815 | 0.0175 | 0.8755 | 0.0177 | 0.9823 | 0.0173 | 0.8634 |
| | 0.2 | 0.0554 | 0.9446 | 0.0542 | 0.7842 | 0.0560 | 0.9440 | 0.0546 | 0.7817 | 0.0186 | 0.9814 | 0.0185 | 0.7842 | 0.0171 | 0.9829 | 0.0178 | 0.7817 |
| | 0.3 | 0.0533 | 0.9467 | 0.0525 | 0.6872 | 0.0504 | 0.9496 | 0.0528 | 0.6870 | 0.0148 | 0.9852 | 0.0153 | 0.6872 | 0.0161 | 0.9839 | 0.0147 | 0.6870 |
| | 0.4 | 0.0527 | 0.9473 | 0.0532 | 0.5944 | 0.0522 | 0.9478 | 0.0538 | 0.5956 | 0.0141 | 0.9859 | 0.0145 | 0.5944 | 0.0147 | 0.9853 | 0.0144 | 0.5956 |
| | 0.5 | 0.0541 | 0.9459 | 0.0536 | 0.4923 | 0.0545 | 0.9455 | 0.0538 | 0.4946 | 0.0146 | 0.9854 | 0.0142 | 0.4923 | 0.0142 | 0.9858 | 0.0146 | 0.4946 |
| | 0.6 | 0.0561 | 0.9439 | 0.0534 | 0.3928 | 0.0552 | 0.9448 | 0.0536 | 0.3955 | 0.0153 | 0.9847 | 0.0144 | 0.3928 | 0.0159 | 0.9841 | 0.0149 | 0.3955 |
| | 0.7 | 0.0528 | 0.9472 | 0.0537 | 0.2982 | 0.0529 | 0.9471 | 0.0536 | 0.3004 | 0.0137 | 0.9863 | 0.0143 | 0.2982 | 0.0142 | 0.9858 | 0.0142 | 0.3004 |
| | 0.8 | 0.0555 | 0.9445 | 0.0521 | 0.1983 | 0.0547 | 0.9453 | 0.0519 | 0.1998 | 0.0148 | 0.9852 | 0.0141 | 0.1983 | 0.0149 | 0.9851 | 0.0140 | 0.1998 |
| | 0.85 | 0.0547 | 0.9453 | 0.0518 | 0.1489 | 0.0549 | 0.9451 | 0.0519 | 0.1500 | 0.0151 | 0.9849 | 0.0144 | 0.1489 | 0.0151 | 0.9849 | 0.0144 | 0.1500 |
| | 0.9 | 0.0555 | 0.9445 | 0.0507 | 0.0999 | 0.0562 | 0.9438 | 0.0508 | 0.1009 | 0.0127 | 0.9873 | 0.0140 | 0.0999 | 0.0131 | 0.9869 | 0.0140 | 0.1009 |
| | 0.95 | 0.0649 | 0.9351 | 0.0496 | 0.0507 | 0.0677 | 0.9323 | 0.0499 | 0.0513 | 0.0138 | 0.9862 | 0.0132 | 0.0507 | 0.0137 | 0.9863 | 0.0133 | 0.0513 |
| | 0.99 | 0.9717 | 0.0283 | 0.0504 | 0.0116 | 0.9800 | 0.0200 | 0.0509 | 0.0118 | 0.0132 | 0.9868 | 0.0103 | 0.0116 | 0.0135 | 0.9865 | 0.0104 | 0.0118 |
| **0.20** | 0.1 | 0.0543 | 0.9457 | 0.0515 | 0.8735 | 0.0535 | 0.9465 | 0.0520 | 0.8628 | 0.0189 | 0.9811 | 0.0177 | 0.8735 | 0.0202 | 0.9798 | 0.0170 | 0.8628 |
| | 0.2 | 0.0549 | 0.9451 | 0.0533 | 0.7866 | 0.0561 | 0.9439 | 0.0540 | 0.7827 | 0.0180 | 0.9820 | 0.0173 | 0.7866 | 0.0177 | 0.9823 | 0.0174 | 0.7827 |
| | 0.3 | 0.0576 | 0.9424 | 0.0535 | 0.6879 | 0.0581 | 0.9419 | 0.0538 | 0.6879 | 0.0167 | 0.9833 | 0.0155 | 0.6879 | 0.0172 | 0.9828 | 0.0155 | 0.6879 |
| | 0.4 | 0.0544 | 0.9456 | 0.0517 | 0.5959 | 0.0532 | 0.9468 | 0.0524 | 0.5974 | 0.0147 | 0.9853 | 0.0130 | 0.5959 | 0.0144 | 0.9856 | 0.0129 | 0.5974 |
| | 0.5 | 0.0566 | 0.9434 | 0.0536 | 0.4960 | 0.0555 | 0.9445 | 0.0535 | 0.4983 | 0.0147 | 0.9853 | 0.0138 | 0.4960 | 0.0153 | 0.9847 | 0.0135 | 0.4983 |
| | 0.6 | 0.0557 | 0.9443 | 0.0518 | 0.3964 | 0.0557 | 0.9443 | 0.0519 | 0.3992 | 0.0138 | 0.9862 | 0.0127 | 0.3964 | 0.0144 | 0.9856 | 0.0129 | 0.3992 |
| | 0.7 | 0.0569 | 0.9431 | 0.0528 | 0.3020 | 0.0571 | 0.9429 | 0.0524 | 0.3044 | 0.0138 | 0.9862 | 0.0134 | 0.3020 | 0.0148 | 0.9852 | 0.0133 | 0.3044 |
| | 0.8 | 0.0639 | 0.9361 | 0.0515 | 0.2037 | 0.0636 | 0.9364 | 0.0515 | 0.2054 | 0.0169 | 0.9831 | 0.0140 | 0.2037 | 0.0174 | 0.9826 | 0.0141 | 0.2054 |
| | 0.85 | 0.0596 | 0.9404 | 0.0510 | 0.1535 | 0.0610 | 0.9390 | 0.0510 | 0.1547 | 0.0152 | 0.9848 | 0.0140 | 0.1535 | 0.0150 | 0.9850 | 0.0141 | 0.1547 |
| | 0.9 | 0.0725 | 0.9275 | 0.0514 | 0.1074 | 0.0740 | 0.9260 | 0.0518 | 0.1084 | 0.0136 | 0.9864 | 0.0139 | 0.1074 | 0.0137 | 0.9863 | 0.0140 | 0.1084 |
| | 0.95 | 0.3650 | 0.6350 | 0.0494 | 0.0574 | 0.4379 | 0.5621 | 0.0501 | 0.0581 | 0.0161 | 0.9839 | 0.0133 | 0.0574 | 0.0164 | 0.9836 | 0.0133 | 0.0581 |
| | 0.99 | 0.9914 | 0.0086 | 0.0541 | 0.0185 | 0.9957 | 0.0043 | 0.0521 | 0.0189 | 0.5123 | 0.4877 | 0.0105 | 0.0185 | 0.6696 | 0.3304 | 0.0107 | 0.0189 |

Table 2. (continued)

| | | **q=0.95** | | | | | | | | **q=0.99** | | | | | | | |
|---|---|---|---|---|---|---|---|---|---|---|---|---|---|---|---|---|---|
| | | NW | | | | LL | | | | NW | | | | LL | | | |
| $\gamma$ | $\rho$ | TDR | M | S | MCE | TDR | M | S | MCE | TDR | M | S | MCE | TDR | M | S | MCE |
| **0.30** | 0.1 | 0.0591 | 0.9409 | 0.0508 | 0.8744 | 0.0556 | 0.9444 | 0.0504 | 0.8654 | 0.0199 | 0.9801 | 0.0174 | 0.8744 | 0.0176 | 0.9824 | 0.0164 | 0.8654 |
| | 0.2 | 0.0591 | 0.9409 | 0.0522 | 0.7921 | 0.0600 | 0.9400 | 0.0527 | 0.7878 | 0.0196 | 0.9804 | 0.0159 | 0.7921 | 0.0178 | 0.9822 | 0.0158 | 0.7878 |
| | 0.3 | 0.0586 | 0.9414 | 0.0508 | 0.6921 | 0.0584 | 0.9416 | 0.0513 | 0.6919 | 0.0141 | 0.9859 | 0.0139 | 0.6921 | 0.0145 | 0.9855 | 0.0137 | 0.6919 |
| | 0.4 | 0.0597 | 0.9403 | 0.0507 | 0.5964 | 0.0608 | 0.9392 | 0.0509 | 0.5984 | 0.0148 | 0.9852 | 0.0126 | 0.5964 | 0.0154 | 0.9846 | 0.0122 | 0.5984 |
| | 0.5 | 0.0650 | 0.9350 | 0.0527 | 0.5017 | 0.0655 | 0.9345 | 0.0526 | 0.5042 | 0.0170 | 0.9830 | 0.0130 | 0.5017 | 0.0171 | 0.9829 | 0.0129 | 0.5042 |
| | 0.6 | 0.0626 | 0.9374 | 0.0523 | 0.4033 | 0.0633 | 0.9367 | 0.0521 | 0.4061 | 0.0131 | 0.9869 | 0.0126 | 0.4033 | 0.0152 | 0.9848 | 0.0130 | 0.4061 |
| | 0.7 | 0.0674 | 0.9326 | 0.0513 | 0.3083 | 0.0673 | 0.9327 | 0.0510 | 0.3106 | 0.0167 | 0.9833 | 0.0127 | 0.3083 | 0.0165 | 0.9835 | 0.0127 | 0.3106 |
| | 0.8 | 0.0739 | 0.9261 | 0.0521 | 0.2132 | 0.0741 | 0.9259 | 0.0521 | 0.2149 | 0.0157 | 0.9843 | 0.0140 | 0.2132 | 0.0160 | 0.9840 | 0.0141 | 0.2149 |
| | 0.85 | 0.0806 | 0.9194 | 0.0510 | 0.1636 | 0.0819 | 0.9181 | 0.0513 | 0.1653 | 0.0181 | 0.9819 | 0.0142 | 0.1636 | 0.0180 | 0.9820 | 0.0144 | 0.1653 |
| | 0.9 | 0.1272 | 0.8728 | 0.0502 | 0.1150 | 0.1357 | 0.8643 | 0.0505 | 0.1162 | 0.0152 | 0.9848 | 0.0135 | 0.1150 | 0.0158 | 0.9842 | 0.0137 | 0.1162 |
| | 0.95 | 0.9262 | 0.0738 | 0.0495 | 0.0668 | 0.9403 | 0.0597 | 0.0499 | 0.0676 | 0.0187 | 0.9813 | 0.0128 | 0.0668 | 0.0191 | 0.9809 | 0.0129 | 0.0676 |
| | 0.99 | 0.9957 | 0.0043 | 0.0598 | 0.0285 | 0.9976 | 0.0024 | 0.0560 | 0.0290 | 0.9760 | 0.0240 | 0.0101 | 0.0285 | 0.9858 | 0.0142 | 0.0101 | 0.0290 |
| **0.40** | 0.1 | 0.0572 | 0.9428 | 0.0500 | 0.8752 | 0.0597 | 0.9403 | 0.0509 | 0.8649 | 0.0196 | 0.9804 | 0.0173 | 0.8752 | 0.0176 | 0.9824 | 0.0161 | 0.8649 |
| | 0.2 | 0.0609 | 0.9391 | 0.0520 | 0.7919 | 0.0653 | 0.9347 | 0.0528 | 0.7871 | 0.0164 | 0.9836 | 0.0153 | 0.7919 | 0.0193 | 0.9807 | 0.0157 | 0.7871 |
| | 0.3 | 0.0610 | 0.9390 | 0.0503 | 0.6962 | 0.0588 | 0.9412 | 0.0498 | 0.6956 | 0.0148 | 0.9852 | 0.0130 | 0.6962 | 0.0156 | 0.9844 | 0.0127 | 0.6956 |
| | 0.4 | 0.0687 | 0.9313 | 0.0504 | 0.6037 | 0.0701 | 0.9299 | 0.0503 | 0.6047 | 0.0166 | 0.9834 | 0.0117 | 0.6037 | 0.0170 | 0.9830 | 0.0119 | 0.6047 |
| | 0.5 | 0.0761 | 0.9239 | 0.0509 | 0.5098 | 0.0744 | 0.9256 | 0.0507 | 0.5118 | 0.0167 | 0.9833 | 0.0114 | 0.5098 | 0.0164 | 0.9836 | 0.0112 | 0.5118 |
| | 0.6 | 0.0799 | 0.9201 | 0.0504 | 0.4124 | 0.0790 | 0.9210 | 0.0506 | 0.4150 | 0.0178 | 0.9822 | 0.0117 | 0.4124 | 0.0177 | 0.9823 | 0.0120 | 0.4150 |
| | 0.7 | 0.0832 | 0.9168 | 0.0522 | 0.3187 | 0.0837 | 0.9163 | 0.0522 | 0.3211 | 0.0188 | 0.9812 | 0.0132 | 0.3187 | 0.0198 | 0.9802 | 0.0132 | 0.3211 |
| | 0.8 | 0.1025 | 0.8975 | 0.0516 | 0.2227 | 0.1049 | 0.8951 | 0.0517 | 0.2246 | 0.0199 | 0.9801 | 0.0136 | 0.2227 | 0.0204 | 0.9796 | 0.0134 | 0.2246 |
| | 0.85 | 0.1380 | 0.8620 | 0.0507 | 0.1741 | 0.1448 | 0.8552 | 0.0511 | 0.1756 | 0.0244 | 0.9756 | 0.0139 | 0.1741 | 0.0247 | 0.9753 | 0.0139 | 0.1756 |
| | 0.9 | 0.5817 | 0.4183 | 0.0500 | 0.1277 | 0.6436 | 0.3564 | 0.0503 | 0.1289 | 0.0222 | 0.9778 | 0.0137 | 0.1277 | 0.0229 | 0.9771 | 0.0139 | 0.1289 |
| | 0.95 | 0.9691 | 0.0309 | 0.0491 | 0.0794 | 0.9759 | 0.0241 | 0.0497 | 0.0804 | 0.0276 | 0.9724 | 0.0128 | 0.0794 | 0.0284 | 0.9716 | 0.0129 | 0.0804 |
| | 0.99 | 0.9960 | 0.0040 | 0.0668 | 0.0415 | 0.9976 | 0.0024 | 0.0601 | 0.0422 | 0.9881 | 0.0119 | 0.0099 | 0.0415 | 0.9926 | 0.0074 | 0.0100 | 0.0422 |

Table 2. (continued)

| | | **q=0.95** | | | | | | | | **q=0.99** | | | | | | | |
|---|---|---|---|---|---|---|---|---|---|---|---|---|---|---|---|---|---|
| | | NW | | | | LL | | | | NW | | | | LL | | | |
| $\gamma$ | $\rho$ | TDR | M | S | MCE | TDR | M | S | MCE | TDR | M | S | MCE | TDR | M | S | MCE |
| **0.50** | 0.1 | 0.0556 | 0.9444 | 0.0500 | 0.8768 | 0.0579 | 0.9421 | 0.0520 | 0.8646 | 0.0203 | 0.9797 | 0.0165 | 0.8768 | 0.0193 | 0.9807 | 0.0173 | 0.8646 |
| | 0.2 | 0.0680 | 0.9320 | 0.0499 | 0.7956 | 0.0688 | 0.9312 | 0.0507 | 0.7910 | 0.0223 | 0.9777 | 0.0143 | 0.7956 | 0.0199 | 0.9801 | 0.0143 | 0.7910 |
| | 0.3 | 0.0738 | 0.9262 | 0.0488 | 0.7005 | 0.0779 | 0.9221 | 0.0489 | 0.6996 | 0.0198 | 0.9802 | 0.0119 | 0.7005 | 0.0183 | 0.9817 | 0.0120 | 0.6996 |
| | 0.4 | 0.0806 | 0.9194 | 0.0484 | 0.6089 | 0.0812 | 0.9188 | 0.0484 | 0.6099 | 0.0174 | 0.9826 | 0.0108 | 0.6089 | 0.0193 | 0.9807 | 0.0106 | 0.6099 |
| | 0.5 | 0.0911 | 0.9089 | 0.0496 | 0.5164 | 0.0897 | 0.9103 | 0.0497 | 0.5181 | 0.0195 | 0.9805 | 0.0107 | 0.5164 | 0.0207 | 0.9793 | 0.0106 | 0.5181 |
| | 0.6 | 0.1027 | 0.8973 | 0.0501 | 0.4210 | 0.1031 | 0.8969 | 0.0502 | 0.4233 | 0.0234 | 0.9766 | 0.0116 | 0.4210 | 0.0233 | 0.9767 | 0.0117 | 0.4233 |
| | 0.7 | 0.1120 | 0.8880 | 0.0515 | 0.3283 | 0.1163 | 0.8837 | 0.0515 | 0.3309 | 0.0253 | 0.9747 | 0.0128 | 0.3283 | 0.0242 | 0.9758 | 0.0131 | 0.3309 |
| | 0.8 | 0.1762 | 0.8238 | 0.0512 | 0.2332 | 0.1833 | 0.8167 | 0.0513 | 0.2354 | 0.0285 | 0.9715 | 0.0134 | 0.2332 | 0.0299 | 0.9701 | 0.0135 | 0.2354 |
| | 0.85 | 0.4099 | 0.5901 | 0.0514 | 0.1881 | 0.4429 | 0.5571 | 0.0516 | 0.1897 | 0.0256 | 0.9744 | 0.0137 | 0.1881 | 0.0266 | 0.9734 | 0.0139 | 0.1897 |
| | 0.9 | 0.8885 | 0.1115 | 0.0500 | 0.1404 | 0.9024 | 0.0976 | 0.0505 | 0.1417 | 0.0281 | 0.9719 | 0.0130 | 0.1404 | 0.0289 | 0.9711 | 0.0131 | 0.1417 |
| | 0.95 | 0.9840 | 0.0160 | 0.0488 | 0.0934 | 0.9861 | 0.0139 | 0.0490 | 0.0944 | 0.0596 | 0.9404 | 0.0126 | 0.0934 | 0.0636 | 0.9364 | 0.0127 | 0.0944 |
| | 0.99 | 0.9972 | 0.0028 | 0.0703 | 0.0571 | 0.9991 | 0.0009 | 0.0616 | 0.0578 | 0.9935 | 0.0065 | 0.0105 | 0.0571 | 0.9968 | 0.0032 | 0.0106 | 0.0578 |
| **0.60** | 0.1 | 0.0573 | 0.9427 | 0.0478 | 0.8800 | 0.0604 | 0.9396 | 0.0485 | 0.8678 | 0.0182 | 0.9818 | 0.0159 | 0.8800 | 0.0182 | 0.9818 | 0.0153 | 0.8678 |
| | 0.2 | 0.0749 | 0.9251 | 0.0490 | 0.7985 | 0.0780 | 0.9220 | 0.0499 | 0.7939 | 0.0220 | 0.9780 | 0.0143 | 0.7985 | 0.0238 | 0.9762 | 0.0147 | 0.7939 |
| | 0.3 | 0.0880 | 0.9120 | 0.0476 | 0.7060 | 0.0866 | 0.9134 | 0.0474 | 0.7056 | 0.0218 | 0.9782 | 0.0113 | 0.7060 | 0.0219 | 0.9781 | 0.0113 | 0.7056 |
| | 0.4 | 0.0988 | 0.9012 | 0.0489 | 0.6176 | 0.1018 | 0.8982 | 0.0484 | 0.6184 | 0.0211 | 0.9789 | 0.0105 | 0.6176 | 0.0212 | 0.9788 | 0.0101 | 0.6184 |
| | 0.5 | 0.1190 | 0.8810 | 0.0497 | 0.5239 | 0.1211 | 0.8789 | 0.0497 | 0.5260 | 0.0252 | 0.9748 | 0.0106 | 0.5239 | 0.0269 | 0.9731 | 0.0104 | 0.5260 |
| | 0.6 | 0.1467 | 0.8533 | 0.0500 | 0.4327 | 0.1518 | 0.8482 | 0.0499 | 0.4353 | 0.0296 | 0.9704 | 0.0112 | 0.4327 | 0.0296 | 0.9704 | 0.0112 | 0.4353 |
| | 0.7 | 0.1793 | 0.8207 | 0.0506 | 0.3373 | 0.1854 | 0.8146 | 0.0506 | 0.3398 | 0.0331 | 0.9669 | 0.0125 | 0.3373 | 0.0339 | 0.9661 | 0.0127 | 0.3398 |
| | 0.8 | 0.4257 | 0.5743 | 0.0518 | 0.2480 | 0.4479 | 0.5521 | 0.0519 | 0.2502 | 0.0372 | 0.9628 | 0.0142 | 0.2480 | 0.0388 | 0.9612 | 0.0142 | 0.2502 |
| | 0.85 | 0.7895 | 0.2105 | 0.0510 | 0.2004 | 0.8131 | 0.1869 | 0.0510 | 0.2022 | 0.0397 | 0.9603 | 0.0141 | 0.2004 | 0.0412 | 0.9588 | 0.0142 | 0.2022 |
| | 0.9 | 0.9432 | 0.0568 | 0.0500 | 0.1543 | 0.9494 | 0.0506 | 0.0501 | 0.1557 | 0.0520 | 0.9480 | 0.0134 | 0.1543 | 0.0542 | 0.9458 | 0.0134 | 0.1557 |
| | 0.95 | 0.9883 | 0.0117 | 0.0487 | 0.1088 | 0.9919 | 0.0081 | 0.0489 | 0.1099 | 0.6623 | 0.3377 | 0.0132 | 0.1088 | 0.7254 | 0.2746 | 0.0132 | 0.1099 |
| | 0.99 | 0.9971 | 0.0029 | 0.0714 | 0.0719 | 0.9987 | 0.0013 | 0.0625 | 0.0726 | 0.9937 | 0.0063 | 0.0102 | 0.0719 | 0.9970 | 0.0030 | 0.0103 | 0.0726 |

Table 2. (continued)

| | | q=0.95 | | | | | | | | q=0.99 | | | | | | | |
|---|---|---|---|---|---|---|---|---|---|---|---|---|---|---|---|---|---|
| | | NW | | | | LL | | | | NW | | | | LL | | | |
| $\gamma$ | $\rho$ | TDR | M | S | MCE | TDR | M | S | MCE | TDR | M | S | MCE | TDR | M | S | MCE |
| **0.70** | 0.1 | 0.0623 | 0.9377 | 0.0496 | 0.8785 | 0.0648 | 0.9352 | 0.0501 | 0.8687 | 0.0207 | 0.9793 | 0.0167 | 0.8785 | 0.0206 | 0.9794 | 0.0160 | 0.8687 |
| | 0.2 | 0.0846 | 0.9154 | 0.0477 | 0.7982 | 0.0843 | 0.9157 | 0.0479 | 0.7953 | 0.0259 | 0.9741 | 0.0140 | 0.7982 | 0.0224 | 0.9776 | 0.0137 | 0.7953 |
| | 0.3 | 0.1041 | 0.8959 | 0.0463 | 0.7113 | 0.1016 | 0.8984 | 0.0458 | 0.7099 | 0.0263 | 0.9737 | 0.0105 | 0.7113 | 0.0249 | 0.9751 | 0.0103 | 0.7099 |
| | 0.4 | 0.1350 | 0.8650 | 0.0481 | 0.6211 | 0.1343 | 0.8657 | 0.0482 | 0.6225 | 0.0321 | 0.9679 | 0.0102 | 0.6211 | 0.0300 | 0.9700 | 0.0101 | 0.6225 |
| | 0.5 | 0.1773 | 0.8227 | 0.0481 | 0.5287 | 0.1826 | 0.8174 | 0.0479 | 0.5309 | 0.0398 | 0.9602 | 0.0098 | 0.5287 | 0.0403 | 0.9597 | 0.0098 | 0.5309 |
| | 0.6 | 0.2283 | 0.7717 | 0.0502 | 0.4402 | 0.2315 | 0.7685 | 0.0498 | 0.4426 | 0.0453 | 0.9547 | 0.0113 | 0.4402 | 0.0469 | 0.9531 | 0.0119 | 0.4426 |
| | 0.7 | 0.3599 | 0.6401 | 0.0515 | 0.3500 | 0.3740 | 0.6260 | 0.0515 | 0.3523 | 0.0583 | 0.9417 | 0.0129 | 0.3500 | 0.0589 | 0.9411 | 0.0131 | 0.3523 |
| | 0.8 | 0.7328 | 0.2672 | 0.0511 | 0.2582 | 0.7492 | 0.2508 | 0.0514 | 0.2602 | 0.0687 | 0.9313 | 0.0137 | 0.2582 | 0.0712 | 0.9288 | 0.0137 | 0.2602 |
| | 0.85 | 0.8922 | 0.1078 | 0.0508 | 0.2121 | 0.9012 | 0.0988 | 0.0514 | 0.2140 | 0.0840 | 0.9160 | 0.0137 | 0.2121 | 0.0881 | 0.9119 | 0.0139 | 0.2140 |
| | 0.9 | 0.9652 | 0.0348 | 0.0495 | 0.1667 | 0.9694 | 0.0306 | 0.0496 | 0.1680 | 0.1414 | 0.8586 | 0.0133 | 0.1667 | 0.1543 | 0.8457 | 0.0133 | 0.1680 |
| | 0.95 | 0.9886 | 0.0114 | 0.0488 | 0.1224 | 0.9918 | 0.0082 | 0.0494 | 0.1234 | 0.9392 | 0.0608 | 0.0132 | 0.1224 | 0.9486 | 0.0514 | 0.0132 | 0.1234 |
| | 0.99 | 0.9982 | 0.0018 | 0.0669 | 0.0859 | 0.9996 | 0.0004 | 0.0604 | 0.0866 | 0.9948 | 0.0052 | 0.0097 | 0.0859 | 0.9977 | 0.0023 | 0.0098 | 0.0866 |
| **0.80** | 0.1 | 0.0623 | 0.9377 | 0.0478 | 0.8809 | 0.0635 | 0.9365 | 0.0486 | 0.8687 | 0.0221 | 0.9779 | 0.0155 | 0.8809 | 0.0195 | 0.9805 | 0.0155 | 0.8687 |
| | 0.2 | 0.0869 | 0.9131 | 0.0467 | 0.8007 | 0.0909 | 0.9091 | 0.0482 | 0.7950 | 0.0256 | 0.9744 | 0.0127 | 0.8007 | 0.0249 | 0.9751 | 0.0133 | 0.7950 |
| | 0.3 | 0.1201 | 0.8799 | 0.0465 | 0.7142 | 0.1223 | 0.8777 | 0.0460 | 0.7122 | 0.0293 | 0.9707 | 0.0106 | 0.7142 | 0.0281 | 0.9719 | 0.0105 | 0.7122 |
| | 0.4 | 0.1793 | 0.8207 | 0.0470 | 0.6252 | 0.1808 | 0.8192 | 0.0475 | 0.6258 | 0.0379 | 0.9621 | 0.0099 | 0.6252 | 0.0374 | 0.9626 | 0.0097 | 0.6258 |
| | 0.5 | 0.2499 | 0.7501 | 0.0489 | 0.5355 | 0.2546 | 0.7454 | 0.0488 | 0.5375 | 0.0556 | 0.9444 | 0.0103 | 0.5355 | 0.0574 | 0.9426 | 0.0103 | 0.5375 |
| | 0.6 | 0.3582 | 0.6418 | 0.0504 | 0.4470 | 0.3688 | 0.6312 | 0.0503 | 0.4500 | 0.0751 | 0.9249 | 0.0109 | 0.4470 | 0.0770 | 0.9230 | 0.0111 | 0.4500 |
| | 0.7 | 0.5791 | 0.4209 | 0.0510 | 0.3579 | 0.5977 | 0.4023 | 0.0509 | 0.3600 | 0.1063 | 0.8937 | 0.0125 | 0.3579 | 0.1073 | 0.8927 | 0.0126 | 0.3600 |
| | 0.8 | 0.8536 | 0.1464 | 0.0519 | 0.2686 | 0.8616 | 0.1384 | 0.0520 | 0.2706 | 0.1640 | 0.8360 | 0.0135 | 0.2686 | 0.1705 | 0.8295 | 0.0139 | 0.2706 |
| | 0.85 | 0.9295 | 0.0705 | 0.0512 | 0.2227 | 0.9345 | 0.0655 | 0.0514 | 0.2243 | 0.2699 | 0.7301 | 0.0141 | 0.2227 | 0.2926 | 0.7074 | 0.0143 | 0.2243 |
| | 0.9 | 0.9691 | 0.0309 | 0.0511 | 0.1796 | 0.9726 | 0.0274 | 0.0515 | 0.1810 | 0.7029 | 0.2971 | 0.0139 | 0.1796 | 0.7419 | 0.2581 | 0.0140 | 0.1810 |
| | 0.95 | 0.9899 | 0.0101 | 0.0499 | 0.1344 | 0.9926 | 0.0074 | 0.0501 | 0.1353 | 0.9672 | 0.0328 | 0.0138 | 0.1344 | 0.9719 | 0.0281 | 0.0139 | 0.1353 |
| | 0.99 | 0.9959 | 0.0041 | 0.0634 | 0.0975 | 0.9987 | 0.0013 | 0.0575 | 0.0983 | 0.9921 | 0.0079 | 0.0105 | 0.0975 | 0.9969 | 0.0031 | 0.0105 | 0.0983 |

Table 2. (continued)

| | | q=0.95 | | | | | | | | q=0.99 | | | | | | | |
|---|---|---|---|---|---|---|---|---|---|---|---|---|---|---|---|---|---|
| | | NW | | | | LL | | | | NW | | | | LL | | | |
| $\gamma$ | $\rho$ | TDR | M | S | MCE | TDR | M | S | MCE | TDR | M | S | MCE | TDR | M | S | MCE |
| **0.85** | 0.1 | 0.0644 | 0.9356 | 0.0483 | 0.8794 | 0.0676 | 0.9324 | 0.0491 | 0.8674 | 0.0237 | 0.9763 | 0.0162 | 0.8794 | 0.0208 | 0.9792 | 0.0157 | 0.8674 |
| | 0.2 | 0.0981 | 0.9019 | 0.0478 | 0.8013 | 0.1006 | 0.8994 | 0.0487 | 0.7960 | 0.0291 | 0.9709 | 0.0136 | 0.8013 | 0.0279 | 0.9721 | 0.0136 | 0.7960 |
| | 0.3 | 0.1319 | 0.8681 | 0.0457 | 0.7168 | 0.1360 | 0.8640 | 0.0464 | 0.7153 | 0.0292 | 0.9708 | 0.0102 | 0.7168 | 0.0298 | 0.9702 | 0.0107 | 0.7153 |
| | 0.4 | 0.1937 | 0.8063 | 0.0470 | 0.6266 | 0.1960 | 0.8040 | 0.0470 | 0.6286 | 0.0444 | 0.9556 | 0.0099 | 0.6266 | 0.0436 | 0.9564 | 0.0096 | 0.6286 |
| | 0.5 | 0.2994 | 0.7006 | 0.0489 | 0.5375 | 0.3054 | 0.6946 | 0.0489 | 0.5393 | 0.0654 | 0.9346 | 0.0102 | 0.5375 | 0.0706 | 0.9294 | 0.0102 | 0.5393 |
| | 0.6 | 0.4477 | 0.5523 | 0.0497 | 0.4489 | 0.4590 | 0.5410 | 0.0498 | 0.4514 | 0.1009 | 0.8991 | 0.0115 | 0.4489 | 0.1092 | 0.8908 | 0.0120 | 0.4514 |
| | 0.7 | 0.6743 | 0.3257 | 0.0507 | 0.3589 | 0.6856 | 0.3144 | 0.0507 | 0.3607 | 0.1601 | 0.8399 | 0.0129 | 0.3589 | 0.1654 | 0.8346 | 0.0126 | 0.3607 |
| | 0.8 | 0.8751 | 0.1249 | 0.0514 | 0.2703 | 0.8800 | 0.1200 | 0.0513 | 0.2722 | 0.2841 | 0.7159 | 0.0137 | 0.2703 | 0.2988 | 0.7012 | 0.0136 | 0.2722 |
| | 0.85 | 0.9409 | 0.0591 | 0.0510 | 0.2265 | 0.9447 | 0.0553 | 0.0513 | 0.2280 | 0.5122 | 0.4878 | 0.0144 | 0.2265 | 0.5479 | 0.4521 | 0.0145 | 0.2280 |
| | 0.9 | 0.9717 | 0.0283 | 0.0502 | 0.1827 | 0.9741 | 0.0259 | 0.0505 | 0.1838 | 0.8413 | 0.1587 | 0.0137 | 0.1827 | 0.8553 | 0.1447 | 0.0138 | 0.1838 |
| | 0.95 | 0.9903 | 0.0097 | 0.0501 | 0.1378 | 0.9928 | 0.0072 | 0.0503 | 0.1389 | 0.9668 | 0.0332 | 0.0130 | 0.1378 | 0.9712 | 0.0288 | 0.0130 | 0.1389 |
| | 0.99 | 0.9963 | 0.0037 | 0.0598 | 0.1021 | 0.9990 | 0.0010 | 0.0552 | 0.1027 | 0.9941 | 0.0059 | 0.0101 | 0.1021 | 0.9978 | 0.0022 | 0.0101 | 0.1027 |
| **0.90** | 0.1 | 0.0651 | 0.9349 | 0.0475 | 0.8835 | 0.0623 | 0.9377 | 0.0491 | 0.8703 | 0.0216 | 0.9784 | 0.0155 | 0.8835 | 0.0193 | 0.9807 | 0.0151 | 0.8703 |
| | 0.2 | 0.0928 | 0.9072 | 0.0475 | 0.8022 | 0.0998 | 0.9002 | 0.0482 | 0.7974 | 0.0247 | 0.9753 | 0.0131 | 0.8022 | 0.0280 | 0.9720 | 0.0131 | 0.7974 |
| | 0.3 | 0.1394 | 0.8606 | 0.0446 | 0.7175 | 0.1433 | 0.8567 | 0.0452 | 0.7161 | 0.0324 | 0.9676 | 0.0095 | 0.7175 | 0.0353 | 0.9647 | 0.0097 | 0.7161 |
| | 0.4 | 0.2173 | 0.7827 | 0.0477 | 0.6286 | 0.2244 | 0.7756 | 0.0480 | 0.6299 | 0.0493 | 0.9507 | 0.0096 | 0.6286 | 0.0486 | 0.9514 | 0.0097 | 0.6299 |
| | 0.5 | 0.3324 | 0.6676 | 0.0489 | 0.5399 | 0.3365 | 0.6635 | 0.0486 | 0.5418 | 0.0774 | 0.9226 | 0.0102 | 0.5399 | 0.0775 | 0.9225 | 0.0099 | 0.5418 |
| | 0.6 | 0.5113 | 0.4887 | 0.0505 | 0.4503 | 0.5205 | 0.4795 | 0.0506 | 0.4533 | 0.1369 | 0.8631 | 0.0112 | 0.4503 | 0.1414 | 0.8586 | 0.0112 | 0.4533 |
| | 0.7 | 0.7251 | 0.2749 | 0.0521 | 0.3634 | 0.7290 | 0.2710 | 0.0516 | 0.3658 | 0.2312 | 0.7688 | 0.0128 | 0.3634 | 0.2449 | 0.7551 | 0.0129 | 0.3658 |
| | 0.8 | 0.8906 | 0.1094 | 0.0514 | 0.2729 | 0.8934 | 0.1066 | 0.0512 | 0.2747 | 0.4690 | 0.5310 | 0.0138 | 0.2729 | 0.4933 | 0.5067 | 0.0141 | 0.2747 |
| | 0.85 | 0.9453 | 0.0547 | 0.0505 | 0.2287 | 0.9482 | 0.0518 | 0.0508 | 0.2303 | 0.7070 | 0.2930 | 0.0141 | 0.2287 | 0.7277 | 0.2723 | 0.0141 | 0.2303 |
| | 0.9 | 0.9716 | 0.0284 | 0.0503 | 0.1851 | 0.9740 | 0.0260 | 0.0505 | 0.1861 | 0.8908 | 0.1092 | 0.0139 | 0.1851 | 0.8971 | 0.1029 | 0.0140 | 0.1861 |
| | 0.95 | 0.9916 | 0.0084 | 0.0489 | 0.1401 | 0.9934 | 0.0066 | 0.0490 | 0.1409 | 0.9745 | 0.0255 | 0.0128 | 0.1401 | 0.9769 | 0.0231 | 0.0129 | 0.1409 |
| | 0.99 | 0.9965 | 0.0035 | 0.0566 | 0.1060 | 0.9984 | 0.0016 | 0.0527 | 0.1064 | 0.9938 | 0.0062 | 0.0106 | 0.1060 | 0.9975 | 0.0025 | 0.0107 | 0.1064 |

Table 3. Simulation results for n=200, % 10 percentage of contamination

| | | **q=0.95** | | | | | | | | **q=0.99** | | | | | | | |
|---|---|---|---|---|---|---|---|---|---|---|---|---|---|---|---|---|---|
| | | NW | | | | LL | | | | NW | | | | LL | | | |
| $\gamma$ | $\rho$ | TDR | M | S | MCE | TDR | M | S | MCE | TDR | M | S | MCE | TDR | M | S | MCE |
| **0.10** | 0.1 | 0.0528 | 0.9472 | 0.0511 | 0.8735 | 0.0536 | 0.9464 | 0.0521 | 0.8646 | 0.0178 | 0.9823 | 0.0179 | 0.8735 | 0.0170 | 0.9831 | 0.0169 | 0.8646 |
| | 0.2 | 0.0535 | 0.9465 | 0.0542 | 0.7861 | 0.0529 | 0.9472 | 0.0537 | 0.7820 | 0.0175 | 0.9825 | 0.0176 | 0.7861 | 0.0175 | 0.9826 | 0.0174 | 0.7820 |
| | 0.3 | 0.0544 | 0.9456 | 0.0516 | 0.6884 | 0.0537 | 0.9464 | 0.0512 | 0.6878 | 0.0148 | 0.9852 | 0.0142 | 0.6884 | 0.0136 | 0.9865 | 0.0140 | 0.6878 |
| | 0.4 | 0.0548 | 0.9453 | 0.0526 | 0.5905 | 0.0527 | 0.9474 | 0.0525 | 0.5917 | 0.0160 | 0.9841 | 0.0140 | 0.5905 | 0.0153 | 0.9847 | 0.0139 | 0.5917 |
| | 0.5 | 0.0540 | 0.9460 | 0.0529 | 0.4914 | 0.0544 | 0.9456 | 0.0528 | 0.4940 | 0.0145 | 0.9855 | 0.0139 | 0.4914 | 0.0140 | 0.9861 | 0.0136 | 0.4940 |
| | 0.6 | 0.0558 | 0.9442 | 0.0524 | 0.3943 | 0.0555 | 0.9445 | 0.0524 | 0.3971 | 0.0149 | 0.9851 | 0.0137 | 0.3943 | 0.0153 | 0.9847 | 0.0136 | 0.3971 |
| | 0.7 | 0.0525 | 0.9476 | 0.0524 | 0.2976 | 0.0529 | 0.9472 | 0.0522 | 0.2999 | 0.0138 | 0.9862 | 0.0139 | 0.2976 | 0.0137 | 0.9863 | 0.0139 | 0.2999 |
| | 0.8 | 0.0545 | 0.9455 | 0.0518 | 0.1997 | 0.0542 | 0.9458 | 0.0518 | 0.2015 | 0.0146 | 0.9855 | 0.0140 | 0.1997 | 0.0146 | 0.9855 | 0.0140 | 0.2015 |
| | 0.85 | 0.0520 | 0.9481 | 0.0515 | 0.1509 | 0.0521 | 0.9480 | 0.0517 | 0.1523 | 0.0133 | 0.9867 | 0.0149 | 0.1509 | 0.0137 | 0.9864 | 0.0150 | 0.1523 |
| | 0.9 | 0.0528 | 0.9472 | 0.0489 | 0.1001 | 0.0531 | 0.9470 | 0.0489 | 0.1010 | 0.0134 | 0.9866 | 0.0132 | 0.1001 | 0.0134 | 0.9866 | 0.0133 | 0.1010 |
| | 0.95 | 0.0600 | 0.9400 | 0.0502 | 0.0531 | 0.0624 | 0.9377 | 0.0506 | 0.0538 | 0.0131 | 0.9869 | 0.0133 | 0.0531 | 0.0136 | 0.9865 | 0.0133 | 0.0538 |
| | 0.99 | 0.9625 | 0.0376 | 0.0477 | 0.0138 | 0.9777 | 0.0223 | 0.0477 | 0.0140 | 0.0116 | 0.9885 | 0.0103 | 0.0138 | 0.0122 | 0.9879 | 0.0105 | 0.0140 |
| **0.20** | 0.1 | 0.0527 | 0.9473 | 0.0500 | 0.8750 | 0.0517 | 0.9484 | 0.0515 | 0.8636 | 0.0169 | 0.9831 | 0.0170 | 0.8750 | 0.0167 | 0.9834 | 0.0172 | 0.8636 |
| | 0.2 | 0.0558 | 0.9443 | 0.0508 | 0.7892 | 0.0574 | 0.9426 | 0.0519 | 0.7845 | 0.0201 | 0.9800 | 0.0156 | 0.7892 | 0.0199 | 0.9802 | 0.0158 | 0.7845 |
| | 0.3 | 0.0541 | 0.9459 | 0.0519 | 0.6942 | 0.0544 | 0.9457 | 0.0521 | 0.6936 | 0.0137 | 0.9863 | 0.0142 | 0.6942 | 0.0153 | 0.9847 | 0.0140 | 0.6936 |
| | 0.4 | 0.0556 | 0.9445 | 0.0513 | 0.5953 | 0.0551 | 0.9449 | 0.0518 | 0.5966 | 0.0129 | 0.9871 | 0.0130 | 0.5953 | 0.0139 | 0.9861 | 0.0127 | 0.5966 |
| | 0.5 | 0.0601 | 0.9399 | 0.0516 | 0.5018 | 0.0602 | 0.9399 | 0.0514 | 0.5044 | 0.0145 | 0.9856 | 0.0125 | 0.5018 | 0.0154 | 0.9846 | 0.0126 | 0.5044 |
| | 0.6 | 0.0536 | 0.9464 | 0.0506 | 0.4010 | 0.0534 | 0.9467 | 0.0505 | 0.4038 | 0.0124 | 0.9877 | 0.0122 | 0.4010 | 0.0121 | 0.9879 | 0.0126 | 0.4038 |
| | 0.7 | 0.0600 | 0.9400 | 0.0516 | 0.3083 | 0.0604 | 0.9397 | 0.0515 | 0.3109 | 0.0154 | 0.9847 | 0.0122 | 0.3083 | 0.0153 | 0.9848 | 0.0125 | 0.3109 |
| | 0.8 | 0.0579 | 0.9422 | 0.0504 | 0.2101 | 0.0585 | 0.9416 | 0.0503 | 0.2119 | 0.0153 | 0.9847 | 0.0130 | 0.2101 | 0.0157 | 0.9844 | 0.0131 | 0.2119 |
| | 0.85 | 0.0562 | 0.9438 | 0.0505 | 0.1608 | 0.0570 | 0.9430 | 0.0510 | 0.1623 | 0.0123 | 0.9877 | 0.0135 | 0.1608 | 0.0129 | 0.9872 | 0.0137 | 0.1623 |
| | 0.9 | 0.0662 | 0.9339 | 0.0496 | 0.1135 | 0.0680 | 0.9321 | 0.0500 | 0.1147 | 0.0154 | 0.9846 | 0.0132 | 0.1135 | 0.0156 | 0.9845 | 0.0133 | 0.1147 |
| | 0.95 | 0.2192 | 0.7809 | 0.0486 | 0.0652 | 0.2647 | 0.7354 | 0.0490 | 0.0660 | 0.0151 | 0.9850 | 0.0133 | 0.0652 | 0.0151 | 0.9850 | 0.0134 | 0.0660 |
| | 0.99 | 0.9908 | 0.0093 | 0.0533 | 0.0263 | 0.9942 | 0.0059 | 0.0506 | 0.0268 | 0.1157 | 0.8843 | 0.0101 | 0.0263 | 0.1831 | 0.8170 | 0.0103 | 0.0268 |

Table 3. (continued)

| | | **q=0.95** | | | | | | | | **q=0.99** | | | | | | | |
|---|---|---|---|---|---|---|---|---|---|---|---|---|---|---|---|---|---|
| | | NW | | | | LL | | | | NW | | | | LL | | | |
| $\gamma$ | $\rho$ | TDR | M | S | MCE | TDR | M | S | MCE | TDR | M | S | MCE | TDR | M | S | MCE |
| **0.30** | 0.1 | 0.0532 | 0.9469 | 0.0505 | 0.8769 | 0.0546 | 0.9455 | 0.0509 | 0.8631 | 0.0178 | 0.9822 | 0.0169 | 0.8769 | 0.0184 | 0.9817 | 0.0169 | 0.8631 |
| | 0.2 | 0.0568 | 0.9432 | 0.0506 | 0.7939 | 0.0591 | 0.9410 | 0.0507 | 0.7904 | 0.0164 | 0.9837 | 0.0151 | 0.7939 | 0.0155 | 0.9845 | 0.0145 | 0.7904 |
| | 0.3 | 0.0571 | 0.9430 | 0.0486 | 0.6981 | 0.0574 | 0.9426 | 0.0482 | 0.6977 | 0.0151 | 0.9849 | 0.0121 | 0.6981 | 0.0147 | 0.9853 | 0.0122 | 0.6977 |
| | 0.4 | 0.0569 | 0.9431 | 0.0493 | 0.6040 | 0.0588 | 0.9413 | 0.0493 | 0.6058 | 0.0137 | 0.9863 | 0.0113 | 0.6040 | 0.0131 | 0.9869 | 0.0109 | 0.6058 |
| | 0.5 | 0.0608 | 0.9393 | 0.0505 | 0.5124 | 0.0605 | 0.9395 | 0.0501 | 0.5145 | 0.0121 | 0.9879 | 0.0106 | 0.5124 | 0.0124 | 0.9877 | 0.0102 | 0.5145 |
| | 0.6 | 0.0614 | 0.9386 | 0.0494 | 0.4162 | 0.0613 | 0.9388 | 0.0493 | 0.4184 | 0.0127 | 0.9874 | 0.0106 | 0.4162 | 0.0137 | 0.9864 | 0.0105 | 0.4184 |
| | 0.7 | 0.0641 | 0.9359 | 0.0497 | 0.3207 | 0.0636 | 0.9365 | 0.0498 | 0.3230 | 0.0144 | 0.9857 | 0.0115 | 0.3207 | 0.0141 | 0.9860 | 0.0117 | 0.3230 |
| | 0.8 | 0.0697 | 0.9303 | 0.0506 | 0.2263 | 0.0702 | 0.9299 | 0.0508 | 0.2281 | 0.0169 | 0.9831 | 0.0127 | 0.2263 | 0.0172 | 0.9829 | 0.0128 | 0.2281 |
| | 0.85 | 0.0737 | 0.9264 | 0.0494 | 0.1779 | 0.0762 | 0.9239 | 0.0499 | 0.1798 | 0.0161 | 0.9839 | 0.0134 | 0.1779 | 0.0165 | 0.9835 | 0.0137 | 0.1798 |
| | 0.9 | 0.1070 | 0.8931 | 0.0490 | 0.1318 | 0.1159 | 0.8842 | 0.0495 | 0.1332 | 0.0173 | 0.9827 | 0.0135 | 0.1318 | 0.0175 | 0.9826 | 0.0138 | 0.1332 |
| | 0.95 | 0.8845 | 0.1155 | 0.0470 | 0.0836 | 0.9091 | 0.0910 | 0.0475 | 0.0846 | 0.0174 | 0.9827 | 0.0130 | 0.0836 | 0.0178 | 0.9823 | 0.0132 | 0.0846 |
| | 0.99 | 0.9948 | 0.0053 | 0.0669 | 0.0459 | 0.9971 | 0.0030 | 0.0574 | 0.0467 | 0.9636 | 0.0365 | 0.0096 | 0.0459 | 0.9765 | 0.0236 | 0.0097 | 0.0467 |
| **0.40** | 0.1 | 0.0520 | 0.9480 | 0.0494 | 0.8783 | 0.0539 | 0.9461 | 0.0495 | 0.8681 | 0.0174 | 0.9826 | 0.0164 | 0.8783 | 0.0163 | 0.9837 | 0.0163 | 0.8681 |
| | 0.2 | 0.0601 | 0.9400 | 0.0493 | 0.7934 | 0.0598 | 0.9402 | 0.0504 | 0.7891 | 0.0196 | 0.9805 | 0.0147 | 0.7934 | 0.0182 | 0.9819 | 0.0144 | 0.7891 |
| | 0.3 | 0.0601 | 0.9399 | 0.0474 | 0.7082 | 0.0607 | 0.9394 | 0.0472 | 0.7081 | 0.0130 | 0.9871 | 0.0110 | 0.7082 | 0.0139 | 0.9861 | 0.0106 | 0.7081 |
| | 0.4 | 0.0620 | 0.9380 | 0.0456 | 0.6176 | 0.0614 | 0.9387 | 0.0462 | 0.6186 | 0.0118 | 0.9882 | 0.0089 | 0.6176 | 0.0121 | 0.9879 | 0.0087 | 0.6186 |
| | 0.5 | 0.0679 | 0.9322 | 0.0484 | 0.5246 | 0.0665 | 0.9336 | 0.0481 | 0.5265 | 0.0130 | 0.9870 | 0.0088 | 0.5246 | 0.0135 | 0.9865 | 0.0087 | 0.5265 |
| | 0.6 | 0.0689 | 0.9312 | 0.0470 | 0.4290 | 0.0703 | 0.9298 | 0.0470 | 0.4316 | 0.0146 | 0.9855 | 0.0090 | 0.4290 | 0.0148 | 0.9852 | 0.0093 | 0.4316 |
| | 0.7 | 0.0747 | 0.9254 | 0.0487 | 0.3378 | 0.0759 | 0.9241 | 0.0487 | 0.3407 | 0.0150 | 0.9851 | 0.0105 | 0.3378 | 0.0165 | 0.9835 | 0.0106 | 0.3407 |
| | 0.8 | 0.0880 | 0.9120 | 0.0491 | 0.2452 | 0.0905 | 0.9096 | 0.0496 | 0.2476 | 0.0177 | 0.9824 | 0.0123 | 0.2452 | 0.0180 | 0.9820 | 0.0125 | 0.2476 |
| | 0.85 | 0.1112 | 0.8889 | 0.0499 | 0.2010 | 0.1167 | 0.8833 | 0.0504 | 0.2031 | 0.0191 | 0.9810 | 0.0127 | 0.2010 | 0.0199 | 0.9802 | 0.0130 | 0.2031 |
| | 0.9 | 0.3766 | 0.6234 | 0.0484 | 0.1546 | 0.4278 | 0.5723 | 0.0487 | 0.1561 | 0.0203 | 0.9798 | 0.0131 | 0.1546 | 0.0204 | 0.9797 | 0.0134 | 0.1561 |
| | 0.95 | 0.9610 | 0.0391 | 0.0461 | 0.1077 | 0.9679 | 0.0322 | 0.0465 | 0.1089 | 0.0239 | 0.9762 | 0.0123 | 0.1077 | 0.0252 | 0.9748 | 0.0124 | 0.1089 |
| | 0.99 | 0.9967 | 0.0034 | 0.0886 | 0.0714 | 0.9985 | 0.0016 | 0.0681 | 0.0724 | 0.9869 | 0.0131 | 0.0096 | 0.0714 | 0.9919 | 0.0081 | 0.0095 | 0.0724 |

Table 3. (continued)

| | | q=0.95 | | | | | | | | q=0.99 | | | | | | | |
|---|---|---|---|---|---|---|---|---|---|---|---|---|---|---|---|---|---|
| | | NW | | | | LL | | | | NW | | | | LL | | | |
| $\gamma$ | $\rho$ | TDR | M | S | MCE | TDR | M | S | MCE | TDR | M | S | MCE | TDR | M | S | MCE |
| | 0.1 | 0.0555 | 0.9446 | 0.0482 | 0.8801 | 0.0584 | 0.9416 | 0.0494 | 0.8676 | 0.0187 | 0.9813 | 0.0163 | 0.8801 | 0.0190 | 0.9810 | 0.0161 | 0.8676 |
| | 0.2 | 0.0606 | 0.9394 | 0.0468 | 0.8051 | 0.0625 | 0.9376 | 0.0471 | 0.8001 | 0.0163 | 0.9837 | 0.0123 | 0.8051 | 0.0169 | 0.9832 | 0.0120 | 0.8001 |
| | 0.3 | 0.0656 | 0.9345 | 0.0438 | 0.7164 | 0.0651 | 0.9349 | 0.0442 | 0.7139 | 0.0112 | 0.9888 | 0.0082 | 0.7164 | 0.0123 | 0.9878 | 0.0085 | 0.7139 |
| | 0.4 | 0.0730 | 0.9270 | 0.0436 | 0.6261 | 0.0730 | 0.9271 | 0.0444 | 0.6272 | 0.0139 | 0.9862 | 0.0078 | 0.6261 | 0.0139 | 0.9861 | 0.0076 | 0.6272 |
| **0.50** | 0.5 | 0.0791 | 0.9209 | 0.0465 | 0.5392 | 0.0794 | 0.9207 | 0.0460 | 0.5411 | 0.0120 | 0.9880 | 0.0075 | 0.5392 | 0.0120 | 0.9880 | 0.0075 | 0.5411 |
| | 0.6 | 0.0842 | 0.9159 | 0.0457 | 0.4469 | 0.0853 | 0.9147 | 0.0460 | 0.4500 | 0.0145 | 0.9855 | 0.0077 | 0.4469 | 0.0158 | 0.9843 | 0.0079 | 0.4500 |
| | 0.7 | 0.1024 | 0.8977 | 0.0482 | 0.3590 | 0.1034 | 0.8966 | 0.0485 | 0.3614 | 0.0216 | 0.9785 | 0.0108 | 0.3590 | 0.0211 | 0.9790 | 0.0105 | 0.3614 |
| | 0.8 | 0.1387 | 0.8613 | 0.0491 | 0.2705 | 0.1441 | 0.8559 | 0.0494 | 0.2727 | 0.0227 | 0.9774 | 0.0122 | 0.2705 | 0.0237 | 0.9764 | 0.0124 | 0.2727 |
| | 0.85 | 0.2689 | 0.7311 | 0.0485 | 0.2242 | 0.2938 | 0.7062 | 0.0488 | 0.2259 | 0.0249 | 0.9752 | 0.0127 | 0.2242 | 0.0251 | 0.9749 | 0.0129 | 0.2259 |
| | 0.9 | 0.8309 | 0.1691 | 0.0477 | 0.1817 | 0.8576 | 0.1424 | 0.0483 | 0.1834 | 0.0272 | 0.9728 | 0.0127 | 0.1817 | 0.0286 | 0.9715 | 0.0131 | 0.1834 |
| | 0.95 | 0.9782 | 0.0218 | 0.0462 | 0.1367 | 0.9825 | 0.0175 | 0.0468 | 0.1382 | 0.0453 | 0.9548 | 0.0123 | 0.1367 | 0.0475 | 0.9526 | 0.0124 | 0.1382 |
| | 0.99 | 0.9973 | 0.0027 | 0.1085 | 0.1013 | 0.9987 | 0.0014 | 0.0763 | 0.1026 | 0.9922 | 0.0079 | 0.0098 | 0.1013 | 0.9958 | 0.0043 | 0.0099 | 0.1026 |
| | | | | | | | | | | | | | | | | | |
| | 0.1 | 0.0593 | 0.9408 | 0.0471 | 0.8829 | 0.0593 | 0.9408 | 0.0483 | 0.8693 | 0.0181 | 0.9819 | 0.0150 | 0.8829 | 0.0182 | 0.9818 | 0.0156 | 0.8693 |
| | 0.2 | 0.0656 | 0.9345 | 0.0456 | 0.8074 | 0.0666 | 0.9334 | 0.0463 | 0.8025 | 0.0175 | 0.9826 | 0.0117 | 0.8074 | 0.0179 | 0.9822 | 0.0116 | 0.8025 |
| | 0.3 | 0.0760 | 0.9240 | 0.0425 | 0.7231 | 0.0743 | 0.9258 | 0.0427 | 0.7206 | 0.0143 | 0.9858 | 0.0082 | 0.7231 | 0.0153 | 0.9847 | 0.0081 | 0.7206 |
| | 0.4 | 0.0897 | 0.9104 | 0.0431 | 0.6392 | 0.0919 | 0.9081 | 0.0429 | 0.6403 | 0.0156 | 0.9844 | 0.0065 | 0.6392 | 0.0151 | 0.9850 | 0.0065 | 0.6403 |
| **0.60** | 0.5 | 0.1034 | 0.8967 | 0.0451 | 0.5537 | 0.1037 | 0.8964 | 0.0450 | 0.5559 | 0.0150 | 0.9851 | 0.0068 | 0.5537 | 0.0161 | 0.9839 | 0.0067 | 0.5559 |
| | 0.6 | 0.1200 | 0.8801 | 0.0466 | 0.4691 | 0.1216 | 0.8785 | 0.0471 | 0.4716 | 0.0192 | 0.9809 | 0.0076 | 0.4691 | 0.0185 | 0.9815 | 0.0076 | 0.4716 |
| | 0.7 | 0.1523 | 0.8477 | 0.0479 | 0.3776 | 0.1568 | 0.8433 | 0.0481 | 0.3806 | 0.0260 | 0.9741 | 0.0102 | 0.3776 | 0.0270 | 0.9731 | 0.0102 | 0.3806 |
| | 0.8 | 0.3258 | 0.6742 | 0.0490 | 0.2948 | 0.3456 | 0.6544 | 0.0490 | 0.2969 | 0.0328 | 0.9672 | 0.0120 | 0.2948 | 0.0343 | 0.9658 | 0.0123 | 0.2969 |
| | 0.85 | 0.6919 | 0.3081 | 0.0488 | 0.2519 | 0.7295 | 0.2706 | 0.0490 | 0.2539 | 0.0332 | 0.9669 | 0.0127 | 0.2519 | 0.0353 | 0.9647 | 0.0129 | 0.2539 |
| | 0.9 | 0.9305 | 0.0696 | 0.0493 | 0.2109 | 0.9405 | 0.0595 | 0.0496 | 0.2126 | 0.0416 | 0.9584 | 0.0130 | 0.2109 | 0.0430 | 0.9570 | 0.0132 | 0.2126 |
| | 0.95 | 0.9862 | 0.0138 | 0.0466 | 0.1667 | 0.9888 | 0.0112 | 0.0468 | 0.1681 | 0.3103 | 0.6898 | 0.0127 | 0.1667 | 0.3656 | 0.6344 | 0.0127 | 0.1681 |
| | 0.99 | 0.9973 | 0.0028 | 0.1143 | 0.1321 | 0.9987 | 0.0014 | 0.0808 | 0.1333 | 0.9933 | 0.0067 | 0.0104 | 0.1321 | 0.9964 | 0.0036 | 0.0101 | 0.1333 |

Table 3. (continued)

| | | q=0.95 | | | | | | | | q=0.99 | | | | | | | |
|---|---|---|---|---|---|---|---|---|---|---|---|---|---|---|---|---|---|
| | | NW | | | | LL | | | | NW | | | | LL | | | |
| $\gamma$ | $\rho$ | TDR | M | S | MCE | TDR | M | S | MCE | TDR | M | S | MCE | TDR | M | S | MCE |
| **0.70** | 0.1 | 0.0590 | 0.9410 | 0.0466 | 0.8841 | 0.0583 | 0.9417 | 0.0475 | 0.8716 | 0.0189 | 0.9812 | 0.0148 | 0.8841 | 0.0174 | 0.9826 | 0.0151 | 0.8716 |
| | 0.2 | 0.0731 | 0.9270 | 0.0428 | 0.8110 | 0.0777 | 0.9224 | 0.0452 | 0.8062 | 0.0194 | 0.9806 | 0.0105 | 0.8110 | 0.0201 | 0.9799 | 0.0111 | 0.8062 |
| | 0.3 | 0.0858 | 0.9143 | 0.0391 | 0.7361 | 0.0897 | 0.9103 | 0.0388 | 0.7337 | 0.0151 | 0.9850 | 0.0065 | 0.7361 | 0.0152 | 0.9849 | 0.0061 | 0.7337 |
| | 0.4 | 0.1091 | 0.8910 | 0.0417 | 0.6522 | 0.1130 | 0.8870 | 0.0416 | 0.6530 | 0.0167 | 0.9834 | 0.0063 | 0.6522 | 0.0169 | 0.9831 | 0.0059 | 0.6530 |
| | 0.5 | 0.1436 | 0.8564 | 0.0443 | 0.5662 | 0.1457 | 0.8543 | 0.0442 | 0.5687 | 0.0226 | 0.9775 | 0.0069 | 0.5662 | 0.0219 | 0.9781 | 0.0064 | 0.5687 |
| | 0.6 | 0.1863 | 0.8138 | 0.0464 | 0.4848 | 0.1912 | 0.8089 | 0.0466 | 0.4875 | 0.0304 | 0.9697 | 0.0076 | 0.4848 | 0.0301 | 0.9700 | 0.0081 | 0.4875 |
| | 0.7 | 0.2888 | 0.7113 | 0.0478 | 0.4029 | 0.3019 | 0.6982 | 0.0481 | 0.4057 | 0.0419 | 0.9582 | 0.0101 | 0.4029 | 0.0428 | 0.9573 | 0.0102 | 0.4057 |
| | 0.8 | 0.6655 | 0.3345 | 0.0496 | 0.3184 | 0.6926 | 0.3074 | 0.0499 | 0.3206 | 0.0522 | 0.9479 | 0.0123 | 0.3184 | 0.0545 | 0.9455 | 0.0125 | 0.3206 |
| | 0.85 | 0.8755 | 0.1245 | 0.0488 | 0.2770 | 0.8882 | 0.1119 | 0.0492 | 0.2789 | 0.0668 | 0.9333 | 0.0126 | 0.2770 | 0.0696 | 0.9305 | 0.0128 | 0.2789 |
| | 0.9 | 0.9580 | 0.0421 | 0.0481 | 0.2348 | 0.9623 | 0.0377 | 0.0486 | 0.2364 | 0.0977 | 0.9024 | 0.0125 | 0.2348 | 0.1061 | 0.8940 | 0.0127 | 0.2364 |
| | 0.95 | 0.9890 | 0.0111 | 0.0478 | 0.1945 | 0.9909 | 0.0091 | 0.0480 | 0.1958 | 0.9053 | 0.0948 | 0.0128 | 0.1945 | 0.9194 | 0.0807 | 0.0128 | 0.1958 |
| | 0.99 | 0.9979 | 0.0022 | 0.0971 | 0.1609 | 0.9991 | 0.0010 | 0.0756 | 0.1619 | 0.9954 | 0.0047 | 0.0095 | 0.1609 | 0.9974 | 0.0026 | 0.0094 | 0.1619 |
| **0.80** | 0.1 | 0.0593 | 0.9407 | 0.0455 | 0.8853 | 0.0611 | 0.9390 | 0.0466 | 0.8727 | 0.0194 | 0.9807 | 0.0143 | 0.8853 | 0.0183 | 0.9817 | 0.0139 | 0.8727 |
| | 0.2 | 0.0790 | 0.9210 | 0.0411 | 0.8175 | 0.0825 | 0.9175 | 0.0429 | 0.8122 | 0.0181 | 0.9819 | 0.0098 | 0.8175 | 0.0185 | 0.9815 | 0.0098 | 0.8122 |
| | 0.3 | 0.1047 | 0.8953 | 0.0388 | 0.7389 | 0.1041 | 0.8959 | 0.0389 | 0.7378 | 0.0174 | 0.9827 | 0.0062 | 0.7389 | 0.0188 | 0.9813 | 0.0062 | 0.7378 |
| | 0.4 | 0.1434 | 0.8567 | 0.0394 | 0.6645 | 0.1476 | 0.8524 | 0.0394 | 0.6645 | 0.0192 | 0.9809 | 0.0052 | 0.6645 | 0.0194 | 0.9806 | 0.0050 | 0.6645 |
| | 0.5 | 0.2196 | 0.7804 | 0.0441 | 0.5790 | 0.2204 | 0.7796 | 0.0439 | 0.5805 | 0.0316 | 0.9685 | 0.0062 | 0.5790 | 0.0332 | 0.9669 | 0.0062 | 0.5805 |
| | 0.6 | 0.3227 | 0.6774 | 0.0468 | 0.4975 | 0.3313 | 0.6688 | 0.0474 | 0.5001 | 0.0505 | 0.9495 | 0.0082 | 0.4975 | 0.0556 | 0.9444 | 0.0085 | 0.5001 |
| | 0.7 | 0.5218 | 0.4782 | 0.0480 | 0.4193 | 0.5380 | 0.4621 | 0.0484 | 0.4218 | 0.0770 | 0.9231 | 0.0102 | 0.4193 | 0.0817 | 0.9184 | 0.0103 | 0.4218 |
| | 0.8 | 0.8332 | 0.1668 | 0.0499 | 0.3392 | 0.8430 | 0.1571 | 0.0500 | 0.3410 | 0.1268 | 0.8733 | 0.0124 | 0.3392 | 0.1353 | 0.8648 | 0.0127 | 0.3410 |
| | 0.85 | 0.9246 | 0.0755 | 0.0492 | 0.2970 | 0.9304 | 0.0697 | 0.0495 | 0.2989 | 0.1970 | 0.8030 | 0.0129 | 0.2970 | 0.2158 | 0.7843 | 0.0131 | 0.2989 |
| | 0.9 | 0.9662 | 0.0338 | 0.0481 | 0.2565 | 0.9700 | 0.0301 | 0.0483 | 0.2581 | 0.5736 | 0.4264 | 0.0127 | 0.2565 | 0.6296 | 0.3705 | 0.0129 | 0.2581 |
| | 0.95 | 0.9908 | 0.0093 | 0.0479 | 0.2174 | 0.9924 | 0.0077 | 0.0479 | 0.2187 | 0.9632 | 0.0368 | 0.0128 | 0.2174 | 0.9686 | 0.0315 | 0.0129 | 0.2187 |
| | 0.99 | 0.9968 | 0.0033 | 0.0778 | 0.1846 | 0.9988 | 0.0013 | 0.0663 | 0.1857 | 0.9949 | 0.0051 | 0.0098 | 0.1846 | 0.9976 | 0.0024 | 0.0097 | 0.1857 |

Table 3. (continued)

| | | **q=0.95** | | | | | | | | **q=0.99** | | | | | | | |
|---|---|---|---|---|---|---|---|---|---|---|---|---|---|---|---|---|---|
| | | NW | | | | LL | | | | NW | | | | LL | | | |
| $\gamma$ | $\rho$ | TDR | M | S | MCE | TDR | M | S | MCE | TDR | M | S | MCE | TDR | M | S | MCE |
| **0.85** | 0.1 | 0.0612 | 0.9389 | 0.0444 | 0.8881 | 0.0598 | 0.9402 | 0.0453 | 0.8749 | 0.0193 | 0.9808 | 0.0134 | 0.8881 | 0.0174 | 0.9826 | 0.0136 | 0.8749 |
| | 0.2 | 0.0788 | 0.9213 | 0.0405 | 0.8204 | 0.0817 | 0.9183 | 0.0427 | 0.8133 | 0.0180 | 0.9820 | 0.0095 | 0.8204 | 0.0197 | 0.9803 | 0.0098 | 0.8133 |
| | 0.3 | 0.1142 | 0.8859 | 0.0400 | 0.7420 | 0.1162 | 0.8838 | 0.0400 | 0.7405 | 0.0184 | 0.9816 | 0.0068 | 0.7420 | 0.0181 | 0.9819 | 0.0068 | 0.7405 |
| | 0.4 | 0.1650 | 0.8351 | 0.0397 | 0.6644 | 0.1678 | 0.8322 | 0.0403 | 0.6643 | 0.0241 | 0.9759 | 0.0052 | 0.6644 | 0.0240 | 0.9761 | 0.0049 | 0.6643 |
| | 0.5 | 0.2514 | 0.7487 | 0.0441 | 0.5849 | 0.2547 | 0.7454 | 0.0440 | 0.5867 | 0.0383 | 0.9618 | 0.0061 | 0.5849 | 0.0375 | 0.9625 | 0.0062 | 0.5867 |
| | 0.6 | 0.3930 | 0.6071 | 0.0458 | 0.5058 | 0.4016 | 0.5984 | 0.0458 | 0.5080 | 0.0633 | 0.9367 | 0.0075 | 0.5058 | 0.0661 | 0.9339 | 0.0078 | 0.5080 |
| | 0.7 | 0.6272 | 0.3728 | 0.0489 | 0.4252 | 0.6437 | 0.3563 | 0.0490 | 0.4276 | 0.1212 | 0.8788 | 0.0109 | 0.4252 | 0.1285 | 0.8716 | 0.0108 | 0.4276 |
| | 0.8 | 0.8685 | 0.1316 | 0.0502 | 0.3466 | 0.8759 | 0.1242 | 0.0503 | 0.3487 | 0.2351 | 0.7650 | 0.0126 | 0.3466 | 0.2510 | 0.7491 | 0.0127 | 0.3487 |
| | 0.85 | 0.9277 | 0.0723 | 0.0498 | 0.3052 | 0.9319 | 0.0682 | 0.0501 | 0.3070 | 0.4174 | 0.5827 | 0.0136 | 0.3052 | 0.4540 | 0.5461 | 0.0138 | 0.3070 |
| | 0.9 | 0.9718 | 0.0282 | 0.0486 | 0.2661 | 0.9749 | 0.0252 | 0.0491 | 0.2677 | 0.7989 | 0.2011 | 0.0131 | 0.2661 | 0.8245 | 0.1755 | 0.0133 | 0.2677 |
| | 0.95 | 0.9891 | 0.0109 | 0.0482 | 0.2258 | 0.9914 | 0.0086 | 0.0484 | 0.2269 | 0.9695 | 0.0305 | 0.0125 | 0.2258 | 0.9737 | 0.0264 | 0.0127 | 0.2269 |
| | 0.99 | 0.9971 | 0.0030 | 0.0728 | 0.1944 | 0.9992 | 0.0009 | 0.0633 | 0.1954 | 0.9945 | 0.0056 | 0.0100 | 0.1944 | 0.9980 | 0.0020 | 0.0101 | 0.1954 |
| **0.90** | 0.1 | 0.0595 | 0.9405 | 0.0449 | 0.8866 | 0.0597 | 0.9403 | 0.0474 | 0.8721 | 0.0176 | 0.9824 | 0.0135 | 0.8866 | 0.0187 | 0.9814 | 0.0142 | 0.8721 |
| | 0.2 | 0.0825 | 0.9176 | 0.0398 | 0.8212 | 0.0842 | 0.9158 | 0.0411 | 0.8163 | 0.0188 | 0.9812 | 0.0091 | 0.8212 | 0.0183 | 0.9818 | 0.0092 | 0.8163 |
| | 0.3 | 0.1220 | 0.8781 | 0.0380 | 0.7458 | 0.1214 | 0.8786 | 0.0390 | 0.7441 | 0.0198 | 0.9802 | 0.0061 | 0.7458 | 0.0210 | 0.9791 | 0.0064 | 0.7441 |
| | 0.4 | 0.1842 | 0.8158 | 0.0409 | 0.6684 | 0.1896 | 0.8105 | 0.0410 | 0.6687 | 0.0284 | 0.9717 | 0.0059 | 0.6684 | 0.0299 | 0.9702 | 0.0057 | 0.6687 |
| | 0.5 | 0.2950 | 0.7051 | 0.0434 | 0.5909 | 0.3012 | 0.6988 | 0.0435 | 0.5920 | 0.0449 | 0.9551 | 0.0060 | 0.5909 | 0.0463 | 0.9538 | 0.0062 | 0.5920 |
| | 0.6 | 0.4664 | 0.5336 | 0.0458 | 0.5072 | 0.4780 | 0.5221 | 0.0460 | 0.5097 | 0.0932 | 0.9069 | 0.0078 | 0.5072 | 0.0964 | 0.9036 | 0.0081 | 0.5097 |
| | 0.7 | 0.6987 | 0.3014 | 0.0490 | 0.4302 | 0.7090 | 0.2910 | 0.0485 | 0.4325 | 0.1861 | 0.8140 | 0.0109 | 0.4302 | 0.1932 | 0.8068 | 0.0108 | 0.4325 |
| | 0.8 | 0.8821 | 0.1179 | 0.0506 | 0.3525 | 0.8862 | 0.1139 | 0.0507 | 0.3544 | 0.4013 | 0.5988 | 0.0129 | 0.3525 | 0.4241 | 0.5760 | 0.0132 | 0.3544 |
| | 0.85 | 0.9399 | 0.0602 | 0.0492 | 0.3099 | 0.9438 | 0.0563 | 0.0493 | 0.3117 | 0.6535 | 0.3465 | 0.0136 | 0.3099 | 0.6819 | 0.3181 | 0.0138 | 0.3117 |
| | 0.9 | 0.9711 | 0.0290 | 0.0498 | 0.2718 | 0.9730 | 0.0270 | 0.0502 | 0.2733 | 0.8714 | 0.1286 | 0.0130 | 0.2718 | 0.8829 | 0.1171 | 0.0130 | 0.2733 |
| | 0.95 | 0.9900 | 0.0101 | 0.0493 | 0.2329 | 0.9924 | 0.0076 | 0.0497 | 0.2340 | 0.9715 | 0.0285 | 0.0132 | 0.2329 | 0.9751 | 0.0249 | 0.0133 | 0.2340 |
| | 0.99 | 0.9957 | 0.0043 | 0.0649 | 0.2008 | 0.9978 | 0.0022 | 0.0580 | 0.2018 | 0.9929 | 0.0072 | 0.0102 | 0.2008 | 0.9966 | 0.0035 | 0.0103 | 0.2018 |
